# Competing Routes in the Extraction of Lanthanide Nitrates by 1,10-Phenanthroline-2,9-diamides: An Impact of Structure of Complexes on the Extraction

**DOI:** 10.3390/ijms232415538

**Published:** 2022-12-08

**Authors:** Yuri A. Ustynyuk, Nelly I. Zhokhova, Igor P. Gloriozov, Petr I. Matveev, Mariia V. Evsiunina, Pavel S. Lemport, Anton S. Pozdeev, Vladimir G. Petrov, Alexandr V. Yatsenko, Viktor A. Tafeenko, Valentine G. Nenajdenko

**Affiliations:** Department of Chemistry, M. V. Lomonosov Moscow State University, 119991 Moscow, Russia

**Keywords:** lanthanides, N,O-hybrid ligands, complex, DFT calculations, X-ray diffraction

## Abstract

The fact of the fracture of the extraction curve of lanthanides by 1,10-phenanthroline-2,9-diamides is explained in terms of the structure of complexes, solvent extraction data and quantum chemical calculations. The solvent extraction proceeds in two competing directions: in the form of neutral complexes **L**Ln(NO_3_)_3_ and in the form of tight ion pairs {[**L**Ln(NO_3_)_2_ H_2_O]^+^ (NO_3_^−^).

## 1. Introduction

In recent decades, the number of critical technologies based on the use of lanthanides (Ln) and their compouns have been rapidly growing in the world economy. As a result, the production and consumption of Ln in the world over the past 10 years has increased by more than five times [1,2]. Therefore, the improvement of existing and the development of new highly efficient methods for the production of high-purity Ln remains a task of paramount importance. The existing technologies for the isolation and separation of Ln are based on extraction in the two-phase organic solvent/water systems by the highly selective extractants, which are organic ligands capable of forming complexes with Ln cations that are soluble in the organic phase [3]. The same process is used to solve the equally difficult and important task of separating Ln and “minor actinides” (MAn, americium, neptunium and curium). The development of nuclear fuel cycle technologies for nuclear power production is increasingly establishing itself as an economically viable way to generate electricity not associated with the emission of carbon dioxide into the atmosphere [3,4,5,6,7,8]. A significant complication of the intragroup separation of Ln, as well as the separation of Ln and Man, is due to the exceptional similarity of their chemical properties. These elements are characterized by the same oxidation state +3; the ionic radii of MAn and Ln are close; when moving from La^3+^ to Lu^3+^ in the lanthanide series and from Ac^3+^ to Lr^3+^ in the actinide series, only a gradual filling of the inner 4f and 5f electron shells takes place, and a decrease in the ionic radii of the cations (Ln and Ac contraction) occur. At the same time, the structure of the outer electron shell of Ln ions [Xe]4f^n^ and Ac ions [Rn]5f^n^ (*n* = 0–14) is preserved, which determines the similarity of their chemical behavior [9].

**Figure 1:**
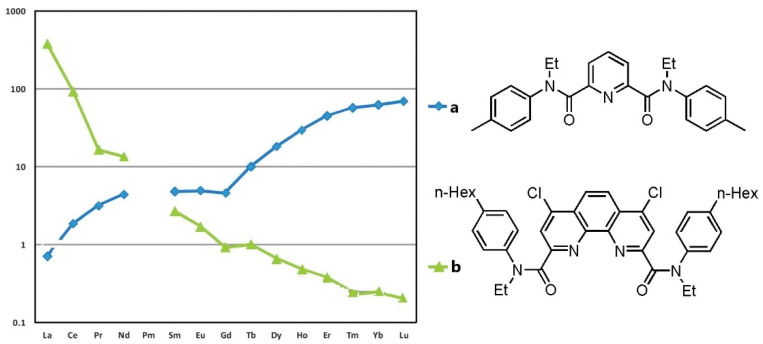
Distribution ratios ***D*** of Ln^3+^ in the system 3 M HNO_3_/3-nitrobenzotrifluoride (F-3) using (**a**) *N*,*N*′-diethyl-*N*,*N*′-di(p-tolyl)-pyridine-2,6-dicarboxamide and (**b**) *N*,*N*′-diethyl-*N*,*N*′-di(4-n-hexylphenyl)-4,7-dichloro-1,10-phenantroline-2.9-dicarboxamide (adapted from [8]).

Among the extractants used to separate Ln and An, most are neutral donor ligands containing oxygen, nitrogen or sulfur atoms carrying lone electron pairs. They bind Ln and An cations by a standard coordination (solvation) mechanism. In the series of Ln during extraction, two types of dependence of the distribution coefficients of Ln^3+^ cations between the aqueous and organic phases ***D*** on the atomic number of the cation ***Z*** (***D*** vs. ***Z***) are usually observed. Due to the Ln contraction, when moving from La to Lu, the radius of the cation decreases, and the charge density on it increases. Since metal-ligand bonds in Ln and An complexes are predominantly electrostatic (ionic), the affinity of donor extractants for the cation increases. As a result, the distribution coefficients ***D*** increase in the series from La to Lu (“positive sequence ***D*** vs. ***Z***”). However, the dependence of ***D*** vs. ***Z*** can change for polydentate donor ligands depending on several factors. For example, in the extraction of Ln with pyridine-2,6-dicarboxylic acid diamides [10], positive sequences ***D*** vs. ***Z*** are observed (Equation (1), Figure 1a). However, tetradentate diamides of 1,10-phenanthroline-2,9-dicarboxylic acid, containing one aryl and one alkyl substituent in the amide fragments, with a larger coordination cavity, exhibit a higher affinity for the early Ln with the larger ionic radii. An inversion of the “***D*** vs. ***Z***” dependence is observed for these ligands (“negative sequence ***D*** vs. ***Z***”, Equation (1), Figure 1b) [11].

{[Ln(H_2_O)_n_]^3+^}_aq_ + 3 (NO_3_^−^)_aq_ + **L**_org_ = {**L**Ln(NO_3_)_3_}_org_ + {(H_2_O)_n_}_aq_ (1)

It should be noted that “***D*** vs. ***Z***” curves are rarely monotonic. The appearance of inflection points and other features on them may be a consequence of the manifestation of the “tetrad effect” [12,13], the occurrence of spatial tensions in the resulting complexes if the bulky ligands are used (dome-shape dependence), as well as a consequence of a change in the extraction mechanism when moving along the series of cations. The form of dependence can be affected by the conformational properties of the ligand [14] as well as the additional coordination of water molecules on the outer sphere of the extractable complex [15]. In some cases, changes in the composition of the extraction system and/or extraction conditions lead to a transition from a positive ***D*** vs. ***Z*** sequence to a negative one [16,17]. The incorporation of alkyl substituents into the aryl rings of *N*,*N*′-dialkyl-*N*,*N*′-diaryl-1,10-phenanthroline-2,9-dicarboxamides also affects the shape of the dependence ***D*** vs. ***Z*** in the extraction of Ln [18]. Elucidation of the reasons for such features is of considerable interest. These peculiarities should be taken into account when designing new highly selective ligands. However, the number of works that deal with this problem remains small in the literature.

Diamides of 1,10-phenanthroline-2,9-dicarboxylic acid are highly selective ligands used both for the inter- and intragroup extraction separation of Ln and An. Their undoubted advantages are also high radiation and hydrolytic stability, fast extraction kinetics and compliance with the requirements of C, H, N and O, which opens real prospects for their use in technologies [3,4,5,6,7,8,19,20,21,22,23]. Synthetic methods for the structural modification of such ligands by introducing various substituents into the phenanthroline core and into amide moieties to finely tune the properties of the ligand to practical requirements are well developed. Thus, detailed information on how such modifications affect the extraction properties of the ligand (the study of structure/property relationships) is especially important.

**Figure 2:**
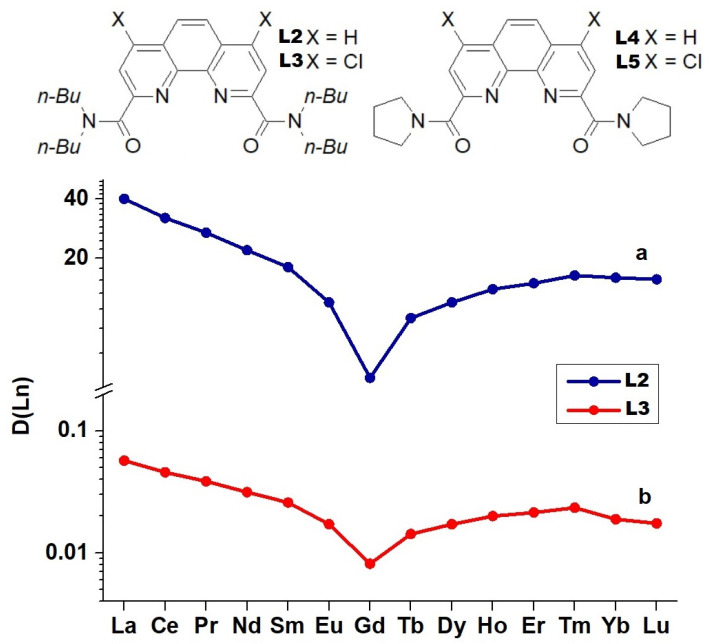
V-shaped dependence ***D*** vs. ***Z*** observed for Ln extraction by (**a**) 0.5 M **L2** from 3 M HNO_3_ and (**b**) 0.5 M **L3** from 5 M HNO_3_.

It was first noticed [18] that when *N*,*N*,*N*′,*N*′-tetra(*n*-butyl)-1,10-phenanthroline-2,9-dicarboxylic acid diamide (**L2**) was used as an extractant, the negative sequence ***D*** vs. ***Z*** is violated and ceases to be monotonic. During extraction from 3 M HNO_3_, the ***D*** ratios decrease when moving from La to Gd, and then begin to grow again. For ligand (**L2**), this result was reproduced in our work [20]. Similar dependencies were obtained for ligands **L3**–**L5** (Figure 2). 

Japanese authors synthesized *N*,*N*,*N′*,*N′*-tetra(*n*-dodecyl)-1,10-phenanthroline-2,9-dicarboxylic acid diamide and studied the extraction of Ln from nitric acid solutions, varying the concentration of HNO_3_ in the range from 0.2 M to 10 M [21]. At an HNO_3_ concentration of 3 M, the character of changes in the ratios ***D*** when moving along the Ln series in all solvents coincides with that found in [20,21]: a decrease in ***D*** when moving from La to Gd followed by an increase from Gd to Lu. These results also show the V-shaped character of ***D*** vs. ***Z*** dependence with a minimum on the gadolinium atom. This intriguing phenomenon has not yet been explained, but its understanding is quite important for the development of new selective ligands for the separation of Ln and An. In the present work, we try to solve this interesting and important problem.

## 2. Results and Discussion

Analysis of the numerous structures of Ln nitrate complexes with different neutral donor polydentate ligands makes it possible to clearly reveal two trends when moving along the series from lanthanum to lutetium. First, there is a decrease in the coordination number for the cations at the end of the series. This reveals a change in the coordination mode of one of the nitrate anions (transition з^2^ → η^1^). For complexes of diamides of 1,10-phenanthroline-2,9-dicarboxylic acid **L**Ln(NO_3_)_3_, such a transition was found by us for the lutetium complex [**L2**Lu(η^2^-NO_3_)_2_(η^1^-NO_3_)] [19] and was also observed for tetradentate N,O-hybrid phenanthroline-derived organophosphorus extractants [22]. For other polydentate ligands, e.g., for 2,2′,6,2″-terpyridine, one of the nitrates can be displaced into the outer coordination sphere by a more compact water molecule with the formation of ion pairs [**L**Ln(NO_3_)_2_(H_2_O)]^+^(NO_3_)^−^ [23,24]. Although complexes of this type have not yet been described for diamides **L**, the existence of equilibrium in solution between a neutral complex and a tight ion pair (Equation (2) seems very likely.

**L**Ln(NO_3_)_3_ + H_2_O ⇄ {[LLn(NO_3_)_2_(H_2_O)]^+^(NO_3_)^−^} (2)

In this regard, it can be assumed that the appearance of V-shaped dependence ***D*** vs. ***Z*** in the extraction of Ln with diamides **L2**–**L5** can be explained by the existence of equilibrium (2) in aqueous solutions. The early Ln are predominantly extracted in the form of neutral complexes **L**Ln(NO_3_)_3_, while the heavy Ln cations are extracted predominantly as tight ion pairs {[LLn(NO_3_)_2_(H_2_O)]^+^(NO_3_)^−^}, in which the 1:1 metal/ligand stoichiometry is retained.

To better understand the reasons why ***D*** vs. ***Z*** patterns for tetradentane diamides **L2**–**L5** are V-shaped, we performed DFT modeling of a hypothetical equilibrium (3) for ligands **L2** and **L4**. The calculations were carried out using the ab initio PBE functional and TZ full-electron relativistic basis sets using the PRIRODA program [25,26]. A detailed description of the calculation methods is given in Supplementary Materials. The calculation results are presented in full in Tables S2–S6 in Supplementary Materials.

[**L**Ln]^3+^ + NO_3_^−^ = [**L**Ln(NO_3_)]^2+^ + NO_3_^−^ = [**L**Ln(NO_3_)_2_]^+^ + NO_3_^−^ = **L**Ln(NO_3_)_3_ (3)

**Figure 3:**
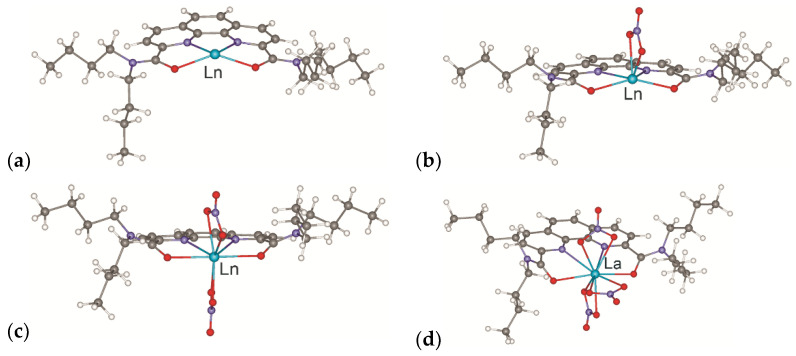

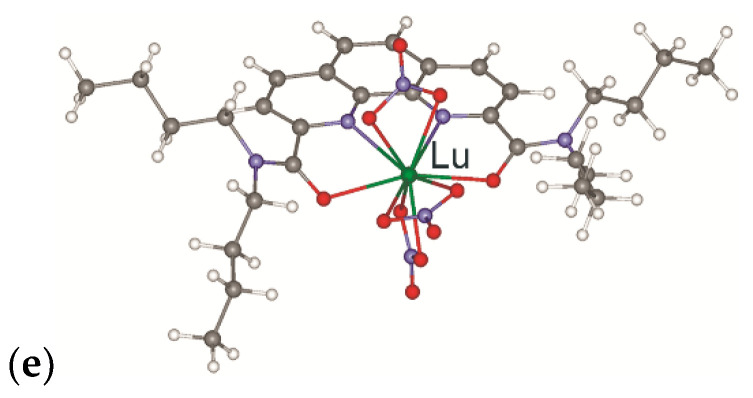
Structures of [**L2**Ln]^3+^ (**a**), [**L2**Ln(NO_3_)]^2+^ (**b**), and [**L2**Ln(NO_3_)_2_]^+^ (**c**) cations (Ln = La–Lu); structures of **L2**La(NO_3_)_3_ (**d**) and **L2**Lu(NO_3_)_3_ (**e**) complexes according to calculation data.

The [**L**Ln]^3+^, [**L**Ln(NO_3_)]^2+^, and [**L**Ln(NO_3_)_2_]^+^ cations have the similar structures for both ligands. In contrast, the structures of the neutral **L**Ln(NO_3_)_3_ complexes for cations of the beginning of the series (La^3+^) and the end of the series (Lu^3+^) are different. The structures of such complexes calculated for ligand **L2** are shown in Figure 3. According to both the simulation data (Figure 3c) and the experimental results [19], one of the nitrates in the lutetium complex becomes monodentate. 

**Figure 4:**
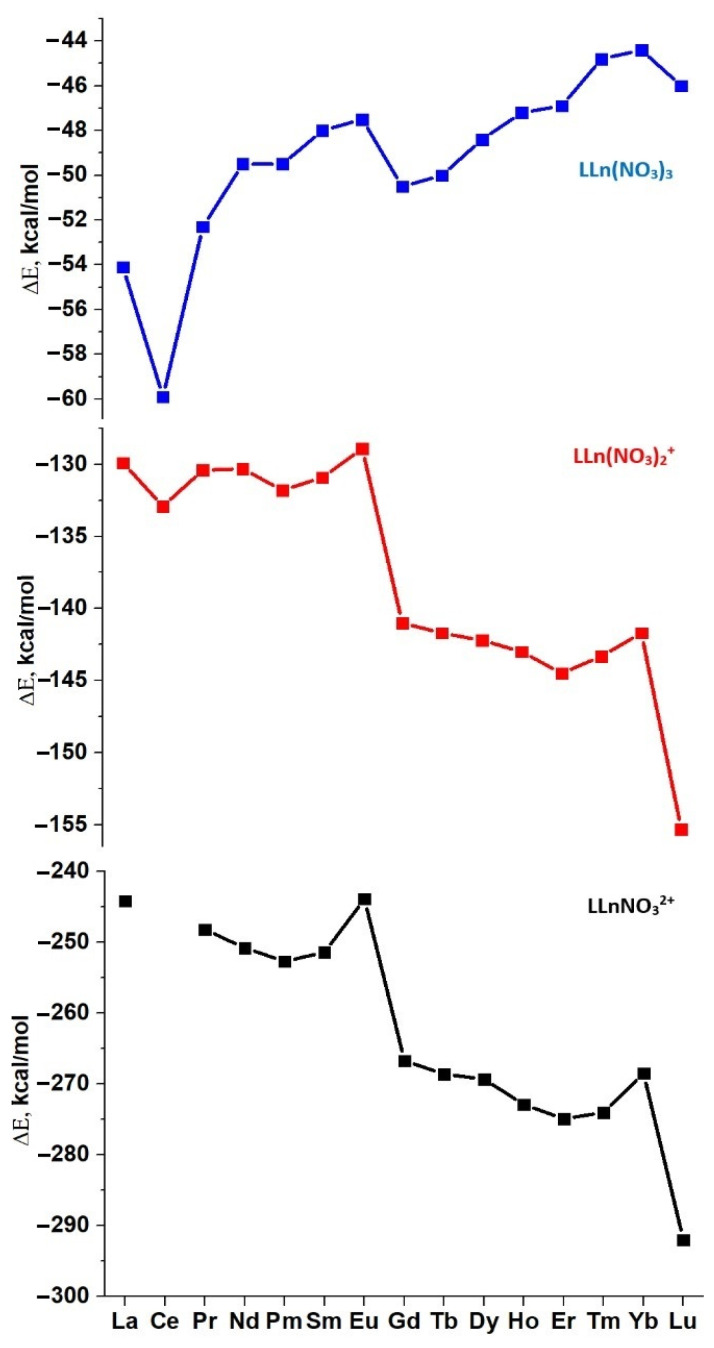
Calculated binding energies ∆E for successive addition of nitrate anions to the [**L2**Ln]^3+^, [**L2**Ln(NO_3_)]^2+^, [**L2**Ln(NO_3_)_2_]^+^, and **L2**Ln(NO_3_)_3_ complexes (gas phase conditions).

**Figure 5:**
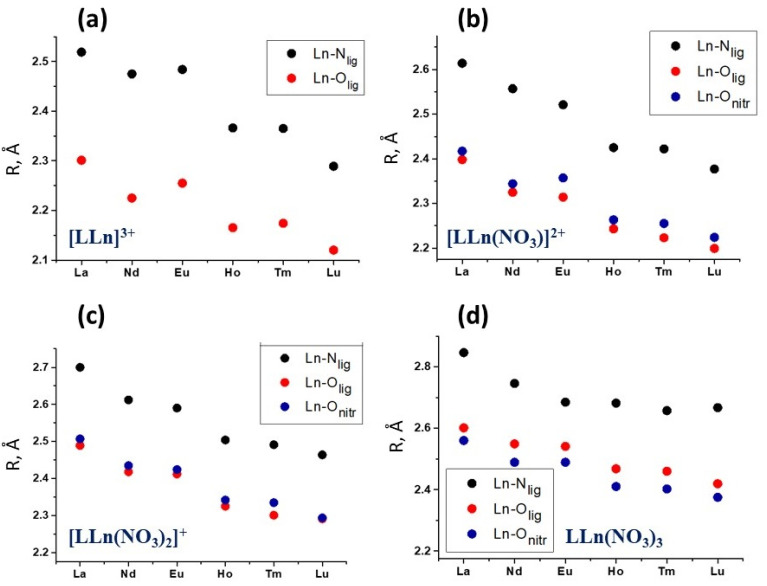
Average calculated bond lengths in the (**a**) [**L2**Ln]^3+^, (**b**) [**L2**Ln(NO_3_)]^2+^, (**c**) [**L2**Ln(NO_3_)_2_]^+^ and (**d**) **L2**Ln(NO_3_)_3_ complexes.

Changes in the binding energies ∆E in the complexes of **L2** are shown in Figure 4. The corresponding changes in bond lengths in the coordination node are given in Figure 5. When adding anions to the complexes [**L**Ln]^3+^ and [**L**Ln(NO_3_)]^2+^, the binding energies ∆E rapidly increase with an increasing atomic number of the cation. The addition of the third anion to the [**L**Ln(NO_3_)_2_]^+^ cation, which leads to the formation of the neutral complex **L**Ln(NO_3_)_3_, becomes less and less favorable as one moves along the Ln series from lanthanum to lutetium. Obviously, this effect is explained by the mutual repulsion between the bulky bidentate nitrates in the first coordination sphere of the **L**Ln(NO_3_)_3_ complexes formed, which rapidly increases as the cation radius decreases.

Considering the dependence of bond lengths in the coordination unit (Figure 5 and Table 1), one can see that during the transition from [**L**Ln]^3+^, [**L**Ln(NO_3_)]^2+^, [**L**Ln(NO_3_)_2_]^+^ cations to **L**Ln(NO_3_)_3_ all bonds are elongated.

Since the resulting **L**Ln(NO_3_)_3_ complexes pass into the organic phase during extraction, the calculations of the **L2**Ln(NO_3_)_3_ complexes for a limited number of metals were performed, taking into account the effects of the polar solvent in the continuum approximation at the SMD level [27]. Since SMD is not parameterized for F-3, the parameters of acetone were used, which dielectric constant is close to that of F-3 (20.5 vs. 22.0, respectively). 

The calculations were performed using the ORCA program [28,29] employing the hybrid functional B3LYP [30], def2-TZVP basis set [31] and the effective core potentials for La and other Ln with 46 and 28 in-core electrons, respectively [32]. Details of calculations are given in the Supplementary Materials. Two effects are clearly manifested upon transition to a polar solvent. The energies of the addition of the third NO_3_^−^ anion to the [**L**Ln(NO_3_)_2_]^+^ cation sharply decrease. This effect is to be expected since, as mentioned above, the metal-ligand coordination bonds in the complexes under study are predominantly of an electrostatic (ionic) nature. However, in this case, only the most polar Ln-O_nitr_ bonds are significantly lengthened (weakened), while the less polar Ln-N_lig_ and Ln-O_lig_ bonds are noticeably shortened. It is noteworthy that in calculations accounting for the effects of a polar solvent, the transition of one of the bidentate nitrates to the monodentate one occurs earlier, already for holmium. In accordance with this, ongoing from Eu to Ho, the binding energy of the third ligand somewhat increases.

We estimated the relative stability of complexes {[**L**Ln(NO_3_)_2_H_2_O]^+^NO_3_^−^} (**A**) and **L**Ln(NO_3_)_3_·H_2_O (**B**), respectively, by calculating the energies of their formation using Equations (4) and (5):

[**L**Ln(NO_3_)_2_]^+1^ + (NO_3_^−^·H_2_O)^−^ → {[**L**Ln(NO_3_)_2_H_2_O]^+^NO_3_^−^} (**A**) (4)

[**L**Ln(NO_3_)_2_]^+1^ + (NO_3_·H_2_O)^−^ → [**L**Ln(NO_3_)_3_]·H_2_O (**B**) (5)

**Figure 6:**
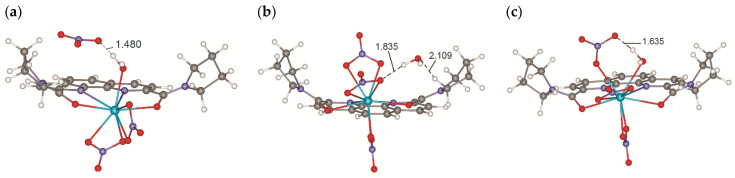
Structures of (**a**) {[**L4**Ln(NO_3_)_2_H_2_O]^+^NO_3_^−^} (**A**), (**b**) [**L4**Ln(NO_3_)_3_]·H_2_O (**B**) and (**c**) [**L4**Ln(NO_3_)_3_H_2_O] (**C**) complexes according to calculation data.

Pyrrolidine-derived ligands **L4** and **L5**, being more compact and conformationally rigid than the butyl groups in **L2** and **L3**, were chosen as ligands at this stage. This significantly reduces the number of local minima on the PES, reduces calculation time and yields more reliable results. The structures of the resulting complexes with the ligand **L4** are shown in Figure 6. In complexes (**A**) (Figure 6a), the nitrate anion located in the outer coordination sphere is hydrogen bonded to a water molecule coordinated by the metal. In complexes (**B**) (Figure 6b), the water molecule in the outer coordination sphere of the cation is hydrogen bonded to one of the nitrate oxygen atoms coordinated onto the metal. Similar structures are described in [33]. In addition to the complexes of these types, there are other minima on the PES of the systems under study. In particular, for the cations of the early Ln, another structure was found (Figure 6c, complexes **C**), where the water molecule enters the first coordination sphere of the cations, and one of the nitrates becomes monodentate, which lies somewhat lower in energy. However, the relative stability of such structures for the cations of the middle and end of the Ln series drops sharply; thus, their formation upon extraction seems improbable.

**Figure 7:**
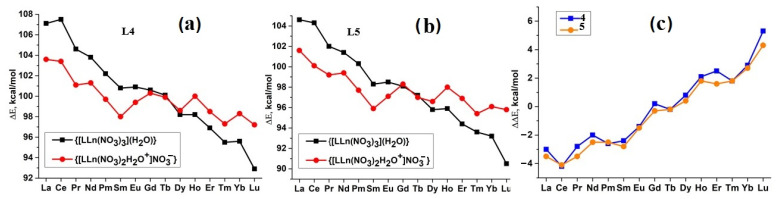
Calculated ∆E of formation of complexes (**B**) and (**A**) with **L4** (**a**) and **L5** (**b**); ∆∆E_B,A_ for Ln cations (**c**).

The formation energies ∆E of tight ionic pairs (Figure 7a) and hydrated neutral complexes (Figure 7b) for the whole series of Ln with ligands **L4** and **L5** and the differences of these energies ∆∆E = ∆E_B_ − ∆E_A_ are presented in Supplementary Materials and in Figure 7. For chosen ligands, both ∆E_C_ and ∆E_B_ decrease from La^3+^ to Lu^3+^ (Figure 7a,b). As obvious, the neutral complexes (**B**) are more stable for cations with the larger radii, i.e., at the beginning of the series, while ion pairs (**A**) are more stable for the late Ln (Figure 7c). The intersection points of the two dependences (∆∆E = 0) are observed at Gd and Tb. Similar trends were also found for ligands **L2** and **L3**.

**Figure 8:**
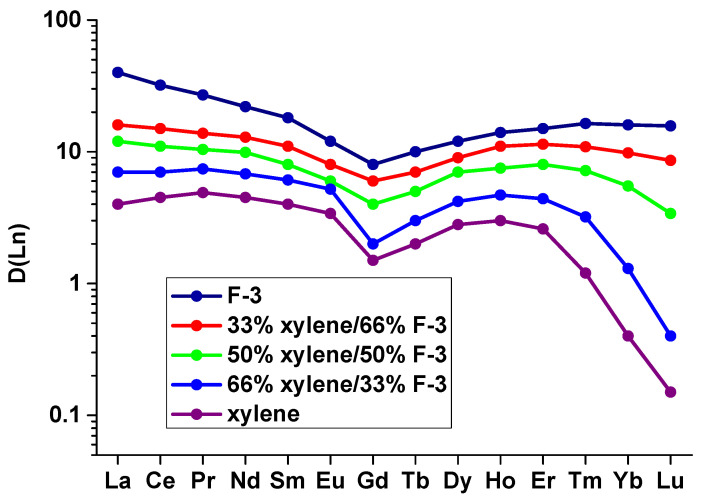
Distribution ratios ***D*** of Ln cations during their extraction from 3 M HNO_3_ with ligand **L2** (0.7 mol/L) in pure F-3 and its mixtures with para-xylene.

The trends in ∆E and ∆∆E_B,A_ changes for ligands **L4** and L**5**, found in the calculations, are consistent with the aforementioned hypothesis that the Ln^3+^ cations at the beginning of the series form neutral **L**Ln(NO_3_)_3_ complexes during extraction, while the heavy cations form {[**L**Ln(NO_3_)_2_H_2_O]^+^NO_3_^−^} ion pairs. To confirm this hypothesis, we carried out extraction experiments changing the polarity of the organic phase. There is no doubt that the decrease in the solvent polarity will destabilize the ion pair relative to the neutral complex. As a result, a change in the polarity of the organic phase should significantly affect the values of the distribution ratios ***D*** during the extraction of Ln by the ligands **L2**–**L5**. The results of Ln extraction experiments from 3 M HNO_3_ using 3-nitrobenzotrifluoride (F-3, ε = 22), p-xylene (ε = 2.26) and their mixtures confirmed this statement (see Figure 8). The details of extraction experiments and the values of ***D*** for these systems are given in the Supplementary Materials. These extraction data are in complete agreement with the results of the DFT simulation presented above. A decrease in polarity leads to a strong decrease in the distribution coefficients for heavy Ln.

**Figure 9:**
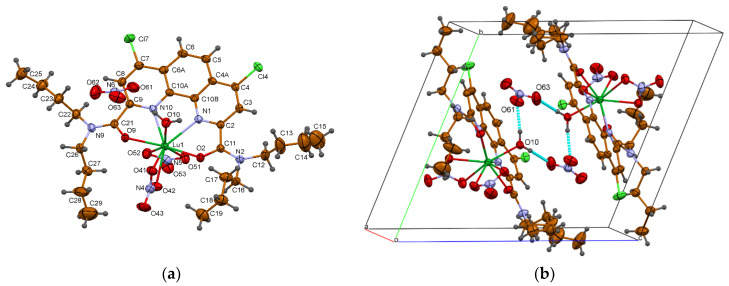
X-ray structure of cationic complex of lutetium nitrate with ligand **L3** (**a**) and of the {C_30_H_42_Cl_2_LuN_6_O_9_^+^}_2_{NO_3_^−^}_2_ aggregate (**b**). H-bonds are presented by dashed lines.

So far, no X-ray structures of ion pairs similar to those depicted in Figure 6a have been published in the literature for phenantroline diamides. However, a number of cationic complexes of Ln are known for terpyridines and similar ligands [23,24]. Our attempt to obtain a complex with a structure of tight ion pair for **L3** with lutetium nitrate appeared to be successful. Single crystals of {[**L3**Lu(NO_3_)_2_(H_2_O)]^+^(NO_3_)^−^} were obtained upon slow isothermal (25 °C) recrystallization of powder substance **L3**Lu(NO_3_)_3_ from wet acetonitrile. The crystal structure was determined with SHELXT [34] and refined with SHELXL [35]. The experimental and crystallographic details are given in the Supplementary Materials. The crystal structure of this complex contains centrosymmetric aggregates {C_30_H_42_Cl_2_LuN_6_O_9_^+^}_2_{NO_3_^−^}_2_ with complex cations and nitrate anions are connected to each other by the medium-strong hydrogen bonds (Figure 9). This is the first example of a cationic Ln complex of diamides **L** having the structure of tight ion pair [**L**Ln(NO_3_)_2_H_2_O]^+^NO_3_^−^ with the nitrate anion in the outer coordination sphere.

## 3. Materials and Methods

Ar-saturated solvents, purified and dried using standard techniques, were used. Lutetium trinitrate hydrate Lu(NO_3_)_3_∙xH_2_O was purchased from Sigma-Aldrich, Co. (St. Louis, MO, USA) and used without further purification. Water content x in lutetium nitrate was determined as x = 3. CDCl_3_ for NMR analysis was purchased from Cambridge Isotope Laboratories, Inc. (Andover, MA, USA) and used without further purification. 3-Nitrobenzotrifluoride (“F3”) analytical grade was purchased from Rhodia (France) and was used as a solvent in the extraction experiments without further purification. *N*^2^,*N*^2^,*N*^9^,*N*^9^-tetrabutyl-1,10-phenanthroline-2,9-dicarboxamide (**L2**) was synthesized according to procedure described in [18]. *N*^2^,*N*^2^,*N*^9^,*N*^9^-tetrabutyl-4,7-dichloro-1,10-phenanthroline-2,9-dicarboxamide (**L3**) was prepared according to known procedure [36]. Pyrrolidin-1-yl(9-(pyrrolidin-1-ylcarbonyl)-1,10-phenanthrolin-2-yl)methanone (**L4**) was prepared according to known procedure [37]. (4,7-Dichloro-9-(pyrrolidin-1-ylcarbonyl)-1,10-phenanthrolin-2-yl)(pyrrolidin-1-yl)methanone (**L5**) was obtained by the procedure described in [20]. ^1^H NMR spectrum was recorded using standard 5 mm sample tube on Agilent 400-MR spectrometer (Agilent Technologies, Santa Clara, CA, USA) with operating frequency of 400.1 MHz. IR spectrum was recorded on FTIR spectrometer Nicolet iS5 (Thermo Fisher Scientific, Waltham, MA, USA) using an internal reflectance attachment with diamond optical element—attenuated total reflection (ATR) with 45° angle of incidence. Resolution 4 cm^−1^, the number of scans is 32. HRMS ESI—mass spectra were recorded on the MicroTof Bruker Daltonics and Orbitrap Elite instruments (Shimadzu, Kyoto, Japan). Single crystals of [(**L3**)•Lu(NO_3_)_2_•H_2_O]^+^•NO_3_^−^ were obtained upon slow isothermal (25 °C) recrystallization of powder substance (**L3**)•Lu(NO_3_)_3_ from acetonitrile. X-ray diffraction data for [(**L3**)•Lu(NO_3_)_2_•H_2_O]^+^•NO_3_^−^ were collected at 120 K with a Bruker APEXII DUO CCD diffractometer; using graphite monochromated Mo-Kα radiation (λ = 0.71073 Å). Using Olex2^i^, the structure was solved with the ShelXT structure solution program using Intrinsic Phasing and refined with the XL refinement package using Least-Squares minimization against F^2^ in anisotropic approximation for non-hydrogen atoms []. Positions of hydrogen atoms were calculated, and they were refined in isotropic approximation within the riding model. Crystal data and structure refinement parameters for [(**L3**)•Lu(NO_3_)_2_•H_2_O]^+^•NO_3_^−^ are given in Supplementary Materials, Table S1. CCDC 2174096 contains the supplementary crystallographic data for this paper.

### 3.1. Solvent Extraction Experiments

Extraction experiments were performed in 1.5 mL polypropylene vials, and the volume of both the organic and aqueous phases was 0.5 mL. Moreover, 0.7 M solutions of ligands in F-3 or F-3/para-xylene mixture were used as organic phases. Aqueous phases were solutions of 3 mol∙L^−1^ nitric acid with Ln nitrates at a concentration of 10^−4^ mol∙L^−1^. The contact time was 30 min, and shaking of the samples was carried out in a vortex shaker with an air thermostat at 25 ± 1 °C. After being thoroughly shaken, the samples were centrifuged, and 0.35 mL of aqueous solution was taken for analysis. Lanthanum and Ln series contents were determined by ICP-OES (Agilent ICP-OES 720). The concentration in the organic phase was calculated as the difference between the initial solution and equilibrium aqueous phase. Distribution ratios (D) were determined as the ratio of the concentration in organic and aqueous phases. All of the extraction experiments were repeated three times.

### 3.2. Synthesis and Analytical Data

*N*^2^,*N*^2^,*N*^9^,*N*^9^-tetrabutyl-4,7-dichloro-1,10-phenanthroline-2,9-dicarboxamide lutetium trinitrate (**L3**)•Lu(NO_3_)_3_. A solution of 0.1 mmol of lutetium nitrate in 1 mL of dry acetonitrile was added dropwise to a solution of 0.1 mmol of ligand (2) in 1 mL of chloroform. After that, the reaction mixture was concentrated in vacuo (~20 Torr) to 1/5 of the initial volume and treated with 2 mL of diethyl ether. The resulting precipitate of the complex was filtered, washed with a fresh portion of ether and dried in air to constant mass. Yield 89.6 mg (97%). Yellowish powder. T_decomp_. 207 °C. ^1^H NMR (400 MHz, CDCl_3_) δ 8.47 (s, 2H), 8.16 (s, 2H), 3.79 (m, 4H), 3.67 (m, 4H), 1.99 (p, *J* = 7.9 Hz, 4H), 1.81 (p, *J* = 7.9 Hz, 4H), 1.55 (h, *J* = 7.4 Hz, 4H), 1.44 (h, *J* = 7.4 Hz, 4H), 1.10 (t, *J* = 7.4 Hz, 6H), 1.01 (t, *J* = 7.4 Hz, 6H); IR (ν, cm^−1^) 1607 (CO), 1494, 1467, 1291 (ONO_2_); HRMS (ESI–TOF) (*m*/*z*) [(**L3**)•Lu(NO_3_)_2_]^+^ calcd for C_30_H_40_Cl_2_LuN_6_O_8_ 857.1687, found 857.1641.

## 4. Conclusions

In summary, the results of theoretical calculations, solvent extraction experiments and X-ray data convincingly confirmed the idea that two types of complexes can be formed during the extraction of Ln cations from nitric acid solutions using 1,10-phenanthroline 2,9-diamides. The Ln at the beginning of the row preferably does form neutral complexes **L**Ln(NO_3_)_3_ with phenanthroline diamides containing alkyl or cycloalkyl substituents at the amide N-atoms. In contrast, heavy Ln is extracted mainly as tight ion pairs [**L**Ln(NO_3_)_2_H_2_O]^+^NO_3_^−^}. As a result, the extraction of Ln cations in such systems proceeds along two competing directions, the relative contributions of which change as one moves from lanthanum to lutetium. The complex formation constants in both directions have minimum values for cations in the middle of the row. These data permit an explanation of V-shape dependences ***D*** vs. ***Z*** for extractions of Ln. The switch from neutral to cationic complexes is in agreement with a tendency to minimize strain in the coordination sphere caused by the Ln contraction. Most probably, this effect is rather general and can take place for the extraction of Ln with other N-donor heterocyclic ligands. A more detailed address of this problem will be presented elsewhere soon.

## Figures and Tables

**Table 1:** Binding energies of the third nitrate anion to the [**L2**Ln(NO_3_)_2_]^+^ cation ∆E (kcal/mol) and the average bond lengths (Å) in the coordination site of the [**L2**Ln(NO_3_)_3_] complexes, calculated in the gas phase approximation and in a polar solvent (in parentheses).

Ln	∆E, kcal/mol	Bond Lengths, Å		
		Ln-N_lig_	Ln-O_lig_	Ln-O_nitr_
La	−54.1 (−31.5)	2.841 (2.765)	2.594 (2.544)	2.561 (2.647)
Nd	−49.5 (−28.2)	2.750 (2.684)	2.547 (2.468)	2.488 (2.570)
Eu	−47.5 (−24.7)	2.684 (2.630)	2.538 (2.442)	2.487 (2.531)
Ho	−47.2 (−25.6)	2.688 (2.575)	2.463 (2.378)	2.409 (2.455 ^1^, 2.355 ^2^)
Tm	−44.8 (−19.0)	2.680 (2.531)	2.454 (2.351)	2.399 (2.433 ^1^, 2.321 ^2^)
Lu	−46.0 (−17.7)	2.664 (2.524)	2.414 (2.328)	2.371 ^1^, 2.432 ^2^ (2.413 ^1^, 2.288 ^2^)

^1^ Average Ln-O_nitr_ distance for bidentate nitrate ligands. ^2^ Ln-O_nitr_ distance for monodentate nitrate ligand.

## Data Availability

Samples of the compounds are not available from the authors.

## Supplementary Materials

The supporting information can be downloaded at: https://www.mdpi.com/article/10.3390/ijms232415538/s1.
