# Supplementary material for: Competing Routes in the Extraction of Lanthanide Nitrates by 1,10-Phenanthroline-2,9-diamides: An Impact of Structure of Complexes on the Extraction
Source: Int J Mol Sci. 2022 Dec 8;23(24):15538. doi: 10.3390/ijms232415538 (PMC9779341; doi:10.3390/ijms232415538)
Supplement: Supplementary file 1 [file ijms-23-15538-s001.zip › ijms-2047837-supplementary.pdf]

Electronic Supplementary Information

Competing routes in the extraction of lanthanide nitrates by 1,10-phenanthroline-2,9-dicarboxylic acid diamides: an impact of structure of complexes on the extraction

Yuri A. Ustynyuk, Nelly I. Zhokhova, Igor P. Gloriozov, Petr I. Matveev, Mariia V. Evsiunina, Pavel S. Lemport, Anton S. Pozdeev, Vladimir G. Petrov, Alexandr V. Yatsenko, Viktor A. Tafeenko and Valentine G. Nenajdenko *

Department of Chemistry, M.V. Lomonosov Moscow State University, E-mail: nenajdenko@gmail.com

Table of contents

1. NMR and IR spectra drawings……………………………………………………………..S2-S3

2. X-ray diffraction data……………………………………………………………………….S4

3. Theoretical computations…………………………………………………………….S5-S349

1. NMR and IR spectra drawings

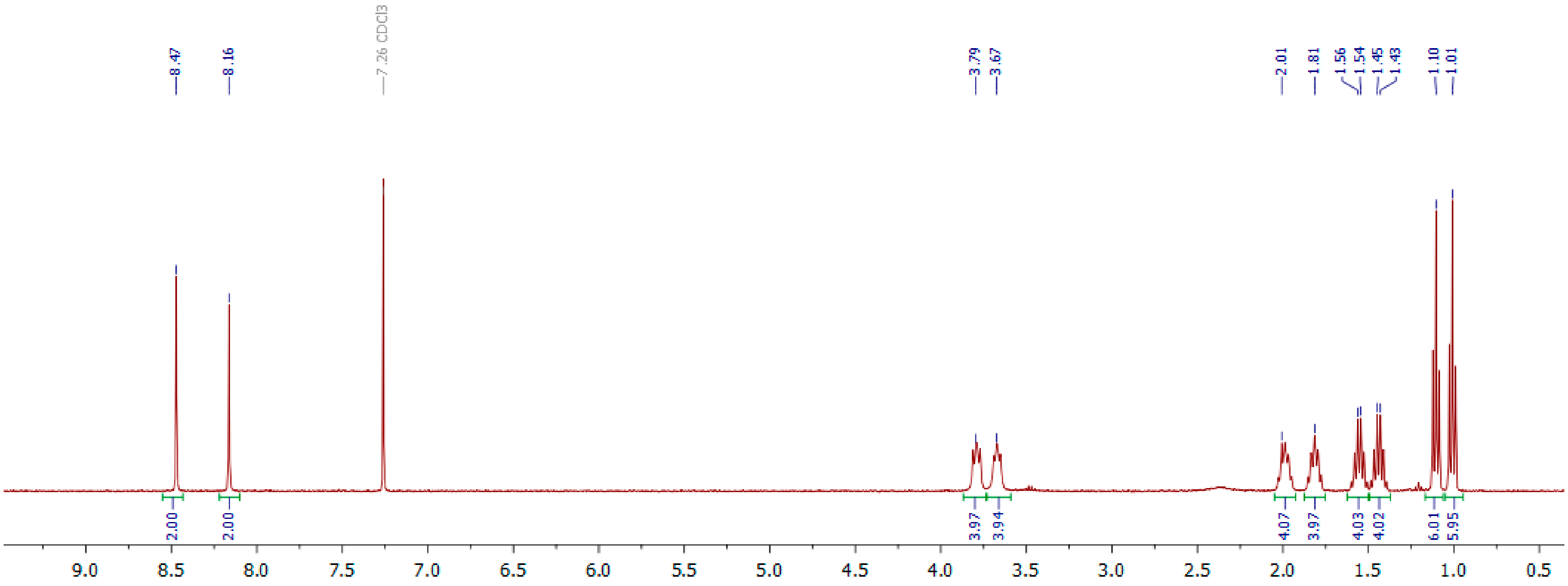

Figure S1. ^{1}H NMR spectra of **L3Lu(NO$_3$)$_3$** in $CDCl_3$ at 25°C

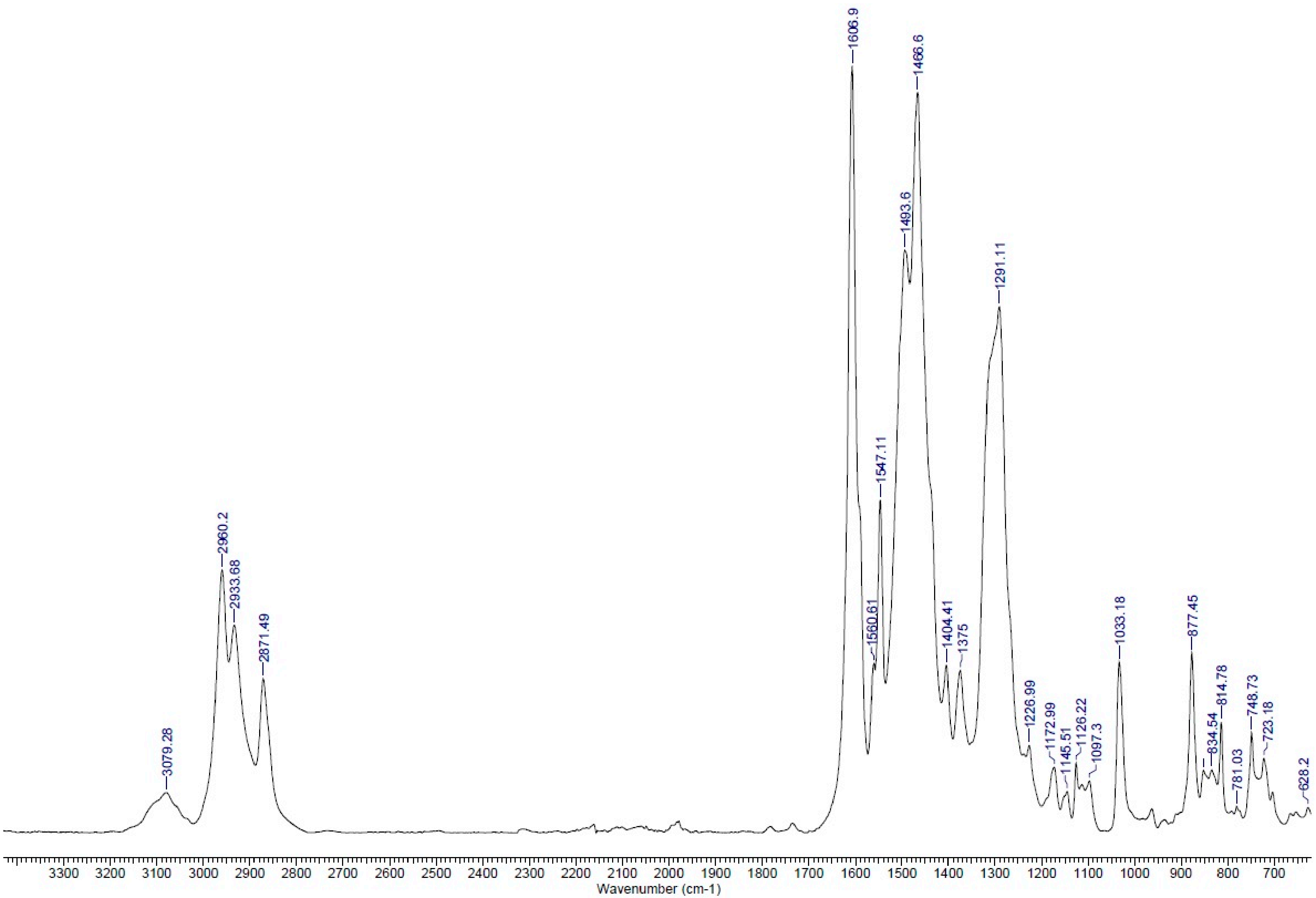

Figure S2. Solid-state IR spectra of **L3Lu(NO_3)$_3$**

2. X-ray diffraction data

Table S1 Crystal data and structure refinement parameters for **$L3\bullet Lu(NO_3)_2\bullet H_2O]^+\bullet NO_3^-$**

	**$L3\bullet Lu(NO_3)_2\bullet H_2O]^+\bullet NO_3^-$**
Empirical formula	$C_{30}H_{42}Cl_2LuN_7O_{12}$
Formula weight	938.57
T, K	295
Diffractometer	Stoe STADI VARI
Radiation	CuKα, 1.54186 Å
Crystal system	Triclinic
Space group	P-1
Z	2
a, Å	9.3262(3)
b, Å	14.0448(5)
c, Å	15.8316(5)
α/°	66.734(3)
β/°	87.821(3)
γ/°	85.175(3)
V, $Å^3$	1898.29(12)
D_{calc} (g cm^{-1})	1.642
Linear absorption, μ (mm^{-1})	6.855
F(000)	944
$2\theta_{max}$, °	70.890
Reflections measured	19264
Independent reflections	6642
Observed reflections [$I > 2\sigma(I)$]	5591
Parameters	480
R1 ($I > 2\sigma(I)$)	0.0322
wR2 (all reflections)	0.0758
GOF	0.947
$\Delta\rho_{max}$/ $\Delta\rho_{min}$ (e $Å^{-3}$)	0.664 / -0.731

3. Theoretical computations

All molecular geometries have been fully optimized (tolerance on gradient: 10^{-7} au) at PBE/L1 level.

All stationary points on the potential energy surface (PES) were checked by vibrational analysis and none of them had imaginary frequencies. We used eq. (1) as a model reaction of complex formation.

$$Met(NO_3)_3 + \mathbf{L} = \mathbf{L}{*}Met(NO_3)_3 \quad (1)$$

The energy of complex formation in gas phase was calculated according to eq. (2)

$$\Delta E = E_{LMet(NO3)3} - E_{Met(NO3)3} - E_L \quad (2)$$

where $E_{LMet(NO3)3}$, $E_{Met(NO3)3}$, E_L are energies for optimized geometrical structures of $[Met(NO_3)_3]L$, $Met(NO_3)_3$, and L respectively.

Table S2. Energies of consecutive attachment of NO_3^- anions to cations $[\mathbf{L2}Ln]^{3+}$ (gas phase approximation) in kcal/mol.

$[\mathbf{L2}Ln]^{3+} + NO_3^- = [\mathbf{L2}Ln(NO_3)]^{2+} + NO_3^- = [\mathbf{L2}Ln(NO_3)_2]^{+} + NO_3^- = \mathbf{L2}Ln(NO_3)_3$

	NO_3^-		$2NO_3^-$		$3NO_3^-$	
Ln	ΔE	ΔG	ΔE	ΔG	ΔE	ΔG
La	-288.9	-276.0	-197.5	-186.4	-117.0	-104.0
Ce	-286.9	-274.3	-202.6	-191.1	-118.5	-104.2
Pr	-286.0	-273.4	-199.5	-187.7	-115.5	-102.8
Nd	-287.5	-273.5	-199.1	-188.0	-114.4	-101.8
Pm	-287.8	-271.2	-200.1	-187.6	-113.3	-100.1
Sm	-283.6	-266.6	-200.5	-188.1	-111.5	-99.0
Eu	-279.4	-260.8	-200.6	-187.9	-111.4	-98.1
Gd	-295.5	-281.8	-201.5	-189.6	-111.1	-97.2
Tb	-295.5	-280.2	-201.9	-189.8	-110.6	-97.0
Dy	-293.1	-276.7	-202.5	-190.0	-109.3	-95.4
Ho	-295.3	-279.0	-201.7	-189.5	109.0	94.9
Er	-295.0	-275.7	-202.8	-190.9	-107.5	-92.7
Tm	-291.0	-273.1	-202.9	-189.9	-106.3	-92.7
Yb	-287.4	-267.8	-202.8	-189.7	-105.9	-92.2
Lu	-303.8	-289.9	-205.0	-192.1	-102.9	-89.2

Table S3 ΔE and ΔG for $[\mathbf{L3}Ln(NO_3)]^{2+}$ (kcal·mol^{-1}) and subsequent addition of NO_3^- (eq 1).

	$[LLn(NO_3)]^{2+}$		$[LLn(NO_3)_2]^{+}$		$LLn(NO_3)_3$	
Ln	ΔE	ΔG	ΔE	ΔG	ΔE	ΔG
La	-244.2	-226.5	-129.9	-112.8	-54.1	-36.2
Ce	-531.8	-229.4	-132.9	-116.0	-59.9	-38.6
Pr	-248.2	-229.9	-130.4;	-112.9	-52.3	-33.9
Nd	-250.8	-233.0	-130.3	-112.1	-49.5	-30.3
Pm	-252.7	-232.6	-131.8	-113.6	-49.5	-29.8
Sm	-251.4	-232.7	-130.9	-112.3	-48.0	-29.6
Eu	-243.9	-225.2	-128.9	-110.4	-47.5	-28.3
Gd	-266.7	-248.1	-141.0	-123.0	-50.5	-30.1
Tb	-268.6	-249.9	-141.7	-123.3	-50.0	-30.0
Dy	-269.3	-250.6	-142.2	-123.5	-48.4	-28.3
Ho	-272.9	-254.1	-143.0	-125.8	-47.2	-27.3
Er	-274.9	-255.6	-144.5	-125.1	-46.9	-26.2
Tm	-274.0	-254.5	-143.3	-123.9	-44.8	-24.9
Yb	-268.5	-249.1	-141.7	-122.4	-44.4	-24.7
Lu	-292.0	-273.3	-155.3	-135.8	-46.0	-25.1

Table S4. Energies of consecutive attachment of NO_3^- anions to cations $[(\mathbf{L4})Ln]^{3+}$ (gas phase approximation) in kcal/mol.

	NO_3^-		$2NO_3^-$		$3NO_3^-$	
Ln	ΔE	ΔG	ΔE	ΔG	ΔE	ΔG
La	-292.8	-280.4	-198.8	-188.1	-117.1	-104.8
Ce	-291.7	-279.5	-203.3	-192.0	-119.7	-105.6
Pr	-290.9	-277.9	-201.3	-289.8	-115.5	-102.9
Nd	-292.4	-279.9	-200.6	-188.2	-114.3	-101.7
Pm	-293.9	-280.8	-201.6	-189.5	-113.1	-99.7
Sm	-291.5	-278.1	-202.3	-202.3	-111.1	-99.2
Eu	-288.3	-274.8	-202.8	-202.8	-110.8	-97.7
Gd	-300.5	-287.3	-203.1	-203.1	-110.8	-97.3
Tb	-301.0	-287.6	-203.6	-203.6	-110.3	-96.8
Dy	-299.5	-285.8	-204.2	-204.2	-109.0	-95.6
Ho	-301.8	-288.3	-203.5	-203.5	-108.6	-94.7
Er	-302.4	-288.2	-204.6	-204.6	-107.6	-94.4
Tm	-299.5	-285.4	-204.8	-204.8	-105.8	-93.0
Yb	-297.1	-282.7	-205.1	-205.1	-106.0	-92.8
Lu	-308.7	-295.4	-206.9	-206.9	-102.9	-89.6

Table S5. Energies of successive addition of nitrate anions ΔE and (ΔG) in kcal/mol to the cation $[Ln]^{3+}$ calculated according to $[\mathbf{L}Ln]^{3+}$ + NO_3^- = $[\mathbf{L}Ln(NO_3)]^{2+}$ + NO_3^- = $[\mathbf{L}Ln(NO_3)_2]^+$ + NO_3^- = $\mathbf{L}Ln(NO_3)_3$ reaction

Ln	NO_3^-	2 NO_3^-	3 NO_3^-
La	-305.8 (-292.9)	-211.6 (-199.1)	-127.1 (-115.0)
Nd	-305.9) (-288.8)	-213.8 (-202.5)	-126.4 (-112.3)
Eu	-293.9 (-274.7)	-215.0 (-202.8)	-124.5 (-110.3)
Dy	-311.8 (-293.3)	-218.8 (-206.4)	-124.3 (-109.9)
Yb	-303.7 (-284.5)	-219.4 (-204.6)	-123.0 (-110.5)
Lu	-327.6 (313.7)	-223.2 (-210.9)	-122.7 (-107.8)

Table S6 The formation energies calculated with PBE and B3LYP

	NO^{3-}		$2NO^{3-}$		$3NO^{3-}$	
Ln	ΔE (pbe)	ΔE (b3lyp)	ΔE (pbe)	ΔE (b3lyp)	ΔE	ΔE b3lyp
La	-244.2	-243.9	-129.9	-133.1	-54.1	-54.9
Ce	-246.7	-246.5	-132.9	-132.4	-59.9	-52.6
Pr	-248.2	-249.8	-130.4	-134.0	-52.3	-51.4
Nd	-250.8	-254.0	-130.3	-135.6	-49.5	-51.5
Pm	-252.7	-257.1	-131.8	-137.7	-49.5	-51.4
Sm	-251.4	-258.7	-130.9	-138.4	-48.0	-50.5
Eu	-243.9	-252.2	-128.9	-137.8	-47.5	-50.0
Gd	-266.7	-269.4	-141.0	-145.8	-50.5	-51.2
Tb	-268.6	-272.8	-141.7	-147.1	-50.0	-50.1
Dy	-269.3	-276.4	-142.2	-148.5	-48.4	-48.9
Ho	-272.9	-280.2	-143.0	-150.2	-47.2	-48.4
Er	-274.9	-283.1	-144.5	-151.7	-46.9	-47.5
Tm	-274.0	-285.5	-143.3	-152.6	-44.8	-45.9
Yb	-268.5	-285.0	-141.7	-153.5	-44.4	-45.2
Lu	-292.0	-293.5	-155.3	-157.9	-46.0	-45.1

To compare two types of complexes (water molecules in outer coordination sphere – I, and water molecule in inner coordination sphere – II, Figure S3) we optimized molecular geometries of complexes for ligands **L2-L4** with lanthanides (from La to Lu) at PBE/L1 level of theory (tolerance on gradient: 10^{-7} au) using a PRIODA-19 program developed by Laikov [Laikov, D.N. Atomic basis functions for molecular electronic structure calculations. *Theor. Chem. Acc.* **2019**, *138*, 40. https://doi.org/10.1007/s00214-019-2432-3].

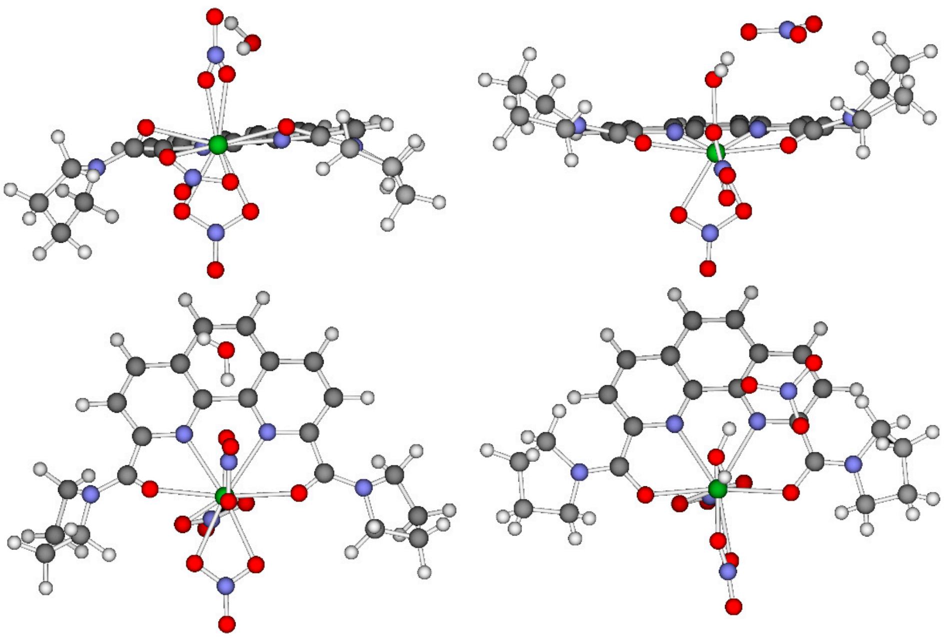

I II

Figure S3. Structures of two types of complexes - **I** (H_2O in outer coordination sphere) and **II** (H_2O in inner coordination sphere)

xyz data

Ligand L2

E= -2458.982234, E0 = -2458.342421, G= 351.5475 at T=298.150 K

Cl -4.53443527 4.40782070 -3.30053735

Cl -5.20993662 -3.14527750 -3.72041559

N -1.58535874 1.57410634 -1.30706620

N 0.96606773 2.99341249 -0.67157280

N -1.96913099 -1.11286223 -1.25714874

N -0.84170198 -3.00824738 0.75871980

O -0.56885952 4.25740814 0.46990582

O -0.44765243 -4.12524939 -1.20433164

C -1.43717253 2.89075065 -1.25154722

C -2.35162997 3.80709767 -1.81764197

H -2.20297217 4.87944508 -1.68790185

C -3.41632152 3.30162501 -2.53062534

C -3.61005259 1.89812601 -2.64236975

C -4.67225695 1.29334116 -3.37782502

H	-5.38231325	1.94188643	-3.89373946
C	-4.79865408	-0.06619585	-3.44670129
H	-5.60693121	-0.51818967	-4.02406454
C	-3.88919783	-0.92622989	-2.76246381
C	-3.96260095	-2.34531999	-2.78722715
C	-3.05696177	-3.10590053	-2.08098865
H	-3.07142639	-4.19566965	-2.11343741
C	-2.09451294	-2.43249488	-1.29575145
C	-2.82537818	-0.36878082	-1.98864388
C	-2.65613294	1.07947350	-1.96334422
C	-0.29725426	3.44706345	-0.41638678
C	-1.06037009	-3.27035451	-0.56429797
C	-1.70463181	-2.15802717	1.58808339
C	0.23426451	-3.76782417	1.41046417
C	1.34055781	2.20614219	-1.85286653
C	2.03013325	3.47519851	0.22013471
C	1.62998557	-3.16302752	1.20893681
H	-0.00834809	-3.82895160	2.48452663
H	0.22206604	-4.78754520	0.98940086
C	-1.12626934	-0.78447652	1.94855750
H	-2.66160440	-2.02196336	1.06232202
H	-1.92896783	-2.72383618	2.51184750
C	1.62091506	0.72113293	-1.59324086
H	0.53570330	2.29863524	-2.59744716
H	2.23542213	2.68474150	-2.29323483
C	2.16048789	2.67094588	1.52033222
H	2.97518253	3.44673061	-0.34755954
H	1.80247271	4.52535582	0.47030666
C	3.26494503	3.22325730	2.42810750
H	1.19304430	2.70602965	2.04886317
H	2.36468482	1.61193776	1.28418291

C	2.02803922	-0.00002292	-2.88443780
H	2.42687702	0.61724430	-0.84474093
H	0.70812231	0.26499325	-1.17504060
C	2.72007632	-3.99781919	1.88993597
H	1.82959163	-3.10392523	0.12579536
H	1.64876735	-2.13102961	1.60090816
C	-2.09456635	0.00827873	2.83558178
H	-0.16361018	-0.90987897	2.47600698
H	-0.93166536	-0.23468895	1.01260710
C	4.12180185	-3.41199923	1.68955493
H	2.50317740	-4.07657433	2.97146010
H	2.68930459	-5.02819872	1.49131024
C	-1.55695331	1.38939714	3.22525668
H	-3.05628777	0.12640262	2.30291581
H	-2.31657791	-0.57392162	3.74963379
C	2.32253218	-1.48881066	-2.67208004
H	1.21854603	0.11076291	-3.62945199
H	2.91731095	0.49516228	-3.31770873
C	3.40155411	2.43509221	3.73514533
H	4.22890186	3.21095324	1.88628721
H	3.05033231	4.28323317	2.65592408
H	4.19660521	2.85094142	4.37433958
H	2.46034217	2.45833111	4.30896235
H	3.64668345	1.37837982	3.53653741
H	2.63812494	-1.97017121	-3.61147046
H	3.13130260	-1.62948549	-1.93511772
H	1.43302679	-2.02478313	-2.30385494
H	4.88971519	-4.02866936	2.18315411
H	4.37408400	-3.35267925	0.61787719
H	4.18844986	-2.39311194	2.10620070
H	-2.26293159	1.92003047	3.88388872

H -0.59786135 1.30170643 3.76326346

H -1.38971162 2.01961970 2.33704352

L2*La($(NO_3)_3$)(H_2O)

(H_2O in outer coordination sphere)

E=-11871.787674, E0 = -11871.076771, G= 378.7348 at T=298.150 K

Cl -5.52118683 3.59837317 -0.2400401504

Cl -4.38623381 -3.19738984 -3.48092198

N -1.69283342 1.23818433 0.26213586

N -0.28642842 4.05627060 1.93433750

N -1.30030036 -1.16488612 -0.86667764

N 1.05000436 -3.66783786 -1.70320821

O -0.14073125 1.83043957 2.34949422

O 0.47518417 -2.79157329 0.31123731

C -1.84679866 2.42922735 0.83684456

C -3.02722931 3.18242908 0.70002151

H -3.16264749 4.12369585 1.23089075

C -4.04665565 2.68231130 -0.08873926

C -3.90203261 1.44048798 -0.75178862

C -4.89546633 0.84421414 -1.58462596

H -5.83060169 1.38308001 -1.74390006

C -4.69530916 -0.37813923 -2.16199136

H -5.46665573 -0.82594907 -2.79024363

C -3.48345780 -1.10290587 -1.95097494

C -3.20584965 -2.38504481 -2.48773479

C -2.00227666 -3.01612091 -2.22525167

H -1.80567050 -4.01272917 -2.62082672

C -1.06370854 -2.35790467 -1.40922642

C -2.46233416 -0.54132229 -1.13065004

C -2.67516470 0.75805753 -0.51914692

C -0.69064093 2.78104997 1.75505984

C 0.22485520 -2.97264719 -0.89792532

C	-0.65421605	5.19099426	1.07624745
C	-1.48526990	6.26927900	1.78149235
H	-1.18050897	4.80873203	0.18921770
C	0.83327162	4.27372122	2.87819672
H	0.69303340	3.57358527	3.71508789
H	0.73461306	5.30092669	3.26618242
C	2.29183006	-4.18392086	-1.08006573
H	2.69113755	-3.39154005	-0.42373064
H	3.01042366	-4.34939528	-1.89830792
C	0.97217679	-3.67206335	-3.16986108
H	0.17602775	-2.98035526	-3.48210168
C	0.75203305	-5.06074381	-3.77712798
La	0.52532554	-0.33016407	1.12207365
N	-1.16401744	-1.32574427	3.41740680
O	0.10716507	-1.21217322	3.51882935
O	-1.65627444	-1.09990525	2.21650243
O	-1.89204764	-1.61406648	4.34921169
N	3.32002831	-0.50873744	2.16504765
O	2.61901855	0.58735543	2.21699691
O	2.70825720	-1.53133619	1.65417182
O	4.46740055	-0.56312203	2.56487679
N	1.66057611	0.86460227	-1.41033554
O	2.07067084	1.33865988	-2.45466447
O	1.67609537	-0.41467583	-1.17989731
O	1.17555130	1.59906828	-0.45959499
H	0.28735605	5.63254833	0.70394754
C	2.21249080	4.05425978	2.24726582
C	-1.83816373	7.42449617	0.83680278
H	-2.41054344	5.82494307	2.19241667
H	-0.92633003	6.66178751	2.64913559
C	-2.65392041	8.52238655	1.52635920

H	-0.90643018	7.85543394	0.42758515
H	-2.40174556	7.03043604	-0.02873448
H	-2.89497113	9.33754063	0.82639349
H	-3.60367799	8.12432098	1.92015207
H	-2.09714532	8.95809841	2.37220860
C	3.33389282	4.22418261	3.27883816
H	2.25808954	3.03486896	1.83176661
H	2.36417317	4.75796747	1.40798879
C	4.71430779	3.92046738	2.68848133
H	3.31338668	5.25012112	3.69341159
H	3.14491701	3.53657341	4.12333822
H	5.50749493	4.06097078	3.43984270
H	4.75921345	2.87649322	2.33798957
H	4.93724346	4.57912302	1.83193922
C	2.08455968	-5.46927023	-0.27366072
H	1.91634023	-3.23864079	-3.54672623
C	0.69763070	-5.01149321	-5.30874491
H	1.56493974	-5.73708725	-3.45814157
H	-0.18445583	-5.49581289	-3.38114619
C	0.48896462	-6.39290428	-5.93681860
H	-0.11549123	-4.33247280	-5.62473249
H	1.63507223	-4.56775284	-5.69023228
H	0.45274404	-6.32897329	-7.03552198
H	1.30822134	-7.07910728	-5.66619682
H	-0.45543024	-6.84723186	-5.59425783
C	3.38866401	-5.92495823	0.39186180
H	1.32561123	-5.27840471	0.50372154
H	1.69439518	-6.26850605	-0.93022513
C	3.21227241	-7.20634842	1.21206057
H	4.16461992	-6.08067942	-0.38090113
H	3.75600076	-5.11373758	1.04577553

H 4.16115999 -7.51276445 1.67991567

H 2.47343063 -7.05905008 2.01712108

H 2.86125565 -8.04042435 0.58118731

O -4.20741940 0.12163923 2.19404197

H -3.38024902 -0.40931782 2.27442670

H -4.55460644 0.10392769 3.10203767

(NO_3^- in outer coordination sphere)

E= -11871.774736, E0 = -11871.063432, G= 380.8389 at T=298.150 K

La -0.90557778 -0.53899211 0.01477725

Cl 3.70405483 2.05276918 4.92820263

Cl 2.53523493 5.61195564 -1.65928566

O -1.17187881 0.48950687 -2.19344378

O -0.91260630 -1.28505540 2.49133277

O -3.38669705 -0.90585071 0.22898123

O -4.38261271 -2.87217236 0.15231645

O -2.21448231 -2.71067953 -0.21090414

O 0.87810749 -2.36670685 0.41152176

O 0.65099835 -1.67686772 -1.66008866

O 2.09690571 -3.27093482 -1.18425202

O -1.98220778 1.56760597 0.83224189

H -1.80795026 2.57499647 0.53841579

H -2.95173597 1.46102524 0.87919813

N 1.02540529 0.38882813 1.69111860

N 0.60068011 -2.56193995 3.60543251

N 0.69848949 1.59142613 -0.72227836

N -0.73378199 1.64996779 -4.06485653

N -3.37630558 -2.19962430 0.05909375

N 1.24737215 -2.47609258 -0.82957286

C 1.16364586 -0.24081823 2.85840631

C 1.98785567 0.25250623 3.88407755

H 2.04941487 -0.24853213 4.85002232

C	2.69512248	1.42014396	3.65620470
C	2.59299231	2.10291576	2.41829491
C	3.26793718	3.32259965	2.11498189
H	3.93592787	3.75023103	2.86367893
C	3.06292129	3.95565796	0.92259926
H	3.56061077	4.90174723	0.70481586
C	2.18162298	3.41509056	-0.06068257
C	1.52350950	2.17533803	0.17089124
C	1.71975112	1.51751041	1.45313787
C	1.85734117	4.05787563	-1.27775002
C	0.97842526	3.47742534	-2.17200947
H	0.67411959	4.05204344	-3.03900218
C	0.43212906	2.21021557	-1.88222003
C	-0.54912430	1.42482102	-2.74836969
C	-0.10703258	2.68534255	-4.89651203
H	-0.01798707	2.26061988	-5.91051769
H	0.92609680	2.85101795	-4.55589676
C	-0.91849494	3.99041128	-4.92966127
H	-1.87649906	3.79989767	-5.44569445
H	-1.19468141	4.28542519	-3.89980054
C	-0.17012870	5.12444782	-5.63890934
H	0.07592789	4.82357073	-6.67413950
H	0.79866999	5.30024147	-5.13283157
C	-0.97818482	6.42646980	-5.64852190
H	-1.93053472	6.29577446	-6.18828726
H	-0.42038512	7.23951578	-6.13934374
H	-1.21554923	6.74201107	-4.61987019
C	-1.73700118	0.80172646	-4.74390984
H	-2.13875961	1.39224577	-5.58307314
H	-2.55601859	0.61227828	-4.03478384
C	-1.14358759	-0.52018702	-5.24356556

H	-0.70573640	-1.05975318	-4.38607597
H	-0.31868348	-0.31203210	-5.95044231
C	-2.19966102	-1.39700365	-5.92581749
H	-3.01440597	-1.60255909	-5.20764256
H	-2.65711498	-0.84163755	-6.76603794
C	-1.62187994	-2.72137499	-6.43423033
H	-0.81905240	-2.54904509	-7.17063332
H	-2.39810324	-3.33565354	-6.91742420
H	-1.19506848	-3.30809975	-5.60402346
C	0.22379448	-1.42423105	2.99256229
C	1.99055982	-2.91476679	3.92825103
H	2.65329289	-2.12481117	3.54632950
H	2.23609781	-3.82237768	3.34775209
C	2.23794508	-3.15871620	5.41954422
H	1.97193849	-2.25219655	5.99457264
H	1.56752741	-3.95927048	5.77995872
C	3.69496369	-3.54562330	5.70024443
H	4.36340618	-2.74355531	5.33703423
H	3.94938993	-4.44738340	5.11435032
C	3.96088219	-3.80488563	7.18626595
H	3.74181175	-2.91002011	7.79193497
H	5.01301861	-4.07941151	7.35970259
H	3.33156991	-4.62741709	7.56401110
C	-0.39793333	-3.65673971	3.62120819
H	0.16079836	-4.59200382	3.78379297
H	-0.85151875	-3.69982290	2.61814022
C	-1.47589254	-3.48551536	4.69683218
H	-1.96606469	-2.50736117	4.55533934
H	-0.99602926	-3.47374272	5.69140911
C	-2.53629351	-4.59858704	4.64217663
H	-2.03860784	-5.58470631	4.70799732

H	-3.16465712	-4.51286507	5.54579878
C	-3.43373227	-4.54869413	3.39970875
H	-3.94013882	-3.57267952	3.31637073
H	-4.21235418	-5.32648420	3.44798470
H	-2.87297702	-4.70698833	2.46468496
O	-1.42130482	3.96580911	0.25120541
O	-1.18899536	5.35874510	-1.42993319
O	-2.19500279	3.43431735	-1.75108588
N	-1.61725271	4.26839685	-1.00152552

L2*La$(NO_3)_2^+$

E= -11514.812210, E0 = -11514.139526, G= 361.0390 at T=298.150 K

Cl	-5.47865820	3.60568333	-0.25474846
Cl	-4.19033432	-3.14690876	-3.49129748
N	-1.63633609	1.30424452	0.32509458
N	-0.29275426	4.12395287	2.05832100
N	-1.17663717	-1.09121096	-0.82738602
N	1.16823232	-3.64805102	-1.68192685
O	-0.06287735	1.88896334	2.35606217
O	0.75646001	-2.54885483	0.26143229
C	-1.81836879	2.49624062	0.90478343
C	-3.00665498	3.22823739	0.74305034
H	-3.17423081	4.16334438	1.27494264
C	-4.00386810	2.71709967	-0.07346840
C	-3.82234430	1.47856045	-0.74217075
C	-4.78719091	0.87996185	-1.60755026
H	-5.72219658	1.41061783	-1.79281914
C	-4.55444527	-0.33410576	-2.19035816
H	-5.30189276	-0.78344876	-2.84584117
C	-3.33874130	-1.04449046	-1.95411527
C	-3.04010487	-2.32375455	-2.49256015
C	-1.82753122	-2.93545413	-2.21669078

H	-1.62237787	-3.93050098	-2.60967469
C	-0.90356147	-2.27197194	-1.39070511
C	-2.34585881	-0.47947487	-1.10620761
C	-2.59306645	0.80880469	-0.48642531
C	-0.66928947	2.85898304	1.82704532
C	0.41242865	-2.85270357	-0.91127950
C	-0.77887309	5.31948996	1.34436953
C	-1.67892241	6.22712278	2.19027781
H	-1.28582919	5.00371742	0.42070532
C	0.85636950	4.33418608	2.97461057
H	0.76597464	3.60908747	3.79655218
H	0.74551135	5.34646988	3.39361429
C	2.38811231	-4.22813749	-1.06446075
H	2.89105797	-3.42985678	-0.49496266
H	3.04853797	-4.52326918	-1.89410150
C	1.01258528	-3.82245994	-3.13901019
H	0.26404494	-3.10443330	-3.50345063
C	0.66288638	-5.24977636	-3.56888604
La	0.27277678	-0.33412692	1.30328000
N	-1.36689448	-1.73374999	3.31182599
O	-0.06727159	-1.59461725	3.40301633
O	-1.90191317	-1.19813228	2.24655128
O	-2.01591158	-2.31156898	4.14701557
N	2.75212026	0.62889576	-0.00764709
O	3.76656461	1.03142190	-0.52571267
O	2.66902876	0.38702667	1.27515852
O	1.65662229	0.40799236	-0.68257791
H	0.11802733	5.87839699	1.02598286
C	2.21081018	4.18182421	2.27294159
C	-2.14782333	7.45523357	1.39909041
H	-2.55314374	5.65975237	2.56044173

H	-1.13243401	6.55605316	3.09133887
C	-3.03729081	8.38350105	2.23153138
H	-1.26451099	8.01209736	1.03802514
H	-2.69471049	7.12488937	0.49670231
H	-3.36035967	9.25320053	1.63962054
H	-3.94167876	7.86115837	2.58404374
H	-2.49991035	8.76028347	3.11670709
C	3.37933803	4.44746971	3.23040485
H	2.29618526	3.15699601	1.87243426
H	2.26903510	4.87577295	1.41415286
C	4.73769283	4.27842236	2.54324389
H	3.29264450	5.46737385	3.64819455
H	3.30940127	3.75061083	4.08546019
H	5.56161547	4.46597052	3.24874449
H	4.85588169	3.25569725	2.14870071
H	4.84878445	4.98045015	1.70041502
C	2.09982824	-5.42500591	-0.15192775
H	1.97227001	-3.51857114	-3.59403419
C	0.51871127	-5.36062527	-5.09242249
H	1.44771004	-5.94438171	-3.22190642
H	-0.27253020	-5.57511568	-3.07633638
C	0.18410525	-6.78434896	-5.54714680
H	-0.26669189	-4.66398764	-5.43912697
H	1.45837581	-5.02918768	-5.56991196
H	0.08516907	-6.83458996	-6.64205694
H	0.97371167	-7.49258471	-5.24838543
H	-0.76464999	-7.13224983	-5.10688019
C	3.39034867	-5.97742701	0.46695367
H	1.41114259	-5.11030006	0.65031314
H	1.59170377	-6.21775484	-0.72913802
C	3.12986588	-7.17623472	1.38384211

H 4.09059811 -6.26853991 -0.33748502

H 3.88923740 -5.17467451 1.03986764

H 4.06967592 -7.55356932 1.81521404

H 2.46406317 -6.90025139 2.21797585

H 2.65542483 -8.00479889 0.83274043

L2*Ce(NO_3)$_3$(H_2O)

(H_2O in outer coordination sphere)

E= -12239.208698 , E0 = -12238.498179, G= 379.3895 at T=298.150 K

Cl -5.38830900 3.68461299 -0.47863379

Cl -4.13156796 -3.01257038 -3.89721632

N -1.57952487 1.30878425 0.06828741

N -0.27020249 4.05604219 1.97267938

N -1.13508022 -1.05593002 -1.10732663

N 1.11563969 -3.76320195 -1.77267396

O 0.07227688 1.82056355 2.01922321

O 0.62034023 -2.58067083 0.09793225

C -1.74547279 2.48372889 0.68201721

C -2.92976570 3.23455930 0.53342962

H -3.10230398 4.14385414 1.10499108

C -3.91511583 2.76618004 -0.31052810

C -3.75085568 1.54988134 -1.02195477

C -4.70132160 0.99020070 -1.91277564

H -5.62866163 1.53353608 -2.09841919

C -4.47024155 -0.22129570 -2.52158165

H -5.21329594 -0.64800137 -3.19697380

C -3.27779484 -0.95516610 -2.28157377

C -2.98360920 -2.23294783 -2.83812070

C -1.80363274 -2.88301563 -2.52851009

H -1.61830997 -3.88204694 -2.92172813

C -0.88649243 -2.26191401 -1.66344821

C -2.28690958 -0.42444131 -1.40324080

C	-2.52972674	0.85381311	-0.76309192
C	-0.59551597	2.79903054	1.59927571
C	0.33890465	-2.89565372	-1.08984375
C	-0.75474203	5.29259443	1.34448814
C	-1.68810534	6.12443495	2.23271036
H	-1.23581636	5.04310274	0.38741261
C	0.86624587	4.20361662	2.90890908
H	0.81638336	3.36672044	3.62088656
H	0.70483810	5.14177370	3.46541452
C	2.27568698	-4.32761335	-1.04523289
H	2.75313783	-3.50746512	-0.48240319
H	2.98480606	-4.68198490	-1.81010330
C	1.07785046	-3.94647765	-3.22889161
H	0.39593640	-3.19815731	-3.65833211
C	0.68880904	-5.36011791	-3.67377782
Ce	0.59128433	-0.20915997	0.71961832
N	-0.87836933	-1.19097483	3.08725810
O	0.39894247	-1.03545868	3.06846309
O	-1.47298777	-0.97561318	1.94056702
O	-1.49967659	-1.50795531	4.08142567
N	3.41051388	-0.25812978	1.57015789
O	2.71922159	0.83987337	1.43636930
O	2.75258040	-1.33871877	1.30962574
O	4.57577467	-0.25408569	1.90916455
N	1.75157523	0.89068395	-1.76196909
O	2.24890351	1.34462714	-2.77142525
O	1.87559426	-0.35886976	-1.42217398
O	1.05128813	1.60928857	-0.94158620
H	0.13794683	5.89086676	1.08934331
C	2.22999644	4.20882797	2.20927238
C	-2.13253021	7.41640711	1.53648126

H	-2.57219505	5.52613258	2.51962209
H	-1.17312479	6.37434483	3.17716765
C	-3.06544590	8.26566696	2.40516806
H	-1.23794591	8.00583172	1.26465333
H	-2.63721180	7.16652060	0.58515871
H	-3.34668374	9.19726181	1.88943577
H	-3.99460602	7.72073364	2.64004898
H	-2.58351040	8.54017925	3.35778785
C	3.37979364	4.31600332	3.21780443
H	2.33991051	3.27367353	1.63666928
H	2.28475451	5.04724312	1.49020946
C	4.75234461	4.25029278	2.54066873
H	3.28667808	5.25376844	3.79777551
H	3.29406118	3.48610330	3.94288301
H	5.56474781	4.33365583	3.27987647
H	4.87396002	3.29282379	2.00842285
H	4.87579250	5.06555557	1.80778885
C	1.90546632	-5.46684074	-0.09047297
H	2.08440590	-3.69871187	-3.61297917
C	0.66890037	-5.49170017	-5.20138121
H	1.40112185	-6.08983231	-3.24902892
H	-0.30286545	-5.62272692	-3.26084018
C	0.29774034	-6.90267420	-5.66791058
H	-0.04615611	-4.76106215	-5.62197304
H	1.66160846	-5.21693707	-5.60201645
H	0.28972638	-6.96893454	-6.76709366
H	1.01782215	-7.64743662	-5.29076815
H	-0.70297348	-7.19081450	-5.30577326
C	3.13811040	-5.99795628	0.65035605
H	1.17209089	-5.08928871	0.64182758
H	1.42082274	-6.28564072	-0.65313965

C 2.79705191 -7.13333464 1.62019706

H 3.88787556 -6.34892654 -0.08342401

H 3.60972691 -5.16584587 1.20390558

H 3.69746971 -7.49629974 2.14053392

H 2.07976890 -6.79458904 2.38598585

H 2.34472656 -7.98853827 1.09034729

O -3.93708754 0.44724032 2.29033947

H -3.16999841 -0.16336331 2.21282411

H -4.20521307 0.32845590 3.21747613

(NO_3^- in outer coordination sphere)

E= -12239.196494, E0 = -12238.487842, G= 378.4462 at T=298.150 K

Ce -0.62523645 -0.55376226 -0.03314401

Cl 3.94993663 1.87506688 4.83344412

Cl 2.63411188 5.58727551 -1.67951775

O -0.97555101 0.38601041 -2.19498944

O -0.70983666 -1.39045393 2.27455592

O -3.04005909 -1.12597537 0.03526488

O -3.86225224 -3.09091377 -0.53788334

O -1.70407665 -2.68001270 -0.72633463

O 1.03768349 -2.44126940 0.32599366

O 1.14448130 -1.38802719 -1.58584023

O 2.48443675 -3.09583497 -1.19996202

O -1.76827526 1.26810539 0.72646701

H -1.67760623 2.57110667 0.45238915

H -2.70377040 1.07799911 0.93689758

N 1.20402145 0.29316759 1.60012889

N 0.64825559 -2.61341190 3.62203622

N 0.82568026 1.55322123 -0.75317156

N -0.78235346 1.68279099 -4.01761866

N -2.91909766 -2.33943963 -0.41806275

N 1.59989655 -2.34786844 -0.83677566

C	1.34393084	-0.36729190	2.76982832
C	2.19142437	0.11281154	3.78476858
H	2.25534344	-0.38899770	4.74975348
C	2.91225028	1.27086425	3.56687570
C	2.79462934	1.98932195	2.33984709
C	3.46355081	3.20375872	2.04445195
H	4.14754057	3.61530066	2.78796268
C	3.23843241	3.87232447	0.86122900
H	3.73825669	4.82035589	0.65851623
C	2.33809423	3.36101151	-0.10486032
C	1.67392433	2.11658406	0.12416274
C	1.89499485	1.42548251	1.38471854
C	1.97562373	4.01719332	-1.31106782
C	1.08031058	3.44935060	-2.19313645
H	0.77434009	4.02975512	-3.05649900
C	0.53695750	2.17568421	-1.91158986
C	-0.44424382	1.38984990	-2.74436808
C	-0.22475494	2.73612714	-4.87437344
H	-0.21457639	2.32986617	-5.90020227
H	0.83073461	2.89975405	-4.61191273
C	-1.03739476	4.03965378	-4.83821583
H	-2.05049634	3.83717465	-5.22903585
H	-1.18792999	4.37001610	-3.79420996
C	-0.37983760	5.15113735	-5.66371632
H	-0.23721479	4.80582714	-6.70440054
H	0.63264519	5.35553503	-5.26644707
C	-1.20135689	6.44450474	-5.65311289
H	-2.20091414	6.28051138	-6.08817673
H	-0.70514590	7.23692799	-6.23511076
H	-1.34112442	6.81149244	-4.62360477
C	-1.83195496	0.84463900	-4.63635683

H	-2.33457613	1.47175324	-5.39065790
H	-2.56609702	0.59313899	-3.85685682
C	-1.27117968	-0.43042797	-5.27482462
H	-0.73787266	-1.00814354	-4.50058365
H	-0.52839845	-0.16421063	-6.05005550
C	-2.37853432	-1.29094505	-5.89420176
H	-3.11011100	-1.55410695	-5.10860634
H	-2.92935658	-0.69887519	-6.64890957
C	-1.83573413	-2.57143450	-6.53581715
H	-1.11549878	-2.34058261	-7.33863592
H	-2.64816809	-3.17303038	-6.97316504
H	-1.31788635	-3.19607687	-5.78936005
C	0.38510007	-1.50812018	2.89250207
C	1.98805106	-3.01096129	4.07571220
H	2.72171760	-2.30696774	3.65739202
H	2.20852590	-3.99116230	3.61491013
C	2.12906098	-3.10792804	5.59797621
H	1.87942278	-2.13413000	6.05865860
H	1.39306688	-3.83150244	5.99223423
C	3.54188418	-3.53479862	6.01387882
H	4.27463293	-2.80745387	5.61894751
H	3.78233361	-4.50336218	5.53899574
C	3.70037436	-3.65261292	7.53285551
H	3.49424481	-2.69035339	8.02999878
H	4.72294855	-3.95887995	7.80326796
H	3.00407434	-4.40000486	7.94760942
C	-0.41026223	-3.64686036	3.66329837
H	0.07786512	-4.58827066	3.96218157
H	-0.79257601	-3.77152205	2.63786292
C	-1.55154896	-3.31302953	4.63053703
H	-1.98348951	-2.33864617	4.34584236

H -1.13953841 -3.20527887 5.64941978

C -2.65654325 -4.38256598 4.62773705

H -2.21482325 -5.36961269 4.86235714

H -3.35127115 -4.15485191 5.45492125

C -3.44762182 -4.46551132 3.31645703

H -3.88107109 -3.48607874 3.05270362

H -4.27576590 -5.18664455 3.40351319

H -2.82285881 -4.78773785 2.46791601

O -1.46819985 3.67219424 0.30492765

O -1.57330227 5.13740444 -1.33099973

O -2.57873321 3.20208144 -1.57577384

N -1.90871215 4.02180958 -0.94176048

L2*Ce(NO_3)$_2^+$

E= -11882.232517, E0 = -11881.560848, G= 361.1090 at T=298.150 K

Cl -5.37176180 3.69900680 -0.42174765

Cl -4.12010527 -3.06592035 -3.69888377

N -1.57435906 1.31503391 0.19515544

N -0.24921601 4.08212948 2.06011415

N -1.12668526 -1.04497063 -0.97278678

N 1.20335293 -3.65389204 -1.77674294

O 0.03733343 1.83831060 2.15759802

O 0.84455293 -2.39075756 0.07019125

C -1.74472821 2.51593924 0.77995223

C -2.91562796 3.26557374 0.60402292

H -3.07979107 4.19858932 1.13907838

C -3.91691947 2.77663231 -0.22915383

C -3.75164390 1.53929985 -0.89868563

C -4.71432543 0.95591152 -1.77827358

H -5.64094973 1.49920523 -1.96981692

C -4.48943520 -0.25698864 -2.36817718

H -5.23480844 -0.69289857 -3.03515053

C	-3.28209949	-0.98139185	-2.12789989
C	-2.98117828	-2.25211406	-2.67678571
C	-1.76812088	-2.87147713	-2.39419365
H	-1.57106054	-3.86460018	-2.79385114
C	-0.84980536	-2.22428298	-1.55539858
C	-2.29950261	-0.42629093	-1.26221907
C	-2.53833389	0.84528905	-0.63742107
C	-0.60551989	2.83897948	1.71231592
C	0.46126199	-2.78928065	-1.07198119
C	-0.75104028	5.32997751	1.45479739
C	-1.70027912	6.12430382	2.35945368
H	-1.22014582	5.09325171	0.48864743
C	0.87956965	4.23216629	3.01174450
H	0.80249387	3.42483974	3.75433755
H	0.72866333	5.19232893	3.52974677
C	2.41766787	-4.20261192	-1.11949694
H	2.94066381	-3.36922312	-0.62325644
H	3.06393695	-4.58095980	-1.92611229
C	1.02363956	-3.96681976	-3.20785260
H	0.31842116	-3.24313521	-3.64055991
C	0.58483082	-5.40726280	-3.48863792
Ce	0.25847045	-0.26903543	1.04033720
N	-1.33193338	-1.61066687	3.04951525
O	-0.03670777	-1.40521550	3.13699627
O	-1.86966145	-1.15085793	1.95458436
O	-1.95804513	-2.17408037	3.90756607
N	2.61328292	0.78084582	-0.29241836
O	3.60555053	1.21334219	-0.82179904
O	2.61418366	0.31255138	0.93177485
O	1.45071471	0.74054140	-0.88195556
H	0.13830672	5.94133663	1.22405994

C	2.24953318	4.19850397	2.32476211
C	-2.18806362	7.40883112	1.67663741
H	-2.56443930	5.49862432	2.65013051
H	-1.18552589	6.38079309	3.30192542
C	-3.12436485	8.22769547	2.57023025
H	-1.31418574	8.02237892	1.39257824
H	-2.70393538	7.15104389	0.73327988
H	-3.45858145	9.14137840	2.05574083
H	-4.02114344	7.64932013	2.84637451
H	-2.61991811	8.53167915	3.50164032
C	3.38969564	4.41122341	3.32863092
H	2.37767410	3.22265792	1.82530618
H	2.29602695	4.97560549	1.53982341
C	4.76707315	4.36733007	2.66049123
H	3.25284648	5.38028669	3.84281373
H	3.33302021	3.63116479	4.10955286
H	5.56782770	4.51943827	3.40041065
H	4.93938828	3.39389801	2.17218041
H	4.86378098	5.15225697	1.89238167
C	2.11602783	-5.31257296	-0.10645812
H	1.99756312	-3.77418160	-3.69130325
C	0.40432814	-5.65779161	-4.99162102
H	1.33659458	-6.10811806	-3.08552790
H	-0.35693198	-5.62789965	-2.95239830
C	-0.01937350	-7.09732342	-5.29816151
H	-0.34738353	-4.95388126	-5.39391518
H	1.35121906	-5.42970943	-5.51337624
H	-0.14211828	-7.24877501	-6.38131666
H	0.73423231	-7.81772804	-4.94116306
H	-0.97786266	-7.34390688	-4.81292295
C	3.40276980	-5.83502626	0.54550809

H 1.44131219 -4.91854095 0.67229199

H 1.58771789 -6.14136934 -0.61000025

C 3.13020229 -6.94809389 1.56158948

H 4.08928061 -6.20555592 -0.23782155

H 3.92171144 -4.99656773 1.04472804

H 4.06748152 -7.30554628 2.01485682

H 2.47786808 -6.59136581 2.37554359

H 2.63554621 -7.81080770 1.08584547

L2*Pr(NO_3)$_3$(H_2O)

(H_2O in outer coordination sphere)

E= -12616.278499, E0 = -12615.567862, G= 379.2671 at T=298.150 K

Cl -5.45373631 3.64594746 -0.36233506

Cl -4.23733473 -3.08298326 -3.72143936

N -1.63138175 1.28029072 0.15902267

N -0.26997915 4.05509996 1.95617723

N -1.20925748 -1.09520602 -0.99140257

N 1.08793366 -3.71354103 -1.71837425

O -0.02753886 1.81279469 2.17026925

O 0.52533245 -2.67822123 0.22099334

C -1.79550397 2.45916438 0.75773787

C -2.97744823 3.21069050 0.61885798

H -3.13305807 4.13351345 1.17470980

C -3.97920084 2.72921753 -0.20344464

C -3.82012486 1.50646293 -0.89708978

C -4.78959084 0.93066472 -1.76754725

H -5.72065496 1.47195911 -1.94125640

C -4.57052994 -0.28137580 -2.36471725

H -5.32423878 -0.71665490 -3.02266550

C -3.36732316 -1.01271439 -2.13580513

C -3.08159351 -2.28978729 -2.68305635

C -1.89084482 -2.93255472 -2.38718271

H	-1.69707179	-3.93027329	-2.78053951
C	-0.97006112	-2.29426193	-1.53999639
C	-2.36617064	-0.46677396	-1.28114128
C	-2.59689546	0.81711155	-0.65358639
C	-0.63707024	2.78630519	1.67482960
C	0.27992368	-2.92570424	-0.98144770
C	-0.71129769	5.25470448	1.23177004
C	-1.59175587	6.19845009	2.05983782
H	-1.22405851	4.94561386	0.30919319
C	0.87282026	4.22867346	2.88071132
H	0.79464436	3.44338751	3.64688253
H	0.74590647	5.20750475	3.37211370
C	2.27973080	-4.25972939	-1.02789545
H	2.70736027	-3.45246553	-0.40882787
H	3.00815988	-4.51886272	-1.81249297
C	1.05004513	-3.80689526	-3.18338323
H	0.31538877	-3.08144188	-3.56228828
C	0.74936247	-5.21275997	-3.71233797
Pr	0.53391773	-0.26000503	0.88574195
N	-1.01802433	-1.20904231	3.20602965
O	0.25414073	-1.04836047	3.24795437
O	-1.56055796	-1.02586329	2.02438378
O	-1.69154227	-1.50255930	4.17499304
N	3.30701852	-0.39096686	1.83523548
O	2.63402700	0.72034639	1.77182174
O	2.64923072	-1.44161689	1.46166337
O	4.46104193	-0.43382856	2.21246839
N	1.69852149	0.85941505	-1.58343518
O	2.17695856	1.32337189	-2.60032272
O	1.76419449	-0.40565860	-1.29642010
O	1.07891762	1.58876848	-0.70886314

H	0.20039651	5.78824949	0.90922594
C	2.23379660	4.13573647	2.18208432
C	-2.02176833	7.43011236	1.25400293
H	-2.48419929	5.65872192	2.42674184
H	-1.04232812	6.52159929	2.96162963
C	-2.88734436	8.39588070	2.06906319
H	-1.12145138	7.95641041	0.88812971
H	-2.57508612	7.10401726	0.35416910
H	-3.18275332	9.26941872	1.46720028
H	-3.80814624	7.90314913	2.42242575
H	-2.34399772	8.76474380	2.95453238
C	3.38856649	4.24591398	3.18448496
H	2.30101824	3.16670632	1.66171551
H	2.32436395	4.92958546	1.41756260
C	4.75553989	4.07819891	2.51394367
H	3.33979940	5.21784019	3.71139073
H	3.26531148	3.46157169	3.95331788
H	5.57206869	4.16831207	3.24783897
H	4.83175135	3.08549237	2.04122758
H	4.91484690	4.84150600	1.73349774
C	1.97432947	-5.47870111	-0.15217362
H	2.03384399	-3.46759844	-3.55569243
C	0.73427176	-5.25414085	-5.24504423
H	1.50660396	-5.92078590	-3.33096647
H	-0.22330332	-5.56006098	-3.31692457
C	0.44786143	-6.65457201	-5.79557037
H	-0.02389486	-4.54385042	-5.62284756
H	1.70805597	-4.89707184	-5.62662458
H	0.44216105	-6.65547323	-6.89675617
H	1.21214628	-7.37570524	-5.46207619
H	-0.53304005	-7.02369976	-5.45319176

C 3.23094702 -5.97231865 0.57461488

H 1.21027589 -5.19514942 0.59121317

H 1.55075133 -6.29105234 -0.77076411

C 2.95513916 -7.18918276 1.46295202

H 4.01407719 -6.22123051 -0.16586767

H 3.63261175 -5.14747190 1.19019961

H 3.87124658 -7.52438784 1.97445095

H 2.20706725 -6.94957256 2.23665833

H 2.56852698 -8.03653717 0.87187904

O -4.11285162 0.22764041 2.15792370

H -3.29721355 -0.32432437 2.17030644

H -4.40072966 0.19272828 3.08601308

(NO_3^- in outer coordination sphere)

E= -12616.265677, E0 = -12615.555044, G= 380.9306 at T=298.150 K

Pr -0.69520229 -0.54104149 -0.04697223

Cl 3.77544761 1.97976458 4.88030815

Cl 2.64330840 5.53899860 -1.73164701

O -1.10553730 0.43634158 -2.18674374

O -0.78625804 -1.40101147 2.30643773

O -3.11039996 -0.88609236 0.18373486

O -4.13167667 -2.82830834 -0.04110397

O -1.96359289 -2.65723777 -0.40026802

O 1.02433634 -2.34041142 0.28040057

O 0.80212116 -1.56011403 -1.75295341

O 2.24118686 -3.18175840 -1.35196888

O -1.74117851 1.47989440 0.79913855

H -1.58787930 2.51683235 0.54162073

H -2.70890450 1.34337568 0.81185240

N 1.10366154 0.33247852 1.62462080

N 0.64210051 -2.60766959 3.59102678

N 0.77012837 1.54133463 -0.76427078

N	-0.78626162	1.64386523	-4.05211926
N	-3.11877894	-2.16299891	-0.08688645
N	1.39758873	-2.40059614	-0.96242309
C	1.24249685	-0.31023255	2.79033780
C	2.06035042	0.18465777	3.81944346
H	2.11384988	-0.31101286	4.78819895
C	2.77243257	1.35284185	3.59957242
C	2.67914796	2.03952742	2.36279440
C	3.36157537	3.25392246	2.05745959
H	4.03212929	3.67789435	2.80615187
C	3.16244602	3.88872242	0.86309516
H	3.66798019	4.83058167	0.64512146
C	2.27579427	3.35519552	-0.11833322
C	1.60940588	2.12136149	0.12114875
C	1.80302536	1.46261382	1.39697111
C	1.94983172	3.99461436	-1.33593965
C	1.05138052	3.42281604	-2.21885443
H	0.74896169	3.99901152	-3.08538556
C	0.49072194	2.16508698	-1.92099702
C	-0.52044427	1.39268804	-2.75488949
C	-0.20737053	2.69391370	-4.90019369
H	-0.18795285	2.28962088	-5.92627907
H	0.84728330	2.84441829	-4.62472343
C	-1.00828695	4.00476456	-4.85613251
H	-2.00228453	3.82826114	-5.30467510
H	-1.20749140	4.29032993	-3.80635095
C	-0.30228788	5.14217854	-5.60240602
H	-0.13093682	4.85268831	-6.65585375
H	0.70053834	5.30525923	-5.16265965
C	-1.09953892	6.44943476	-5.54028559
H	-2.08712840	6.33187008	-6.01599693

H	-0.57033288	7.26548624	-6.05715036
H	-1.26487410	6.75215960	-4.49385881
C	-1.82654285	0.80665773	-4.68723869
H	-2.28086972	1.41486418	-5.48600149
H	-2.59958911	0.59724098	-3.93350649
C	-1.26188552	-0.50087577	-5.25362301
H	-0.77063304	-1.05782080	-4.43726063
H	-0.48324701	-0.27334037	-6.00553799
C	-2.35664749	-1.36555731	-5.88873148
H	-3.12203455	-1.59444869	-5.12493753
H	-2.86977172	-0.79087555	-6.68250704
C	-1.80828643	-2.67268586	-6.46920681
H	-1.05788338	-2.47623849	-7.25330448
H	-2.61246729	-3.27917552	-6.91509533
H	-1.32397449	-3.27814984	-5.68529558
C	0.31271341	-1.49701953	2.90030599
C	1.99976647	-2.94646740	4.03970528
H	2.69921947	-2.19516230	3.64578772
H	2.27174640	-3.89989781	3.55158162
C	2.14008880	-3.08091354	5.55907249
H	1.83904469	-2.13548040	6.04755211
H	1.44075930	-3.85252047	5.92766237
C	3.57146120	-3.44758844	5.96900892
H	4.26804304	-2.67336750	5.59825230
H	3.86194849	-4.38876390	5.46785641
C	3.73076320	-3.60079503	7.48469925
H	3.47506046	-2.66485715	8.00832844
H	4.76655197	-3.86308765	7.75083351
H	3.07168770	-4.39380026	7.87467241
C	-0.36403596	-3.69498944	3.59936881
H	0.17485061	-4.62290335	3.84879422

H -0.75446022 -3.78849602 2.57352233

C -1.50756109 -3.46081901 4.59296656

H -1.98619497 -2.49435425 4.36075258

H -1.09044051 -3.38605857 5.61271763

C -2.56373620 -4.57791519 4.54306889

H -2.07280636 -5.55620956 4.70573092

H -3.24922514 -4.43505764 5.39653301

C -3.37870026 -4.61270428 3.24451423

H -3.87401772 -3.64471531 3.06140018

H -4.16181374 -5.38586473 3.29455924

H -2.75754094 -4.83434439 2.36195135

O -1.25395989 3.88567877 0.28543919

O -1.14449942 5.30302715 -1.38812637

O -2.18831396 3.39063811 -1.65806293

N -1.54719889 4.20889425 -0.94931620

L2*Pr(NO$_3$)$_2^+$

E= -12259.306664, E0 = -12258.634131, G= 361.9967 at T=298.150 K

Cl -5.39641762 3.67587280 -0.38351601

Cl -4.14047480 -3.09310985 -3.62966561

N -1.58849895 1.31840777 0.22415876

N -0.26305139 4.08742857 2.07211971

N -1.13934803 -1.05580342 -0.93505263

N 1.19321871 -3.65127921 -1.74041617

O 0.00217906 1.84279275 2.22336578

O 0.81977284 -2.43771768 0.13793729

C -1.76425743 2.50893760 0.81608081

C -2.94175577 3.25480461 0.64444602

H -3.10993266 4.18452454 1.18464625

C -3.93527293 2.76507473 -0.19181851

C -3.76166797 1.53038311 -0.86827910

C -4.72206974 0.94507802 -1.74854445

H	-5.64909697	1.48670554	-1.94261146
C	-4.49634027	-0.26993024	-2.33352447
H	-5.24131775	-0.70856482	-2.99909878
C	-3.29084897	-0.99432069	-2.08609867
C	-2.99545979	-2.27515984	-2.61922145
C	-1.78893781	-2.89624929	-2.32921481
H	-1.59326756	-3.89494514	-2.71660233
C	-0.86602122	-2.23853230	-1.49844074
C	-2.30406237	-0.43784016	-1.22625041
C	-2.54442191	0.84459269	-0.60423243
C	-0.62367964	2.83631349	1.75393391
C	0.44995534	-2.80623317	-1.01172948
C	-0.75582308	5.32278872	1.43506670
C	-1.69229770	6.15065098	2.32268357
H	-1.23385084	5.06547785	0.47848493
C	0.86863887	4.25126219	3.01803827
H	0.78325707	3.46467113	3.78177977
H	0.72967291	5.22665310	3.51024199
C	2.41337681	-4.20568037	-1.09943986
H	2.93056750	-3.37958884	-0.58509076
H	3.06118298	-4.55966234	-1.91588485
C	1.01704073	-3.92377043	-3.18004107
H	0.29420444	-3.20437932	-3.59036231
C	0.61012769	-5.36459446	-3.50275803
Pr	0.27168879	-0.27385667	1.09871256
N	-1.36439455	-1.56270671	3.09270692
O	-0.05915033	-1.44040000	3.16333246
O	-1.89224374	-1.04274535	2.02048683
O	-2.01131368	-2.10762310	3.94904184
N	2.59127474	0.69570929	-0.33935228
O	3.57876921	1.08819330	-0.90965748

O	2.58719850	0.40033033	0.93870085
O	1.44455111	0.52795243	-0.93299890
H	0.13735200	5.91922569	1.18100762
C	2.23704743	4.18227863	2.33037877
C	-2.16938829	7.42322540	1.61066365
H	-2.56219149	5.54194403	2.63221288
H	-1.17133963	6.42435646	3.25680923
C	-3.09808493	8.27044678	2.48554754
H	-1.29052246	8.02252865	1.31206679
H	-2.68816829	7.14823580	0.67373455
H	-3.42379737	9.17581272	1.95133483
H	-4.00028515	7.70662165	2.77391124
H	-2.59107041	8.58983898	3.41036463
C	3.38096285	4.40433502	3.32795262
H	2.35219264	3.19281483	1.85485065
H	2.29270291	4.94018126	1.52729189
C	4.75694847	4.32668257	2.65996218
H	3.25744724	5.38686275	3.81954885
H	3.31499195	3.64343882	4.12685013
H	5.56046629	4.48585272	3.39543581
H	4.91551447	3.33978558	2.19463229
H	4.86295366	5.09196234	1.87341952
C	2.12276411	-5.34093666	-0.11173600
H	1.98442113	-3.69357157	-3.66049099
C	0.43877697	-5.57845926	-5.01242161
H	1.37535203	-6.06034088	-3.11640286
H	-0.32812181	-5.62032652	-2.97573495
C	0.04899942	-7.01856518	-5.35867643
H	-0.32803389	-4.88153601	-5.39804029
H	1.38138795	-5.31504679	-5.52517271
H	-0.06750580	-7.14431667	-6.44576931

H 0.81801134 -7.73069954 -5.01833630

H -0.90469599 -7.30006599 -4.88302851

C 3.41503954 -5.87093639 0.52291328

H 1.44889688 -4.96917200 0.67853534

H 1.59766388 -6.16094112 -0.63287002

C 3.15292692 -7.00948858 1.51323175

H 4.10091591 -6.21856117 -0.27141842

H 3.93090105 -5.04170322 1.04028189

H 4.09395838 -7.37181902 1.95473516

H 2.50131583 -6.67611980 2.33756232

H 2.66189194 -7.86373615 1.01880145

L2*Nd($(NO_3)_3$)(H_2O)

(H_2O in outer coordination sphere)

E= -13003.181345, E0 = -13002.470367, G= 379.3146 at T=298.150 K

Cl -5.48306513 3.62480307 -0.29397711

Cl -4.32288647 -3.14390564 -3.59373236

N -1.66019404 1.25357819 0.20753388

N -0.26813528 4.05006313 1.94254708

N -1.26416516 -1.13283002 -0.92786747

N 1.06807792 -3.68355918 -1.68759596

O -0.07035071 1.81109416 2.24438834

O 0.46269599 -2.75029564 0.29264635

C -1.81336474 2.44039130 0.79231060

C -2.99384570 3.19439077 0.65836912

H -3.13509893 4.12752962 1.20145106

C -4.00971222 2.70570111 -0.14263384

C -3.86211610 1.47290266 -0.82086045

C -4.84755516 0.88641441 -1.67029309

H -5.78023005 1.42832315 -1.83407950

C -4.64289141 -0.33076030 -2.25764060

H -5.40811443 -0.77122390 -2.89854193

C	-3.43511939	-1.06073010	-2.03955126
C	-3.15453434	-2.34055376	-2.57874918
C	-1.95822847	-2.97882271	-2.29821301
H	-1.76315451	-3.97671914	-2.69118404
C	-1.02864075	-2.32904029	-1.46611607
C	-2.42210770	-0.50601095	-1.20505762
C	-2.63859653	0.78605831	-0.58587468
C	-0.65144658	2.77580237	1.70683420
C	0.23804326	-2.95379186	-0.91756630
C	-0.67531633	5.22211885	1.15623879
C	-1.53028619	6.23192835	1.93138325
H	-1.19707477	4.87924051	0.25066924
C	0.86938477	4.23987865	2.87014556
H	0.76560205	3.49139833	3.66950274
H	0.76016629	5.24205732	3.31674337
C	2.28327775	-4.21073437	-1.02283216
H	2.68045640	-3.41243529	-0.37239906
H	3.01844382	-4.40804482	-1.81904697
C	1.02161944	-3.72430158	-3.15464568
H	0.25091767	-3.02040672	-3.50170398
C	0.77839100	-5.12214088	-3.73190379
Nd	0.49895307	-0.29528913	0.98471403
N	-1.11655092	-1.25480592	3.25001812
O	0.15872703	-1.13815582	3.31030226
O	-1.63939512	-1.00990903	2.07107234
O	-1.81099498	-1.56004918	4.20106983
N	3.24560142	-0.46877268	1.95740163
O	2.56010675	0.63790548	1.94670117
O	2.60135531	-1.50640261	1.53235328
O	4.39985371	-0.51527512	2.33579803
N	1.65537167	0.87102842	-1.46308458

O	2.12930584	1.33032131	-2.48566771
O	1.69794309	-0.40175349	-1.19091904
O	1.07092631	1.60231888	-0.57341218
H	0.24995054	5.71316624	0.80581915
C	2.23500967	4.08414173	2.19208288
C	-1.92628276	7.43078518	1.06122923
H	-2.43783402	5.73705816	2.32374287
H	-0.97338891	6.58728933	2.81629252
C	-2.76643205	8.46074486	1.82259738
H	-1.01206732	7.91317177	0.67030865
H	-2.48737097	7.07284641	0.17846540
H	-3.03814578	9.30911541	1.17524970
H	-3.70024872	8.01189041	2.19956589
H	-2.21398807	8.86104202	2.68857789
C	3.38136768	4.20989180	3.20217586
H	2.28305149	3.09211206	1.71468616
H	2.35308552	4.84112740	1.39468920
C	4.75058222	3.97599244	2.55640817
H	3.35291696	5.20572901	3.68413687
H	3.22926354	3.46463680	4.00404119
H	5.56152201	4.08009863	3.29468751
H	4.80584812	2.96023846	2.13217044
H	4.93773127	4.69700146	1.74259782
C	2.02944851	-5.47465038	-0.19597931
H	1.98484266	-3.32609248	-3.52213144
C	0.75293595	-5.10965204	-5.26499414
H	1.56865668	-5.80950785	-3.38109183
H	-0.17535757	-5.52480984	-3.34286404
C	0.52579266	-6.50131893	-5.86333418
H	-0.03967197	-4.42171526	-5.61235142
H	1.70656085	-4.69546270	-5.63965988

H 0.51110888 -6.46360636 -6.96377993

H 1.32477260 -7.19807291 -5.56085396

H -0.43443191 -6.92688036 -5.52786875

C 3.30775189 -5.94557190 0.50781852

H 1.25816965 -5.25054932 0.56013751

H 1.63600945 -6.27849007 -0.84491885

C 3.08548522 -7.20889616 1.34477437

H 4.09819937 -6.13072109 -0.24348538

H 3.67661715 -5.13084888 1.15642023

H 4.01579618 -7.52530718 1.84239972

H 2.32884312 -7.03395748 2.12740779

H 2.73502564 -8.04681396 0.71873111

O -4.21508408 0.18133336 2.15964150

H -3.38237858 -0.34427053 2.19742179

H -4.51571035 0.15925528 3.08399057

(NO_3^- in outer coordination sphere)

E= -13003.170460, E0 = -13002.458810, G= 381.7123 at T=298.150 K

Nd -0.71445066 -0.54265755 -0.03806892

Cl 3.77309155 2.00870347 4.88240099

Cl 2.65678215 5.53405046 -1.74529326

O -1.12710333 0.45573783 -2.16159058

O -0.80035657 -1.35960197 2.33530307

O -3.13239837 -0.89884204 0.15804134

O -4.12135172 -2.84696150 -0.14233173

O -1.95137239 -2.63389421 -0.46769580

O 1.00918341 -2.33145666 0.31257561

O 0.83643264 -1.51906681 -1.71283102

O 2.28903437 -3.12391043 -1.29561281

O -1.76882923 1.47456300 0.83753932

H -1.60650229 2.49262476 0.57086080

H -2.73661876 1.34219790 0.82419801

N	1.11174846	0.34417188	1.63223422
N	0.64353085	-2.59420896	3.57678938
N	0.76895511	1.54976356	-0.76002955
N	-0.79159254	1.63504875	-4.04293776
N	-3.11903906	-2.16332102	-0.15287037
N	1.41984344	-2.36269689	-0.91881847
C	1.24019277	-0.28634518	2.80089450
C	2.05503368	0.21264388	3.83197975
H	2.10358858	-0.27733102	4.80400038
C	2.77197671	1.37512457	3.60430145
C	2.68533325	2.05261230	2.36237812
C	3.37163377	3.26403689	2.05111527
H	4.04279470	3.69092107	2.79751277
C	3.17365146	3.89154863	0.85370797
H	3.68106842	4.83129930	0.63067967
C	2.28362489	3.35660529	-0.12496827
C	1.61395717	2.12644815	0.11813687
C	1.81074226	1.47032130	1.39823353
C	1.95601714	3.99493217	-1.34355903
C	1.05287910	3.42464948	-2.22111869
H	0.74964964	3.99866438	-3.08895588
C	0.48721987	2.16968226	-1.91585219
C	-0.53247750	1.39608586	-2.74195576
C	-0.20799778	2.67761087	-4.89676237
H	-0.18994799	2.26814175	-5.92076635
H	0.84735197	2.82519197	-4.62212420
C	-1.00368583	3.99194145	-4.85750628
H	-1.99662030	3.81886911	-5.30973339
H	-1.20606422	4.27642441	-3.80790067
C	-0.29115510	5.12696505	-5.60107183
H	-0.12056493	4.83872843	-6.65497541

H	0.71232772	5.28409100	-5.16055059
C	-1.08146465	6.43834734	-5.53666306
H	-2.07004714	6.32653141	-6.01170254
H	-0.54849261	7.25232506	-6.05293083
H	-1.24426782	6.74051571	-4.48964787
C	-1.83740962	0.79921269	-4.67081738
H	-2.28564000	1.40168905	-5.47738838
H	-2.61326742	0.60447264	-3.91592169
C	-1.28171456	-0.51924151	-5.22048426
H	-0.79184544	-1.06785083	-4.39765263
H	-0.50346559	-0.30666026	-5.97716570
C	-2.38331604	-1.38624704	-5.84035492
H	-3.14785147	-1.59894586	-5.07101297
H	-2.89495301	-0.82018590	-6.64125061
C	-1.84473276	-2.70535922	-6.40257168
H	-1.09456587	-2.52557421	-7.19090748
H	-2.65383196	-3.31299853	-6.83788443
H	-1.36305523	-3.30228567	-5.61055374
C	0.30424860	-1.47340000	2.91051102
C	2.00309181	-2.92982531	4.02215195
H	2.69839692	-2.16833878	3.64050007
H	2.28290296	-3.87414479	3.52140450
C	2.13728738	-3.08155084	5.54037476
H	1.82727695	-2.14325237	6.03709269
H	1.43998218	-3.86095381	5.89608288
C	3.56737494	-3.44404769	5.95838356
H	4.26342440	-2.66409850	5.59883070
H	3.86682510	-4.38076591	5.45435238
C	3.71087813	-3.60486770	7.47482586
H	3.44100094	-2.67311811	8.00002003
H	4.74313688	-3.86268401	7.75478220

H 3.04945064 -4.40308571 7.85157013
C -0.35667744 -3.68743992 3.56758690
H 0.18811592 -4.61665678 3.79832935
H -0.74665833 -3.76441169 2.54014611
C -1.50101316 -3.47880554 4.56575537
H -1.98298967 -2.50932908 4.35447359
H -1.08484960 -3.42559719 5.58725452
C -2.55322671 -4.59826040 4.48971939
H -2.05923367 -5.57832575 4.63089371
H -3.23993015 -4.47668934 5.34548712
C -3.36684155 -4.60658026 3.18982625
H -3.86374760 -3.63574862 3.02717161
H -4.14850187 -5.38207531 3.22250628
H -2.74455428 -4.80842781 2.30339050
O -1.23806310 3.89142060 0.31028804
O -1.08751142 5.32111645 -1.34957349
O -2.13550758 3.41609955 -1.65382814
N -1.50368273 4.22599173 -0.92240453

L2*Nd(NO_3)$_2^+$

E= -12646.211128 , E0 = -12645.538252, G= 362.3006 at T=298.150 K

Cl -5.40932178 3.67242122 -0.38010222
Cl -4.14332390 -3.08037496 -3.64773250
N -1.58766496 1.33563316 0.19888641
N -0.27079800 4.08988667 2.06938624
N -1.14694643 -1.05053735 -0.94528294
N 1.17601597 -3.66043973 -1.71192229
O -0.00372904 1.84451151 2.20993114
O 0.77084213 -2.45895410 0.16981509
C -1.77030528 2.51517606 0.80528599
C -2.95423365 3.25511575 0.64505595
H -3.12758088 4.17735243 1.19649947

C	-3.94365978	2.76864386	-0.19650868
C	-3.76462173	1.54033399	-0.88382965
C	-4.72340393	0.95690840	-1.76687527
H	-5.65147972	1.49716175	-1.95967042
C	-4.49514771	-0.25605628	-2.35500813
H	-5.23917007	-0.69372338	-3.02230930
C	-3.29109287	-0.98221695	-2.10515857
C	-2.99891520	-2.26685429	-2.63362193
C	-1.79890597	-2.89341998	-2.33371520
H	-1.60488677	-3.89446259	-2.71635771
C	-0.87785083	-2.23773384	-1.49780321
C	-2.30412006	-0.42765746	-1.24509978
C	-2.54354501	0.85897899	-0.62488461
C	-0.62747639	2.83859515	1.74715459
C	0.42619050	-2.81607676	-0.98959094
C	-0.77329904	5.32705164	1.44380510
C	-1.70837891	6.14416647	2.34268236
H	-1.25560737	5.07553196	0.48774678
C	0.86859459	4.25169802	3.00682473
H	0.79377872	3.45766020	3.76391554
H	0.72924829	5.22209692	3.50870109
C	2.38358331	-4.22390938	-1.05605328
H	2.89640117	-3.40253448	-0.52970219
H	3.04249573	-4.57580900	-1.86446142
C	1.02370012	-3.91537642	-3.15723133
H	0.30708811	-3.19153380	-3.57079768
C	0.62400836	-5.35228539	-3.50475478
Nd	0.30163482	-0.25344494	1.05552733
N	-1.19422495	-1.56015015	3.12231970
O	0.10750161	-1.40781641	3.13413668
O	-1.78392243	-1.05825925	2.07298231

O	-1.79030120	-2.11647081	4.00844860
N	2.61765623	0.65524304	-0.41939208
O	3.60016894	1.03996599	-1.00455165
O	2.55786657	0.58983701	0.88813788
O	1.53043032	0.27512273	-1.02653694
H	0.11551759	5.92956352	1.18895113
C	2.22995687	4.19406033	2.30420041
C	-2.18867159	7.42384386	1.64574230
H	-2.57672906	5.53122520	2.64837527
H	-1.18504608	6.40821838	3.27820015
C	-3.11681724	8.26012421	2.53167510
H	-1.31098056	8.02742577	1.35239553
H	-2.70877719	7.15885496	0.70663482
H	-3.44440532	9.17111015	2.00828242
H	-4.01791668	7.69214821	2.81528330
H	-2.60852265	8.56967068	3.45911646
C	3.38377523	4.40135241	3.29360700
H	2.34157419	3.21244144	1.81177795
H	2.27697301	4.96572113	1.51363921
C	4.75212765	4.33939934	2.60846829
H	3.26369548	5.37407589	3.80520654
H	3.32856917	3.62511134	4.07842064
H	5.56426382	4.48404264	3.33742428
H	4.90545511	3.36271238	2.12055945
H	4.84820414	5.12122202	1.83702862
C	2.07385421	-5.36295700	-0.07872106
H	1.99803340	-3.67719674	-3.61951613
C	0.48258445	-5.54759789	-5.02001572
H	1.38198125	-6.05244112	-3.11205769
H	-0.32435355	-5.61471558	-2.99952388
C	0.10011003	-6.98306942	-5.39263153

H -0.27642110 -4.84567928 -5.41201830

H 1.43518031 -5.27753592 -5.51036978

H 0.00444829 -7.09425879 -6.48335505

H 0.86277926 -7.69973040 -5.04752636

H -0.86235690 -7.27112961 -4.93908024

C 3.35449457 -5.89850473 0.57461149

H 1.38808811 -4.99294424 0.70203596

H 1.55570984 -6.17931557 -0.61246669

C 3.07484794 -7.04157543 1.55486739

H 4.05305815 -6.24301958 -0.20998926

H 3.86265516 -5.07245827 1.10463572

H 4.00843668 -7.40721178 2.00931597

H 2.41057038 -6.71130657 2.37025237

H 2.59076071 -7.89267111 1.04827654

L2*Pm(NO_3)$_3$(H_2O)

(H_2O in outer coordination sphere)

E= -13400.043888, E0 = -13399.332734, G= 379.4690 at T=298.150 K

Cl -5.47805405 3.61840534 -0.31192183

Cl -4.34088802 -3.16435575 -3.58919096

N -1.64773500 1.25201511 0.16953260

N -0.28521395 4.02737904 1.96376204

N -1.26818037 -1.14379692 -0.94989157

N 1.07262146 -3.68067575 -1.75478482

O -0.11362352 1.78334999 2.26442075

O 0.53093022 -2.69785523 0.21724044

C -1.80686045 2.43172312 0.76738077

C -2.98925519 3.18383312 0.63939273

H -3.12903595 4.11432838 1.18762696

C -4.00518703 2.69828439 -0.16274418

C -3.85974526 1.46483219 -0.84019846

C -4.85061073 0.87601167 -1.68175662

H -5.78430700 1.41768003 -1.84048033

C -4.65034294 -0.34253535 -2.26770115

H -5.41971016 -0.78432643 -2.90269709

C -3.44178891 -1.07211256 -2.05414128

C -3.16382074 -2.35396910 -2.58992577

C -1.96254694 -2.98739147 -2.32101774

H -1.77014065 -3.98514915 -2.71526241

C -1.02748883 -2.33381343 -1.49705327

C -2.42430019 -0.51629692 -1.22594571

C -2.63341832 0.78049833 -0.61287898

C -0.66464710 2.75512648 1.71097338

C 0.26138616 -2.93645883 -0.97670949

C -0.64853430 5.19592667 1.15136182

C -1.50560927 6.23295403 1.88707614

H -1.15208828 4.84929323 0.23684524

C 0.81554335 4.21135855 2.93560362

H 0.68367696 3.45229101 3.72072005

H 0.68468392 5.20727539 3.39052153

C 2.31116295 -4.18346548 -1.11415029

H 2.72270179 -3.36661601 -0.49677035

H 3.02256203 -4.39845848 -1.92718959

C 0.97738302 -3.77289963 -3.21725512

H 0.20257597 -3.07416558 -3.56518674

C 0.70517993 -5.18803930 -3.73704457

Pm 0.51450884 -0.27297559 0.94822425

N -1.02939558 -1.23404860 3.24300838

O 0.24209028 -1.10117185 3.28739262

O -1.56971538 -1.00717199 2.06580853

O -1.71205568 -1.54028273 4.20254803

N 3.22060204 -0.42359716 2.00531626

O 2.49945164 0.65194726 2.06984210

O	2.63293600	-1.43471563	1.44662511
O	4.35875750	-0.47616142	2.43089557
N	1.62223399	1.05234706	-1.42822707
O	2.05308533	1.58256507	-2.43562555
O	1.57110715	-0.24167506	-1.29517138
O	1.18104279	1.72613740	-0.41879544
H	0.29446819	5.66489315	0.81818289
C	2.20778656	4.06849337	2.31104302
C	-1.85139596	7.42672873	0.98889756
H	-2.43440366	5.76202631	2.25868368
H	-0.96833295	6.59080935	2.78295588
C	-2.69210362	8.48445702	1.71052909
H	-0.91612226	7.88457823	0.61879742
H	-2.39348435	7.06747627	0.09488240
H	-2.92706656	9.32878780	1.04378808
H	-3.64569759	8.06012440	2.06581903
H	-2.15723014	8.88588047	2.58696628
C	3.31063747	4.17604828	3.37046099
H	2.27578998	3.08552909	1.81801212
H	2.35867357	4.83996868	1.53340352
C	4.70606852	3.95424938	2.77874470
H	3.26139379	5.16285992	3.86909223
H	3.12567067	3.41592312	4.15111542
H	5.48527908	4.04601955	3.55203676
H	4.78053522	2.94594049	2.34000254
H	4.92623806	4.68928051	1.98600066
C	2.09172750	-5.42576265	-0.24543506
H	1.93183291	-3.39819288	-3.62945199
C	0.63009602	-5.22891998	-5.26799774
H	1.50132298	-5.86919641	-3.38752937
H	-0.23808274	-5.56933737	-3.30342960

C 0.36976877 -6.63805056 -5.80921268

H -0.16630068 -4.54465866 -5.61398792

H 1.57511997 -4.83872986 -5.68749905

H 0.32016867 -6.63866997 -6.90926933

H 1.17090881 -7.33275604 -5.50778913

H -0.58347201 -7.04071951 -5.42866421

C 3.39375138 -5.86566210 0.43463427

H 1.34030151 -5.18672609 0.52584249

H 1.68756747 -6.25079727 -0.86019433

C 3.20725060 -7.10772038 1.31119847

H 4.16486216 -6.06395388 -0.33324507

H 3.77198100 -5.03028440 1.05073643

H 4.15423107 -7.40203524 1.79054892

H 2.47137046 -6.91817856 2.11002731

H 2.84749460 -7.96547079 0.71817708

O -4.16168785 0.14027429 2.12275457

H -3.31895137 -0.36784601 2.17991424

H -4.45988894 0.15193707 3.04805541

(NO_3^- in outer coordination sphere)

E= -13400.033374, E0 = -13399.321796, G= 381.8308 at T=298.150 K

Pm -0.74179590 -0.49398690 -0.03551845

Cl 3.71114516 2.03300261 4.91167736

Cl 2.63304567 5.57379484 -1.71834087

O -1.12365580 0.47990125 -2.16611958

O -0.85284269 -1.31729460 2.34999084

O -3.13332224 -0.86393976 0.24313971

O -4.10401869 -2.84292865 0.16286688

O -1.94450247 -2.63757634 -0.22880927

O 1.00139725 -2.25111318 0.31835982

O 0.60699046 -1.65339613 -1.74721897

O 2.05937409 -3.25878119 -1.32935262

O	-1.79142547	1.51803708	0.81951702
H	-1.64480400	2.53689575	0.54975659
H	-2.75621438	1.38143039	0.88366634
N	1.07772338	0.37366372	1.63474381
N	0.60397130	-2.57075763	3.56060243
N	0.76244283	1.57737517	-0.75713712
N	-0.76263553	1.64215779	-4.05363894
N	-3.10951424	-2.15366721	0.06242230
N	1.26040280	-2.42987704	-0.93953317
C	1.19792724	-0.25922012	2.80212831
C	2.00510716	0.23692466	3.84089327
H	2.04901838	-0.26018372	4.80969715
C	2.72065902	1.40216208	3.62412500
C	2.64137220	2.08383608	2.38412690
C	3.32446051	3.29999375	2.08223367
H	3.98685837	3.72758675	2.83606601
C	3.13456798	3.93073153	0.88524854
H	3.63952374	4.87361956	0.67003775
C	2.25720930	3.39326739	-0.10380992
C	1.59342980	2.15872836	0.13041729
C	1.77825975	1.50083137	1.41079223
C	1.93764555	4.03046227	-1.32542837
C	1.04760790	3.45503926	-2.21357322
H	0.74909598	4.02784538	-3.08409572
C	0.48669675	2.19643331	-1.91444421
C	-0.52313936	1.41532183	-2.74709678
C	-0.17000608	2.68188190	-4.90461683
H	-0.13076586	2.26507592	-5.92503977
H	0.87913132	2.83818865	-4.61145353
C	-0.97330886	3.99202871	-4.88843918
H	-1.95544696	3.81211448	-5.36110783

H	-1.19915366	4.27860069	-3.84419608
C	-0.25072783	5.12794495	-5.62088346
H	-0.05586531	4.83688259	-6.66975307
H	0.74238187	5.29150343	-5.15943241
C	-1.04859698	6.43561888	-5.57818222
H	-2.02626920	6.31714916	-6.07371283
H	-0.50872999	7.25033426	-6.08602238
H	-1.23499787	6.74076843	-4.53596544
C	-1.79277623	0.79413193	-4.69167852
H	-2.22677660	1.38437474	-5.51486111
H	-2.58309960	0.60577387	-3.95033240
C	-1.22010148	-0.52910298	-5.21190834
H	-0.74797976	-1.06617808	-4.37119818
H	-0.42551294	-0.32266831	-5.95317030
C	-2.30379677	-1.40783799	-5.84677267
H	-3.08704209	-1.61149502	-5.09393263
H	-2.79660511	-0.85553914	-6.66880322
C	-1.74721611	-2.73376966	-6.37453365
H	-0.97651309	-2.56376839	-7.14504290
H	-2.54284453	-3.35011888	-6.82226753
H	-1.28533351	-3.31696892	-5.56084681
C	0.25658733	-1.44350302	2.90857148
C	1.97355008	-2.91491985	3.96654820
H	2.65859461	-2.14178181	3.58966541
H	2.24406314	-3.84450960	3.43383288
C	2.14670682	-3.10744524	5.47611713
H	1.85143900	-2.18303394	6.00648737
H	1.45947301	-3.89686823	5.82926369
C	3.58826709	-3.48112345	5.84162569
H	4.27317190	-2.68983936	5.48537731
H	3.87234378	-4.40130472	5.29956388

C 3.78073239 -3.69006991 7.34679317

H 3.53180575 -2.77591062 7.91068268

H 4.82318544 -3.95651960 7.58087635

H 3.13384247 -4.50074148 7.72048426

C -0.39677420 -3.66331697 3.55573487

H 0.15324552 -4.59667826 3.75597024

H -0.81342572 -3.72024369 2.53731847

C -1.51323473 -3.47170734 4.58819675

H -2.02093291 -2.51325774 4.38592434

H -1.06578827 -3.40235233 5.59549475

C -2.54325199 -4.61351204 4.55519772

H -2.02416945 -5.58025360 4.69850016

H -3.21036601 -4.49337912 5.42659903

C -3.38866377 -4.65984631 3.27681684

H -3.91028166 -3.70296931 3.11040282

H -4.15236712 -5.45121336 3.34174299

H -2.78474975 -4.86171532 2.37800360

O -1.28448212 3.93749785 0.28535268

O -1.10257828 5.36036825 -1.37673473

O -2.12570882 3.44500875 -1.69937849

N -1.52034271 4.26389551 -0.95526940

L2*Pm(NO_3)$_2^+$

E= -13043.075840, E0 = -13042.402793, G= 362.5775 at T=298.150 K

Cl -5.40494204 3.67035246 -0.36559176

Cl -4.14995766 -3.09151530 -3.63096952

N -1.60707581 1.30371678 0.24178673

N -0.26240143 4.05865574 2.08433795

N -1.15030372 -1.06002474 -0.93406308

N 1.19035065 -3.64475465 -1.74256170

O -0.02444683 1.81158924 2.25601387

O 0.81781644 -2.42378855 0.13141863

C	-1.77891397	2.48868012	0.83766127
C	-2.95463657	3.23988366	0.66686022
H	-3.12019396	4.16856050	1.21003163
C	-3.94599915	2.75639439	-0.17424841
C	-3.77260423	1.52512598	-0.85766679
C	-4.73048735	0.94454443	-1.74369621
H	-5.65637445	1.48783720	-1.93860233
C	-4.50406647	-0.26891372	-2.33181834
H	-5.24808168	-0.70470273	-3.00041056
C	-3.29971361	-0.99569243	-2.08437181
C	-3.00437331	-2.27648211	-2.61932898
C	-1.79893720	-2.89804029	-2.32997513
H	-1.60294139	-3.89599991	-2.71923137
C	-0.87567008	-2.24050808	-1.49784589
C	-2.31337070	-0.44378129	-1.22176647
C	-2.55697823	0.83704382	-0.59189200
C	-0.63338649	2.80781841	1.77807403
C	0.44566518	-2.80085349	-1.01320291
C	-0.74028355	5.29123545	1.43036807
C	-1.66361868	6.14497566	2.30690074
H	-1.22328115	5.02678871	0.47828805
C	0.87020016	4.22106409	3.02944350
H	0.77646476	3.44224930	3.80018044
H	0.74040055	5.20245409	3.51211905
C	2.41837859	-4.18624878	-1.10508621
H	2.93204570	-3.35272384	-0.59917682
H	3.06388021	-4.54119730	-1.92292166
C	1.00973094	-3.92384863	-3.18025112
H	0.28194016	-3.20989394	-3.59135294
C	0.60966992	-5.36814117	-3.49587631
Pm	0.24870405	-0.28524241	1.10229540

N	-1.40434241	-1.60918534	3.02989459
O	-0.11500743	-1.41616285	3.15927696
O	-1.90424240	-1.13988698	1.92294025
O	-2.06379461	-2.17299652	3.86555624
N	2.45881510	0.87945753	-0.31707495
O	3.40958929	1.36445487	-0.88057125
O	2.54291868	0.35959482	0.88234574
O	1.26915169	0.83002299	-0.84041637
H	0.16019642	5.87167454	1.16532302
C	2.23818469	4.13215017	2.34351540
C	-2.11756539	7.41683817	1.57863533
H	-2.54434991	5.55507183	2.62221551
H	-1.13961732	6.42113590	3.23855710
C	-3.03349853	8.29067707	2.44071198
H	-1.22772896	7.99714231	1.27507961
H	-2.63880348	7.13955975	0.64376831
H	-3.34193802	9.19480705	1.89422679
H	-3.94612598	7.74630833	2.73358512
H	-2.52323604	8.61273575	3.36280370
C	3.38412166	4.35477018	3.33857465
H	2.34425020	3.13625026	1.87982142
H	2.30140829	4.87956524	1.53139293
C	4.75901937	4.25811720	2.67072535
H	3.26934791	5.34353924	3.81966400
H	3.31173801	3.60307407	4.14557171
H	5.56427908	4.41584730	3.40461516
H	4.90785980	3.26594687	2.21350932
H	4.87186432	5.01553297	1.87761700
C	2.14245248	-5.31666040	-0.10767048
H	1.97377455	-3.69079447	-3.66597533
C	0.43541175	-5.58832169	-5.00430250

H 1.38045943 -6.05761003 -3.10944724

H -0.32536930 -5.62722921 -2.96475744

C 0.06313675 -7.03420448 -5.34591818

H -0.34103164 -4.90165186 -5.38911343

H 1.37304497 -5.31461239 -5.52082968

H -0.05651453 -7.16339684 -6.43224764

H 0.84363377 -7.73507547 -5.00831699

H -0.88428688 -7.32880068 -4.86564589

C 3.44295001 -5.82752800 0.52600020

H 1.46796656 -4.94535112 0.68222547

H 1.62402785 -6.14618731 -0.62032449

C 3.19810891 -6.96109295 1.52639711

H 4.12953091 -6.17363453 -0.26838976

H 3.95125270 -4.98818111 1.03451252

H 4.14554071 -7.30892801 1.96594679

H 2.54658008 -6.62862778 2.35114193

H 2.71462989 -7.82477093 1.04097807

L2*Sm(NO_3)$_3$(H_2O)

(H_2O in outer coordination sphere)

E= -13806.980575, E0 = -13806.269714, G= 378.9535 at T=298.150 K

Cl -5.48811531 3.63062072 -0.33216551

Cl -4.36545563 -3.19699740 -3.52497315

N -1.66171885 1.26337349 0.17219321

N -0.29028410 4.04469109 1.94784427

N -1.28090835 -1.14052904 -0.92614281

N 1.06073654 -3.67442393 -1.72933197

O -0.12966439 1.80157506 2.25801730

O 0.52023965 -2.69943118 0.24966526

C -1.81904459 2.44864726 0.75833422

C -2.99894166 3.20299625 0.62142724

H -3.13639617 4.14037991 1.15841544

C	-4.01700592	2.70933747	-0.17292780
C	-3.87627387	1.46586180	-0.83336532
C	-4.87037897	0.86624616	-1.66370440
H	-5.80473948	1.40564215	-1.82620203
C	-4.67166901	-0.35977012	-2.23473597
H	-5.44338131	-0.80974150	-2.86113453
C	-3.46157193	-1.08622801	-2.01806021
C	-3.18448353	-2.37390900	-2.54052424
C	-1.98056972	-3.00154757	-2.27080297
H	-1.78572083	-4.00256205	-2.65576124
C	-1.04132962	-2.33650327	-1.46066558
C	-2.44072318	-0.51949686	-1.20180547
C	-2.64962721	0.78334159	-0.60176319
C	-0.67658043	2.77307224	1.70046258
C	0.25153738	-2.93478870	-0.94397533
C	-0.64627123	5.21157837	1.12988377
C	-1.50101829	6.25504446	1.85917974
H	-1.14962840	4.86322308	0.21582560
C	0.80809152	4.22640944	2.92253232
H	0.65661353	3.48780560	3.72366762
H	0.69540352	5.23462057	3.35449600
C	2.30614805	-4.17117310	-1.09843957
H	2.72472143	-3.34884262	-0.49290109
H	3.00893903	-4.39123201	-1.91753972
C	0.96742916	-3.74277544	-3.19301629
H	0.18245094	-3.04967666	-3.52923632
C	0.71556818	-5.15289640	-3.73583698
Sm	0.49274507	-0.26192358	0.95405149
N	-1.02215886	-1.53584051	3.10560751
O	0.25910753	-1.54565012	3.05813313
O	-1.60729086	-0.97308385	2.07671309

O	-1.66405892	-2.00245690	4.02763987
N	3.20089221	-0.32617331	2.01220202
O	2.33911514	0.60011464	2.30515814
O	2.75960970	-1.24039078	1.21646988
O	4.33543348	-0.32150328	2.45335937
N	1.61208129	0.90875077	-1.49088383
O	2.03727031	1.42904747	-2.50667119
O	1.52670622	-0.36986548	-1.34402919
O	1.20731175	1.61065483	-0.47375455
H	0.29955116	5.67550230	0.79726720
C	2.20157480	4.04095936	2.31184983
C	-1.83804405	7.44737577	0.95578039
H	-2.43318152	5.78919077	2.22862959
H	-0.96534687	6.61318779	2.75594211
C	-2.67772555	8.51075554	1.67025101
H	-0.89932275	7.90031338	0.58833629
H	-2.37755895	7.08729124	0.06057183
H	-2.90620017	9.35385990	0.99973780
H	-3.63462234	8.09134388	2.02245426
H	-2.14521527	8.91277599	2.54784679
C	3.29960942	4.16642714	3.37442780
H	2.25478840	3.03959441	1.85496914
H	2.36873174	4.78212404	1.50854588
C	4.69405031	3.88616657	2.80549669
H	3.27041698	5.17462301	3.82987285
H	3.09062076	3.44510674	4.18525839
H	5.46950388	4.00197458	3.57930946
H	4.75258303	2.85498762	2.42049026
H	4.93495750	4.57478094	1.97793531
C	2.09669518	-5.40932751	-0.22159705
H	1.91603422	-3.34659886	-3.59888411

C 0.64943403 -5.17196751 -5.26760054

H 1.51815307 -5.82955217 -3.39211106

H -0.22534719 -5.55217600 -3.31345034

C 0.41033190 -6.57600594 -5.83140516

H -0.15374503 -4.49285936 -5.60796595

H 1.59169102 -4.76345205 -5.67572832

H 0.36703849 -6.56085920 -6.93165159

H 1.21858585 -7.26487064 -5.53553438

H -0.53979868 -6.99649334 -5.46247196

C 3.40691495 -5.85220671 0.44043514

H 1.35706544 -5.16588402 0.55958527

H 1.68134451 -6.23491287 -0.82821345

C 3.22806454 -7.08923197 1.32575119

H 4.16534901 -6.05753088 -0.33815828

H 3.79905939 -5.01613283 1.04687822

H 4.18069458 -7.38596869 1.79231393

H 2.50484252 -6.89251232 2.13430500

H 2.85550761 -7.94792891 0.74208289

O -4.12074471 0.31657436 2.27313757

H -3.31158304 -0.24522540 2.30160046

H -4.35789967 0.39315882 3.21278024

(NO_3^- in outer coordination sphere)

E= -13806.969757, E0 = -13806.258589, G= 380.4480 at T=298.150 K

Sm -0.72473896 -0.51631635 -0.01198136

Cl 3.77511454 2.00777268 4.89407539

Cl 2.62359142 5.55577660 -1.72554147

O -1.12079799 0.43986368 -2.14511156

O -0.79961342 -1.34401929 2.34408474

O -3.14729524 -0.81765366 0.20016015

O -4.19090414 -2.69915938 -0.27859390

O -2.01551175 -2.51599646 -0.57910007

O	0.86503232	-2.41725373	0.32356551
O	0.99948728	-1.36632180	-1.58908165
O	2.34328175	-3.06394196	-1.17564356
O	-1.78282690	1.48964274	0.86369562
H	-1.63970041	2.49359870	0.59691936
H	-2.74856758	1.34257889	0.85138148
N	1.13007116	0.34016189	1.63334620
N	0.63945240	-2.59472084	3.57644320
N	0.74757403	1.57132471	-0.73189193
N	-0.79302353	1.62309849	-4.02437592
N	-3.16936421	-2.04468060	-0.22454704
N	1.44443583	-2.31823826	-0.83255249
C	1.25449455	-0.29134023	2.80063128
C	2.06465912	0.20822740	3.83576345
H	2.11614156	-0.28651384	4.80538988
C	2.77531743	1.37624705	3.61386395
C	2.68141961	2.06241298	2.37719703
C	3.35702801	3.28177524	2.07010746
H	4.02628517	3.71062303	2.81719470
C	3.15514135	3.91210508	0.87466305
H	3.65866995	4.85463142	0.65428865
C	2.26602507	3.37600803	-0.10512177
C	1.60041654	2.14407492	0.13809460
C	1.81216824	1.47759116	1.41086221
C	1.93424726	4.01121044	-1.32405138
C	1.03821528	3.43252492	-2.20447731
H	0.74066180	4.00040960	-3.07850742
C	0.47325698	2.17796922	-1.89393818
C	-0.53522921	1.38623214	-2.72198272
C	-0.20966570	2.66297030	-4.88135719
H	-0.18312384	2.24617791	-5.90227270

H	0.84329009	2.81793308	-4.60186386
C	-1.01106071	3.97390795	-4.85994101
H	-2.00506997	3.78939128	-5.30515432
H	-1.21004403	4.27683735	-3.81519628
C	-0.30571753	5.09853363	-5.62584496
H	-0.14033736	4.79262590	-6.67544222
H	0.69954091	5.26589870	-5.19337368
C	-1.09948373	6.40856981	-5.58201933
H	-2.08908391	6.28560877	-6.05211544
H	-0.57025105	7.21459723	-6.11432791
H	-1.26036191	6.72962475	-4.54033899
C	-1.82450938	0.77243757	-4.65626335
H	-2.27583170	1.36698329	-5.46706200
H	-2.60179067	0.56910479	-3.90527296
C	-1.24916387	-0.54062456	-5.19857454
H	-0.76065856	-1.08086669	-4.36954260
H	-0.46702990	-0.32094568	-5.94921207
C	-2.33523345	-1.42286301	-5.82442141
H	-3.10506558	-1.63879633	-5.06134653
H	-2.84425569	-0.86722726	-6.63423729
C	-1.77609348	-2.73973179	-6.37165165
H	-1.01843035	-2.55689502	-7.15204716
H	-2.57331491	-3.35886264	-6.81265163
H	-1.29785359	-3.32587290	-5.56973410
C	0.30727175	-1.47203994	2.90954208
C	2.00230718	-2.94569254	3.99858832
H	2.69726920	-2.18315816	3.61815476
H	2.26927066	-3.88440514	3.48035574
C	2.15950418	-3.11991262	5.51209116
H	1.86811328	-2.18573523	6.02744913
H	1.46105766	-3.89783835	5.86885977

C 3.59379125 -3.50263858 5.89640188

H 4.28973150 -2.72269654 5.53656864

H 3.87412357 -4.43252563 5.36918449

C 3.76964784 -3.69350219 7.40598679

H 3.52411509 -2.76965928 7.95537424

H 4.80717134 -3.96669531 7.65372276

H 3.11146259 -4.49301815 7.78394461

C -0.37007836 -3.67899108 3.57077193

H 0.16720521 -4.61347055 3.79776549

H -0.76486319 -3.75050807 2.54482222

C -1.50782907 -3.46068549 4.57425308

H -1.97430706 -2.48155355 4.37309980

H -1.08818412 -3.42397547 5.59506893

C -2.57906222 -4.56138563 4.49017954

H -2.10105538 -5.55147552 4.61526775

H -3.25902462 -4.43992996 5.35131121

C -3.40005851 -4.53847885 3.19493914

H -3.88166547 -3.55724597 3.04908919

H -4.19371557 -5.30186892 3.22114706

H -2.78546739 -4.73746681 2.30238461

O -1.28864670 3.93858194 0.31376323

O -1.14641070 5.33690071 -1.36974335

O -2.18863583 3.42542291 -1.63517368

N -1.55705309 4.24742746 -0.91913509

L2*Sm$(NO_3)_2^+$

E= -13450.014875, E0 = -13449.341979, G= 362.3886 at T=298.150 K

Cl -5.37558460 3.69190335 -0.41452506

Cl -4.11575794 -3.05974030 -3.70206261

N -1.57829750 1.32432818 0.18436578

N -0.24941191 4.05580807 2.07880855

N -1.12400532 -1.03690755 -0.99202359

N	1.20445371	-3.64922285	-1.76305568
O	0.01963539	1.81007040	2.18318987
O	0.84851617	-2.37762499	0.07793574
C	-1.75217891	2.50249696	0.79142833
C	-2.92926264	3.25366521	0.62521732
H	-3.10074592	4.17557764	1.17766130
C	-3.91644406	2.77747607	-0.22522369
C	-3.74065471	1.55232382	-0.91862047
C	-4.69501638	0.97702104	-1.81223118
H	-5.61960697	1.52181184	-2.00919485
C	-4.46784830	-0.23453224	-2.40436006
H	-5.20981073	-0.66629225	-3.07782769
C	-3.26674151	-0.96546936	-2.15279102
C	-2.97264957	-2.24777913	-2.68462992
C	-1.77182186	-2.87466574	-2.38687110
H	-1.58095455	-3.87496805	-2.77212954
C	-0.84971684	-2.21873331	-1.55132067
C	-2.28264809	-0.41747627	-1.28544593
C	-2.52606249	0.86281443	-0.65296078
C	-0.60617310	2.81060410	1.73517537
C	0.46847755	-2.77842283	-1.05682480
C	-0.74766976	5.30285692	1.46937799
C	-1.68648410	6.10784864	2.37518024
H	-1.22369993	5.06588078	0.50665301
C	0.88511938	4.20363665	3.02407002
H	0.80531448	3.40054679	3.77105570
H	0.74419218	5.16779375	3.53744984
C	2.42499495	-4.18874025	-1.10982430
H	2.94724178	-3.34887815	-0.62357670
H	3.06776857	-4.57143688	-1.91723573
C	1.02035499	-3.96668386	-3.19222713

H	0.30989611	-3.24887228	-3.62624264
C	0.58967894	-5.41052580	-3.46724725
Sm	0.27463597	-0.27105156	1.05015969
N	-1.40277708	-1.58705425	2.96590853
O	-0.11405826	-1.40045142	3.09950662
O	-1.89695203	-1.11847544	1.86053801
O	-2.06583452	-2.14751458	3.80225396
N	2.47849631	0.88225025	-0.39002195
O	3.43264365	1.35964191	-0.95617968
O	2.56150746	0.37469792	0.81366384
O	1.29298615	0.83030492	-0.91316819
H	0.14336136	5.90782356	1.22846949
C	2.25087762	4.15480375	2.32970238
C	-2.16453648	7.39603138	1.69246495
H	-2.55563664	5.49071455	2.66981483
H	-1.16659403	6.36048365	3.31582427
C	-3.09094882	8.22452354	2.58751512
H	-1.28583455	8.00123978	1.40553546
H	-2.68510985	7.14220190	0.75056213
H	-3.41832113	9.14059734	2.07287335
H	-3.99216199	7.65471888	2.86708474
H	-2.58113837	8.52500153	3.51711297
C	3.39855671	4.36052799	3.32643509
H	2.36753178	3.17608166	1.83313119
H	2.30017114	4.92920542	1.54224694
C	4.77140760	4.30468178	2.64978123
H	3.27258873	5.33142185	3.84001446
H	3.34048462	3.58208156	4.10884666
H	5.57802725	4.44992161	3.38473725
H	4.93170166	3.32993293	2.16016340
H	4.86989594	5.08878136	1.88107264

C 2.13366532 -5.29099751 -0.08552446

H 1.99036109 -3.76886439 -3.68149567

C 0.41020930 -5.66919899 -4.96894026

H 1.34572768 -6.10499811 -3.06111383

H -0.35034549 -5.63490868 -2.92924666

C -0.00312594 -7.11301804 -5.26940441

H -0.34668759 -4.97259045 -5.37438488

H 1.35525751 -5.43660641 -5.49200344

H -0.12558623 -7.26998472 -6.35177469

H 0.75601131 -7.82649040 -4.91023302

H -0.95926857 -7.36495209 -4.78225994

C 3.42622781 -5.80284262 0.56337458

H 1.46241832 -4.89245796 0.69383276

H 1.60568357 -6.12621641 -0.57880658

C 3.16394353 -6.90845442 1.59026849

H 4.10993814 -6.17703724 -0.22067150

H 3.94424152 -4.95805740 1.05269468

H 4.10514069 -7.25833178 2.04133177

H 2.51459932 -6.54746866 2.40468597

H 2.67022204 -7.77714109 1.12450516

L2*Eu(NO_3)$_3$(H_2O)

(H_2O in outer coordination sphere)

E= -14224.178487, E0 = -14223.467573, G= 379.8670 at T=298.150 K

Cl -5.43425083 3.65385890 -0.33941340

Cl -4.30498266 -3.12228608 -3.62323332

N -1.61732674 1.27836430 0.15889169

N -0.25993583 4.04981852 1.97717965

N -1.22230959 -1.10758781 -0.99251366

N 1.05192685 -3.74138117 -1.67294550

O -0.00950104 1.80341041 2.17894721

O 0.47180277 -2.72405505 0.27678701

C	-1.77319002	2.45622873	0.75971949
C	-2.95393968	3.21062398	0.62691408
H	-3.10373998	4.13379431	1.18382871
C	-3.96209145	2.73289466	-0.18940873
C	-3.81101418	1.50759637	-0.88072908
C	-4.79510021	0.92875046	-1.73723984
H	-5.72404623	1.47596335	-1.90429735
C	-4.59621906	-0.29065192	-2.32178926
H	-5.36296368	-0.72808707	-2.96296763
C	-3.39469695	-1.02884114	-2.09662342
C	-3.12831068	-2.32014728	-2.61670208
C	-1.94418991	-2.97241759	-2.31892872
H	-1.76451373	-3.98148799	-2.69023871
C	-1.00400376	-2.32002616	-1.49994957
C	-2.37610030	-0.47445288	-1.26955318
C	-2.58934450	0.81735975	-0.64631808
C	-0.61387128	2.77729392	1.68510747
C	0.24708495	-2.95841336	-0.92565858
C	-0.68562186	5.24663830	1.23863459
C	-1.57208014	6.20241737	2.04649806
H	-1.18767023	4.93361855	0.31147343
C	0.86145985	4.22902536	2.92622781
H	0.77443165	3.43750930	3.68506265
H	0.71261126	5.20230103	3.42270064
C	2.24882102	-4.28336382	-0.98967904
H	2.66974592	-3.47676873	-0.36478770
H	2.97958708	-4.52847815	-1.77662504
C	1.02934790	-3.78797436	-3.14028215
H	0.27792305	-3.07160687	-3.50387716
C	0.76859272	-5.18375015	-3.71431136
Eu	0.51047897	-0.23910268	0.83988804

N	-1.01825309	-1.27636814	3.11557007
O	0.26107064	-1.18519497	3.11090398
O	-1.59892356	-0.98605347	1.98230124
O	-1.66122043	-1.60226548	4.09790516
N	3.32743692	-0.19337045	1.59841740
O	2.64300990	0.88146275	1.35620487
O	2.67236376	-1.29357457	1.48158288
O	4.50498009	-0.15371591	1.91170490
N	1.62922895	0.59414595	-1.75012529
O	2.08850312	0.95961499	-2.81939816
O	1.79157424	-0.60536188	-1.29840946
O	0.93390375	1.38203847	-0.99533677
H	0.23208058	5.77423716	0.92314631
C	2.23904252	4.15648508	2.25884318
C	-1.98764145	7.42721176	1.22273910
H	-2.47117233	5.67077303	2.40894079
H	-1.03208840	6.53332233	2.95108604
C	-2.85720110	8.40592003	2.01790357
H	-1.08104026	7.94525099	0.86068141
H	-2.53299713	7.09377098	0.32073388
H	-3.14241958	9.27400589	1.40338373
H	-3.78380132	7.92123032	2.36722517
H	-2.32128668	8.78225040	2.90475059
C	3.37103605	4.28941774	3.28476310
H	2.33555150	3.18666172	1.74448514
H	2.33449006	4.94969606	1.49426055
C	4.75271702	4.12134409	2.64409590
H	3.30456519	5.26745510	3.79793906
H	3.23706722	3.51568532	4.06298494
H	5.55363464	4.22435093	3.39330649
H	4.84401035	3.12527514	2.18124223

H 4.92332983 4.87811947 1.85980439

C 1.95425797 -5.51298189 -0.12563512

H 2.00516510 -3.40844893 -3.49454308

C 0.77584457 -5.18150806 -5.24761486

H 1.53630877 -5.88559723 -3.34237385

H -0.20159809 -5.56358242 -3.34330153

C 0.53105277 -6.57188654 -5.84201288

H 0.00604632 -4.47888756 -5.61617184

H 1.74590528 -4.79049873 -5.60449266

H 0.54064882 -6.54137230 -6.94276524

H 1.30792916 -7.28383493 -5.51801014

H -0.44520193 -6.97418547 -5.52450323

C 3.21648955 -6.00978422 0.58907676

H 1.19236398 -5.24117374 0.62428200

H 1.53171003 -6.32012033 -0.75180650

C 2.95034933 -7.23771667 1.46516037

H 3.99654579 -6.24722528 -0.15832597

H 3.61875033 -5.19067287 1.21186745

H 3.87028360 -7.57468319 1.96859765

H 2.20462298 -7.01024723 2.24467874

H 2.56492352 -8.08000088 0.86613894

O -4.11918402 0.29136381 2.19003558

H -3.30824804 -0.26904193 2.19156456

H -4.37931633 0.28167996 3.12673187

(NO_3^- in outer coordination sphere)

E= -14224.169022, E0 = -14223.457659, G= 380.5914 at T=298.150 K

Eu -0.74868727 -0.50300997 0.00678426

Cl 3.70211816 2.04364014 4.96113253

Cl 2.60398030 5.60055494 -1.66543603

O -1.20544827 0.55410135 -2.08579659

O -0.84507161 -1.32610404 2.37637067

O	-3.16758204	-0.82332003	0.04163977
O	-4.15013075	-2.79725242	0.04540302
O	-1.96353412	-2.63614440	-0.16712113
O	1.11898744	-2.14890575	0.26825014
O	0.51875365	-1.63877904	-1.77221978
O	2.05938458	-3.17801118	-1.43694580
O	-1.87639499	1.51848853	0.90394354
H	-1.73697054	2.48990250	0.62642723
H	-2.83205199	1.34483695	0.79861897
N	1.05869436	0.39737079	1.68606496
N	0.63213682	-2.58428454	3.55594373
N	0.73733550	1.59988284	-0.70050460
N	-0.77818078	1.65596342	-3.99746156
N	-3.14155436	-2.12179375	-0.02434306
N	1.26816547	-2.36191845	-1.00121593
C	1.19246483	-0.24759649	2.84427071
C	2.00437260	0.24233763	3.88289261
H	2.05977798	-0.26457685	4.84612846
C	2.70960212	1.41556251	3.67300558
C	2.62042522	2.10645437	2.43854237
C	3.29671597	3.32738376	2.13871551
H	3.95979309	3.75547981	2.89183974
C	3.10200548	3.96110845	0.94401217
H	3.60372066	4.90620565	0.73075646
C	2.22641420	3.42211819	-0.04679435
C	1.56630599	2.18462133	0.18637417
C	1.75535738	1.52525675	1.46646929
C	1.90974402	4.05649662	-1.27017927
C	1.02151513	3.47724080	-2.15862012
H	0.72816539	4.04423332	-3.03533769
C	0.46218646	2.21915221	-1.85649395

C	-0.56348681	1.44615078	-2.68142176
C	-0.13852751	2.65116858	-4.86517000
H	-0.06752800	2.19532394	-5.86726952
H	0.90114480	2.81618333	-4.54372978
C	-0.92375129	3.97101831	-4.93506813
H	-1.90312755	3.77864480	-5.40819073
H	-1.15619731	4.32394505	-3.91315651
C	-0.17492193	5.05129766	-5.72332907
H	0.03300718	4.68973970	-6.74733162
H	0.81199211	5.23156071	-5.25551653
C	-0.95587146	6.36836863	-5.78295898
H	-1.92723179	6.22852755	-6.28518867
H	-0.39715880	7.13927174	-6.33662701
H	-1.15479422	6.74957180	-4.76841593
C	-1.81213582	0.81293106	-4.63610268
H	-2.22270298	1.39201641	-5.47913933
H	-2.61641693	0.65261167	-3.90333414
C	-1.25268614	-0.53083444	-5.11706543
H	-0.79881704	-1.05448198	-4.25800467
H	-0.44612405	-0.35462692	-5.85324669
C	-2.34203935	-1.40818501	-5.74413729
H	-3.13618088	-1.58275282	-4.99544859
H	-2.81760049	-0.86831397	-6.58450317
C	-1.79886723	-2.75409675	-6.23395777
H	-1.01719594	-2.61346507	-6.99934101
H	-2.59831166	-3.36959982	-6.67604399
H	-1.35444736	-3.32375908	-5.40124416
C	0.26645535	-1.44659078	2.93158054
C	2.00603867	-2.91676664	3.95628023
H	2.68023729	-2.12653732	3.59573817
H	2.29080319	-3.83172441	3.40601850

C 2.17963552 -3.13644123 5.46212196

H 1.87314379 -2.22567534 6.00965214

H 1.50077105 -3.94019175 5.79877329

C 3.62466574 -3.50103784 5.82291126

H 4.30122805 -2.69519663 5.48380899

H 3.91998959 -4.40698528 5.26318645

C 3.81682038 -3.73785853 7.32399511

H 3.55681348 -2.83794308 7.90552425

H 4.86167669 -3.99756289 7.55495977

H 3.17815113 -4.56284571 7.68010664

C -0.35345727 -3.69019318 3.52310395

H 0.20896699 -4.62116432 3.69754601

H -0.77156663 -3.72503352 2.50426984

C -1.47263706 -3.54200315 4.56007004

H -1.96712136 -2.56721735 4.41023827

H -1.03043032 -3.53652048 5.57195663

C -2.51821160 -4.66535807 4.45281744

H -2.00978279 -5.64683628 4.50565720

H -3.16900492 -4.61104012 5.34293747

C -3.38548565 -4.59311771 3.19029427

H -3.91101766 -3.62594461 3.12710929

H -4.14720774 -5.38897371 3.19240999

H -2.79839802 -4.70418262 2.26497102

O -1.33334625 4.03357601 0.29363540

O -1.17118502 5.44980717 -1.36905253

O -2.26668596 3.57832885 -1.64575291

N -1.60239673 4.36432076 -0.91704226

L2*Eu(NO_3)$_2^+$

E= -13867.212865, E0 = -13866.540227, G= 362.3709 at T=298.150 K

Cl -5.39401102 3.67122936 -0.37240425

Cl -4.13812637 -3.08682418 -3.64184403

N	-1.58289623	1.32154453	0.21060356
N	-0.26063773	4.06977606	2.08740425
N	-1.13151562	-1.04744995	-0.96218306
N	1.19436705	-3.65622830	-1.74417996
O	0.00540664	1.82342899	2.22714138
O	0.83594263	-2.41224670	0.11827615
C	-1.75967622	2.49989605	0.81751209
C	-2.94086504	3.24501157	0.65546453
H	-3.11217952	4.16772318	1.20687866
C	-3.93027329	2.76295137	-0.18884823
C	-3.75347710	1.53658974	-0.88019431
C	-4.71189594	0.95600098	-1.76604486
H	-5.63899899	1.49796295	-1.95887113
C	-4.48580217	-0.25710249	-2.35521984
H	-5.23106050	-0.69314080	-3.02228189
C	-3.28167486	-0.98434043	-2.10796547
C	-2.98911047	-2.26928186	-2.63498688
C	-1.78656995	-2.89393854	-2.34037900
H	-1.59580016	-3.89606023	-2.72148013
C	-0.86012441	-2.23267055	-1.51354301
C	-2.29227829	-0.43030635	-1.25049198
C	-2.53417230	0.85263634	-0.62056649
C	-0.61394298	2.81616497	1.76311982
C	0.45983613	-2.79622364	-1.02113211
C	-0.75008714	5.30520058	1.44865084
C	-1.68341982	6.13915586	2.33383536
H	-1.22928488	5.04892540	0.49237147
C	0.86888200	4.23193884	3.03539562
H	0.78507084	3.43940473	3.79315042
H	0.72664201	5.20367289	3.53400397
C	2.41514206	-4.20715427	-1.10267758

H	2.93684959	-3.37722898	-0.59906983
H	3.05891228	-4.57267380	-1.91729259
C	1.01343572	-3.94085884	-3.18055344
H	0.29330054	-3.22198439	-3.59650826
C	0.59942561	-5.38245249	-3.49052811
Eu	0.27445623	-0.26811689	1.06047606
N	-1.34163260	-1.59399855	3.01919317
O	-0.08826111	-1.27097690	3.18493295
O	-1.83429646	-1.24979258	1.86968601
O	-1.98144829	-2.16478872	3.86803150
N	2.50317669	0.81033301	-0.39626065
O	3.47195721	1.24215817	-0.97474283
O	2.53470302	0.48104918	0.86791986
O	1.35323763	0.63674676	-0.96670121
H	0.14418937	5.89907122	1.19207537
C	2.23854446	4.17094994	2.34952307
C	-2.14916897	7.41513634	1.62048531
H	-2.55862927	5.53674364	2.64078188
H	-1.16325784	6.40919113	3.26943374
C	-3.07331538	8.27062798	2.49215031
H	-1.26471829	8.00715923	1.32380641
H	-2.66759253	7.14393234	0.68224061
H	-3.39003611	9.17820549	1.95625043
H	-3.98092961	7.71452951	2.77854562
H	-2.56609845	8.58671570	3.41799092
C	3.38072968	4.38817787	3.35012412
H	2.35546207	3.18506694	1.86729467
H	2.29416704	4.93524933	1.55248046
C	4.75772381	4.32313681	2.68278599
H	3.25269961	5.36542082	3.85099292
H	3.31733418	3.61952734	4.14170408

H	5.56012440	4.47695923	3.42063808
H	4.92024899	3.34227991	2.20628452
H	4.86154795	5.09766006	1.90506721
C	2.12570786	-5.33023071	-0.10079075
H	1.98017824	-3.71958041	-3.66656828
C	0.42449290	-5.60755301	-4.99811172
H	1.36263275	-6.07810974	-3.10008788
H	-0.33881199	-5.62991619	-2.95950341
C	0.02806728	-7.04840994	-5.33354807
H	-0.33989602	-4.91015530	-5.38776636
H	1.36739028	-5.35178423	-5.51422453
H	-0.09094554	-7.18116665	-6.41954899
H	0.79470819	-7.76155996	-4.98996162
H	-0.92588216	-7.32265091	-4.85419989
C	3.41880417	-5.85317564	0.53799343
H	1.45341277	-4.94834042	0.68599731
H	1.59905028	-6.15613317	-0.61085075
C	3.15894699	-6.97961044	1.54267645
H	4.10294628	-6.21066427	-0.25342461
H	3.93596625	-5.01764345	1.04388356
H	4.10105562	-7.33640146	1.98643529
H	2.50890899	-6.63650417	2.36422563
H	2.66701794	-7.83981276	1.05962348

L2*Gd($(NO_3)_3(H_2O)$)

(H_2O in outer coordination sphere)

E= -14651.737895, E0 = -14651.025965, G= 380.9346 at T=298.150 K

Cl	-5.44965744	3.67270160	-0.37655744
Cl	-4.28738117	-3.08894420	-3.70251942
N	-1.63883138	1.28706849	0.12192142
N	-0.26120105	4.03263855	1.95560360
N	-1.23504496	-1.08004749	-1.03017259

N	1.06794131	-3.68996096	-1.70434475
O	-0.02645199	1.78550231	2.12615848
O	0.46850538	-2.65720725	0.22684632
C	-1.79207182	2.46301317	0.72545868
C	-2.97084355	3.22115850	0.59358341
H	-3.12024331	4.14405441	1.15130901
C	-3.97959042	2.74797344	-0.22619653
C	-3.82961965	1.52539623	-0.92215574
C	-4.80855846	0.94820797	-1.78581750
H	-5.73855925	1.49326789	-1.95450425
C	-4.60289812	-0.26720646	-2.37723303
H	-5.36516094	-0.70247376	-3.02524567
C	-3.39991617	-1.00361311	-2.15208435
C	-3.12217903	-2.28766370	-2.68268824
C	-1.93394852	-2.93360949	-2.38541341
H	-1.74677408	-3.93766665	-2.76606631
C	-1.00424707	-2.28351974	-1.55292141
C	-2.38955569	-0.45289552	-1.31303406
C	-2.60963750	0.83448952	-0.68518275
C	-0.62786329	2.76958013	1.64819443
C	0.24890026	-2.90905666	-0.97504336
C	-0.69163322	5.24440384	1.24570179
C	-1.57532024	6.18040800	2.07926130
H	-1.19768965	4.95162249	0.31403729
C	0.87789369	4.18582821	2.88887644
H	0.78960109	3.39039469	3.64343047
H	0.75400198	5.15855455	3.39321327
C	2.26915622	-4.20786476	-1.00673735
H	2.67743158	-3.38626099	-0.39289489
H	3.00419569	-4.45784521	-1.78807819
C	1.03188455	-3.79379201	-3.16838336

H	0.28529587	-3.08385015	-3.55366635
C	0.75424856	-5.20858574	-3.68588805
Gd	0.52517688	-0.24336459	0.84546238
N	-0.95543337	-1.18597174	3.14225984
O	0.31545028	-1.02986169	3.16152048
O	-1.51692045	-0.98079103	1.97094488
O	-1.61558902	-1.49248767	4.11666441
N	3.26443172	-0.37366119	1.72118723
O	2.57322621	0.73008198	1.65652132
O	2.61402869	-1.43201363	1.37146878
O	4.42621183	-0.39199018	2.08008695
N	1.63400114	0.82958025	-1.60499561
O	2.09099650	1.26409149	-2.64529157
O	1.71244037	-0.42895293	-1.28270984
O	1.02870762	1.57578230	-0.73996574
H	0.22464626	5.77871895	0.93795604
C	2.24243927	4.09135294	2.19760513
C	-1.99252808	7.42482042	1.28620195
H	-2.47384691	5.63987350	2.43000174
H	-1.03310657	6.48779869	2.99079633
C	-2.87578011	8.37582397	2.09950352
H	-1.08649242	7.95861912	0.94635171
H	-2.52646875	7.11167717	0.37024528
H	-3.16068220	9.25863838	1.50616634
H	-3.80243683	7.87609053	2.42680383
H	-2.35052347	8.73106956	3.00128984
C	3.39123464	4.17367601	3.20977712
H	2.30466008	3.12873054	1.66508496
H	2.34609294	4.89627886	1.44644022
C	4.75930309	3.98081708	2.54779887
H	3.35795426	5.14292431	3.74277329

H 3.24663281 3.38683772 3.97236085

H 5.57230234 4.05346203 3.28756332

H 4.81821203 2.98772883 2.07365489

H 4.93872786 4.74301577 1.77063096

C 1.98631489 -5.42535734 -0.12170695

H 2.00917029 -3.43963194 -3.54341793

C 0.74001330 -5.26316738 -5.21819544

H 1.52292335 -5.90061188 -3.29831982

H -0.21273071 -5.56800270 -3.28714919

C 0.47670639 -6.67274523 -5.75679350

H -0.02952669 -4.56857395 -5.60224915

H 1.70784652 -4.89346075 -5.60274172

H 0.47109628 -6.68315697 -6.85791779

H 1.25282574 -7.37815619 -5.41717863

H -0.49790296 -7.05526114 -5.41111088

C 3.25141144 -5.88304615 0.61405385

H 1.21427476 -5.15110683 0.61682379

H 1.58312666 -6.25303555 -0.73367614

C 3.00130844 -7.09888554 1.51131487

H 4.04311895 -6.11941242 -0.12134465

H 3.63020205 -5.04334831 1.22376585

H 3.92286181 -7.40608788 2.03058815

H 2.24321270 -6.87179470 2.27892065

H 2.64003849 -7.96096039 0.92553711

O -4.10777473 0.16411616 2.06766009

H -3.26576185 -0.34693867 2.10677981

H -4.40400219 0.14603145 2.99343109

(NO_3^- in outer coordination sphere)

E= -14651.731090, E0 = -14651.018903, G= 382.3491 at T=298.150 K

Gd -0.60501808 -0.52865732 -0.06172679

Cl 3.79774427 1.99848509 4.86209393

Cl	2.69609261	5.50533867	-1.78964078
O	-1.10901296	0.43135181	-2.13508964
O	-0.74037468	-1.39024556	2.24607706
O	-2.96417499	-0.94422203	0.10147517
O	-3.91718531	-2.83889651	-0.50734454
O	-1.74666572	-2.54068089	-0.76455671
O	1.05818117	-2.31835413	0.29023191
O	1.01724339	-1.36792159	-1.67713034
O	2.42067623	-3.02543783	-1.29208159
O	-1.65848303	1.40101051	0.85969675
H	-1.50896347	2.42649817	0.61033416
H	-2.62401557	1.25439453	0.83735001
N	1.17099798	0.32075241	1.59446537
N	0.64896899	-2.59309769	3.57644987
N	0.80388206	1.53435755	-0.77486050
N	-0.82929015	1.61750340	-4.02031088
N	-2.92710686	-2.14654922	-0.39901671
N	1.54235685	-2.27556086	-0.91370541
C	1.28233945	-0.30492482	2.76655054
C	2.08424258	0.20206094	3.80435491
H	2.11977887	-0.27935633	4.78111410
C	2.80761218	1.36155403	3.57764578
C	2.73370624	2.03488326	2.33296037
C	3.42100787	3.24542975	2.01795292
H	4.09138060	3.67509484	2.76352477
C	3.22459149	3.86943579	0.81789404
H	3.73364782	4.80787134	0.59281170
C	2.33091760	3.33524060	-0.15860768
C	1.66262269	2.10701656	0.08976050
C	1.86587465	1.44908547	1.36649406
C	1.99309123	3.97058892	-1.37614238

C	1.07774222	3.40121388	-2.24273443
H	0.77183479	3.97289753	-3.11126804
C	0.50648409	2.15111089	-1.92689466
C	-0.53382576	1.37458813	-2.72826791
C	-0.27153537	2.66527486	-4.88478708
H	-0.28799015	2.26351881	-5.91178799
H	0.79279512	2.80819225	-4.64447689
C	-1.06242633	3.98082232	-4.80912638
H	-2.07000470	3.81326866	-5.22989893
H	-1.22959721	4.25617552	-3.75102687
C	-0.37227041	5.12067842	-5.56624985
H	-0.23792022	4.84129667	-6.62770939
H	0.64607692	5.27159500	-5.15873957
C	-1.15627694	6.43344545	-5.46401310
H	-2.16068769	6.32802486	-5.90612650
H	-0.63892692	7.25044489	-5.99126768
H	-1.28272903	6.72726107	-4.40961361
C	-1.88868058	0.77916968	-4.62213564
H	-2.35941935	1.38116431	-5.41621590
H	-2.64348793	0.58042413	-3.84729457
C	-1.34355152	-0.53745002	-5.18646193
H	-0.83503580	-1.08565784	-4.37488461
H	-0.58327615	-0.32283843	-5.96063995
C	-2.45776653	-1.40629637	-5.78075886
H	-3.20411229	-1.61949015	-4.99403954
H	-2.98766232	-0.84161574	-6.57072687
C	-1.92953241	-2.72543263	-6.35271168
H	-1.19492459	-2.54594278	-7.15567541
H	-2.74672246	-3.33375144	-6.77162457
H	-1.43249488	-3.32129216	-5.56953335
C	0.34041145	-1.49046659	2.86535931

C	1.99356496	-2.93067455	4.06395102
H	2.70606613	-2.18545032	3.68221736
H	2.27610922	-3.88886523	3.59180021
C	2.09085250	-3.05079746	5.58774757
H	1.78403258	-2.09800148	6.05811834
H	1.37471366	-3.81262827	5.94418240
C	3.50696802	-3.42508197	6.04146719
H	4.22053051	-2.66072440	5.68270874
H	3.80313587	-4.37379646	5.55829811
C	3.62217498	-3.56392503	7.56253576
H	3.35988021	-2.62063074	8.06944752
H	4.64772367	-3.83215880	7.86039114
H	2.94555211	-4.34735537	7.94159222
C	-0.35805821	-3.68007302	3.56183267
H	0.16902991	-4.60414696	3.84741783
H	-0.70511085	-3.78930998	2.52200150
C	-1.54154122	-3.42912197	4.50290442
H	-2.00175071	-2.46117806	4.24263477
H	-1.16861689	-3.35076737	5.53936768
C	-2.60444307	-4.53741932	4.41391993
H	-2.13216066	-5.51875925	4.60970783
H	-3.32833195	-4.37862778	5.23205948
C	-3.35700011	-4.57798147	3.07814574
H	-3.82372952	-3.60380602	2.85634398
H	-4.15580130	-5.33601046	3.10207772
H	-2.69797468	-4.82583523	2.23066545
O	-1.15309870	3.83020616	0.37589321
O	-1.04146838	5.29880619	-1.25305879
O	-2.08805799	3.39673185	-1.58044147
N	-1.44434249	4.19299889	-0.84362113

L2*Gd(NO$_3$)$_2^+$

E= -14294.772616, E0 = -14294.099230, G= 362.6637 at T=298.150 K

Cl	-5.38440180	3.71400166	-0.46554002
Cl	-4.13414526	-3.04007626	-3.75639248
N	-1.58450902	1.34853840	0.11180023
N	-0.26371500	4.06082439	2.04700470
N	-1.13224590	-1.00790298	-1.06692553
N	1.18611884	-3.64182687	-1.80259216
O	0.02326530	1.81566310	2.09767866
O	0.83049446	-2.33923101	0.01630012
C	-1.75767338	2.52305984	0.72810566
C	-2.93603683	3.27269316	0.56649941
H	-3.10795474	4.19141912	1.12395811
C	-3.92545390	2.79985309	-0.28388843
C	-3.75205159	1.57665765	-0.98205882
C	-4.70953035	1.00066531	-1.87203670
H	-5.63555336	1.54442453	-2.06504583
C	-4.48401785	-0.21127817	-2.46445036
H	-5.22875881	-0.64430618	-3.13400435
C	-3.28132486	-0.94135594	-2.21737885
C	-2.98850036	-2.22585559	-2.74560237
C	-1.78767908	-2.85324669	-2.44680882
H	-1.59982312	-3.85611272	-2.82682490
C	-0.86171865	-2.19598579	-1.61723566
C	-2.29365206	-0.39041305	-1.35724664
C	-2.53577399	0.88894659	-0.72370517
C	-0.60982549	2.82094383	1.67533016
C	0.45387933	-2.75623798	-1.11191523
C	-0.77239019	5.31813717	1.46847701
C	-1.72220349	6.08896494	2.39252710
H	-1.24212027	5.10268307	0.49755850
C	0.87118703	4.19393158	2.99477077

H	0.80285263	3.36647487	3.71568727
H	0.72046906	5.14046669	3.53732228
C	2.40317416	-4.17518568	-1.13642335
H	2.92732549	-3.32876945	-0.66398799
H	3.04552937	-4.57586145	-1.93538237
C	1.00129473	-3.98497009	-3.22541523
H	0.29975539	-3.26803660	-3.67542505
C	0.55508447	-5.42931128	-3.47223735
Gd	0.24122663	-0.24015240	0.95070964
N	-1.08721924	-1.57350564	3.03735232
O	0.19295418	-1.32185495	3.04826331
O	-1.71078181	-1.14793146	1.96189249
O	-1.65315676	-2.14622331	3.93183756
N	2.68032026	0.75869852	-0.05132899
O	3.71858954	1.20516574	-0.47340071
O	2.57753515	0.17727780	1.11013925
O	1.55701447	0.82502657	-0.72645104
H	0.11328476	5.93801069	1.24704993
C	2.23452950	4.18015099	2.29446697
C	-2.21278453	7.38970423	1.74337411
H	-2.58516383	5.45486212	2.66904736
H	-1.20696247	6.32181120	3.34074140
C	-3.15016055	8.18329620	2.65841889
H	-1.34019578	8.01197910	1.47502339
H	-2.72854209	7.15585613	0.79360503
H	-3.48697567	9.10934448	2.16839767
H	-4.04534674	7.59600639	2.92066097
H	-2.64541745	8.46400547	3.59689307
C	3.38695431	4.33545685	3.29466224
H	2.34825230	3.22621536	1.75213242
H	2.28123999	4.99022961	1.54366994

C 4.75572395 4.30276299 2.60813832

H 3.26806784 5.28246260 3.85251260

H 3.32818484 3.52139139 4.03964281

H 5.56714725 4.40934610 3.34432554

H 4.90703392 3.35036969 2.07386112

H 4.85494947 5.12096310 1.87588382

C 2.10471916 -5.25611877 -0.09161983

H 1.97414494 -3.80760527 -3.71662092

C 0.37704706 -5.71717644 -4.96874952

H 1.30174136 -6.12350368 -3.04879212

H -0.38897336 -5.63194609 -2.93255711

C -0.06035202 -7.15968275 -5.24008036

H -0.36603710 -5.01638174 -5.39233208

H 1.32786405 -5.51224756 -5.49283123

H -0.17866667 -7.33832264 -6.31953144

H 0.68352258 -7.87841892 -4.86014509

H -1.02394485 -7.38352680 -4.75393009

C 3.39463639 -5.75788927 0.57049757

H 1.43378866 -4.83951616 0.67834759

H 1.57550657 -6.09950256 -0.56953430

C 3.12707376 -6.84187794 1.61887681

H 4.07931805 -6.14940691 -0.20419598

H 3.91259170 -4.90443325 1.04426777

H 4.06677151 -7.18442869 2.07863045

H 2.47735381 -6.46239567 2.42444205

H 2.63222146 -7.71873140 1.16984093

L2*Tb(NO_3)$_3$(H_2O)

(H_2O in outer coordination sphere)

E= -15089.681394, E0 = -15088.969479, G= 381.1737 at T=298.150 K

Cl -5.41335154 3.69257998 -0.43099645

Cl -4.24714279 -3.07050657 -3.76040697

N	-1.60499394	1.29868269	0.07526462
N	-0.25641274	4.02858400	1.96857285
N	-1.19890928	-1.05727434	-1.08782935
N	1.08408141	-3.70465994	-1.72803903
O	0.01634821	1.78119636	2.05539989
O	0.52066606	-2.59254026	0.16861753
C	-1.76082277	2.47561002	0.68480688
C	-2.93881249	3.23345017	0.54884171
H	-3.09206557	4.15247440	1.11142397
C	-3.94416547	2.76611376	-0.27805752
C	-3.79230762	1.54513383	-0.97851497
C	-4.76712990	0.97189015	-1.84787762
H	-5.69576979	1.51880515	-2.01815987
C	-4.56215334	-0.24374048	-2.44252443
H	-5.32353783	-0.67610592	-3.09339380
C	-3.36220121	-0.98108661	-2.21451473
C	-3.08338785	-2.26682091	-2.74056649
C	-1.89789653	-2.91448474	-2.43689084
H	-1.71518004	-3.92182088	-2.81041217
C	-0.96727020	-2.26315045	-1.60586178
C	-2.35319638	-0.43076593	-1.37183487
C	-2.57385874	0.85252547	-0.73845434
C	-0.60488379	2.77264667	1.61206245
C	0.28341603	-2.88344431	-1.02154076
C	-0.69896525	5.26038933	1.30208147
C	-1.60712647	6.14776993	2.16229558
H	-1.18854976	4.99645615	0.35315454
C	0.87341189	4.16405869	2.91521025
H	0.79433399	3.33860683	3.63784409
H	0.72899324	5.11398840	3.45638990
C	2.27436662	-4.22922945	-1.01698077

H	2.70949697	-3.39753318	-0.43641591
H	2.99716735	-4.53075838	-1.79153645
C	1.04015362	-3.85890508	-3.18740988
H	0.31236044	-3.14249325	-3.59583139
C	0.72389936	-5.28295374	-3.65528059
Tb	0.54197568	-0.18736294	0.74093294
N	-0.87306422	-1.18508756	3.03285599
O	0.39783359	-1.01109254	3.01735616
O	-1.46721423	-0.95794022	1.88533676
O	-1.49729967	-1.52413571	4.01946497
N	3.29161000	-0.28543755	1.55443525
O	2.57967663	0.80677694	1.47118223
O	2.65107417	-1.35769820	1.24594295
O	4.45898199	-0.27196860	1.89378905
N	1.62176812	0.88613260	-1.70213437
O	2.08709693	1.31065714	-2.74186945
O	1.71319675	-0.36907935	-1.36059916
O	0.99544758	1.63079047	-0.85467041
H	0.21163458	5.82202435	1.02783954
C	2.24441218	4.12147617	2.23162556
C	-2.03847671	7.41597509	1.41610599
H	-2.49915218	5.57874489	2.48311567
H	-1.07833445	6.42784214	3.09044838
C	-2.93177080	8.32543945	2.26539850
H	-1.13896179	7.97255278	1.09613776
H	-2.57096887	7.13234329	0.48965341
H	-3.22831869	9.22639465	1.70600402
H	-3.85182023	7.80257225	2.57467628
H	-2.40946722	8.65285301	3.17936993
C	3.38472986	4.19833660	3.25385857
H	2.33147931	3.17738914	1.67010748

H	2.33499837	4.95194960	1.50705218
C	4.76098061	4.05760479	2.59553719
H	3.32602811	5.14958286	3.81618762
H	3.25361705	3.38578820	3.99163365
H	5.56770897	4.12464857	3.34266710
H	4.84598207	3.08251667	2.08902049
H	4.92768049	4.84868193	1.84488785
C	1.95986581	-5.40410519	-0.08609218
H	2.02577257	-3.54549074	-3.57675767
C	0.70765644	-5.39089727	-5.18472528
H	1.47367096	-5.98152971	-3.24311233
H	-0.25212836	-5.60236835	-3.24475503
C	0.40577304	-6.81082678	-5.67362976
H	-0.04274003	-4.68945313	-5.59342575
H	1.68513477	-5.06151485	-5.58134747
H	0.39978936	-6.85978270	-6.77370310
H	1.16229403	-7.52484989	-5.30871868
H	-0.57893211	-7.15405893	-5.31558514
C	3.21701884	-5.89080524	0.64460802
H	1.21139312	-5.07481527	0.65409487
H	1.51475036	-6.23478842	-0.66380769
C	2.92488337	-7.05538368	1.59607863
H	3.97957683	-6.19701004	-0.09587019
H	3.64921737	-5.04706240	1.21167636
H	3.84012604	-7.38829565	2.11061788
H	2.19508123	-6.75880861	2.36732960
H	2.50835776	-7.92037106	1.05276632
O	-4.06780195	0.17282265	2.03856850
H	-3.22348428	-0.33436617	2.06025958
H	-4.34694338	0.15180314	2.96954536

(NO_3^- in outer coordination sphere)

E= -15089.675885, E0 = -15088.963990, G= 382.3431 at T=298.150 K

Tb	-0.51039428	-0.52922684	-0.08533583
Cl	3.87726283	1.93962169	4.81938887
Cl	2.70349669	5.51202202	-1.79569256
O	-1.05808973	0.40355265	-2.13381767
O	-0.67813766	-1.41463852	2.17055035
O	-2.85774779	-0.98244691	0.06385112
O	-3.77027893	-2.87163305	-0.61877114
O	-1.60248291	-2.52440929	-0.83719385
O	1.12346733	-2.33922076	0.25577661
O	1.12832510	-1.31045675	-1.66857970
O	2.51385379	-2.99297833	-1.32509494
O	-1.59335947	1.34286201	0.84913307
H	-1.46164227	2.38114023	0.61122644
H	-2.55602908	1.17834795	0.82756221
N	1.22499192	0.29163179	1.55099392
N	0.65723902	-2.60748005	3.56284642
N	0.83871454	1.52799892	-0.79200995
N	-0.84139162	1.61771810	-4.00929832
N	-2.79578400	-2.16462660	-0.47404128
N	1.63374174	-2.25445700	-0.93144393
C	1.34051549	-0.34415951	2.72294927
C	2.15072966	0.15583958	3.75713301
H	2.18921328	-0.32943031	4.73168325
C	2.87695694	1.31467736	3.53647065
C	2.79360890	2.00110245	2.29732203
C	3.47555566	3.21480751	1.98681593
H	4.15145969	3.63815069	2.73117399
C	3.26914930	3.85288405	0.79379946
H	3.77570367	4.79405403	0.57505083
C	2.36807132	3.32734346	-0.17856233

C	1.70544136	2.09520435	0.06749933
C	1.91938102	1.42370021	1.33341360
C	2.01351619	3.96954608	-1.38846135
C	1.09196401	3.40328121	-2.25065351
H	0.77629083	3.97974324	-3.11265898
C	0.52859974	2.14836240	-1.93825448
C	-0.51265651	1.36635089	-2.72814989
C	-0.31504843	2.68165946	-4.87414455
H	-0.34962341	2.29030204	-5.90471649
H	0.75253814	2.83382463	-4.65529108
C	-1.11837232	3.98718500	-4.76783466
H	-2.13496065	3.81097698	-5.16256809
H	-1.25982964	4.25523901	-3.70430064
C	-0.46089685	5.13881493	-5.53618336
H	-0.34908131	4.86588144	-6.60195208
H	0.56512386	5.30047655	-5.15277529
C	-1.25839913	6.44120884	-5.40876770
H	-2.27140021	6.32551098	-5.82810926
H	-0.76348829	7.26716948	-5.94353008
H	-1.36409366	6.72815323	-4.35022926
C	-1.90196371	0.77078122	-4.59701061
H	-2.39796805	1.37474990	-5.37400007
H	-2.63693905	0.55367565	-3.80814147
C	-1.35120726	-0.53231275	-5.18668509
H	-0.81803501	-1.08120477	-4.39162445
H	-0.61116683	-0.29941580	-5.97503471
C	-2.46573520	-1.41187274	-5.76448250
H	-3.19434524	-1.63805830	-4.96487617
H	-3.01718044	-0.85044819	-6.54182911
C	-1.93080604	-2.72164154	-6.35153723
H	-1.21447682	-2.52924299	-7.16788054

H	-2.74730444	-3.34003305	-6.75678205
H	-1.41043854	-3.31242943	-5.57986784
C	0.38817286	-1.51451266	2.82012439
C	1.98337078	-2.96245217	4.08661079
H	2.72113085	-2.24790621	3.69440556
H	2.24963832	-3.94252777	3.65111804
C	2.05066156	-3.03735399	5.61510134
H	1.75654256	-2.06432176	6.05078650
H	1.31138909	-3.77212811	5.98105192
C	3.44976854	-3.42813969	6.10616541
H	4.18623447	-2.69027805	5.73834276
H	3.73385715	-4.39704370	5.65692997
C	3.53442955	-3.52361560	7.63257647
H	3.28377628	-2.56007123	8.10616302
H	4.54832315	-3.80457449	7.95741129
H	2.83405423	-4.28063726	8.02203178
C	-0.37452620	-3.67056775	3.55789065
H	0.12475011	-4.59816790	3.87988448
H	-0.70513487	-3.80121589	2.51524854
C	-1.56907248	-3.36562634	4.46879721
H	-2.00394511	-2.39725304	4.16943932
H	-1.21247995	-3.26125550	5.50869799
C	-2.65334892	-4.45415401	4.39709520
H	-2.20617747	-5.43740606	4.63722420
H	-3.38978219	-4.25115061	5.19397593
C	-3.38011980	-4.52720642	3.04844356
H	-3.81801915	-3.55108953	2.78093410
H	-4.19756603	-5.26464748	3.08400559
H	-2.71026707	-4.82256413	2.22501349
O	-1.13919926	3.77500510	0.39068362
O	-1.08360910	5.25181723	-1.23383236

O -2.11357641 3.33798313 -1.54591250

N -1.46343195 4.13933372 -0.82287627

L2*Tb($(NO_3)_2^+$)

E= -15089.681394, E0 = -15088.969479, G= 381.1737 at T=298.150 K

Cl -5.36586714 3.73997188 -0.50237966

Cl -4.12523890 -3.03251433 -3.77973294

N -1.57930803 1.35430419 0.08889898

N -0.25083527 4.05573225 2.04057860

N -1.13055027 -0.99542338 -1.08486140

N 1.18761623 -3.63775516 -1.79588604

O 0.05387235 1.81347227 2.03332591

O 0.83114254 -2.31102562 0.00488343

C -1.74536943 2.53497171 0.69885135

C -2.91912889 3.28960180 0.53191704

H -3.08819771 4.21150732 1.08460557

C -3.91197562 2.81795144 -0.31677532

C -3.74529934 1.59098518 -1.00917184

C -4.70349932 1.01403439 -1.89818847

H -5.62800694 1.55948794 -2.09384537

C -4.48050022 -0.20036353 -2.48732233

H -5.22592402 -0.63293034 -3.15646887

C -3.27905440 -0.93241644 -2.23865008

C -2.98345280 -2.21630192 -2.76574349

C -1.78186405 -2.84277177 -2.46349788

H -1.59355164 -3.84592867 -2.84241295

C -0.85756040 -2.18505168 -1.63327157

C -2.29358482 -0.37986204 -1.37791967

C -2.53318024 0.89826918 -0.74776047

C -0.59359556 2.82399082 1.64051342

C 0.45477179 -2.74165344 -1.12012041

C -0.76976717 5.32467604 1.49802959

C	-1.72744834	6.05940628	2.44319081
H	-1.23526466	5.13343716	0.52006054
C	0.88688260	4.17138004	2.98723888
H	0.82169902	3.33064318	3.69295907
H	0.73609847	5.10734224	3.54786944
C	2.39990425	-4.16695881	-1.11810374
H	2.92577171	-3.31652570	-0.65488684
H	3.04369092	-4.58054256	-1.90926695
C	1.00842607	-3.99690557	-3.21544814
H	0.31104904	-3.28321433	-3.67680097
C	0.56065071	-5.44314528	-3.44765472
Tb	0.23084009	-0.21444194	0.87929791
N	-1.11282253	-1.52102196	2.95241570
O	0.17305923	-1.28985548	2.95305657
O	-1.73355007	-1.08226705	1.88333976
O	-1.67988539	-2.08528590	3.85097742
N	2.62796330	0.71476907	-0.23891862
O	3.65918612	1.12279105	-0.71146429
O	2.54090595	0.23852555	0.97263086
O	1.49594355	0.71499151	-0.89949220
H	0.11097562	5.95852089	1.29744267
C	2.24918675	4.17352486	2.28457928
C	-2.22637391	7.37614155	1.83383274
H	-2.58584571	5.41045046	2.69835401
H	-1.21499348	6.26652145	3.39890027
C	-3.16154552	8.13971138	2.77635694
H	-1.35749364	8.00942421	1.57942164
H	-2.74581385	7.16790295	0.88012111
H	-3.50461888	9.07793140	2.31470108
H	-4.05320883	7.54241610	3.02785611
H	-2.65167737	8.39577866	3.71911621

C	3.40085268	4.32717371	3.28607845
H	2.36876488	3.22541356	1.73315787
H	2.28930402	4.99241543	1.54285145
C	4.77002048	4.31225348	2.59983301
H	3.27515554	5.26763058	3.85336447
H	3.34835148	3.50553894	4.02314901
H	5.58035278	4.41935873	3.33715606
H	4.92991447	3.36583662	2.05750799
H	4.86246061	5.13762474	1.87478042
C	2.09299088	-5.23300982	-0.06054753
H	1.98424292	-3.82762980	-3.70363450
C	0.39038330	-5.74803209	-4.94171810
H	1.30454886	-6.13329077	-3.01278615
H	-0.38636619	-5.63813782	-2.91048551
C	-0.02958202	-7.19826698	-5.19933319
H	-0.35856211	-5.05959463	-5.37502670
H	1.34087527	-5.53839636	-5.46461964
H	-0.14551650	-7.38859940	-6.27702856
H	0.72353733	-7.90377665	-4.81281662
H	-0.99017841	-7.43062973	-4.71116400
C	3.37796998	-5.73193407	0.61316025
H	1.42088437	-4.80358124	0.70131260
H	1.56199527	-6.08018208	-0.52968800
C	3.10108399	-6.80117321	1.67419183
H	4.06399584	-6.13645649	-0.15361942
H	3.89798594	-4.87482166	1.07800579
H	4.03704643	-7.14215803	2.14260435
H	2.44945645	-6.40852308	2.47186518
H	2.60424995	-7.68145561	1.23417187

L2*Dy(NO_3)$_3$(H_2O)

(H_2O in outer coordination sphere)

E= -15538.290842, E0 = -15537.578790, G= 381.2858 at T=298.150 K

Cl	-5.43009901	3.68634009	-0.39969873
Cl	-4.26394129	-3.07405353	-3.73419547
N	-1.62231791	1.29580939	0.09796743
N	-0.25080734	4.02861977	1.96131504
N	-1.21576476	-1.06395495	-1.05687940
N	1.07312000	-3.69868731	-1.69510663
O	0.00860669	1.78106356	2.07501745
O	0.47218844	-2.63308311	0.21818519
C	-1.77446580	2.47058558	0.70444167
C	-2.95296741	3.22942686	0.57308191
H	-3.10398507	4.14948225	1.13475573
C	-3.96075177	2.76015186	-0.25039402
C	-3.81052375	1.54006457	-0.95047414
C	-4.78738737	0.96500301	-1.81814563
H	-5.71660042	1.51090717	-1.98850298
C	-4.58092022	-0.24989723	-2.41065025
H	-5.34195042	-0.68382722	-3.06103396
C	-3.37917519	-0.98796934	-2.18317199
C	-3.10153508	-2.27299213	-2.71093965
C	-1.91592717	-2.92104554	-2.40656614
H	-1.73052859	-3.92710757	-2.78266740
C	-0.98694181	-2.27101636	-1.57357335
C	-2.37033272	-0.43730554	-1.34266520
C	-2.59180450	0.84767675	-0.71316493
C	-0.60721564	2.76937938	1.62514865
C	0.25790560	-2.89900041	-0.98142898
C	-0.69537246	5.25341606	1.28354144
C	-1.59058809	6.15640879	2.14107418
H	-1.19669604	4.97972155	0.34355128
C	0.88983548	4.17036009	2.89436150

H	0.81081676	3.35729027	3.63095403
H	0.75869668	5.13020515	3.42113781
C	2.26411343	-4.21959114	-0.98276645
H	2.67625904	-3.39401484	-0.37696397
H	3.00169158	-4.48778009	-1.75564492
C	1.04717779	-3.81824636	-3.15806246
H	0.31297037	-3.10291696	-3.55701208
C	0.75594449	-5.23498678	-3.66259575
Dy	0.51497823	-0.21724124	0.76746225
N	-0.92847306	-1.16806400	3.04745221
O	0.34395260	-1.01047421	3.04101443
O	-1.51241803	-0.94064939	1.89374089
O	-1.56486678	-1.49391961	4.03083849
N	3.25907707	-0.30678421	1.56937742
O	2.55703497	0.78829628	1.46091175
O	2.60873485	-1.38158143	1.28977954
O	4.42840004	-0.29579550	1.90447438
N	1.59900928	0.80384707	-1.68274283
O	2.06619239	1.21883774	-2.72612023
O	1.70115113	-0.44239658	-1.32044160
O	0.95596737	1.55557811	-0.85206211
H	0.21460363	5.80695820	0.99143243
C	2.25337911	4.10341835	2.19786644
C	-2.02832890	7.41304684	1.37897062
H	-2.47983766	5.59437704	2.48146367
H	-1.05002332	6.44992495	3.05816841
C	-2.91495800	8.33609295	2.22038341
H	-1.13133693	7.96417665	1.04284739
H	-2.56746531	7.11368608	0.46128118
H	-3.21542835	9.22800350	1.64872837
H	-3.83275414	7.81861210	2.54502821

H	-2.38554931	8.67801094	3.12489605
C	3.40445685	4.17356777	3.20859051
H	2.32285380	3.15254402	1.64544177
H	2.34806228	4.92562819	1.46445751
C	4.77145958	3.99794126	2.53935218
H	3.36873460	5.13290644	3.75897479
H	3.26533008	3.37307596	3.95799208
H	5.58647394	4.06164885	3.27774048
H	4.83328772	3.01371431	2.04745793
H	4.94512272	4.77443933	1.77518189
C	1.96249139	-5.42264986	-0.08422456
H	2.03183389	-3.48079991	-3.52921391
C	0.75921929	-5.30772209	-5.19418859
H	1.51012468	-5.93300343	-3.25757504
H	-0.22058953	-5.57655573	-3.27150893
C	0.48140380	-6.71960258	-5.71934938
H	0.00481499	-4.60648966	-5.59580660
H	1.73701942	-4.95697832	-5.57117367
H	0.48903394	-6.74314690	-6.82026052
H	1.24265122	-7.43235254	-5.36184549
H	-0.50297070	-7.08342505	-5.38123178
C	3.21827817	-5.88670206	0.66336954
H	1.19008100	-5.13009357	0.64684170
H	1.55245209	-6.25290394	-0.68809146
C	2.94891071	-7.08793020	1.57467294
H	4.01033163	-6.14224100	-0.06518820
H	3.60488009	-5.04424238	1.26426840
H	3.86403656	-7.39993000	2.10239697
H	2.18980956	-6.84197521	2.33541083
H	2.57992244	-7.95277023	0.99787158
O	-4.10275507	0.20703349	2.06985903

H -3.26296139 -0.30818120 2.08035207

H -4.37556696 0.17680091 3.00244689

(NO_3^- in outer coordination sphere)

E= -15538.284852, E0 = -15537.572656, G= 382.5816 at T=298.150 K

Dy -0.54037243 -0.52262110 -0.07815127

Cl 3.83082438 1.97670507 4.84258080

Cl 2.70911312 5.50287819 -1.80324221

O -1.07835674 0.41436946 -2.12814665

O -0.70219934 -1.40137935 2.19294071

O -2.89198136 -0.94437665 0.10439493

O -3.81492400 -2.84769702 -0.52336508

O -1.64952636 -2.50614047 -0.77722418

O 1.10273480 -2.30134296 0.26501864

O 1.04486763 -1.34285653 -1.69230258

O 2.45464587 -3.00093341 -1.33004773

O -1.61191130 1.35902798 0.84536314

H -1.47139049 2.39074087 0.61194956

H -2.57578826 1.20226848 0.83218968

N 1.20539808 0.30842677 1.56818640

N 0.65397555 -2.59782386 3.56233382

N 0.83005327 1.52671170 -0.78628516

N -0.83714408 1.61128724 -4.01256561

N -2.83890247 -2.13661385 -0.40677422

N 1.57881558 -2.25475097 -0.93992352

C 1.31469631 -0.32094660 2.73913860

C 2.11630750 0.18415819 3.77846074

H 2.15029287 -0.29806599 4.75477219

C 2.84089351 1.34394348 3.55587387

C 2.76697326 2.02187610 2.31358552

C 3.45179486 3.23434854 1.99993968

H 4.12279463 3.66333985 2.74539709

C	3.25218105	3.86183310	0.80174124
H	3.75965786	4.80166674	0.57885885
C	2.35677600	3.32948256	-0.17434733
C	1.69227171	2.09976339	0.07387216
C	1.89946067	1.43825591	1.34665024
C	2.01168871	3.96575594	-1.38941133
C	1.09275794	3.39579678	-2.25238824
H	0.78148931	3.96827149	-3.11864996
C	0.52471638	2.14442182	-1.93558586
C	-0.51992238	1.36425281	-2.72645378
C	-0.29894885	2.66705537	-4.87960720
H	-0.33060792	2.27138829	-5.90862417
H	0.76870066	2.81336141	-4.65645838
C	-1.09389412	3.97869754	-4.78295183
H	-2.10760856	3.80935550	-5.18795872
H	-1.24460196	4.24677753	-3.72059321
C	-0.42108208	5.12584066	-5.54462624
H	-0.30392739	4.85366678	-6.61000681
H	0.60358590	5.27820635	-5.15375948
C	-1.20814908	6.43492889	-5.42066097
H	-2.21953249	6.32839584	-5.84629250
H	-0.70296460	7.25726128	-5.95142078
H	-1.31775463	6.72152567	-4.36239386
C	-1.90065980	0.76933920	-4.60158443
H	-2.38794613	1.37240565	-5.38485718
H	-2.64146662	0.56183791	-3.81556988
C	-1.35707092	-0.54139042	-5.18090725
H	-0.83492160	-1.09131348	-4.37920284
H	-0.60887355	-0.31893268	-5.96458244
C	-2.47559214	-1.41233218	-5.76397419
H	-3.21003628	-1.63250864	-4.96807575

H	-3.01868534	-0.84618133	-6.54385328
C	-1.94968736	-2.72644258	-6.34952831
H	-1.22717905	-2.54001164	-7.16182852
H	-2.77003098	-3.33631706	-6.75993490
H	-1.43926132	-3.32378626	-5.57619429
C	0.36705238	-1.50125670	2.83211350
C	1.98694384	-2.94145274	4.07624149
H	2.71374393	-2.21146917	3.69196630
H	2.26574063	-3.91127062	3.62602210
C	2.06016755	-3.03610730	5.60331678
H	1.75604331	-2.07238698	6.05270100
H	1.33067310	-3.78425956	5.96161795
C	3.46521759	-3.41684031	6.08516836
H	4.19219732	-2.66648388	5.72377157
H	3.75862455	-4.37711811	5.62363195
C	3.55585885	-3.52933145	7.61007547
H	3.29575944	-2.57434154	8.09575748
H	4.57386398	-3.80256486	7.92855358
H	2.86532259	-4.29877329	7.99267340
C	-0.36369833	-3.67479753	3.54695773
H	0.14897294	-4.59965801	3.85552812
H	-0.69509894	-3.79584312	2.50329828
C	-1.55994058	-3.39754581	4.46431446
H	-2.01105714	-2.43353128	4.17518759
H	-1.20195282	-3.29744458	5.50417995
C	-2.62668729	-4.50279522	4.38501692
H	-2.16299653	-5.48052216	4.61619949
H	-3.36478472	-4.31844521	5.18489313
C	-3.35449553	-4.57644510	3.03698492
H	-3.81007552	-3.60613561	2.77829242
H	-4.15881681	-5.32842112	3.06751299

H -2.68131208 -4.85270452 2.20973873

O -1.12600720 3.79592538 0.39648446

O -1.03070974 5.28309917 -1.21665859

O -2.07541299 3.38217235 -1.55711579

N -1.42770147 4.17188740 -0.81736237

L2*Dy(NO$_3$)$_2$$^+$

E= -15538.290842, E0 = -15537.578790, G= 381.2858 at T=298.150 K

Cl -5.36758709 3.74894166 -0.51552254

Cl -4.12725258 -3.02189350 -3.79348969

N -1.58097196 1.36686802 0.07094847

N -0.25369540 4.05466032 2.03829622

N -1.13306415 -0.98574811 -1.09866834

N 1.18273425 -3.63233972 -1.80062079

O 0.04730368 1.81196272 2.02268863

O 0.82830447 -2.29794502 -0.00550822

C -1.74910688 2.54165626 0.68802410

C -2.92378354 3.29660559 0.52317137

H -3.09475398 4.21601105 1.07951355

C -3.91351008 2.82790589 -0.33003950

C -3.74527025 1.60343587 -1.02712786

C -4.70193815 1.02813411 -1.91877103

H -5.62614775 1.57361770 -2.11565089

C -4.47799349 -0.18586186 -2.50843096

H -5.22256899 -0.61764222 -3.17906594

C -3.27801013 -0.91959596 -2.25722241

C -2.98460793 -2.20598578 -2.78044248

C -1.78553689 -2.83408165 -2.47440839

H -1.59912694 -3.83879018 -2.85016322

C -0.86094701 -2.17582369 -1.64374888

C -2.29243374 -0.36874482 -1.39585197

C -2.53266954 0.91144633 -0.76568407

C	-0.59683222	2.82479167	1.63353086
C	0.45199025	-2.73197031	-1.12749112
C	-0.77298725	5.32607698	1.50164378
C	-1.73285973	6.05503225	2.44898629
H	-1.23665702	5.14006996	0.52177322
C	0.88503373	4.16590023	2.98438096
H	0.82244217	3.31979418	3.68383241
H	0.73342383	5.09760332	3.55186629
C	2.39668202	-4.15703201	-1.12234938
H	2.92163754	-3.30367255	-0.66347355
H	3.03996539	-4.57329130	-1.91254413
C	1.00058496	-3.99981928	-3.21747494
H	0.30453005	-3.28750467	-3.68288779
C	0.54893076	-5.44650316	-3.43969440
Dy	0.22986785	-0.20782039	0.87054920
N	-1.05762541	-1.54890943	2.94405437
O	0.21497487	-1.27554381	2.95402479
O	-1.68492615	-1.13231444	1.86574817
O	-1.61826372	-2.12964225	3.83669305
N	2.65814900	0.67580223	-0.18973665
O	3.69973564	1.07658851	-0.64646524
O	2.54843330	0.17913343	1.00800347
O	1.53127992	0.70833284	-0.86234444
H	0.10763530	5.96146297	1.30570734
C	2.24621034	4.17513466	2.27960324
C	-2.22576833	7.37797976	1.84825444
H	-2.59414196	5.40609932	2.69441819
H	-1.22448575	6.25205708	3.40898061
C	-3.17777586	8.12805653	2.78464913
H	-1.35467088	8.01602554	1.61432612
H	-2.73035789	7.17985153	0.88444728

H	-3.51406097	9.07176113	2.32913923
H	-4.07264471	7.52585983	3.01171494
H	-2.68531752	8.37227440	3.73969769
C	3.39912915	4.31667566	3.28146505
H	2.36428785	3.23338032	1.71707916
H	2.28601217	5.00215960	1.54696369
C	4.76771069	4.31008720	2.59390950
H	3.27367902	5.25031281	3.86003637
H	3.34730864	3.48620749	4.00856590
H	5.57818604	4.40854549	3.33230019
H	4.92753124	3.37014437	2.04046249
H	4.85970736	5.14414358	1.87880599
C	2.09318924	-5.21848392	-0.05916716
H	1.97591925	-3.83623743	-3.70853019
C	0.37274060	-5.76010752	-4.93125439
H	1.29305267	-6.13540363	-3.00325370
H	-0.39642513	-5.63647175	-2.89777517
C	-0.04622607	-7.21238613	-5.17882729
H	-0.37877190	-5.07499933	-5.36545420
H	1.32082570	-5.55241299	-5.45929527
H	-0.16774566	-7.40882874	-6.25482082
H	0.71041036	-7.91438770	-4.79278708
H	-1.00352931	-7.44411755	-4.68392706
C	3.38040805	-5.71164179	0.61451900
H	1.42186308	-4.78632259	0.70180923
H	1.56281793	-6.06890249	-0.52313828
C	3.10798192	-6.77623653	1.68137062
H	4.06574392	-6.11849403	-0.15165704
H	3.89939904	-4.85117197	1.07422531
H	4.04589128	-7.11289263	2.14910007
H	2.45748472	-6.38091850	2.47862840

H 2.61192012 -7.65969658 1.24685764

L2*Ho(NO_3)$_3$(H_2O)

(H_2O in outer coordination sphere)

E= -15997.733757, E0 = -15997.021703, G= 381.2149 at T=298.150 K

Cl	-5.43554211	3.68190002	-0.40767777
Cl	-4.25478649	-3.07395840	-3.74906301
N	-1.62530673	1.29563880	0.08996341
N	-0.25371474	4.02834463	1.95297956
N	-1.21572161	-1.06286108	-1.06132460
N	1.07845342	-3.69495344	-1.70259643
O	0.00430948	1.78089774	2.06546474
O	0.47930837	-2.62224698	0.20680608
C	-1.77819955	2.47026849	0.69609040
C	-2.95755529	3.22790670	0.56473905
H	-3.10920358	4.14831018	1.12572956
C	-3.96514153	2.75732660	-0.25833493
C	-3.81363368	1.53746808	-0.95841503
C	-4.78889084	0.96137685	-1.82716179
H	-5.71890068	1.50579667	-1.99799967
C	-4.57955551	-0.25246823	-2.42088985
H	-5.33897543	-0.68688673	-3.07284427
C	-3.37676477	-0.98873228	-2.19298458
C	-3.09539652	-2.27184892	-2.72316241
C	-1.90814948	-2.91687894	-2.41948891
H	-1.71965098	-3.92131972	-2.79827476
C	-0.98226398	-2.26679397	-1.58275616
C	-2.37027431	-0.43819967	-1.34954751
C	-2.59423470	0.84659046	-0.72025752
C	-0.61044955	2.76936698	1.61640120
C	0.26375753	-2.89214325	-0.99118733
C	-0.70234537	5.25495672	1.28142154

C	-1.59576023	6.15192938	2.14702535
H	-1.20595634	4.98491144	0.34160772
C	0.89172310	4.16841125	2.88028359
H	0.81584507	3.35544658	3.61731958
H	0.76507849	5.12858868	3.40759206
C	2.26780176	-4.21652985	-0.98789245
H	2.68122554	-3.39045620	-0.38380700
H	3.00519323	-4.48905373	-1.75945115
C	1.05082250	-3.82342124	-3.16469264
H	0.31850174	-3.10842204	-3.56760097
C	0.75535929	-5.24239349	-3.66068578
Ho	0.51543206	-0.21847446	0.77683812
N	-0.92657250	-1.16616237	3.04778624
O	0.34359455	-1.00101340	3.05013442
O	-1.50119555	-0.94600272	1.88772368
O	-1.57028627	-1.49182260	4.02663040
N	3.23709750	-0.34402102	1.61404026
O	2.53888249	0.75396812	1.53467488
O	2.58415198	-1.40677822	1.29417503
O	4.40334845	-0.34967107	1.95922089
N	1.61413193	0.86192250	-1.63197148
O	2.09174085	1.29845929	-2.66172695
O	1.69743407	-0.39533460	-1.30293500
O	0.98053759	1.59830928	-0.78204185
H	0.20585823	5.81144619	0.98940927
C	2.25100970	4.09970045	2.17570281
C	-2.02410531	7.42172527	1.40182734
H	-2.48901868	5.59046221	2.47771668
H	-1.05608344	6.42995024	3.06951022
C	-2.90294194	8.33939075	2.25734186
H	-1.12288713	7.97054720	1.07326651

H	-2.56657529	7.14055490	0.48042291
H	-3.19670987	9.24232674	1.69969463
H	-3.82466316	7.82437992	2.57472801
H	-2.37022138	8.66288280	3.16665554
C	3.40801382	4.17708302	3.17890954
H	2.31721640	3.14639235	1.62712467
H	2.33958077	4.91833496	1.43752682
C	4.77194881	4.00781488	2.50196481
H	3.37057662	5.13760567	3.72714520
H	3.27773190	3.37753105	3.93077421
H	5.59082031	4.07572031	3.23569345
H	4.83606148	3.02358222	2.01039457
H	4.93754005	4.78456068	1.73626053
C	1.96255767	-5.41595411	-0.08561295
H	2.03633308	-3.49171901	-3.53879166
C	0.75782037	-5.32431841	-5.19181347
H	1.50787711	-5.93993187	-3.25180984
H	-0.22182854	-5.57913351	-3.26706338
C	0.47662944	-6.73870373	-5.70838118
H	0.00483558	-4.62381935	-5.59736252
H	1.73629177	-4.97810745	-5.57125616
H	0.48393589	-6.76892805	-6.80912828
H	1.23633468	-7.45100737	-5.34673929
H	-0.50848025	-7.09822464	-5.36783314
C	3.21701765	-5.88164806	0.66318184
H	1.19125700	-5.11852789	0.64463800
H	1.54977572	-6.24678373	-0.68680155
C	2.94278550	-7.07671499	1.58117330
H	4.00719547	-6.14455986	-0.06479184
H	3.60783982	-5.03782129	1.25940132
H	3.85733175	-7.39170933	2.10807657

H 2.18717790 -6.82215977 2.34257555

H 2.56683993 -7.94216347 1.00972855

O -4.10357380 0.17909481 2.04371977

H -3.25842476 -0.32708147 2.05971408

H -4.37998295 0.14868346 2.97523093

(NO_3^- in outer coordination sphere)

E= -15997.730185, E0 = -15997.017725, G= 382.8520 at T=298.150 K

Ho -0.51321846 -0.52220935 -0.08571949

Cl 3.84537220 1.96798503 4.83155107

Cl 2.74077964 5.47812605 -1.82666993

O -1.08802056 0.41882703 -2.11107898

O -0.67418879 -1.42630935 2.15821505

O -2.83727002 -0.94461870 0.08509265

O -3.77448416 -2.82557511 -0.58760780

O -1.60618174 -2.49752283 -0.82874650

O 1.14034665 -2.27746224 0.22955221

O 1.07096231 -1.29852021 -1.71791363

O 2.49553204 -2.94788575 -1.37547505

O -1.54969847 1.35762644 0.86531854

H -1.40719235 2.38665199 0.62991381

H -2.51297402 1.20304000 0.81944442

N 1.21582985 0.30098253 1.56077135

N 0.66573650 -2.60693407 3.55518413

N 0.83800960 1.51772451 -0.79204369

N -0.85654157 1.60777628 -4.00199986

N -2.79123831 -2.12802935 -0.45367900

N 1.61395419 -2.21289539 -0.97666037

C 1.32588303 -0.32859248 2.73102903

C 2.12737012 0.17742148 3.77010560

H 2.15918756 -0.30123922 4.74813032

C 2.85549092 1.33463776 3.54508710

C	2.78582168	2.00882530	2.30081868
C	3.47685504	3.21666884	1.98268998
H	4.15086174	3.64450359	2.72607827
C	3.27910280	3.84135938	0.78269678
H	3.79108858	4.77793407	0.55632967
C	2.37871146	3.31174064	-0.19040209
C	1.70773196	2.08691597	0.06274920
C	1.91441309	1.42716956	1.33620119
C	2.03324676	3.94735980	-1.40573323
C	1.10530519	3.38257146	-2.26249242
H	0.79407620	3.95583391	-3.12829709
C	0.52927482	2.13628244	-1.93975818
C	-0.52837574	1.36252475	-2.71850681
C	-0.32349694	2.66194320	-4.87411594
H	-0.36963269	2.26806426	-5.90323687
H	0.74749273	2.80244470	-4.66369152
C	-1.11104131	3.97708273	-4.76536894
H	-2.13038802	3.81299567	-5.15829277
H	-1.24749088	4.24409819	-3.70082402
C	-0.44291598	5.12314034	-5.53274632
H	-0.33986688	4.85314274	-6.60014677
H	0.58701706	5.27062845	-5.15405416
C	-1.22340274	6.43487835	-5.39567184
H	-2.24023271	6.33320904	-5.80937147
H	-0.72161597	7.25692272	-5.93008471
H	-1.31919205	6.71858311	-4.33528662
C	-1.92787659	0.76809353	-4.57991409
H	-2.41900587	1.37020731	-5.36152220
H	-2.66351390	0.56585342	-3.78767610
C	-1.39297664	-0.54642314	-5.15878057
H	-0.86634529	-1.09502912	-4.35908937

H	-0.65007603	-0.32903188	-5.94887638
C	-2.51847219	-1.41614091	-5.73006868
H	-3.24724460	-1.63168073	-4.92772293
H	-3.06609988	-0.85115105	-6.50762606
C	-2.00057697	-2.73361301	-6.31524563
H	-1.28405416	-2.55177784	-7.13386774
H	-2.82566714	-3.34281564	-6.71705008
H	-1.48551297	-3.32953334	-5.54389906
C	0.38451445	-1.51462007	2.81634355
C	1.98930800	-2.93925905	4.09995747
H	2.72176123	-2.21279907	3.71997333
H	2.27914000	-3.91442275	3.66883469
C	2.03262544	-3.01343465	5.62937450
H	1.71410108	-2.04576135	6.05988884
H	1.30008578	-3.76094532	5.98272705
C	3.42981219	-3.37980413	6.14411497
H	4.15965700	-2.62967515	5.78802824
H	3.73799872	-4.34401131	5.70075274
C	3.49015689	-3.47265267	7.67184782
H	3.21489072	-2.51321292	8.14006424
H	4.50301552	-3.73588634	8.01423359
H	2.79633474	-4.24129009	8.05007267
C	-0.34579507	-3.68953729	3.52745652
H	0.16639550	-4.60992813	3.84980226
H	-0.65828061	-3.81765151	2.47886205
C	-1.55973995	-3.41469932	4.42214870
H	-2.00793862	-2.45197201	4.12448406
H	-1.22168088	-3.31378222	5.46855402
C	-2.62200880	-4.52269936	4.32262802
H	-2.15968156	-5.49982214	4.55898857
H	-3.37375450	-4.34229565	5.11058617

C -3.32725000 -4.59446144 2.96252918

H -3.78178740 -3.62475753 2.69986606

H -4.12930250 -5.34930611 2.97729158

H -2.63944101 -4.86572409 2.14567566

O -1.06459880 3.79750276 0.40935916

O -0.99966121 5.28605795 -1.20403504

O -2.05713844 3.38846731 -1.52352977

N -1.39110887 4.17559671 -0.79682231

L2*Ho(NO_3)$_2^+$

E= -15640.772405, E0 = -15640.098904, G= 362.8072 at T=298.150 K

Cl -5.36756039 3.75597620 -0.52836746

Cl -4.12054253 -3.00795817 -3.82315016

N -1.58169329 1.37144184 0.05544505

N -0.25953370 4.05167198 2.03860879

N -1.14079237 -0.98346847 -1.10440600

N 1.17751324 -3.62998796 -1.79001677

O 0.04610882 1.81051040 2.00878549

O 0.80566263 -2.29390883 0.00173162

C -1.75141275 2.54392910 0.67765844

C -2.92644215 3.29841495 0.51413590

H -3.09837079 4.21538401 1.07421553

C -3.91465569 2.83350420 -0.34308246

C -3.74568915 1.61116767 -1.04382503

C -4.70010471 1.03869426 -1.93981326

H -5.62323713 1.58551717 -2.13808060

C -4.47522736 -0.17394899 -2.53216219

H -5.21781158 -0.60300165 -3.20676923

C -3.27752233 -0.91033834 -2.27744889

C -2.98285913 -2.19636846 -2.80130792

C -1.78663456 -2.82681775 -2.48914146

H -1.59854257 -3.83049250 -2.86685681

C	-0.86689103	-2.17297649	-1.64941251
C	-2.29559708	-0.36272946	-1.41035914
C	-2.53427172	0.91791344	-0.78161180
C	-0.60008132	2.82349110	1.62582946
C	0.44133106	-2.72974229	-1.12306821
C	-0.78062010	5.32581615	1.51080120
C	-1.74318087	6.04561996	2.46242380
H	-1.24295962	5.14656973	0.52896899
C	0.87986112	4.15721416	2.98495603
H	0.81096494	3.31361008	3.68695855
H	0.73444462	5.09186792	3.54917049
C	2.38669109	-4.15353966	-1.10272300
H	2.91011977	-3.29904318	-0.64399624
H	3.03417516	-4.57262754	-1.88792658
C	1.00587201	-3.99702811	-3.20824528
H	0.31768450	-3.28094530	-3.67971349
C	0.54909730	-5.44140244	-3.43457007
Ho	0.23256919	-0.18644863	0.83216757
N	-0.91900867	-1.52296495	2.96185040
O	0.34956989	-1.23234820	2.89946151
O	-1.60921943	-1.12134326	1.91770160
O	-1.42023563	-2.10546446	3.88763022
N	2.64086175	0.65320289	-0.28290820
O	3.68452120	1.01630139	-0.76542372
O	2.51100969	0.32815632	0.97246903
O	1.53392267	0.55419654	-0.97927696
H	0.09900480	5.96438932	1.32067502
C	2.24140859	4.15365362	2.28068376
C	-2.23953032	7.37138462	1.87085295
H	-2.60271144	5.39243841	2.70274115
H	-1.23588896	6.23749065	3.42402697

C	-3.19477820	8.11135674	2.81196523
H	-1.37015319	8.01383495	1.64266932
H	-2.74251413	7.17898178	0.90503180
H	-3.53333402	9.05779266	2.36391997
H	-4.08813572	7.50477028	3.03322148
H	-2.70409346	8.34926128	3.76951098
C	3.39419746	4.30261517	3.28169966
H	2.35689139	3.20424151	1.73051596
H	2.28395987	4.97282791	1.53932619
C	4.76279306	4.28015375	2.59449959
H	3.27338433	5.24406147	3.84850860
H	3.33804488	3.48141837	4.01891136
H	5.57418442	4.38333225	3.33122540
H	4.91684866	3.33247185	2.05281234
H	4.85921144	5.10471725	1.86900318
C	2.07655668	-5.21149445	-0.03805242
H	1.98613954	-3.83789992	-3.69062018
C	0.38937616	-5.75540400	-4.92788744
H	1.28373551	-6.13408470	-2.98814440
H	-0.40393427	-5.62573195	-2.90418005
C	-0.04921606	-7.20119381	-5.17908669
H	-0.34558603	-5.05991745	-5.37381124
H	1.34778225	-5.56288195	-5.44280767
H	-0.15664051	-7.39815235	-6.25650024
H	0.68864483	-7.91514921	-4.77894974
H	-1.01844633	-7.41435385	-4.69934464
C	3.35957980	-5.70354748	0.64443612
H	1.40130687	-4.77663803	0.71786410
H	1.54818237	-6.06288958	-0.50258422
C	3.08023167	-6.76494932	1.71268511
H	4.04923344	-6.11295462	-0.11649331

H 3.87635541 -4.84209633 1.10480821

H 4.01507282 -7.10039043 2.18736196

H 2.42476940 -6.36715412 2.50460744

H 2.58678293 -7.64958525 1.27759278

L2*Er(NO_3)$_3$(H_2O)

(H_2O in outer coordination sphere)

E= -16468.111104, E0 = -16467.398768, G= 381.8229 at T=298.150 K

Cl -5.39934015 3.71636868 -0.45171857

Cl -4.23453188 -3.04958940 -3.77446699

N -1.60164714 1.31804013 0.06289811

N -0.24051625 4.03068924 1.97010815

N -1.18592465 -1.03535461 -1.10198343

N 1.07457113 -3.71440530 -1.68739986

O 0.04939860 1.78524220 2.00747705

O 0.48720855 -2.59065437 0.19714071

C -1.75327647 2.49101853 0.67244577

C -2.92952657 3.25251508 0.53504199

H -3.08535981 4.16991758 1.09928536

C -3.93230438 2.78768659 -0.29713365

C -3.77976918 1.57022452 -1.00137568

C -4.75246668 0.99846321 -1.87595749

H -5.67940855 1.54644871 -2.05177736

C -4.54565716 -0.21746442 -2.46627951

H -5.30452394 -0.65039825 -3.11985540

C -3.34704995 -0.95871627 -2.23186612

C -3.07281685 -2.24793839 -2.75111556

C -1.89289880 -2.90069818 -2.43538356

H -1.71294737 -3.91151428 -2.80107284

C -0.96197796 -2.24939942 -1.60547185

C -2.33947825 -0.40784490 -1.39057386

C -2.56392574 0.87510765 -0.75890785

C	-0.58394104	2.77924061	1.59449959
C	0.27313039	-2.88111830	-0.99626523
C	-0.70491999	5.27405071	1.34083748
C	-1.61806178	6.12460232	2.23241234
H	-1.19730330	5.03105593	0.38781789
C	0.90032554	4.15579653	2.90582752
H	0.82995719	3.32142806	3.61918783
H	0.75966775	5.09876013	3.45995164
C	2.25418305	-4.23663568	-0.95790583
H	2.67797804	-3.40237427	-0.37233013
H	2.98976684	-4.53710890	-1.72075438
C	1.05934954	-3.85808682	-3.14822388
H	0.34040567	-3.13807321	-3.56603885
C	0.75063801	-5.27849817	-3.63145351
Er	0.51368684	-0.17665929	0.67040616
N	-0.89001882	-1.17269075	2.94510245
O	0.38038370	-1.01514590	2.92336607
O	-1.48800635	-0.92780817	1.80353177
O	-1.51470375	-1.51392817	3.93199611
N	3.27090096	-0.18127690	1.37718439
O	2.56050467	0.89638847	1.18658972
O	2.61772037	-1.27727938	1.22265720
O	4.45035028	-0.13598567	1.67429030
N	1.55374348	0.68570828	-1.84643483
O	2.01231217	1.05252683	-2.91218710
O	1.72195697	-0.51815552	-1.39064288
O	0.85150307	1.45651007	-1.08231771
H	0.19645956	5.85494757	1.07667005
C	2.26375914	4.12302303	2.20684171
C	-2.06913018	7.40909481	1.52679002
H	-2.50071287	5.53523207	2.54226732

H	-1.08601069	6.38309336	3.16491365
C	-2.96691537	8.28260612	2.40844274
H	-1.17802393	7.98509312	1.21774590
H	-2.60457397	7.14704084	0.59565461
H	-3.27751064	9.19611740	1.87781894
H	-3.87880611	7.74023533	2.70809317
H	-2.44206667	8.58892632	3.32821226
C	3.41561007	4.18977690	3.21724653
H	2.34715414	3.18439388	1.63542998
H	2.34530711	4.96173000	1.49067926
C	4.78271008	4.03490782	2.54262805
H	3.37253451	5.14092159	3.78098345
H	3.28481960	3.37845850	3.95662069
H	5.59872484	4.09461164	3.28020716
H	4.85177326	3.05929947	2.03473759
H	4.94805908	4.82458305	1.79026389
C	1.92911065	-5.41139126	-0.03063468
H	2.05228472	-3.54206610	-3.51633930
C	0.77021527	-5.37840509	-5.16137791
H	1.48874271	-5.98127604	-3.20551229
H	-0.23563260	-5.59764910	-3.24581528
C	0.47603837	-6.79476547	-5.66511774
H	0.03179924	-4.67256021	-5.58405685
H	1.75772893	-5.04973173	-5.53285313
H	0.49610284	-6.83789444	-6.76528502
H	1.22162652	-7.51297951	-5.28616190
H	-0.51786566	-7.13708639	-5.33244085
C	3.17599797	-5.88625717	0.72497368
H	1.16485453	-5.08526230	0.69472283
H	1.49972439	-6.24609566	-0.61464912
C	2.88079429	-7.05776882	1.66652703

H 3.95961618 -6.17819929 0.00099270

H 3.58439302 -5.03895283 1.30426478

H 3.79010224 -7.38026190 2.19798875

H 2.13163352 -6.77410650 2.42397356

H 2.48712969 -7.92666578 1.11251664

O -4.06015825 0.24139284 2.03881907

H -3.22638535 -0.28391704 2.02858257

H -4.32835484 0.18481886 2.97147369

(NO_3^- in outer coordination sphere)

E= -16468.106593, E0 = -16467.394192, G= 382.7415 at T=298.150 K

Er -0.47085753 -0.54065734 -0.09927899

Cl 3.87687707 1.94427323 4.81430197

Cl 2.74322605 5.46980095 -1.83235967

O -1.04823470 0.37897882 -2.13088393

O -0.64404541 -1.44339466 2.13327122

O -2.77771854 -1.01165450 -0.05653120

O -3.68668842 -2.73882771 -1.08708382

O -1.50432491 -2.42279387 -1.12594664

O 1.08580756 -2.36056709 0.20803376

O 1.21836007 -1.23592973 -1.65376401

O 2.54932928 -2.96435118 -1.32784116

O -1.56868613 1.29732370 0.82667702

H -1.41688812 2.33475542 0.62670857

H -2.52758670 1.14166474 0.72144264

N 1.25337112 0.27442297 1.54186761

N 0.66953593 -2.61344886 3.56473947

N 0.84870464 1.50538540 -0.79693890

N -0.86088902 1.60395002 -4.00203371

N -2.70580363 -2.09527111 -0.77670914

N 1.66286290 -2.22519255 -0.94743800

C 1.35884821 -0.35351664 2.71354485

C	2.15814567	0.15463613	3.75331664
H	2.18900967	-0.32171917	4.73236895
C	2.88630080	1.31209505	3.52808070
C	2.81383348	1.98906100	2.28557825
C	3.50020504	3.19989014	1.96879137
H	4.17515802	3.62807059	2.71110415
C	3.29692101	3.82654595	0.77070230
H	3.80615401	4.76472807	0.54467744
C	2.39337778	3.29799795	-0.20022373
C	1.72521281	2.07168293	0.05260282
C	1.94334078	1.40644920	1.32092631
C	2.04159403	3.93606281	-1.41230392
C	1.11330485	3.36969042	-2.26756144
H	0.80152953	3.94150686	-3.13403940
C	0.53962904	2.12217116	-1.94537461
C	-0.51158720	1.34175968	-2.72646976
C	-0.34859434	2.67208815	-4.86919689
H	-0.41036630	2.29067516	-5.90224361
H	0.72547603	2.81509471	-4.67658472
C	-1.13956213	3.98248696	-4.73294067
H	-2.16521525	3.81746840	-5.10862780
H	-1.25791311	4.23872852	-3.66372442
C	-0.49006271	5.13840151	-5.50148392
H	-0.40603489	4.87882853	-6.57311535
H	0.54641795	5.28585148	-5.14091349
C	-1.27208817	6.44614363	-5.33777571
H	-2.29595995	6.34492683	-5.73379707
H	-0.78278136	7.27486181	-5.87347364
H	-1.34954643	6.71967554	-4.27321863
C	-1.93439865	0.76271075	-4.57390785
H	-2.43973780	1.36961532	-5.34272814

H	-2.65840173	0.54818469	-3.77412081
C	-1.40242672	-0.54364932	-5.17342377
H	-0.85888278	-1.09609771	-4.38807535
H	-0.67681229	-0.31534874	-5.97649717
C	-2.53446150	-1.41585493	-5.72804546
H	-3.23409247	-1.65927827	-4.90804958
H	-3.11253715	-0.84267795	-6.47700739
C	-2.01740003	-2.71458864	-6.35457373
H	-1.32678044	-2.50768399	-7.18941069
H	-2.84648371	-3.32578540	-6.74492741
H	-1.47396410	-3.31892490	-5.60974169
C	0.40776867	-1.53233421	2.80237865
C	1.98582423	-2.95387959	4.12225819
H	2.72989798	-2.24447370	3.73270464
H	2.26424336	-3.94052362	3.71009445
C	2.02199721	-3.00185037	5.65298891
H	1.71263325	-2.02352524	6.06572628
H	1.27981925	-3.73491621	6.01630974
C	3.41306591	-3.37483120	6.17949629
H	4.15242147	-2.63862276	5.81409025
H	3.71244717	-4.34947920	5.75327826
C	3.46657515	-3.44316149	7.70877600
H	3.20002103	-2.47327161	8.16019154
H	4.47527599	-3.71159887	8.05925465
H	2.76315546	-4.19801998	8.09686470
C	-0.35893965	-3.67979503	3.56029582
H	0.13853416	-4.60153389	3.90133953
H	-0.67593461	-3.82442594	2.51536059
C	-1.56526005	-3.36410999	4.45194435
H	-2.00682950	-2.40828109	4.12359285
H	-1.21940768	-3.22941327	5.49194813

C	-2.63762736	-4.46527529	4.40020990
H	-2.18472552	-5.43538904	4.67944288
H	-3.38772345	-4.24315214	5.17898226
C	-3.34329081	-4.59060717	3.04427481
H	-3.78037262	-3.62652063	2.73485875
H	-4.15774441	-5.33037519	3.09433365
H	-2.65854168	-4.91245413	2.24325728
O	-1.06578147	3.74170566	0.44208857
O	-0.99606836	5.25438643	-1.14875019
O	-2.05939054	3.36503005	-1.49654007
N	-1.39076209	4.13926744	-0.75940001

L2*Er(NO_3)$_2^+$

E= -16111.151042, E0 = -16110.477200, G= 363.6982 at T=298.150 K

Cl	-5.28872776	3.79429841	-0.59394646
Cl	-4.07823849	-2.98460579	-3.87254524
N	-1.53497565	1.36864948	0.02944658
N	-0.22183762	4.02839231	2.05584049
N	-1.09609163	-0.97771293	-1.14408302
N	1.20671892	-3.64689016	-1.81512046
O	0.09902575	1.79129851	1.97511506
O	0.87157285	-2.25779748	-0.05919808
C	-1.69850624	2.54149222	0.65233672
C	-2.86467290	3.30757737	0.47543001
H	-3.03677154	4.22327948	1.03604913
C	-3.84720922	2.85574579	-0.39466402
C	-3.68216491	1.63426769	-1.09632170
C	-4.63247538	1.07222176	-2.00324464
H	-5.54794264	1.62860656	-2.21039963
C	-4.41428518	-0.14262551	-2.59336281
H	-5.15467787	-0.56469947	-3.27472019
C	-3.22569871	-0.88984472	-2.32808924

C	-2.93891335	-2.17880368	-2.84735990
C	-1.74915540	-2.81834984	-2.52835011
H	-1.57020783	-3.82480168	-2.90216136
C	-0.82651240	-2.16878605	-1.68862259
C	-2.24587560	-0.35027573	-1.45319998
C	-2.48062539	0.92893910	-0.82153690
C	-0.55267853	2.80841875	1.61082602
C	0.48417658	-2.72285008	-1.16590261
C	-0.75272638	5.31265402	1.56261873
C	-1.73983800	5.98528385	2.52314782
H	-1.19895124	5.16100788	0.56933415
C	0.91200036	4.11996794	3.00959730
H	0.85479718	3.25196838	3.68209863
H	0.74919599	5.03230572	3.60491371
C	2.41826200	-4.16189528	-1.12479198
H	2.95349765	-3.29987979	-0.69502127
H	3.05395126	-4.60939884	-1.90394795
C	1.01208520	-4.06217146	-3.21706128
H	0.33687434	-3.34751153	-3.70879698
C	0.51793885	-5.50268555	-3.38398767
Er	0.25691706	-0.18238214	0.78042293
N	-1.11825848	-1.47893345	2.80462646
O	0.16575631	-1.24348450	2.82545257
O	-1.71867895	-1.04364657	1.72603250
O	-1.69762754	-2.04522252	3.69498229
N	2.48798609	0.88796151	-0.47920540
O	3.46561742	1.35594153	-1.00759912
O	2.51763272	0.32870299	0.70056748
O	1.30428755	0.89769471	-1.03344643
H	0.12124097	5.96738243	1.40645027
C	2.27568316	4.16462708	2.31066799

C	-2.25932670	7.31592178	1.96341300
H	-2.58676171	5.30642748	2.73452735
H	-1.24716520	6.16041660	3.49555016
C	-3.23264003	8.01275539	2.91890383
H	-1.40206552	7.98104572	1.75515723
H	-2.75494647	7.13801908	0.99093926
H	-3.58998585	8.96235085	2.49266291
H	-4.11346912	7.38222885	3.12281775
H	-2.74953985	8.23782825	3.88339686
C	3.42216969	4.30251455	3.32045960
H	2.41025019	3.23742819	1.72782791
H	2.30541587	5.00859642	1.59715843
C	4.79352713	4.33617926	2.63921738
H	3.27837515	5.22027016	3.91979814
H	3.38202667	3.45504475	4.02846146
H	5.59921169	4.43179369	3.38327861
H	4.97241688	3.41280746	2.06424761
H	4.87351894	5.18819904	1.94421029
C	2.10865259	-5.18682146	-0.02795298
H	1.99106431	-3.94551277	-3.71371150
C	0.32046837	-5.86467266	-4.86185741
H	1.24525535	-6.19646311	-2.92751288
H	-0.42804906	-5.64554739	-2.82885242
C	-0.15886931	-7.30680799	-5.05246353
H	-0.40621239	-5.16695499	-5.31786823
H	1.27248919	-5.71468544	-5.40208912
H	-0.29378644	-7.53866386	-6.11977911
H	0.56929725	-8.02485657	-4.64205217
H	-1.12278652	-7.47812939	-4.54598475
C	3.39396524	-5.67338133	0.65417910
H	1.44598889	-4.72429371	0.72266728

H 1.56675279 -6.04515505 -0.46304289

C 3.11543703 -6.70208406 1.75416708

H 4.07088375 -6.11082554 -0.10255290

H 3.92472148 -4.80512524 1.08491957

H 4.05190086 -7.03414631 2.22801161

H 2.47251582 -6.27601814 2.54167104

H 2.60840487 -7.59311247 1.34880710

L2*Tm(NO_3)$_3$(H_2O)

(H_2O in outer coordination sphere)

E= -16949.610280, E0 = -16948.898417, G= 380.8708 at T=298.150 K

Cl -5.41574669 3.69701004 -0.44276237

Cl -4.25915718 -3.07688260 -3.75532436

N -1.60530388 1.30902839 0.04729159

N -0.25469095 4.02373028 1.95901179

N -1.20741487 -1.05771458 -1.08866608

N 1.06997359 -3.71164131 -1.70490003

O 0.02332109 1.77568734 2.01851439

O 0.48168379 -2.60708714 0.19159801

C -1.75973165 2.47995114 0.66010982

C -2.93958569 3.23744345 0.53120518

H -3.09291363 4.15453291 1.09686887

C -3.94666791 2.77030563 -0.29383817

C -3.79769778 1.54964995 -0.99323702

C -4.77584648 0.97257203 -1.85830212

H -5.70499277 1.51845884 -2.02923155

C -4.57068062 -0.24432114 -2.44738984

H -5.33260727 -0.67975014 -3.09578705

C -3.36978316 -0.98316628 -2.21752191

C -3.09467340 -2.27177906 -2.73757505

C -1.91032088 -2.92004895 -2.42934275

H -1.72716463 -3.92864490 -2.79962015

C	-0.98031771	-2.26778817	-1.59904552
C	-2.35947585	-0.42999801	-1.38055325
C	-2.57798243	0.85852438	-0.75780886
C	-0.59702295	2.76885295	1.58944297
C	0.26325753	-2.89312553	-1.00134110
C	-0.70165420	5.26062632	1.30559289
C	-1.61016476	6.13746166	2.17632794
H	-1.19262302	5.00584364	0.35485756
C	0.87602186	4.15241766	2.90539336
H	0.79752487	3.32133770	3.62166476
H	0.73250234	5.09836912	3.45375133
C	2.25862050	-4.23044014	-0.98743105
H	2.68323445	-3.39716053	-0.40122840
H	2.98928571	-4.52319384	-1.75796902
C	1.04286313	-3.85000253	-3.16603971
H	0.31425792	-3.13449049	-3.57469749
C	0.74097580	-5.27108526	-3.65194130
Tm	0.52010149	-0.19398677	0.69348127
N	-0.83710122	-1.20131302	2.97504449
O	0.43871003	-1.10855424	2.90388560
O	-1.46684110	-0.86939400	1.87488103
O	-1.43548739	-1.55944479	3.97191334
N	3.24760270	-0.25834420	1.51365769
O	2.49793530	0.80702972	1.49554682
O	2.65002966	-1.32862186	1.13613594
O	4.41443300	-0.22302122	1.86113429
N	1.58871698	0.78187090	-1.76053298
O	2.03353453	1.20064723	-2.81294107
O	1.69330728	-0.45869017	-1.40019763
O	0.96727061	1.53686440	-0.91388893
H	0.20668560	5.82765865	1.03464329

C	2.24664736	4.11318684	2.22075129
C	-2.04284477	7.41411924	1.44540536
H	-2.50147581	5.56391859	2.49110007
H	-1.08083189	6.40684223	3.10729575
C	-2.93642998	8.31293201	2.30565572
H	-1.14376462	7.97505665	1.13175344
H	-2.57539535	7.14113903	0.51576346
H	-3.23342514	9.22044182	1.75719762
H	-3.85623026	7.78601503	2.60871482
H	-2.41409826	8.62946701	3.22341657
C	3.38743377	4.18407679	3.24302030
H	2.33196378	3.17053008	1.65673065
H	2.33731532	4.94707394	1.50009155
C	4.76280689	4.03132105	2.58535671
H	3.33569336	5.13695669	3.80311990
H	3.25017214	3.37415457	3.98244834
H	5.56998110	4.09806681	3.33204532
H	4.84202719	3.05258775	2.08510041
H	4.93428993	4.81702375	1.83016527
C	1.94811738	-5.41178179	-0.06344867
H	2.02997446	-3.52529645	-3.54213595
C	0.74572492	-5.36476946	-5.18237734
H	1.48911464	-5.96939087	-3.23631763
H	-0.23865058	-5.59980297	-3.25756311
C	0.45799300	-6.78151560	-5.68877506
H	-0.00281691	-4.66341209	-5.59460831
H	1.72665393	-5.02655315	-5.56267071
H	0.46716076	-6.82016802	-6.78925323
H	1.21322989	-7.49507523	-5.32029152
H	-0.52963006	-7.13325214	-5.34734869
C	3.20281816	-5.87756777	0.68486989

H 1.18439710 -5.09528112 0.66669583

H 1.52376115 -6.24823713 -0.64860761

C 2.92412925 -7.05749893 1.62090671

H 3.98672700 -6.15725279 -0.04364133

H 3.60416126 -5.02893686 1.26708770

H 3.83854508 -7.37111998 2.14891863

H 2.17299676 -6.78719759 2.38125706

H 2.54048419 -7.92837667 1.06309152

O -4.02888632 0.31540459 2.12388992

H -3.19513822 -0.20989102 2.12042642

H -4.28290176 0.29587948 3.06192350

(NO_3^- in outer coordination sphere)

E= -16949.607591, E0 = -16948.895368, G= 382.2292 at T=298.150 K

Tm -0.50220597 -0.50941277 -0.07213591

Cl 3.86328220 1.95699048 4.83908510

Cl 2.71573949 5.50768423 -1.79941535

O -1.07198811 0.41539568 -2.09461856

O -0.66319036 -1.41360545 2.15592527

O -2.82420135 -0.90998060 0.10630509

O -3.79249287 -2.73776245 -0.66029876

O -1.61943173 -2.43231058 -0.88024837

O 1.08246243 -2.32403564 0.23192306

O 1.13278604 -1.25016451 -1.66408181

O 2.50381947 -2.94717550 -1.33450449

O -1.55724096 1.35305178 0.89227009

H -1.44347537 2.36522007 0.65394551

H -2.51757216 1.17600965 0.86160541

N 1.23628950 0.30045930 1.56044436

N 0.66962534 -2.61047935 3.54658651

N 0.83326787 1.53802848 -0.77027202

N -0.85417140 1.61043751 -3.98325610

N	-2.79683852	-2.06394434	-0.49017102
N	1.61733675	-2.21165848	-0.94333047
C	1.34716046	-0.33469209	2.72716331
C	2.14873409	0.16742007	3.76857305
H	2.18569136	-0.31849197	4.74293375
C	2.87139964	1.32985580	3.55087304
C	2.79301214	2.01521993	2.31315899
C	3.47440171	3.23083901	2.00104642
H	4.14808941	3.65777731	2.74537134
C	3.27107239	3.86239982	0.80543357
H	3.77879357	4.80248308	0.58391613
C	2.37120199	3.33366036	-0.16943473
C	1.70597625	2.10504746	0.08058980
C	1.92382801	1.43465817	1.34690511
C	2.02152491	3.96921587	-1.38335264
C	1.10017049	3.39809084	-2.24424887
H	0.79253060	3.96739554	-3.11418748
C	0.52956837	2.14921904	-1.92156148
C	-0.52105951	1.36343312	-2.70068479
C	-0.31808642	2.65826774	-4.86040974
H	-0.35628834	2.25532365	-5.88645887
H	0.75114530	2.80333304	-4.64436722
C	-1.10788763	3.97338676	-4.77356863
H	-2.12950706	3.79921484	-5.15586662
H	-1.23712850	4.26645803	-3.71533823
C	-0.44560775	5.10251522	-5.57073975
H	-0.34294203	4.80446434	-6.63063097
H	0.58417588	5.26326275	-5.19728899
C	-1.23091686	6.41451597	-5.46898413
H	-2.24711299	6.29823780	-5.88029528
H	-0.73164159	7.22370625	-6.02487230

H	-1.32879627	6.72710562	-4.41695213
C	-1.92028427	0.76349443	-4.56029224
H	-2.41561747	1.36185431	-5.34227943
H	-2.65424514	0.55775237	-3.76744962
C	-1.37823367	-0.54916900	-5.13682699
H	-0.84706557	-1.09199429	-4.33627224
H	-0.63847482	-0.32959828	-5.92929554
C	-2.49974680	-1.42782557	-5.70232964
H	-3.22467613	-1.64517367	-4.89707232
H	-3.05295444	-0.86917734	-6.48053885
C	-1.97496891	-2.74413443	-6.28400612
H	-1.26145387	-2.56087995	-7.10494566
H	-2.79727173	-3.35978913	-6.68166828
H	-1.45466900	-3.33397794	-5.51156759
C	0.39660871	-1.51482856	2.80932307
C	1.99254477	-2.95521474	4.08435440
H	2.72835445	-2.23159623	3.70534897
H	2.27420902	-3.93000627	3.64691186
C	2.04167533	-3.03757191	5.61318779
H	1.73329854	-2.06939721	6.04995108
H	1.30422318	-3.78061032	5.96577883
C	3.43780470	-3.41865063	6.12003946
H	4.17261124	-2.67300367	5.76471472
H	3.73567271	-4.38306713	5.67015123
C	3.50428081	-3.52027726	7.64696598
H	3.23959231	-2.56102347	8.12163544
H	4.51635790	-3.79422140	7.98323727
H	2.80548525	-4.28485632	8.02426338
C	-0.35115501	-3.68394732	3.51824903
H	0.15381926	-4.61043072	3.83459949
H	-0.66846865	-3.80406475	2.47013307

C -1.55927050 -3.40131259 4.41840649

H -1.99983072 -2.43384719 4.12470293

H -1.21653664 -3.30610704 5.46385336

C -2.63212895 -4.49910021 4.32026052

H -2.17898941 -5.48069334 4.55594683

H -3.38109231 -4.31157827 5.10923338

C -3.33973718 -4.56418896 2.96097612

H -3.78173518 -3.58905292 2.69690681

H -4.15129757 -5.30874777 2.97788763

H -2.65589619 -4.84567976 2.14426684

O -1.12562323 3.83177423 0.40524787

O -1.07791686 5.28467321 -1.23648512

O -2.14458776 3.38696980 -1.49986529

N -1.46591127 4.18220663 -0.79811990

L2*Tm(NO_3)$_2^+$

E= -16592.653060, E0 = -16591.979377, G= 363.6854 at T=298.150 K

Cl -5.31276226 3.77963805 -0.56627053

Cl -4.07694292 -2.97838736 -3.89344788

N -1.54969597 1.36232519 0.02824079

N -0.22502244 4.00827074 2.05605578

N -1.10637963 -0.97273088 -1.15263963

N 1.20887625 -3.63207984 -1.81924009

O 0.08862333 1.77012801 1.96904933

O 0.87187225 -2.23034978 -0.07487316

C -1.71359313 2.53010774 0.65797490

C -2.88258362 3.29477143 0.49200273

H -3.05447412 4.20778227 1.05819798

C -3.86711621 2.84477258 -0.37752911

C -3.70089364 1.62818813 -1.08812916

C -4.65019751 1.06707668 -1.99716997

H -5.56857586 1.62035489 -2.19989920

C	-4.42755270	-0.14329495	-2.59569740
H	-5.16738653	-0.56347311	-3.27889204
C	-3.23574424	-0.88813388	-2.33672786
C	-2.94199276	-2.17292500	-2.86266899
C	-1.74943161	-2.80914927	-2.54595065
H	-1.56690741	-3.81291747	-2.92515707
C	-0.83035195	-2.15917039	-1.70181251
C	-2.25801086	-0.34967476	-1.45946896
C	-2.49658513	0.92539430	-0.82098788
C	-0.56230456	2.79074478	1.61072540
C	0.48348427	-2.70478201	-1.17816234
C	-0.75009960	5.29429626	1.56190526
C	-1.72432780	5.98025417	2.52658391
H	-1.20233929	5.14126968	0.57110524
C	0.91079038	4.09483337	3.00780129
H	0.85127354	3.22673368	3.68002391
H	0.75212961	5.00699663	3.60452795
C	2.42243314	-4.13741589	-1.12549138
H	2.95565677	-3.26995993	-0.70407820
H	3.05873823	-4.59009790	-1.90112925
C	1.01609600	-4.05803204	-3.21826720
H	0.34103352	-3.34745431	-3.71596336
C	0.52446306	-5.50028038	-3.37578583
Tm	0.24653295	-0.18738450	0.78076261
N	-1.16885567	-1.49274886	2.77440786
O	0.11583429	-1.26339126	2.80774760
O	-1.75586498	-1.04862356	1.69773579
O	-1.75582349	-2.06333637	3.65802097
N	2.44767737	0.90746838	-0.51381469
O	3.41958714	1.38160646	-1.04906702
O	2.49186563	0.35100216	0.66674620

O	1.26344597	0.90669614	-1.05592859
H	0.12717496	5.94325686	1.39831495
C	2.27410150	4.13548422	2.30818534
C	-2.22339034	7.32031345	1.97054684
H	-2.58194208	5.31565619	2.74071193
H	-1.22551000	6.14650488	3.49739957
C	-3.18999004	8.02716827	2.92541909
H	-1.35582662	7.97380638	1.76826680
H	-2.71769476	7.15423441	0.99541545
H	-3.53033900	8.98459721	2.50279522
H	-4.08168602	7.40956736	3.12148833
H	-2.70817900	8.23923683	3.89350057
C	3.42102265	4.26942110	3.31806374
H	2.40588284	3.20826411	1.72465920
H	2.30635381	4.97977400	1.59521210
C	4.79250479	4.29999590	2.63697839
H	3.27965474	5.18702412	3.91822958
H	3.37851310	3.42155957	4.02549362
H	5.59858894	4.39166784	3.38106203
H	4.96821833	3.37712598	2.06021070
H	4.87493849	5.15300035	1.94345462
C	2.11704588	-5.15356731	-0.01933708
H	1.99571598	-3.94379783	-3.71424556
C	0.33674794	-5.87513494	-4.85173082
H	1.24998605	-6.18910265	-2.90900612
H	-0.42469645	-5.63947010	-2.82522392
C	-0.13795336	-7.31994772	-5.03339100
H	-0.38858902	-5.18292379	-5.31813288
H	1.29174948	-5.72760868	-5.38740253
H	-0.26496008	-7.56110907	-6.09961605
H	0.58916926	-8.03268528	-4.61198378

H -1.10487735 -7.48940945 -4.53208447

C 3.40441585 -5.63204098 0.66450161

H 1.45459497 -4.68630743 0.72856343

H 1.57611382 -6.01663208 -0.44622880

C 3.12986660 -6.65207386 1.77357197

H 4.08062744 -6.07471609 -0.08981899

H 3.93483138 -4.75957966 1.08704519

H 4.06788874 -6.97868681 2.24813747

H 2.48792267 -6.22055912 2.55892038

H 2.62340498 -7.54722261 1.37668872

L2*Yb(NO_3)$_3$(H_2O)

(H_2O in outer coordination sphere)

E= -17442.380461, E0 = -17441.668677, G= 381.0711 at T=298.150 K

Cl -5.38248968 3.71172810 -0.46210477

Cl -4.24013758 -3.06666422 -3.76432014

N -1.57526898 1.32169008 0.02901117

N -0.23981678 4.02341175 1.97682178

N -1.17322421 -1.04030478 -1.12230897

N 1.07261395 -3.73578811 -1.70313466

O 0.06615537 1.77792919 1.98590243

O 0.51487756 -2.58255696 0.17385004

C -1.73072374 2.48959041 0.64796954

C -2.91031194 3.24729633 0.51793462

H -3.06784630 4.16037083 1.08854187

C -3.91377568 2.78461075 -0.31397060

C -3.76235056 1.56798148 -1.01982772

C -4.73983717 0.99421859 -1.88794529

H -5.66743803 1.54227364 -2.06004357

C -4.53752041 -0.22387581 -2.47538424

H -5.30053377 -0.65840322 -3.12302804

C -3.33908916 -0.96590012 -2.24302149

C	-3.07081008	-2.26011729	-2.75307989
C	-1.89158952	-2.91402435	-2.43718600
H	-1.71696424	-3.92848730	-2.79527831
C	-0.95467836	-2.25870275	-1.61690629
C	-2.32558990	-0.41159683	-1.41122913
C	-2.54350281	0.87532187	-0.78490025
C	-0.56819510	2.77285004	1.58016145
C	0.28466251	-2.88754535	-1.01178348
C	-0.70022178	5.27017498	1.35172200
C	-1.62506974	6.11297607	2.23884511
H	-1.18168795	5.03297329	0.39173195
C	0.88472831	4.14599848	2.93129086
H	0.81607538	3.29610372	3.62619901
H	0.72254413	5.07504082	3.50286436
C	2.25232291	-4.26339865	-0.97894776
H	2.69156981	-3.42859435	-0.40591303
H	2.97795129	-4.58077383	-1.74449444
C	1.04837537	-3.88690972	-3.16305518
H	0.33148432	-3.16480875	-3.58065915
C	0.72796214	-5.30796146	-3.63688302
Yb	0.52135533	-0.16401288	0.62829608
N	-0.85013324	-1.15948331	2.91296983
O	0.42300791	-1.02393532	2.85733581
O	-1.47494614	-0.89821321	1.79446363
O	-1.44870949	-1.49722266	3.91854525
N	3.28505063	-0.10834240	1.33798599
O	2.56542134	0.95188379	1.13576579
O	2.65616941	-1.21533334	1.18487990
O	4.46338463	-0.04445247	1.64651656
N	1.57378626	0.68107170	-1.89160681
O	2.02175927	1.05020571	-2.96304512

O	1.73615110	-0.52195048	-1.44384420
O	0.88969195	1.45902562	-1.11899137
H	0.20190620	5.85587358	1.10081232
C	2.25917125	4.14821196	2.25341034
C	-2.07464314	7.39909172	1.53520513
H	-2.50822139	5.51889610	2.53789735
H	-1.10288000	6.36945105	3.17740440
C	-2.98358893	8.26517296	2.41282058
H	-1.18303585	7.97985888	1.23677397
H	-2.60078907	7.13930893	0.59817028
H	-3.29293180	9.17998409	1.88374436
H	-3.89596224	7.71787357	2.70175958
H	-2.46808982	8.56906509	3.33864665
C	3.39413738	4.22118521	3.28227472
H	2.36689448	3.22069073	1.66817200
H	2.33680367	4.99932480	1.55163491
C	4.77498007	4.11152172	2.62706685
H	3.32024813	5.16040373	3.86240768
H	3.27088928	3.39390922	4.00506115
H	5.57759142	4.17265987	3.37905860
H	4.87500429	3.14968491	2.09852624
H	4.93478012	4.92132187	1.89529335
C	1.92092347	-5.42467022	-0.03695615
H	2.04094410	-3.57924342	-3.53936076
C	0.73857582	-5.41709471	-5.16627407
H	1.46358955	-6.01328230	-3.21079373
H	-0.25824311	-5.61853647	-3.24425673
C	0.43273404	-6.83472490	-5.65948248
H	0.00215796	-4.70932055	-5.58913422
H	1.72599900	-5.09682798	-5.54521751
H	0.44591889	-6.88503408	-6.75941849

H 1.17607284 -7.55512762 -5.28031254

H -0.56123978 -7.16863775 -5.31864595

C 3.16661668 -5.90768290 0.71530235

H 1.16511083 -5.08251381 0.68976408

H 1.47797132 -6.25967789 -0.61012185

C 2.86260509 -7.06643772 1.66991460

H 3.94167590 -6.21661758 -0.01081526

H 3.58968139 -5.06043673 1.28404248

H 3.77083755 -7.39502382 2.19939685

H 2.12110949 -6.76623487 2.42849517

H 2.45515752 -7.93549490 1.12630796

O -4.02128172 0.30945313 2.08382964

H -3.19688272 -0.22982129 2.05466461

H -4.25693607 0.27412954 3.02621913

(NO_3^- in outer coordination sphere)

E= -17442.378963, E0 = -17441.666663, G= 382.0452 at T=298.150 K

Yb -0.49752152 -0.51364905 -0.07193659

Cl 3.64965248 2.14770293 4.92722273

Cl 2.50160646 5.63102388 -1.74830723

O -1.13369441 0.44576195 -2.07125449

O -0.65706825 -1.43973649 2.15016508

O -2.81349969 -0.96931642 0.00158776

O -3.65493250 -2.98121715 -0.33212000

O -1.49190271 -2.61057091 -0.55054557

O 1.33304715 -2.08993483 0.21029221

O 0.93284035 -1.32457304 -1.79110503

O 2.52008080 -2.82808948 -1.49480331

O -1.64623809 1.30762839 0.93288517

H -1.58690476 2.29955196 0.67153382

H -2.59043026 1.06841516 0.85059792

N 1.15226388 0.38412246 1.60155451

N	0.72180861	-2.57111692	3.55114269
N	0.77411914	1.58079827	-0.75206995
N	-0.86420846	1.58920169	-3.98789334
N	-2.70116448	-2.22844815	-0.29756305
N	1.63929451	-2.11832476	-1.04626346
C	1.27648020	-0.25073013	2.76672649
C	2.03863955	0.28297514	3.82226229
H	2.08086538	-0.20451719	4.79567719
C	2.71262145	1.47670376	3.61960816
C	2.63140011	2.15586591	2.37853312
C	3.26962900	3.39736772	2.07624292
H	3.90982866	3.85303187	2.83309197
C	3.06592965	4.01802492	0.87511992
H	3.53863692	4.97807932	0.66209441
C	2.21178007	3.44877028	-0.11834628
C	1.59624374	2.19161677	0.12004913
C	1.80563176	1.53910077	1.39720619
C	1.86343694	4.06603193	-1.34165609
C	0.98141271	3.45662045	-2.21804595
H	0.66503978	4.01128197	-3.09465480
C	0.46025351	2.18477488	-1.90498245
C	-0.56659603	1.37246490	-2.69025803
C	-0.30510926	2.61709237	-4.87264490
H	-0.28612205	2.18241143	-5.88624334
H	0.74896091	2.79241610	-4.60976839
C	-1.12325656	3.91795206	-4.86891842
H	-2.12728071	3.70688391	-5.27799129
H	-1.29495192	4.25392580	-3.82961082
C	-0.45524740	5.02569818	-5.69113636
H	-0.30234078	4.67869377	-6.72972393
H	0.55412382	5.22928715	-5.28494644

C	-1.27405167	6.32094812	-5.68884706
H	-2.26992106	6.15837717	-6.13269424
H	-0.77146292	7.11215734	-6.26707745
H	-1.42159617	6.68740320	-4.66024971
C	-1.89694059	0.71229446	-4.58014011
H	-2.37878942	1.28469837	-5.38949537
H	-2.65021777	0.50821328	-3.80525208
C	-1.31052697	-0.60044932	-5.11202717
H	-0.78617507	-1.11265314	-4.28696108
H	-0.55608213	-0.38158590	-5.89071989
C	-2.39663076	-1.51696348	-5.68645906
H	-3.13541508	-1.73641622	-4.89442348
H	-2.94485712	-0.98713291	-6.48803043
C	-1.82603550	-2.83071923	-6.22957325
H	-1.09932506	-2.64519310	-7.03827763
H	-2.62332749	-3.47519088	-6.63240814
H	-1.30768299	-3.39114833	-5.43429470
C	0.39355755	-1.48357034	2.82267284
C	2.05443573	-2.84280777	4.10622263
H	2.75259542	-2.07421970	3.74502039
H	2.39961886	-3.79522943	3.66507888
C	2.08597016	-2.93737459	5.63495159
H	1.71352684	-1.99387419	6.07576752
H	1.38940632	-3.72675824	5.96937275
C	3.49476576	-3.23895741	6.15990019
H	4.18850374	-2.44684196	5.82309532
H	3.85694885	-4.17899656	5.70565367
C	3.54418802	-3.35224485	7.68669367
H	3.21405768	-2.41589904	8.16606998
H	4.56579828	-3.56753016	8.03651619
H	2.88759422	-4.16179085	8.04548550

C -0.23207703 -3.70354772 3.49462676

H 0.32534108 -4.60307789 3.80065846

H -0.53411829 -3.82448673 2.44201660

C -1.46157622 -3.51015162 4.38954973

H -1.96758664 -2.57473803 4.09699631

H -1.13111115 -3.39432311 5.43687248

C -2.45304418 -4.68132401 4.28330851

H -1.92979705 -5.62845707 4.51479721

H -3.21457791 -4.55297375 5.07215977

C -3.15178084 -4.79010677 2.92250443

H -3.67246342 -3.85269499 2.66671872

H -3.89996052 -5.59860229 2.93093038

H -2.44788742 -5.00499439 2.10300040

O -1.32408679 3.83784127 0.36739483

O -1.30108845 5.27897930 -1.27932191

O -2.35671830 3.37673783 -1.52041137

N -1.67655420 4.17432213 -0.82817471

L2*Yb(NO_3)$_2^+$

E= -17085.422767, E0 = -17084.749394, G= 363.3538 at T=298.150 K

Cl -5.32929707 3.76598859 -0.53570992

Cl -4.08405256 -2.98492312 -3.87137675

N -1.55460119 1.36347187 0.04432033

N -0.23162405 4.01879263 2.06070518

N -1.11103320 -0.97333884 -1.13905740

N 1.20369995 -3.63194704 -1.80298066

O 0.07941451 1.77833772 1.99680197

O 0.85540915 -2.24922919 -0.04335627

C -1.72170365 2.52976322 0.67687356

C -2.89439821 3.28931379 0.51506191

H -3.06811190 4.20051670 1.08382857

C -3.87920499 2.83695841 -0.35298467

C	-3.71000171	1.62239516	-1.06665254
C	-4.66030741	1.05960119	-1.97377467
H	-5.58062077	1.61080325	-2.17325735
C	-4.43671846	-0.14990105	-2.57370806
H	-5.17770767	-0.57140315	-3.25482631
C	-3.24277401	-0.89235866	-2.31774640
C	-2.94828129	-2.17775869	-2.84253907
C	-1.75556254	-2.81340265	-2.52531338
H	-1.57202244	-3.81767488	-2.90266061
C	-0.83603877	-2.16185522	-1.68263638
C	-2.26333785	-0.35133991	-1.44409955
C	-2.50240827	0.92347783	-0.80405170
C	-0.56866097	2.79461622	1.62937319
C	0.47575763	-2.71175694	-1.15317953
C	-0.75686842	5.29893589	1.55272400
C	-1.72387147	5.99966049	2.51418781
H	-1.21653843	5.13393927	0.56726825
C	0.90838748	4.11501217	3.00595975
H	0.85095561	3.25463319	3.68817902
H	0.75512105	5.03459406	3.59257650
C	2.41363382	-4.14632034	-1.11054707
H	2.94475245	-3.28498745	-0.67428851
H	3.05446434	-4.58792448	-1.88886666
C	1.01973248	-4.03662157	-3.20937681
H	0.34090844	-3.32360435	-3.69843531
C	0.53858596	-5.47948265	-3.39180875
Yb	0.23017333	-0.18653125	0.78997082
N	-1.14511633	-1.50698054	2.79519773
O	0.11343934	-1.17763209	2.85970259
O	-1.73073268	-1.14134312	1.69012225
O	-1.71458924	-2.09697795	3.67885137

N	2.47782969	0.83140874	-0.47458819
O	3.47737169	1.25298464	-1.00447476
O	2.46399593	0.38730809	0.75133985
O	1.32264495	0.78198618	-1.07013762
H	0.11989534	5.94469786	1.37415993
C	2.26794457	4.14407825	2.29835391
C	-2.22918057	7.33022261	1.94132376
H	-2.57901335	5.33768177	2.74570918
H	-1.21735787	6.18155289	3.47814536
C	-3.18295336	8.05435371	2.89622498
H	-1.36402035	7.97954512	1.71661842
H	-2.73607850	7.14707422	0.97577631
H	-3.53102493	9.00290298	2.46012473
H	-4.07056665	7.43947744	3.11774397
H	-2.68707371	8.28592968	3.85264778
C	3.42184281	4.29063368	3.29840279
H	2.39445233	3.20800352	1.72797775
H	2.29691935	4.97831535	1.57342160
C	4.78830814	4.31006670	2.60687065
H	3.28598499	5.21665478	3.88673735
H	3.38339496	3.45266581	4.01765871
H	5.60059643	4.41103983	3.34287310
H	4.95847464	3.37907791	2.04163814
H	4.86638165	5.15322208	1.90089476
C	2.10354733	-5.17794609	-0.02026437
H	2.00010586	-3.90766406	-3.70040441
C	0.36237276	-5.83322334	-4.87433958
H	1.26556730	-6.17088938	-2.93114805
H	-0.41306299	-5.63306952	-2.84946895
C	-0.09999227	-7.27870512	-5.08090019
H	-0.36556965	-5.13956738	-5.33434010

H 1.31936646 -5.67006922 -5.40183735

H -0.22019953 -7.50412464 -6.15132952

H 0.63085926 -7.99216604 -4.66726208

H -1.06781840 -7.46351099 -4.58681822

C 3.38740301 -5.66348553 0.66524482

H 1.43548739 -4.72196293 0.72957993

H 1.56668544 -6.03595591 -0.46210790

C 3.10763836 -6.69777775 1.75966179

H 4.06866074 -6.09568548 -0.09053395

H 3.91408706 -4.79558277 1.10166764

H 4.04286528 -7.02935076 2.23619819

H 2.46001196 -6.27721453 2.54625511

H 2.60496640 -7.58844852 1.34823442

L2*Lu(NO_3)$_3$(H_2O)

(H_2O in outer coordination sphere)

E= -17946.583685, E0 = -17945.886268, G= 370.6310 at T=298.150 K

Cl -5.40096951 3.72568989 -0.48241910

Cl -4.21313429 -3.02566624 -3.84313369

N -1.60058439 1.32812321 0.01478831

N -0.24152119 4.01332521 1.96000946

N -1.18311334 -1.01448476 -1.14600492

N 1.08156979 -3.69777298 -1.70859396

O 0.05485684 1.77018344 1.95000577

O 0.49306321 -2.53891587 0.15193282

C -1.75353181 2.49453211 0.63508642

C -2.93147969 3.25532985 0.50439984

H -3.08943391 4.16865206 1.07458258

C -3.93347096 2.79647064 -0.33281717

C -3.77994084 1.58439147 -1.04592550

C -4.74910545 1.01484120 -1.92608464

H -5.67722988 1.56107605 -2.10147643

C	-4.53754330	-0.19741207	-2.52311778
H	-5.29380131	-0.62795419	-3.18130541
C	-3.33833599	-0.93818569	-2.28903222
C	-3.05834007	-2.22439861	-2.81186366
C	-1.87798142	-2.87527752	-2.49250507
H	-1.69576585	-3.88475442	-2.86048365
C	-0.95348930	-2.22514248	-1.65432680
C	-2.33517504	-0.38975218	-1.44119465
C	-2.56328011	0.89066428	-0.80626070
C	-0.58240068	2.76972055	1.55855095
C	0.27942115	-2.85096025	-1.03699768
C	-0.71529585	5.26826477	1.36256373
C	-1.63382053	6.08908367	2.27631545
H	-1.20578659	5.04640436	0.40344921
C	0.90690070	4.12161779	2.88856936
H	0.84496230	3.27209640	3.58452559
H	0.76923633	5.05268240	3.46317625
C	2.25558949	-4.21394491	-0.96410370
H	2.68240213	-3.37188506	-0.39188299
H	2.99127936	-4.53373814	-1.71887767
C	1.06316602	-3.88153744	-3.16490483
H	0.35613352	-3.16155553	-3.60249519
C	0.73299521	-5.30995989	-3.60918140
Lu	0.54525089	-0.16991778	0.64647847
N	-0.79883605	-1.11891019	2.93146324
O	0.46840605	-0.93652225	2.89502573
O	-1.40817833	-0.89344919	1.78879201
O	-1.40943527	-1.46430206	3.92397571
N	3.26310706	-0.24375494	1.40458751
O	2.53861570	0.83887988	1.31136441
O	2.62404060	-1.32500374	1.12664342

O	4.43619442	-0.21444438	1.72504330
N	1.57757401	0.83629882	-1.77969563
O	2.03227735	1.25151706	-2.82752848
O	1.69213235	-0.40723449	-1.40992105
O	0.93488473	1.58235610	-0.94294322
H	0.18181451	5.86203718	1.11320043
C	2.26342201	4.10548449	2.17563176
C	-2.09392357	7.38885689	1.60535359
H	-2.51214170	5.48567820	2.57100081
H	-1.10258782	6.32613659	3.21493125
C	-2.99661493	8.23289394	2.51047778
H	-1.20683753	7.97858858	1.31107974
H	-2.62842488	7.14812851	0.66792268
H	-3.31344962	9.15821362	2.00462270
H	-3.90470576	7.67684507	2.79621029
H	-2.47285628	8.51781464	3.43768096
C	3.42452002	4.15727758	3.17609119
H	2.34273291	3.17672372	1.58795798
H	2.33528972	4.95679712	1.47347379
C	4.78565168	4.01671886	2.48668456
H	3.38443661	5.09878254	3.75605845
H	3.30251908	3.33264756	3.90184736
H	5.60835600	4.07004595	3.21730733
H	4.85319614	3.04715037	1.96728289
H	4.94127655	4.81607246	1.74244726
C	1.91873622	-5.36849642	-0.01571996
H	2.06127405	-3.59227848	-3.54047346
C	0.74769348	-5.45124102	-5.13593626
H	1.46198034	-6.01125145	-3.16563821
H	-0.25667053	-5.60476971	-3.21310067
C	0.43244562	-6.87655354	-5.60022402

H 0.01819678 -4.74684286 -5.57615232

H 1.73884690 -5.14674187 -5.51811314

H 0.44871122 -6.94987965 -6.69880390

H 1.16909575 -7.59442234 -5.20367527

H -0.56509596 -7.19570112 -5.25574827

C 3.15908408 -5.83384705 0.75656199

H 1.15271282 -5.02363920 0.69896662

H 1.48792493 -6.21280622 -0.58460176

C 2.85199499 -6.98339653 1.72112668

H 3.94467211 -6.14563322 0.04308714

H 3.56873488 -4.97639275 1.31959748

H 3.75647855 -7.29942703 2.26444364

H 2.10033321 -6.67923355 2.46805739

H 2.45631957 -7.86165857 1.18358481

O -4.02363634 0.18865563 1.97094250

H -3.16809916 -0.30011269 1.98326015

H -4.31648493 0.11741020 2.89504337

(NO_3^- in outer coordination sphere)

E= -17946.584694, E0 = -17945.871519, G= 383.7764 at T=298.150 K

Lu -0.38925478 -0.54040021 -0.13133764

Cl 3.91386080 1.92051959 4.78502083

Cl 2.76781487 5.45857191 -1.86208224

O -1.03491378 0.37200183 -2.10894871

O -0.58690661 -1.47546387 2.05081415

O -2.66951990 -1.01504111 0.00669881

O -3.56261253 -2.85569096 -0.82415658

O -1.39434981 -2.47358608 -0.98944205

O 1.24503446 -2.27551174 0.17953081

O 1.22517967 -1.22047210 -1.72556877

O 2.64717126 -2.88022757 -1.41371202

O -1.44383252 1.25703216 0.85772145

H	-1.31648588	2.29535818	0.64831203
H	-2.40468216	1.08667028	0.81192130
N	1.29085469	0.26145408	1.50690472
N	0.67919451	-2.62114906	3.54018879
N	0.88487405	1.49377370	-0.81875825
N	-0.88623154	1.59608054	-3.98496509
N	-2.59459686	-2.15512705	-0.61657232
N	1.75306642	-2.16447520	-1.00905371
C	1.39339721	-0.37031639	2.67691803
C	2.19168591	0.13684888	3.71823955
H	2.21838856	-0.33780551	4.69802046
C	2.92472506	1.29215956	3.49593759
C	2.85718536	1.97022414	2.25400305
C	3.54480815	3.18046331	1.93607903
H	4.22222519	3.60680056	2.67726398
C	3.33904099	3.81045723	0.73956448
H	3.84867287	4.74877071	0.51501250
C	2.43114495	3.28563142	-0.22956550
C	1.76655269	2.05878425	0.02438002
C	1.98577189	1.39059448	1.28966415
C	2.06811523	3.92530489	-1.43789458
C	1.12878442	3.36243916	-2.28453684
H	0.80804598	3.93771029	-3.14560056
C	0.55685622	2.11491847	-1.95893621
C	-0.51133031	1.33530927	-2.71801066
C	-0.39210349	2.66787648	-4.85852575
H	-0.47431651	2.29017997	-5.89138889
H	0.68539047	2.81062937	-4.68627787
C	-1.18119586	3.97676086	-4.69940758
H	-2.21334529	3.81378984	-5.05784893
H	-1.28012407	4.22473574	-3.62632298

C	-0.54642808	5.13862801	-5.47129917
H	-0.48170406	4.88722944	-6.54618931
H	0.49630147	5.28448153	-5.12856340
C	-1.32637012	6.44438601	-5.28326702
H	-2.35706115	6.34545183	-5.66170073
H	-0.84730989	7.27768850	-5.82104778
H	-1.38482261	6.70921040	-4.21533680
C	-1.96741462	0.75242740	-4.53840828
H	-2.49147058	1.36014616	-5.29388571
H	-2.67304468	0.53067446	-3.72439003
C	-1.43991661	-0.54849255	-5.15363789
H	-0.87889892	-1.10120165	-4.38069677
H	-0.72965777	-0.31317821	-5.96819496
C	-2.57725167	-1.42371798	-5.69236088
H	-3.26999760	-1.65981174	-4.86441660
H	-3.16251826	-0.85422176	-6.43857908
C	-2.06794024	-2.72618079	-6.31731319
H	-1.38760066	-2.52387571	-7.16158915
H	-2.90152526	-3.33972716	-6.69411707
H	-1.51518917	-3.32641292	-5.57607555
C	0.44326159	-1.55090559	2.75411654
C	1.97610068	-2.95755005	4.14317799
H	2.73725843	-2.26448750	3.75747037
H	2.25760674	-3.95602798	3.76306701
C	1.96790528	-2.96940804	5.67509127
H	1.65442502	-1.97940266	6.05559587
H	1.20922089	-3.68788934	6.03332138
C	3.33994460	-3.34027362	6.25073814
H	4.09605885	-2.62005949	5.88772917
H	3.64241576	-4.32813501	5.85854673
C	3.34909701	-3.36894655	7.78220129

H	3.07886744	-2.38519502	8.20012188
H	4.34468126	-3.63684320	8.16865826
H	2.62754560	-4.10695982	8.16930771
C	-0.35201794	-3.68521595	3.51676726
H	0.12929334	-4.60176659	3.89295149
H	-0.63059247	-3.84800124	2.46352005
C	-1.59045124	-3.35056615	4.35609055
H	-2.00738239	-2.39220858	4.00406885
H	-1.28650010	-3.21348453	5.40878534
C	-2.67293811	-4.43936586	4.26413107
H	-2.24307895	-5.41439199	4.56214428
H	-3.44997549	-4.20681000	5.01285839
C	-3.32648396	-4.55747795	2.88160896
H	-3.73960257	-3.58881235	2.55409360
H	-4.15145540	-5.28700399	2.89976072
H	-2.61556292	-4.88829231	2.10778356
O	-0.99026686	3.71045876	0.46529081
O	-0.96853375	5.24184370	-1.10906363
O	-2.01794100	3.34468722	-1.45775330
N	-1.34311843	4.11840343	-0.72439331

L2*Lu(NO$_3$)$_2$$^+$

E= -17589.630064, E0 = -17588.955848, G= 364.1108 at T=298.150 K

Cl	-5.31990147	3.79573941	-0.59598070
Cl	-4.08503866	-2.95736623	-3.93930101
N	-1.55847538	1.37596738	-0.01325433
N	-0.23429707	4.00838614	2.03612804
N	-1.11661065	-0.95412999	-1.19654679
N	1.19269443	-3.62621975	-1.83872163
O	0.09048594	1.77369308	1.91599882
O	0.85410166	-2.20764351	-0.10926218
C	-1.72036278	2.54179597	0.62226433

C	-2.88964224	3.30616760	0.45882672
H	-3.06145191	4.21678591	1.02882826
C	-3.87533760	2.86009145	-0.41196299
C	-3.71069813	1.64576674	-1.12736535
C	-4.66022062	1.08668220	-2.03755856
H	-5.57864761	1.64032149	-2.23900938
C	-4.43781853	-0.12271463	-2.63856196
H	-5.17799807	-0.54141903	-3.32226276
C	-3.24614692	-0.86849558	-2.38102984
C	-2.95178008	-2.15341854	-2.90718102
C	-1.76007915	-2.79077411	-2.58790517
H	-1.57828045	-3.79507279	-2.96611404
C	-0.84103286	-2.14311409	-1.74250901
C	-2.26874423	-0.33088166	-1.50407135
C	-2.50694275	0.94238079	-0.86338490
C	-0.56594008	2.79531431	1.57506716
C	0.46928614	-2.69035006	-1.20870960
C	-0.76811314	5.29861593	1.56354332
C	-1.74726558	5.96097136	2.53976750
H	-1.21842408	5.16006565	0.56972319
C	0.90629435	4.08621740	2.98334718
H	0.85992551	3.20230627	3.63551593
H	0.74250054	4.98360395	3.60071445
C	2.40177894	-4.12836599	-1.13404214
H	2.93621850	-3.25790143	-0.72062117
H	3.03901362	-4.59293127	-1.90184200
C	1.00403571	-4.06515074	-3.23394465
H	0.33654320	-3.35552692	-3.74323750
C	0.50393081	-5.50588799	-3.37861991
Lu	0.21365815	-0.16912778	0.71880490
N	-0.93587261	-1.47989357	2.81490564

O	0.33415630	-1.19624460	2.75775003
O	-1.61895239	-1.07159483	1.76397955
O	-1.44704294	-2.06075788	3.73541331
N	2.56892943	0.80799794	-0.26651236
O	3.59887791	1.25911832	-0.70006639
O	2.47831893	0.20317081	0.88225335
O	1.42823100	0.89015198	-0.91848797
H	0.10456937	5.95623446	1.41121423
C	2.26363444	4.15423298	2.27443433
C	-2.26169848	7.30327463	2.00345659
H	-2.59726238	5.28439331	2.74683285
H	-1.24761391	6.11866570	3.51151633
C	-3.22733974	7.99013853	2.97380972
H	-1.40134430	7.96672392	1.80297267
H	-2.76143456	7.14436007	1.02984929
H	-3.58178043	8.94786263	2.56369042
H	-4.11037207	7.36095095	3.17222595
H	-2.73874569	8.19806194	3.93934870
C	3.42030096	4.25090599	3.27730131
H	2.39045429	3.24890041	1.65686607
H	2.28950834	5.02302122	1.59147692
C	4.78443766	4.29972649	2.58255267
H	3.28790665	5.14782095	3.90983129
H	3.38205886	3.37890196	3.95481896
H	5.59892988	4.36419678	3.32018113
H	4.95018721	3.39535260	1.97447693
H	4.86249495	5.17476940	1.91651607
C	2.08857870	-5.12919712	-0.01619195
H	1.98679686	-3.96255565	-3.72614694
C	0.32395080	-5.89595985	-4.85157871
H	1.22191179	-6.19381714	-2.89915252

H -0.44968712 -5.63295031 -2.83280802

C -0.15700723 -7.34025145 -5.02052116

H -0.39455920 -5.20521402 -5.33059502

H 1.28331220 -5.75916815 -5.38222933

H -0.27940825 -7.59203863 -6.08479643

H 0.56437021 -8.05190659 -4.58766079

H -1.12740707 -7.49962234 -4.52265501

C 3.37315416 -5.60281134 0.67656142

H 1.42737520 -4.64923382 0.72464573

H 1.54494464 -5.99546909 -0.43311587

C 3.09244633 -6.60345650 1.80159593

H 4.04820919 -6.06037140 -0.06982511

H 3.90606499 -4.72571421 1.08592141

H 4.02821207 -6.92720318 2.28242779

H 2.45228791 -6.15555239 2.57907128

H 2.58169913 -7.50233173 1.41891623

Ligand L3

E= -1220.515792, E0 = -1220.117374, G= 215.1705 at T=298.150 K

C 0.50669205 0.02459001 3.43878412

C 1.65194976 -0.15478058 2.45634866

C 2.94941902 -0.22254448 3.01915288

C 4.02924776 -0.34749103 2.17403865

C 3.82200646 -0.36818823 0.77446008

C 2.47820854 -0.25195736 0.30834275

N 1.42391551 -0.16117065 1.14918780

C -0.68469733 0.00051874 -3.41051316

O -0.94687265 -0.54335201 -4.48569393

N -1.62547255 0.67241019 -2.68815398

C -2.98840904 0.77429807 -3.24336934

C -1.44992077 1.54152274 -1.50394046

N -0.73509741 -0.42177150 3.09669256

H	5.04816818	-0.42520496	2.56525731
C	4.89857864	-0.49543497	-0.15820450
O	0.73250860	0.57666463	4.51799297
C	-1.82659876	-0.26538542	4.07663679
C	-1.13993752	-1.27355349	1.95685780
H	3.04688096	-0.16337749	4.10412169
C	-3.62119865	1.93100297	-2.45962405
C	-2.89870000	-1.25131309	3.59593415
H	-2.19443703	0.77664077	4.06664944
H	-2.93743372	0.94305414	-4.33017063
C	2.23036814	-0.23849554	-1.12788475
N	0.96082073	-0.08456746	-1.56525326
C	3.33590221	-0.38020167	-2.01918149
C	0.73348153	-0.09074555	-2.87252092
C	4.66387749	-0.50653398	-1.50386131
H	5.91745186	-0.58595049	0.23012935
C	3.05575061	-0.39294878	-3.40611219
H	3.87853026	-0.50942796	-4.11788321
C	1.75433242	-0.26687273	-3.83769584
H	5.49091721	-0.61078346	-2.21254206
H	1.47054589	-0.30576456	-4.89037704
H	-3.53143024	-0.17471781	-3.08313370
H	-1.45009196	-0.47031078	5.09075403
C	-2.89220858	1.87693322	-1.10746789
H	-0.87621468	1.02255476	-0.72698718
H	-0.90009797	2.46109080	-1.78134537
C	-2.66784883	-1.30512011	2.07721400
H	-0.76934236	-0.85583246	1.01328051
H	-0.72056228	-2.29136992	2.07093000
H	-2.95513272	2.81654596	-0.53849608
H	-3.30747581	1.06634068	-0.48479947

H -4.71237135 1.82304430 -2.37133956

H -3.41066813 2.88933539 -2.96303487

H -3.10026789 -2.19737029 1.60025954

H -3.09985852 -0.41123211 1.59600663

H -3.91300201 -0.92677683 3.87157226

H -2.72420502 -2.24599981 4.03898191

L3*La$(NO_3)_3(H_2O)$

(H_2O in outer coordination sphere)

E= -10633.323658, E0 = -10632.853809, G= 243.2600 at T=298.150 K

H -4.40244198 3.25868177 -0.64169323

H -3.41307306 -3.00236678 -3.19570446

N -1.28413844 1.12625682 0.25440264

N 0.11966471 3.99112105 1.81493378

N -0.90969127 -1.35276496 -0.72809380

N 1.35881436 -3.98766065 -1.29885447

O 0.22084422 1.80339372 2.35539889

O 0.74133879 -2.97826076 0.63185620

C -1.41721809 2.36206913 0.73398364

C -2.53606534 3.16616511 0.43403706

H -2.64587092 4.15429449 0.88266647

C -3.51367497 2.66600180 -0.40920487

C -3.36803627 1.37955689 -0.96734411

C -4.31427145 0.78734505 -1.86429763

H -5.20459557 1.36056340 -2.13688755

C -4.11770391 -0.46559548 -2.37278461

H -4.84805822 -0.90446639 -3.05818391

C -2.96075296 -1.23409045 -2.02253222

C -2.70119667 -2.53093910 -2.51253557

C -1.55588067 -3.20217490 -2.11697054

H -1.34863412 -4.21248150 -2.47419572

C -0.67702162 -2.57171345 -1.21409404

C	-2.00850558	-0.68615514	-1.11939764
C	-2.21213198	0.64456326	-0.58774304
C	-0.30320626	2.71981955	1.69006324
C	0.53554475	-3.21013093	-0.57701182
C	-0.13202247	5.12530756	0.90324992
H	-0.95863622	5.75325632	1.28456926
C	1.19241536	5.89877892	0.94613802
H	1.91496265	5.42556190	0.26103160
H	1.06496716	6.95156717	0.65399671
C	1.64187205	5.71445799	2.40385652
H	1.07767880	6.39301395	3.06543946
H	2.71468210	5.90551090	2.55087399
C	1.28370357	4.25356960	2.69968009
H	2.09403539	3.55277848	2.43452525
H	1.01262856	4.06190252	3.74927926
C	2.61076951	-4.46421146	-0.65413189
H	2.38411164	-5.25386286	0.07891608
H	3.06398892	-3.62048531	-0.10603948
C	3.47074604	-4.93810749	-1.83296466
C	2.97722363	-4.07140970	-3.00223994
C	1.46139336	-4.03420115	-2.77181602
H	3.23651576	-4.47710419	-3.99123836
H	3.37837148	-3.04824209	-2.91554070
H	3.28826690	-6.00524998	-2.04434252
H	4.54284906	-4.80781698	-1.62542331
H	0.98003703	-3.15418673	-3.22099733
La	0.83872336	-0.46217763	1.29230678
N	-0.96805716	-1.34064329	3.55371356
O	0.29452103	-1.19688201	3.71573591
O	-1.40721273	-1.08877599	2.33716226
O	-1.72797155	-1.68466699	4.43856907

N 3.58087325 -0.61733431 2.45621586

O 2.91281843 0.49729642 2.38420439

O 2.96445417 -1.66243231 1.99473536

O 4.70384836 -0.67272466 2.91961813

N 2.11687970 0.50705326 -1.28090858

O 2.58952880 0.88975298 -2.33803654

O 2.10633969 -0.74841261 -0.94637394

O 1.58814120 1.32107091 -0.42463127

H 0.98229730 -4.95095587 -3.16301823

H -0.38861269 4.76534939 -0.10323061

O -4.03421736 -0.96977693 1.34852934

H -3.18674946 -1.04227376 1.84901178

H -4.50020266 -1.78532612 1.59976244

(NO_3^- in outer coordination sphere)

E= -10633.318784, E0 = -10632.848352, G= 245.6491 at T=298.150 K

H -4.69668531 2.89061546 -0.55803037

H -3.06045365 -3.23533607 -3.09341645

N -1.43171453 1.05112183 0.44338444

N -0.42989889 4.04776287 2.11610460

N -0.78011554 -1.37585735 -0.56420475

N 1.69958365 -3.85004592 -1.23345435

O -0.00780323 1.87785232 2.55906487

O 1.25308287 -2.63121057 0.62163615

C -1.69314098 2.26601577 0.92538214

C -2.87343287 2.96114945 0.58726591

H -3.10021973 3.92626595 1.03984082

C -3.76585841 2.38043833 -0.29600328

C -3.48011088 1.12032199 -0.86163914

C -4.33496523 0.45719248 -1.79907405

H -5.25892162 0.95374733 -2.10697198

C -4.00641537 -0.77023125 -2.30281210

H	-4.66468143	-1.26702905	-3.02059841
C	-2.79844785	-1.43285310	-1.91256917
C	-2.41032624	-2.70112467	-2.39582586
C	-1.20881951	-3.25118351	-1.99554563
H	-0.89680034	-4.22665739	-2.36701918
C	-0.39859053	-2.54187393	-1.08379507
C	-1.92925191	-0.80925018	-0.97970450
C	-2.27562380	0.48895073	-0.44287571
C	-0.65413231	2.73217916	1.92099690
C	0.92065793	-3.02115440	-0.52303910
C	-0.83307308	5.18720722	1.26615894
H	-1.77708447	5.63063049	1.63324344
C	0.32264319	6.18026066	1.44763148
H	1.15318751	5.90986729	0.77502513
H	0.01393161	7.21242523	1.22624874
C	0.72689164	5.95955658	2.91301203
H	0.01278555	6.46390533	3.58511043
H	1.73541641	6.33157635	3.14386487
C	0.63168365	4.43881989	3.07581067
H	1.56878924	3.92556095	2.79992199
H	0.36264125	4.11186314	4.09206200
C	3.01551318	-4.22041941	-0.65718055
H	2.87487721	-4.86275434	0.22628568
H	3.52310801	-3.29816413	-0.32648078
C	3.74830914	-4.89443111	-1.82060528
C	3.16947412	-4.17721987	-3.04834175
C	1.68088627	-4.04632187	-2.70661616
H	3.32601357	-4.72295856	-3.99047923
H	3.58687472	-3.16177750	-3.14002800
H	3.51234913	-5.97163916	-1.85356104
H	4.83879280	-4.78436899	-1.72702336

H 1.22541404 -3.17285299 -3.20342374

La 0.40292335 -0.52967852 1.75472796

N 2.65807152 -0.75194389 3.67935705

O 3.58983350 -0.84540302 4.45099115

O 2.79707003 -0.25040156 2.48118258

O 1.45632243 -1.13700736 3.97999644

O 1.64724851 0.84116006 0.04533392

H 1.37645638 0.77681959 -0.97478098

H 2.60638618 0.65836483 0.06556714

N -2.04959011 -1.70822787 3.00809169

O -1.22287965 -2.41998005 2.30362034

O -1.74765527 -0.44647440 3.11326432

O -3.04210997 -2.18243718 3.52526236

H 1.14009130 -4.97767735 -2.95514321

H -0.96842623 4.86835241 0.22270632

N 1.31762779 -0.51052809 -2.78470755

O 0.70740825 -1.10725427 -3.70130491

O 0.82832462 0.61199808 -2.33971334

O 2.36655807 -0.96825194 -2.26218581

L3*La$(NO_3)_2^+$

E= -10276.351556, E0 = -10275.920018, G= 227.2322 at T=298.150 K

H -4.10866928 3.40019989 -0.92950660

H -3.04102516 -2.82888055 -3.47213149

N -1.12121403 1.20764053 0.22194979

N 0.02636522 4.01343012 2.13759828

N -0.69360214 -1.28440154 -0.79593533

N 1.42521286 -4.15042591 -1.19464278

O 0.26618662 1.78773975 2.39449954

O 1.04838634 -2.75763583 0.53595656

C -1.29434586 2.43395352 0.72862732

C -2.37321115 3.25203133 0.33638731

H	-2.53719330	4.22241402	0.80239320
C	-3.25540423	2.78933191	-0.62356150
C	-3.06326151	1.51887465	-1.20438302
C	-3.92682624	0.97566676	-2.20951509
H	-4.76682568	1.58053052	-2.56040955
C	-3.71281719	-0.27314147	-2.71925712
H	-4.37919331	-0.68094587	-3.48360133
C	-2.61834216	-1.07686150	-2.26401472
C	-2.36879468	-2.38235664	-2.73487449
C	-1.28471708	-3.09562778	-2.25541401
H	-1.10786235	-4.11286163	-2.60125375
C	-0.44726819	-2.50415397	-1.28800809
C	-1.73492968	-0.57121724	-1.26977539
C	-1.96203196	0.75346804	-0.72896570
C	-0.28540567	2.75573230	1.80730450
C	0.73110735	-3.16714644	-0.61196929
C	-0.30438423	5.27897978	1.43985784
H	-1.26099467	5.68032265	1.81948185
C	0.85199142	6.20470524	1.83753121
H	1.72078001	6.01898718	1.18507302
H	0.57135433	7.26409292	1.75180471
C	1.15512359	5.76589870	3.27736950
H	0.40645963	6.18160582	3.97125673
H	2.15115857	6.07757187	3.62168336
C	1.03270125	4.24107599	3.21131921
H	1.97906888	3.75652552	2.91597629
H	0.69286066	3.77065301	4.14620590
C	2.57262421	-4.74826145	-0.45743021
H	2.22433901	-5.16356230	0.50017655
H	3.30144429	-3.95077491	-0.23275627
C	3.12950683	-5.78788376	-1.43385017

C 2.79974580 -5.18937159 -2.80877852

C 1.39005888 -4.62243080 -2.59960532

H 2.82387424 -5.92551804 -3.62481117

H 3.49862289 -4.37284470 -3.05342793

H 2.61428714 -6.75320578 -1.30153203

H 4.20567989 -5.95069981 -1.28179312

H 1.14786255 -3.79565310 -3.28256583

La 0.55218887 -0.50521076 1.47059381

N -1.41602159 -1.61648047 3.36703086

O -0.14041120 -1.50565302 3.63543844

O -1.76673853 -1.19340658 2.17958903

O -2.20737839 -2.07332087 4.15365648

N 3.19452691 0.36771548 0.46668297

O 2.10955834 0.48177344 -0.25447902

O 3.01897597 -0.15623216 1.65001655

O 4.27866697 0.72096592 0.06772143

H 0.63751686 -5.42354965 -2.70988154

H -0.37936839 5.11782980 0.35494775

L3*Ce(NO_3)$_3$(H_2O)

(H_2O in outer coordination sphere)

E= -11000.743712, E0 = -11000.274750, G= 243.7494 at T=298.150 K

H -4.37681055 3.29184222 -0.65753949

H -3.28175306 -2.92088914 -3.32264924

N -1.21526372 1.20165241 0.17144923

N 0.04982640 4.04125786 1.95453119

N -0.79736328 -1.25539315 -0.83225995

N 1.32151568 -4.11520958 -1.21148539

O 0.43522659 1.82950664 2.10061288

O 0.89825803 -2.72901678 0.52185041

C -1.36905563 2.42405486 0.69335395

C -2.51530647 3.20465803 0.41854402

H	-2.66347432	4.16779613	0.90597826
C	-3.47420025	2.71253371	-0.44567797
C	-3.30269933	1.44688904	-1.05067062
C	-4.22581244	0.86333567	-1.96192336
H	-5.12394619	1.42476726	-2.23248482
C	-4.00205755	-0.38642392	-2.48978353
H	-4.72196388	-0.82521814	-3.18630815
C	-2.84717417	-1.14514446	-2.14184999
C	-2.57734656	-2.44780946	-2.63372111
C	-1.44244719	-3.11895132	-2.21732140
H	-1.25017464	-4.13628292	-2.56057405
C	-0.56236964	-2.49755478	-1.30902064
C	-1.90106058	-0.59790468	-1.22877991
C	-2.13017750	0.72140288	-0.68164784
C	-0.24827828	2.76894736	1.62360990
C	0.60115439	-3.13427186	-0.63455802
C	-0.37572780	5.28644085	1.28564715
H	-1.27158606	5.70686626	1.77916515
C	0.82916754	6.21625805	1.48363245
H	1.58617651	6.00900078	0.70942152
H	0.54430264	7.27686310	1.42144632
C	1.35405385	5.79969931	2.86576056
H	0.72404480	6.23444080	3.65970993
H	2.39376211	6.10989952	3.04376101
C	1.21422672	4.27422523	2.84554148
H	2.09850907	3.77147555	2.41729903
H	1.02867329	3.82736635	3.83426857
C	2.50603032	-4.63592577	-0.48368943
H	2.18820763	-5.20008850	0.40683421
H	3.11220813	-3.78190851	-0.13583454
C	3.22981882	-5.48833132	-1.53263700

C 2.85753965 -4.80529547 -2.85766363

C 1.38007808 -4.44904900 -2.64741087

H 3.00740695 -5.44453192 -3.74026537

H 3.44149041 -3.87913775 -2.98663664

H 2.85211229 -6.52455187 -1.51864457

H 4.31427765 -5.51577044 -1.35252309

H 1.05165303 -3.59306598 -3.25459290

Ce 0.92008507 -0.31070215 0.95630401

N -0.59255695 -1.15986753 3.35321474

O 0.68149406 -0.97854692 3.35616589

O -1.16957474 -0.93649876 2.19812036

O -1.22226095 -1.51389813 4.32824993

N 3.72335768 -0.31590542 1.83969462

O 3.04379916 0.77659845 1.63232660

O 3.05918050 -1.40760005 1.63811111

O 4.88517141 -0.30347922 2.19023490

N 2.10047984 0.55293185 -1.61599255

O 2.59556246 0.90896934 -2.66663265

O 2.21643376 -0.66093493 -1.16596866

O 1.41104841 1.35000968 -0.86244142

H 0.73562145 -5.31951523 -2.87245989

H -0.60416710 5.09956264 0.22651938

O -3.89806724 -0.93616831 1.37377274

H -3.02588677 -0.97061294 1.82751644

H -4.20339108 -1.85779572 1.42900014

(NO_3^- in outer coordination sphere)

E= -11000.736938, E0 = -11000.269123, G= 245.2978 at T=298.150 K

H -4.78746080 2.93525434 -0.45032835

H -3.15612912 -3.14882779 -3.15215182

N -1.44553149 1.12353241 0.36957580

N -0.48222628 4.11069059 2.17546940

N	-0.83973849	-1.29287338	-0.65990347
N	1.63689530	-3.80466962	-1.24866629
O	0.29370081	2.00383639	2.10641265
O	1.21748996	-2.42839932	0.48566243
C	-1.69726908	2.35284781	0.88096952
C	-2.91578531	3.01365209	0.60818344
H	-3.15107107	3.96268106	1.08652163
C	-3.83727837	2.43680477	-0.24280830
C	-3.55423331	1.18570411	-0.85244566
C	-4.42184782	0.53187001	-1.77277935
H	-5.35988903	1.02404106	-2.04378629
C	-4.10106134	-0.69107187	-2.31602693
H	-4.77929783	-1.17787647	-3.02157664
C	-2.88740420	-1.34778416	-1.96796215
C	-2.49587369	-2.61415195	-2.46424985
C	-1.29223895	-3.17027140	-2.08040857
H	-1.00090086	-4.15100765	-2.45278454
C	-0.46377853	-2.46801686	-1.17655730
C	-1.99626803	-0.72710085	-1.04405260
C	-2.32781005	0.56497955	-0.48142636
C	-0.59053677	2.83089089	1.75236142
C	0.85119164	-2.92450237	-0.61642337
C	-1.15650070	5.32053995	1.66717768
H	-2.11013746	5.48918200	2.20111132
C	-0.16058879	6.44279051	1.99240029
H	0.59930098	6.50704145	1.19629836
H	-0.65851319	7.41957235	2.07972169
C	0.47889844	5.95868254	3.30156136
H	-0.20510438	6.13492537	4.14837408
H	1.43606544	6.45160627	3.52424884
C	0.65369427	4.45665741	3.06298804

H	1.60274243	4.22271681	2.54956698
H	0.61479765	3.84699035	3.97869539
C	2.96492577	-4.11357784	-0.65676868
H	2.83924770	-4.50615215	0.36317042
H	3.53905797	-3.17263031	-0.60824841
C	3.58156872	-5.10081387	-1.65014827
C	2.98543167	-4.65302896	-2.99145770
C	1.53110969	-4.32064247	-2.63450503
H	3.04339981	-5.42088842	-3.77685428
H	3.46636677	-3.72942495	-3.35151315
H	3.27518320	-6.13304043	-1.40960944
H	4.68002462	-5.05314112	-1.63209462
H	1.11978960	-3.55023956	-3.30264282
Ce	0.45427093	-0.32532194	1.43842065
N	2.74448180	-0.69469094	3.21122861
O	3.69933724	-0.86934674	3.93402243
O	2.81665659	-0.01356956	2.10284925
O	1.56318545	-1.16666186	3.47600651
O	1.68500769	0.57326871	-0.38883540
H	1.58455396	0.23059507	-1.45504189
H	2.64899874	0.50840813	-0.22937250
N	-1.77284443	-1.24091327	3.12655282
O	-1.25227869	-1.96488893	2.17569470
O	-1.27179492	-0.05052297	3.23386741
O	-2.65925932	-1.64998138	3.84463024
H	0.89999264	-5.22789764	-2.64971995
H	-1.35992193	5.23036861	0.58988541
N	2.45244908	-1.01070619	-2.97798681
O	2.41477728	-1.64529753	-4.04599953
O	1.42327547	-0.22315802	-2.70096540
O	3.39270473	-1.08703983	-2.15752697

L3*Ce(NO$_3$)$_2^+$

E= -10643.772031, E0 = -10643.341444, G= 225.9392 at T=298.150 K

H -4.08151007 3.42509866 -0.95274878

H -3.00676990 -2.81676817 -3.50926137

N -1.09072149 1.21191061 0.18067843

N 0.04468916 3.98767900 2.16636157

N -0.66813868 -1.24950993 -0.82533854

N 1.43818927 -4.14746094 -1.15467978

O 0.37644923 1.76262367 2.25258112

O 1.12777877 -2.61315370 0.46310401

C -1.25581038 2.44946527 0.68574029

C -2.33142734 3.26790261 0.29827714

H -2.48777604 4.24096775 0.76000524

C -3.23119760 2.80754113 -0.65357238

C -3.05324507 1.53394210 -1.22869194

C -3.92481923 0.98360109 -2.22435832

H -4.77043152 1.58428645 -2.56902266

C -3.70975566 -0.26531398 -2.73576355

H -4.38132143 -0.67541671 -3.49438453

C -2.60605812 -1.06488943 -2.29253340

C -2.34138918 -2.36335492 -2.77059746

C -1.24270320 -3.06504345 -2.29310417

H -1.05745327 -4.07808161 -2.64494777

C -0.41048318 -2.47290039 -1.32686973

C -1.72630942 -0.54767060 -1.30333614

C -1.95168340 0.76345909 -0.76707000

C -0.23805629 2.75119758 1.74627757

C 0.76691473 -3.10885167 -0.64852047

C -0.35331535 5.28992939 1.58216000

H -1.32688546 5.60710287 1.99654329

C 0.75871938 6.23594761 2.05216050

H	1.62587321	6.15873146	1.37615919
H	0.42120361	7.28197908	2.06743050
C	1.10776460	5.68523741	3.44209146
H	0.35030028	5.99594021	4.17996168
H	2.09217525	6.01604748	3.80160880
C	1.06187749	4.16858387	3.23844957
H	2.02525616	3.76361251	2.88388228
H	0.76673675	3.59868264	4.13210773
C	2.58076429	-4.71014404	-0.38349271
H	2.23989892	-5.01793766	0.61630505
H	3.34129214	-3.92143846	-0.25179902
C	3.08035564	-5.85934305	-1.26296556
C	2.74613214	-5.38397360	-2.68397212
C	1.36426735	-4.74158955	-2.51007509
H	2.72510266	-6.19629908	-3.42425323
H	3.47180557	-4.62503386	-3.01909041
H	2.53263807	-6.78676414	-1.02920234
H	4.15264511	-6.04757214	-1.11293781
H	1.14697695	-3.96765208	-3.26025248
Ce	0.57178766	-0.41936344	1.26664388
N	-1.25991213	-1.52688122	3.21201587
O	0.03192604	-1.38280272	3.40297627
O	-1.67339110	-1.12144721	2.04354119
O	-1.99057841	-1.99251378	4.04597998
N	3.06381559	0.49115527	0.10057159
O	1.93434763	0.56661242	-0.54885477
O	2.96261358	-0.02414501	1.30063510
O	4.11319304	0.86259741	-0.36010227
H	0.57568675	-5.51450062	-2.54011297
H	-0.42803469	5.21598244	0.48766270

L3*Pr(NO_3)$_3$(H_2O)

(H_2O in outer coordination sphere)

E= -11377.813864, E0 = -11377.344314, G= 243.9662 at T=298.150 K

H	-4.37773466	3.28027821	-0.66526723
H	-3.35062456	-2.97164583	-3.24074864
N	-1.24139524	1.16466844	0.20590524
N	0.08835694	4.01317406	1.89627349
N	-0.84503657	-1.29958498	-0.77704549
N	1.34117985	-4.05942774	-1.23694134
O	0.33389434	1.79765332	2.21626067
O	0.80984378	-2.84756589	0.59722066
C	-1.38516045	2.39384604	0.70318383
C	-2.51275373	3.18990302	0.41193932
H	-2.63882041	4.16829586	0.87576151
C	-3.48348975	2.69274235	-0.44111907
C	-3.32686853	1.41540778	-1.01659989
C	-4.26552105	0.82271737	-1.91710985
H	-5.16017962	1.38932860	-2.18894625
C	-4.05744553	-0.42952245	-2.42835522
H	-4.78475523	-0.87243861	-3.11450911
C	-2.89922953	-1.19349551	-2.07784772
C	-2.64055300	-2.49543834	-2.55955982
C	-1.49931681	-3.16566229	-2.14833355
H	-1.30042374	-4.18277359	-2.48974657
C	-0.61934149	-2.53351474	-1.25046325
C	-1.94822347	-0.63927335	-1.17723751
C	-2.16375279	0.68473780	-0.64391255
C	-0.26800358	2.73736715	1.65342212
C	0.56741130	-3.17238188	-0.58731079
C	-0.24690938	5.22273779	1.11807728
H	-1.11980152	5.73908567	1.55894876
C	1.01363635	6.08597326	1.26278126

H	1.76789677	5.76154709	0.52714807
H	0.80162019	7.15333414	1.10241544
C	1.48562229	5.75892448	2.68768430
H	0.87299418	6.30209446	3.42658877
H	2.54066443	6.01522446	2.86116242
C	1.24571300	4.24952507	2.79626584
H	2.10284019	3.65474200	2.43625069
H	1.00936604	3.90369081	3.81415939
C	2.56048512	-4.55675459	-0.54882956
H	2.28353262	-5.23769712	0.27084765
H	3.08620858	-3.69541192	-0.10223686
C	3.36251831	-5.22854328	-1.67034411
C	2.93160319	-4.45350409	-2.92537785
C	1.42723608	-4.25865936	-2.69729352
H	3.14272904	-4.98463821	-3.86526179
H	3.42409158	-3.46759415	-2.95289922
H	3.08431697	-6.29178143	-1.76419985
H	4.44436407	-5.17242336	-1.48121691
H	1.01994169	-3.38740110	-3.22966123
Pr	0.85940075	-0.37965205	1.08155787
N	-0.76601428	-1.19763494	3.40973759
O	0.50070572	-1.00843084	3.48626351
O	-1.27753603	-1.01044810	2.21413970
O	-1.45669460	-1.52798343	4.35306120
N	3.61633635	-0.44673792	2.06783438
O	2.96772194	0.66365999	1.87746203
O	2.94244289	-1.52103949	1.79793501
O	4.76450062	-0.47374773	2.46378994
N	2.08562827	0.48739699	-1.46942294
O	2.58943820	0.84939963	-2.51686215
O	2.14633012	-0.74171841	-1.05710256

O 1.44165754 1.29831684 -0.69073069

H 0.86647797 -5.16291285 -2.99934435

H -0.47292036 4.96286774 0.07397532

O -3.96804309 -0.95537806 1.34826779

H -3.09900999 -1.00532258 1.81036460

H -4.35314703 -1.83050108 1.52515161

(NO_3^- in outer coordination sphere)

E= -11377.809424, E0 = -11377.339496 , G= 245.8723 at T=298.150 K

H -4.65733767 2.93883538 -0.58688498

H -3.05994654 -3.21563101 -3.11548376

N -1.41084480 1.05791438 0.41955683

N -0.40730691 4.02854156 2.16134167

N -0.78893518 -1.36145663 -0.57427919

N 1.69219232 -3.85310817 -1.20077479

O 0.06100168 1.84736311 2.47601366

O 1.26811206 -2.53614187 0.58859247

C -1.66036808 2.28132606 0.90134287

C -2.83230495 2.98589802 0.56055647

H -3.05062675 3.95324922 1.01175380

C -3.73389935 2.41626382 -0.32419318

C -3.46305871 1.15086496 -0.88628560

C -4.32143736 0.49094620 -1.82418931

H -5.24007177 0.99538994 -2.13559580

C -4.00438738 -0.74084371 -2.32678843

H -4.66576624 -1.23102689 -3.04625058

C -2.80297661 -1.41369176 -1.93338144

C -2.41431642 -2.68164325 -2.41367388

C -1.21299183 -3.23477817 -2.00783062

H -0.90259671 -4.20962477 -2.38144469

C -0.40483391 -2.52946568 -1.09322631

C -1.93593109 -0.79092866 -0.99779415

C	-2.26733708	0.50721806	-0.46707010
C	-0.61661357	2.72241879	1.89408588
C	0.91806763	-2.98750353	-0.53035378
C	-0.84066236	5.20970964	1.38833106
H	-1.79596436	5.60378027	1.78196466
C	0.28848910	6.21937561	1.63450181
H	1.12298667	6.01885319	0.94253260
H	-0.04810183	7.25588036	1.48601270
C	0.70642471	5.90943098	3.07969165
H	-0.01924780	6.34405279	3.78715777
H	1.70486641	6.29352808	3.33333468
C	0.65500617	4.37915373	3.13518620
H	1.60329449	3.91454387	2.81410146
H	0.40780634	3.97214651	4.12771082
C	3.01131487	-4.19284439	-0.61297882
H	2.87498260	-4.77079010	0.31433985
H	3.52959013	-3.25382805	-0.35352397
C	3.72659802	-4.95112276	-1.73433316
C	3.14678812	-4.30830574	-3.00176477
C	1.66217554	-4.13786983	-2.65902638
H	3.28934526	-4.91558218	-3.90773559
H	3.57443023	-3.30574656	-3.16195059
H	3.47636318	-6.02490425	-1.69464099
H	4.81904650	-4.84962606	-1.65568960
H	1.21606982	-3.29079008	-3.20692229
Pr	0.36689812	-0.47777480	1.61575675
N	2.54560781	-0.48822463	3.54788208
O	3.47110271	-0.47619814	4.33075523
O	2.69332933	-0.15394701	2.29142475
O	1.34211791	-0.82478935	3.88001037
O	1.55509782	0.75992912	-0.12115735

H 1.30542421 0.67811179 -1.15677953

H 2.51741004 0.59799492 -0.07188915

N -1.89789844 -1.71592724 2.99375272

O -1.01445937 -2.40495992 2.33471012

O -1.72465253 -0.42747033 2.95420432

O -2.81702638 -2.23289299 3.59340763

H 1.10728884 -5.07500696 -2.84755635

H -0.96884221 4.95568943 0.32615891

N 1.34449053 -0.62474853 -2.93156028

O 0.76624513 -1.24576867 -3.85053754

O 0.81561565 0.49207693 -2.50782633

O 2.39516449 -1.04552448 -2.38479805

L3*Pr(NO_3)$_2^+$

E= -11020.845953, E0 = -11020.414648, G= 227.7836 at T=298.150 K

H -4.07345963 3.41683006 -0.93772948

H -3.00333548 -2.82424593 -3.48538113

N -1.08443761 1.21185768 0.19658180

N 0.04305416 3.98701978 2.18160462

N -0.66170454 -1.26006854 -0.81112224

N 1.43852603 -4.15741730 -1.14205921

O 0.36880493 1.76210213 2.29633880

O 1.12484789 -2.64639163 0.49729148

C -1.25563157 2.43881845 0.71115845

C -2.33556914 3.25708747 0.32520235

H -2.50065351 4.22447300 0.79614639

C -3.22133255 2.80182099 -0.63766688

C -3.03229928 1.53421259 -1.22479677

C -3.89658999 0.98925549 -2.22926331

H -4.73843908 1.59245992 -2.57869434

C -3.68248367 -0.26016918 -2.73921537

H -4.35087299 -0.66841584 -3.50161028

C	-2.58702874	-1.06442750	-2.28528905
C	-2.33452702	-2.37143159	-2.74912095
C	-1.24914718	-3.08084083	-2.26163840
H	-1.07352531	-4.09967184	-2.60214496
C	-0.41212189	-2.48489118	-1.29773879
C	-1.70608521	-0.55290592	-1.29364860
C	-1.93178630	0.76612437	-0.75573057
C	-0.23734096	2.74294686	1.77869010
C	0.76856136	-3.12618399	-0.61611205
C	-0.34663561	5.28203964	1.57561457
H	-1.31669736	5.61460829	1.98630476
C	0.77332634	6.22767639	2.02749753
H	1.63937640	6.13057375	1.35264039
H	0.44448921	7.27659035	2.02351832
C	1.11875999	5.69944191	3.42704225
H	0.36434534	6.02972317	4.15952206
H	2.10595465	6.02911377	3.77990603
C	1.06092727	4.17975521	3.25090766
H	2.02116656	3.76031160	2.90483904
H	0.76066041	3.62836885	4.15448093
C	2.58728361	-4.72464323	-0.38354397
H	2.25011778	-5.05580568	0.61007297
H	3.33978891	-3.93111777	-0.23585553
C	3.09775543	-5.85076189	-1.28633082
C	2.75606894	-5.35085011	-2.69711614
C	1.36745727	-4.72723484	-2.50825167
H	2.74286652	-6.14824772	-3.45366192
H	3.47268343	-4.57715750	-3.01765823
H	2.56089711	-6.78879213	-1.07038462
H	4.17233706	-6.03015566	-1.14195836
H	1.14096713	-3.94302750	-3.24481058

Pr 0.58531177 -0.43401033 1.31058729

N -1.32202232 -1.51752484 3.18557501

O -0.03382376 -1.39867878 3.41276002

O -1.69999623 -1.09800506 2.01141453

O -2.08220482 -1.97606361 3.99851799

N 3.01759291 0.48787996 0.05144220

O 1.87364376 0.53866285 -0.57077140

O 2.95492411 -0.00585389 1.26423848

O 4.05219698 0.86242098 -0.44184041

H 0.58825547 -5.50909042 -2.55127215

H -0.42319751 5.19190645 0.48252252

L3*Nd(NO_3)$_3$(H_2O)

(H_2O in outer coordination sphere)

E= -11764.716923, E0 = -11764.246970, G= 244.1630 at T=298.150 K

H -4.37421703 3.27296662 -0.63751692

H -3.40208578 -2.99222827 -3.20453453

N -1.24946666 1.13495719 0.22457074

N 0.13054498 3.99160385 1.85215843

N -0.88888669 -1.33701384 -0.74831402

N 1.34009898 -4.02541733 -1.23423886

O 0.32571733 1.78174412 2.25031447

O 0.71295834 -2.94625664 0.65649962

C -1.37739980 2.36878753 0.71214485

C -2.50111365 3.17192245 0.42589056

H -2.61292887 4.15510368 0.88392484

C -3.48348188 2.67895842 -0.41660288

C -3.34004784 1.39735174 -0.98623270

C -4.28902435 0.80750036 -1.88207805

H -5.18000221 1.38159966 -2.15064883

C -4.09598637 -0.44573805 -2.39197350

H -4.82981110 -0.88332921 -3.07446003

C	-2.94096041	-1.21813464	-2.04333377
C	-2.68861198	-2.51967645	-2.52387190
C	-1.55031669	-3.19696450	-2.11619830
H	-1.35090184	-4.21286774	-2.46181583
C	-0.66887927	-2.56615877	-1.21675098
C	-1.98466277	-0.66833270	-1.14616358
C	-2.18278527	0.66079813	-0.61584014
C	-0.25085828	2.71414328	1.65528417
C	0.52225846	-3.20967174	-0.54808670
C	-0.18062587	5.17469883	1.02589571
H	-1.02950048	5.73793459	1.45654535
C	1.10859108	6.00277233	1.11091566
H	1.84109104	5.61776781	0.38255057
H	0.92900407	7.06761360	0.90146732
C	1.58991241	5.73002577	2.54423428
H	1.00701392	6.33054352	3.26252484
H	2.65538263	5.95830584	2.69127584
C	1.30080938	4.23666859	2.73277736
H	2.13442278	3.59402299	2.40086532
H	1.06041276	3.95436215	3.76928282
C	2.57522130	-4.50275898	-0.55835032
H	2.32669735	-5.27658558	0.18455373
H	3.02578020	-3.65310478	-0.01743266
C	3.45120263	-5.01049900	-1.71165729
C	2.98031902	-4.17357826	-2.91186428
C	1.46191692	-4.11786175	-2.70296812
H	3.24980211	-4.60985804	-3.88501549
H	3.38830996	-3.15146065	-2.84914804
H	3.26855922	-6.08233023	-1.89752638
H	4.52046013	-4.87830591	-1.49103606
H	0.99319118	-3.24855065	-3.18513107

Nd 0.82353902 -0.43295348 1.15504837

N -0.88315755 -1.25778794 3.41432023

O 0.38837785 -1.12972081 3.51758885

O -1.36792827 -0.97643369 2.22668266

O -1.60248339 -1.61218977 4.32783127

N 3.54973769 -0.57462341 2.17375612

O 2.90419674 0.54592991 2.02976155

O 2.87483287 -1.63331389 1.85638630

O 4.69695568 -0.61984873 2.57252598

N 2.07992816 0.45613533 -1.37105167

O 2.60142040 0.80296397 -2.41648340

O 2.10580802 -0.77962673 -0.96367389

O 1.45787084 1.27808881 -0.59493721

H 0.98025036 -5.04216290 -3.07303786

H -0.43230245 4.87599134 -0.00176403

O -4.03170109 -1.00555360 1.30717969

H -3.17111683 -1.02400613 1.78849864

H -4.42135000 -1.86635709 1.53648341

(NO_3^- in outer coordination sphere)

E= -11764.713775, E0 = -11764.243346, G= 246.0925 at T=298.150 K

H -4.67651796 2.91749692 -0.56148529

H -3.05279732 -3.22900248 -3.09336615

N -1.41391945 1.06376159 0.42040280

N -0.40841421 4.03732872 2.14777327

N -0.77483904 -1.35272741 -0.57612461

N 1.70532441 -3.84474325 -1.19131315

O 0.07559377 1.86010468 2.46616769

O 1.27698088 -2.52966714 0.60005480

C -1.66635776 2.27986479 0.90667582

C -2.84742904 2.97752738 0.57552069

H -3.07133603 3.94196725 1.03025174

C	-3.74680448	2.40224910	-0.30567813
C	-3.46945047	1.14146745	-0.87459505
C	-4.32713223	0.47645688	-1.80893981
H	-5.25075817	0.97355336	-2.11710501
C	-4.00278950	-0.75391644	-2.31024027
H	-4.66431189	-1.24982047	-3.02568603
C	-2.79561663	-1.41960061	-1.92111063
C	-2.40513587	-2.68980408	-2.39743900
C	-1.20235431	-3.23698401	-1.99314654
H	-0.89087176	-4.21376467	-2.36109161
C	-0.39102903	-2.52259469	-1.08640432
C	-1.92536879	-0.79137689	-0.99377418
C	-2.26605344	0.50710297	-0.46122938
C	-0.61101550	2.72841454	1.89004052
C	0.93174928	-2.97893023	-0.51984799
C	-0.85684788	5.21205044	1.37276697
H	-1.80769956	5.60541677	1.77762032
C	0.27271518	6.22665739	1.59571028
H	1.09789228	6.02293110	0.89368719
H	-0.06904889	7.26098967	1.44413352
C	0.71153808	5.92904949	3.03730583
H	-0.00458206	6.36802864	3.75176096
H	1.71277523	6.31713820	3.27317286
C	0.66469449	4.39911842	3.10600114
H	1.60957384	3.93322420	2.77718639
H	0.42948878	4.00021410	4.10472107
C	3.02364635	-4.18678188	-0.60321027
H	2.88597536	-4.77688313	0.31631795
H	3.53662133	-3.24902630	-0.32916164
C	3.74612403	-4.92890596	-1.73088098
C	3.16554260	-4.27653790	-2.99324799

C 1.67942393 -4.11703205 -2.65206385

H 3.31360936 -4.87404823 -3.90483189

H 3.58869267 -3.27040720 -3.14278793

H 3.50285101 -6.00460291 -1.70307195

H 4.83772945 -4.82128572 -1.64886487

H 1.22982705 -3.26735306 -3.19329309

Nd 0.37742120 -0.47915444 1.63589275

N 2.56441927 -0.66245556 3.52451324

O 3.48750496 -0.74011779 4.30701733

O 2.70841789 -0.18062074 2.32111335

O 1.36055064 -1.04704833 3.81597209

O 1.57259870 0.78248358 -0.08551277

H 1.30936408 0.70384663 -1.10972619

H 2.52684140 0.57488418 -0.05550056

N -1.95086670 -1.64918470 2.95955133

O -1.12731504 -2.35406280 2.24612951

O -1.68430161 -0.37745178 3.00726247

O -2.90428996 -2.13694572 3.53249860

H 1.13017714 -5.05560875 -2.84991360

H -0.99844682 4.95120478 0.31404710

N 1.32044411 -0.59886330 -2.90033078

O 0.73714709 -1.22355342 -3.81503105

O 0.79600298 0.51725197 -2.47655582

O 2.37444186 -1.02092087 -2.35870743

L3*Nd(NO_3)$_2^+$

E= -11407.749948, E0 = -11407.318288, G= 227.9701 at T=298.150 K

H -4.08252954 3.41308284 -0.93163258

H -3.01446986 -2.82961273 -3.47784734

N -1.08466530 1.21741021 0.18573795

N 0.04474495 3.98403192 2.17735386

N -0.66065377 -1.25642562 -0.82426769

N	1.43760002	-4.15336323	-1.14290261
O	0.37203282	1.75952935	2.28664851
O	1.12644112	-2.63651848	0.49105674
C	-1.25702477	2.43898416	0.70565295
C	-2.34175897	3.25586414	0.32605681
H	-2.50893736	4.22128916	0.80035377
C	-3.22725439	2.80027080	-0.63563836
C	-3.03615880	1.53472567	-1.22744155
C	-3.90244818	0.98824465	-2.22927666
H	-4.74656916	1.58972478	-2.57616067
C	-3.68865108	-0.26141298	-2.73897886
H	-4.35982561	-0.67099762	-3.49819613
C	-2.59134459	-1.06459188	-2.28777766
C	-2.34178233	-2.37443113	-2.74644446
C	-1.25684249	-3.08356524	-2.26006031
H	-1.08429861	-4.10454702	-2.59586787
C	-0.41438648	-2.48236251	-1.30255592
C	-1.70503533	-0.55139148	-1.30180550
C	-1.93140376	0.77034581	-0.76239944
C	-0.23415212	2.74017954	1.77338147
C	0.76966548	-3.12040210	-0.61854005
C	-0.34885684	5.28009272	1.57573307
H	-1.31711709	5.61097193	1.99200678
C	0.77258742	6.22561502	2.02444816
H	1.63557374	6.13018608	1.34548354
H	0.44300726	7.27430296	2.02370977
C	1.12459433	5.69526005	3.42155337
H	0.37360331	6.02422190	4.15811110
H	2.11333919	6.02471447	3.77020335
C	1.06641972	4.17582846	3.24341917
H	2.02514815	3.75640583	2.89323735

H 0.76911885 3.62302899 4.14711523

C 2.58914685 -4.71687031 -0.38558868

H 2.25452471 -5.04570484 0.60966927

H 3.34024882 -3.92138886 -0.24154098

C 3.09910607 -5.84479713 -1.28641975

C 2.75160718 -5.35044861 -2.69780874

C 1.36233640 -4.72888422 -2.50662684

H 2.73736644 -6.15050030 -3.45152378

H 3.46551204 -4.57640314 -3.02345705

H 2.56517625 -6.78333044 -1.06553638

H 4.17455578 -6.02126598 -1.14507961

H 1.13181305 -3.94858527 -3.24601793

Nd 0.59079611 -0.43181160 1.30592978

N -1.31829381 -1.51877582 3.16802573

O -0.03614003 -1.39457679 3.40717411

O -1.69122994 -1.10400081 1.98979652

O -2.08534193 -1.97954845 3.97513843

N 3.03683925 0.48966163 0.09510585

O 1.90087759 0.55934232 -0.53957880

O 2.95915508 -0.02362769 1.29671717

O 4.07942009 0.86657417 -0.38150439

H 0.58471853 -5.51263809 -2.54313254

H -0.42990488 5.19289112 0.48278889

L3*Pm(NO_3)$_3$(H_2O)

(H_2O in outer coordination sphere)

E= -12161.580205, E0 = -12161.109911, G= 244.9319 at T=298.150 K

H -4.36813641 3.29523277 -0.64048368

H -3.37702370 -2.96169400 -3.21804905

N -1.25432324 1.15512776 0.24155390

N 0.12112387 3.98978472 1.90208852

N -0.86822945 -1.30489242 -0.76153207

N	1.32537520	-4.04053497	-1.18317008
O	0.30822450	1.77509117	2.27720022
O	0.69877720	-2.91096187	0.68058729
C	-1.38829827	2.38390326	0.73884171
C	-2.50828195	3.18915439	0.44459099
H	-2.62885427	4.16751814	0.91050094
C	-3.47828269	2.70151949	-0.41532123
C	-3.32503629	1.42650616	-0.99692965
C	-4.26461077	0.84178603	-1.90594280
H	-5.15367794	1.41646028	-2.17934775
C	-4.06782103	-0.41028324	-2.41721702
H	-4.79718304	-0.84644598	-3.10538483
C	-2.91605878	-1.18467033	-2.06131673
C	-2.66598868	-2.48975492	-2.53440166
C	-1.53717899	-3.17306113	-2.11185312
H	-1.34375095	-4.19358540	-2.44705009
C	-0.65455794	-2.54116297	-1.21359932
C	-1.96331465	-0.63563204	-1.16082442
C	-2.17072272	0.68826908	-0.62082660
C	-0.26600179	2.71742606	1.69239020
C	0.51998621	-3.19212461	-0.52058744
C	-0.19812997	5.18787289	1.10014224
H	-1.04814076	5.73735714	1.54563916
C	1.08761382	6.01977253	1.19766521
H	1.81945443	5.65355015	0.45901579
H	0.90224236	7.08768749	1.01040268
C	1.57560813	5.72066641	2.62347698
H	0.99330688	6.30451965	3.35577369
H	2.64072752	5.95029211	2.77056098
C	1.29343939	4.22281694	2.78419662
H	2.12839723	3.59012270	2.43672395

H 1.05644584 3.91960788 3.81547761

C 2.54786611 -4.51916695 -0.48549688

H 2.28563881 -5.28888512 0.25702000

H 2.99249554 -3.66725969 0.05684718

C 3.44117618 -5.03435278 -1.62202442

C 2.99632072 -4.19545460 -2.83039188

C 1.47461236 -4.13457537 -2.64972353

H 3.28241944 -4.63180017 -3.79882669

H 3.40548277 -3.17447901 -2.75819969

H 3.25507402 -6.10522747 -1.81017196

H 4.50703621 -4.90815306 -1.38224304

H 1.01981628 -3.26331925 -3.14160132

Pm 0.80693865 -0.38754913 1.10570478

N -0.85273081 -1.35071242 3.33553123

O 0.42090154 -1.23297822 3.41805410

O -1.36203134 -1.00643623 2.17652035

O -1.55367780 -1.75090587 4.24524498

N 3.59366250 -0.40757221 1.93435001

O 2.94159865 0.68599761 1.65096819

O 2.89830208 -1.49099123 1.84785521

O 4.76705027 -0.39720163 2.25404835

N 2.02131677 0.24191079 -1.50864422

O 2.53332210 0.51130027 -2.58222651

O 2.14425421 -0.92424709 -0.95639735

O 1.30643630 1.09269536 -0.84783208

H 0.99598342 -5.05695629 -3.02858782

H -0.45233631 4.90842724 0.06774496

O -4.03152657 -0.95352888 1.29089272

H -3.16703844 -1.00916207 1.76281726

H -4.44548225 -1.80665755 1.50577700

(NO_3^- in outer coordination sphere)

E= -12161.576087, E0 = -12161.105417, G= 246.738 at T=298.150 K

H	-4.66012335	2.92413735	-0.56299210
H	-3.07295680	-3.22779846	-3.11190271
N	-1.40698624	1.04758346	0.40889454
N	-0.39200255	4.00352526	2.16003704
N	-0.78631210	-1.36791611	-0.59400350
N	1.69335198	-3.85639524	-1.21910322
O	0.07045060	1.82097149	2.47670627
O	1.27414072	-2.53260827	0.56766927
C	-1.65377569	2.26064706	0.90291625
C	-2.83122969	2.96677756	0.57500935
H	-3.04931808	3.92968440	1.03588307
C	-3.73330951	2.40234661	-0.30991054
C	-3.46311879	1.14251220	-0.88430953
C	-4.32584333	0.48427778	-1.81933379
H	-5.24734354	0.98733097	-2.12425828
C	-4.01042223	-0.74697173	-2.32428217
H	-4.67669010	-1.23732841	-3.03911376
C	-2.80652070	-1.42061698	-1.93837345
C	-2.42194343	-2.69226718	-2.41617227
C	-1.22090936	-3.24626994	-2.01498318
H	-0.91409391	-4.22309256	-2.38675618
C	-0.40554500	-2.53738308	-1.10745740
C	-1.93280363	-0.79918861	-1.00989676
C	-2.26321197	0.50070602	-0.47408962
C	-0.60088581	2.69595861	1.89648736
C	0.92186725	-2.99026847	-0.54695445
C	-0.82132417	5.18275547	1.38138235
H	-1.77164984	5.58643198	1.77746999
C	0.31663474	6.18576622	1.61477649
H	1.14578474	5.97372818	0.91990900

H	-0.01301390	7.22368574	1.46071136
C	0.73992521	5.88277674	3.05993676
H	0.02188808	6.32829809	3.76841569
H	1.74282837	6.26081753	3.30489850
C	0.67744583	4.35333395	3.12665915
H	1.62024856	3.87843966	2.80466843
H	0.43145084	3.95524597	4.12309551
C	3.01652670	-4.19190025	-0.63790774
H	2.88588238	-4.77063704	0.28974888
H	3.53210473	-3.25079322	-0.38074976
C	3.72867155	-4.94740486	-1.76304603
C	3.14096642	-4.30506325	-3.02738929
C	1.65755224	-4.13816071	-2.67779732
H	3.28089595	-4.91157007	-3.93427634
H	3.56600666	-3.30154037	-3.18819451
H	3.48248315	-6.02203846	-1.72289634
H	4.82110405	-4.84179640	-1.68999481
H	1.20738471	-3.28943920	-3.22052050
Pm	0.38046807	-0.50163537	1.60467207
N	2.42773223	-0.34286311	3.63007832
O	3.29308629	-0.23954880	4.47450256
O	2.63944173	-0.04525206	2.37673092
O	1.22888291	-0.74969912	3.89468002
O	1.56677616	0.79310918	-0.08928984
H	1.33344996	0.69963551	-1.11667573
H	2.53393102	0.67757004	-0.02244898
N	-1.86017859	-1.73471808	2.98854160
O	-0.92117649	-2.42417264	2.41648459
O	-1.75151646	-0.44790533	2.85214162
O	-2.77586532	-2.25216722	3.59613323
H	1.10378122	-5.07606411	-2.86589384

H -0.95603937 4.92426920 0.32113740

N 1.32932341 -0.61970735 -2.90934086

O 0.72722179 -1.24761534 -3.81134868

O 0.81881696 0.50274658 -2.48794889

O 2.38760304 -1.04527092 -2.38076544

L3*Pm(NO_3)$_2^+$

E= -11804.614976, E0 = -11804.183023, G= 227.3258 at T=298.150 K

H -4.07549524 3.41541338 -0.93220186

H -3.00618052 -2.82684064 -3.48104572

N -1.09333348 1.20852697 0.20354927

N 0.04528496 3.97133493 2.18856192

N -0.66950351 -1.26467371 -0.80641615

N 1.43578184 -4.15198946 -1.12627852

O 0.33711800 1.74219906 2.32817268

O 1.09221864 -2.66342354 0.52882969

C -1.26491034 2.42950869 0.72333986

C -2.34374595 3.25110626 0.33713335

H -2.50982833 4.21682215 0.81154764

C -3.22424364 2.79955006 -0.63092721

C -3.03310275 1.53409207 -1.22287333

C -3.89329624 0.99148321 -2.23205948

H -4.73290014 1.59600687 -2.58459139

C -3.67925453 -0.25795972 -2.74221587

H -4.34573460 -0.66410387 -3.50740647

C -2.58780527 -1.06530404 -2.28423190

C -2.33790541 -2.37480593 -2.74365544

C -1.25822937 -3.08720374 -2.25054765

H -1.08422375 -4.10788679 -2.58698392

C -0.42244229 -2.48961449 -1.28492725

C -1.70767689 -0.55637628 -1.29033196

C -1.93418980 0.76551712 -0.75055075

C	-0.24726905	2.72598243	1.79886138
C	0.75583029	-3.13169241	-0.59238434
C	-0.32530081	5.26394606	1.56487560
H	-1.29070771	5.61559296	1.97054195
C	0.80754584	6.19907331	2.00644946
H	1.67366397	6.07957077	1.33530712
H	0.49440718	7.25260544	1.98695028
C	1.14146817	5.68526983	3.41422963
H	0.38995424	6.03673172	4.13978338
H	2.13236594	6.00549126	3.76535845
C	1.06195033	4.16420937	3.25908470
H	2.01687813	3.72558856	2.92232990
H	0.75007510	3.62951756	4.16872883
C	2.58273506	-4.72073269	-0.36614421
H	2.23926163	-5.06970215	0.61918277
H	3.32631445	-3.92264938	-0.19934210
C	3.11127973	-5.82818651	-1.28162062
C	2.77633643	-5.31038666	-2.68771648
C	1.38016522	-4.70315075	-2.50113249
H	2.77711821	-6.09643078	-3.45616007
H	3.48793554	-4.52507019	-2.99067426
H	2.58175445	-6.77443504	-1.08432448
H	4.18629742	-5.99960947	-1.13108003
H	1.15110958	-3.91151619	-3.22880220
Pm	0.58031493	-0.43591371	1.31142616
N	-1.32078576	-1.51583672	3.16073823
O	-0.03869779	-1.39270484	3.39757776
O	-1.68789506	-1.09858978	1.98283494
O	-2.08932757	-1.97600067	3.96664596
N	2.96291709	0.50457847	0.01822838
O	1.80577374	0.55227596	-0.57680982

O 2.92798901 0.00044984 1.22530580

O 3.98464918 0.89111716 -0.49460378

H 0.60946864 -5.49227190 -2.56050444

H -0.40197730 5.16149998 0.47296867

L3*Sm(NO_3)$_3$(H_2O)

(H_2O in outer coordination sphere)

E= -12568.516240, E0 = -12568.046319, G= 244.140 at T=298.150 K

H -4.36221457 3.29610395 -0.65835512

H -3.40557003 -2.97807360 -3.20422411

N -1.22681069 1.16736555 0.18630789

N 0.11675554 4.01742935 1.86870825

N -0.88379687 -1.31805778 -0.76185894

N 1.31539750 -4.04264164 -1.20101202

O 0.35220462 1.80110514 2.20580435

O 0.69836897 -2.91265678 0.66591662

C -1.36019504 2.39654684 0.68297392

C -2.48883486 3.19581437 0.40385830

H -2.60665941 4.17436457 0.86987889

C -3.46959901 2.70372963 -0.44045961

C -3.32479095 1.42349517 -1.01261890

C -4.27660608 0.83224702 -1.90456963

H -5.16775322 1.40638900 -2.17243981

C -4.08669758 -0.42323494 -2.40990615

H -4.82289410 -0.86240345 -3.08881474

C -2.93396306 -1.19743764 -2.05795264

C -2.68985391 -2.50495172 -2.52628493

C -1.55745327 -3.18658590 -2.11053300

H -1.36575460 -4.20754147 -2.44539762

C -0.67300743 -2.55428171 -1.21486974

C -1.97362018 -0.64539981 -1.16654241

C -2.16495085 0.68902427 -0.64565802

C	-0.23844638	2.73761272	1.63388848
C	0.50966978	-3.20020700	-0.53185672
C	-0.20565274	5.21669960	1.07051647
H	-1.07177782	5.75158739	1.50307047
C	1.06468129	6.06824160	1.19874513
H	1.81616485	5.72045851	0.47096801
H	0.86563456	7.13475800	1.01723075
C	1.53016675	5.76406050	2.63096809
H	0.92320734	6.33071852	3.35677338
H	2.58801031	6.01052094	2.80163741
C	1.27019346	4.26013851	2.77156711
H	2.12170315	3.64488053	2.43346071
H	1.02238262	3.94207883	3.79589367
C	2.54446292	-4.51829338	-0.51295823
H	2.28628230	-5.26643658	0.25243011
H	3.00960493	-3.65937662	0.00053272
C	3.41059923	-5.06931496	-1.65284860
C	2.96414828	-4.23997879	-2.86718726
C	1.44590616	-4.14929533	-2.66864419
H	3.22976017	-4.69444036	-3.83310676
H	3.39254642	-3.22570658	-2.81498623
H	3.19989133	-6.13885593	-1.82171273
H	4.48229313	-4.96126556	-1.43086755
H	1.00229526	-3.27225256	-3.16058731
Sm	0.84537745	-0.39429829	1.09036517
N	-0.77379304	-1.36283481	3.33049273
O	0.50799739	-1.35395682	3.35032725
O	-1.30904543	-0.85533649	2.24774075
O	-1.45780015	-1.80613840	4.23368740
N	3.56158638	-0.47852126	2.12094998
O	2.84640312	0.60753316	2.05741835

O 2.96646833 -1.54132617 1.70372808

O 4.70349550 -0.47810847 2.54182625

N 2.06342006 0.30846471 -1.49729240

O 2.53623962 0.62292707 -2.57652521

O 2.11300206 -0.89987218 -1.04214215

O 1.46704042 1.16499949 -0.72899532

H 0.94573754 -5.06473827 -3.03612328

H -0.43633556 4.94262457 0.03106744

O -3.96126556 -1.00677252 1.31272829

H -3.10469913 -0.98348463 1.80123293

H -4.31925678 -1.87784111 1.55463672

(NO_3^- in outer coordination sphere)

E= -12568.512537, E0 = -12568.042363, G= 247.1782 at T=298.150 K

H -4.69042730 2.92362523 -0.55870408

H -3.05572081 -3.22006750 -3.10248256

N -1.41299140 1.08278501 0.39233992

N -0.42593652 4.03395700 2.16658759

N -0.77219558 -1.32771611 -0.60365492

N 1.69248116 -3.84885168 -1.17567980

O 0.07830016 1.85628176 2.44482660

O 1.27905893 -2.49146318 0.58597392

C -1.67324185 2.28836703 0.89683956

C -2.86111975 2.98149443 0.57777095

H -3.09322953 3.93710423 1.04693103

C -3.75662661 2.41169214 -0.31101963

C -3.47272801 1.15869665 -0.89446962

C -4.32918978 0.49334040 -1.82996404

H -5.25613928 0.98683947 -2.13404632

C -4.00261307 -0.73586339 -2.33355927

H -4.66602087 -1.23331034 -3.04623604

C -2.79426408 -1.40081072 -1.94590902

C	-2.40646577	-2.67596149	-2.41179276
C	-1.20732403	-3.22525406	-1.99901855
H	-0.90177315	-4.20825148	-2.35513902
C	-0.39305013	-2.50480103	-1.09883785
C	-1.92173898	-0.76953232	-1.02303112
C	-2.26464009	0.52896762	-0.48881072
C	-0.61715555	2.72782135	1.88564551
C	0.92753696	-2.96016979	-0.52268642
C	-0.88733679	5.21804905	1.41396904
H	-1.84149885	5.59432268	1.82717109
C	0.23213372	6.23999596	1.65407681
H	1.05792129	6.05854130	0.94668323
H	-0.12078928	7.27339745	1.52298951
C	0.67745000	5.91956711	3.08882761
H	-0.04128152	6.33768415	3.81307316
H	1.67532766	6.31321049	3.32962012
C	0.64610624	4.38822603	3.12860489
H	1.59482825	3.93809223	2.78897238
H	0.41767338	3.96811342	4.12018347
C	3.00524926	-4.19419479	-0.57742494
H	2.85826993	-4.75871849	0.35659924
H	3.53173923	-3.25743222	-0.32655492
C	3.71800232	-4.97470570	-1.68515754
C	3.14965248	-4.34389830	-2.96406555
C	1.66551697	-4.15412760	-2.62945604
H	3.29007435	-4.96514845	-3.86088467
H	3.58941197	-3.34869647	-3.13669920
H	3.45768094	-6.04544973	-1.63218141
H	4.81100321	-4.88204575	-1.60397518
H	1.22889268	-3.31185102	-3.19154310
Sm	0.38951811	-0.45273370	1.60224450

N 2.61480498 -0.62277287 3.42660141

O 3.55340147 -0.69826514 4.19259596

O 2.72019792 -0.09366890 2.24340129

O 1.43165243 -1.05791700 3.72078800

O 1.58083987 0.79957879 -0.11660529

H 1.35574186 0.70170379 -1.13491774

H 2.54229927 0.64430469 -0.04533114

N -1.89457858 -1.64293122 2.96686316

O -1.06947327 -2.34209752 2.25346875

O -1.64615560 -0.36819971 2.99929166

O -2.83618855 -2.13856411 3.55540085

H 1.10302055 -5.08884430 -2.80764580

H -1.02782714 4.97593737 0.35066721

N 1.37863731 -0.62528211 -2.94866133

O 0.79267710 -1.25805652 -3.85774493

O 0.86549699 0.49751416 -2.54377890

O 2.42712307 -1.05155551 -2.40133834

L3*Sm(NO$_3$)$_2$$^+$

E= -12211.554264, E0 = -12211.122377, G= 227.4215 at T=298.150 K

H -4.07222462 3.41890526 -0.93502885

H -3.00370860 -2.82820439 -3.48292446

N -1.07616162 1.22052026 0.17558667

N 0.05095728 3.96995807 2.19079757

N -0.65330857 -1.24933934 -0.83214784

N 1.44033515 -4.15148354 -1.12221372

O 0.39365286 1.74707317 2.26531219

O 1.13834059 -2.60940719 0.48883396

C -1.24829078 2.43805957 0.70119393

C -2.33361506 3.25656056 0.32474992

H -2.50306559 4.21963596 0.80289334

C -3.21717262 2.80487084 -0.64083540

C	-3.02540803	1.54175377	-1.23781335
C	-3.88962221	0.99589843	-2.24207282
H	-4.73315907	1.59745610	-2.59032869
C	-3.67581773	-0.25402176	-2.75187778
H	-4.34660912	-0.66249937	-3.51206827
C	-2.58046532	-1.05913508	-2.29868913
C	-2.33178043	-2.37112045	-2.75197077
C	-1.24952483	-3.08092332	-2.26008177
H	-1.07920778	-4.10369396	-2.59147334
C	-0.40747097	-2.47641540	-1.30348980
C	-1.69473422	-0.54563326	-1.31265557
C	-1.92101705	0.77693915	-0.77307731
C	-0.22111028	2.73053193	1.76866460
C	0.77752042	-3.10777521	-0.61261910
C	-0.35678941	5.27255678	1.61316442
H	-1.32817805	5.58576965	2.03565741
C	0.75538111	6.22167492	2.07741189
H	1.61740911	6.14942026	1.39440012
H	0.41398761	7.26636505	2.09792018
C	1.11740983	5.66844130	3.46297789
H	0.36500803	5.97469664	4.20781755
H	2.10340762	6.00213718	3.81518316
C	1.07530987	4.15225410	3.25574017
H	2.03699064	3.75008297	2.89351058
H	0.78794485	3.57880759	4.14973831
C	2.59061360	-4.70825005	-0.35837728
H	2.25701404	-5.01611662	0.64390314
H	3.34754944	-3.91532540	-0.23164396
C	3.08997822	-5.85630465	-1.23949134
C	2.74240398	-5.38538837	-2.65878987
C	1.35841501	-4.74954176	-2.47582603

H 2.71989655 -6.19907141 -3.39756417

H 3.46147370 -4.62321186 -3.00058174

H 2.54972935 -6.78660440 -0.99997020

H 4.16448402 -6.03806210 -1.09773874

H 1.13392889 -3.98002172 -3.22819281

Sm 0.61287695 -0.41675252 1.27772152

N -1.28875363 -1.49635053 3.13157082

O -0.00683425 -1.37079966 3.36178970

O -1.66328716 -1.08321607 1.95872521

O -2.05079269 -1.95739353 3.94451594

N 2.97029567 0.53356361 -0.05580518

O 1.81048751 0.57543474 -0.63758475

O 2.95143843 0.03300405 1.15189910

O 3.98677850 0.92255104 -0.57926971

H 0.57435185 -5.52734375 -2.49831963

H -0.43726173 5.20448399 0.51884389

L3*Eu(NO_3)$_3$(H_2O)

(H_2O in outer coordination sphere)

E= -12985.714441, E0 = -12985.244662, G= 244.4690 at T=298.150 K

H -4.35237360 3.29831791 -0.65558147

H -3.38114905 -2.97175097 -3.20890784

N -1.23254764 1.16241395 0.21583708

N 0.11072682 3.99131584 1.92355692

N -0.86176682 -1.30697787 -0.77021635

N 1.31915772 -4.05622911 -1.18277669

O 0.31728953 1.77157104 2.26556182

O 0.72166181 -2.89658022 0.67283010

C -1.37118459 2.38690996 0.72221154

C -2.49391317 3.19010472 0.43127233

H -2.61756563 4.16555500 0.90229344

C -3.46254563 2.70435214 -0.43081298

C	-3.31004238	1.42919242	-1.01256871
C	-4.25244713	0.84352744	-1.91823626
H	-5.14027500	1.41982639	-2.19230366
C	-4.06018639	-0.41093260	-2.42544723
H	-4.79196310	-0.84721607	-3.11098003
C	-2.91094375	-1.18756258	-2.06688857
C	-2.66713452	-2.49748349	-2.52995777
C	-1.54000938	-3.18217182	-2.10464406
H	-1.35329592	-4.20671034	-2.43127632
C	-0.65328854	-2.54723740	-1.21220183
C	-1.95389950	-0.63641834	-1.17232049
C	-2.15437436	0.69190812	-0.63910496
C	-0.25829035	2.71612930	1.68904865
C	0.52604175	-3.19395924	-0.52087486
C	-0.20259295	5.19480801	1.12746179
H	-1.06610131	5.73338079	1.56034946
C	1.07318425	6.03756523	1.25922751
H	1.82253468	5.68769264	0.53029650
H	0.88099432	7.10594559	1.08158064
C	1.53705299	5.72544622	2.69024611
H	0.93409544	6.29326963	3.41843820
H	2.59651780	5.96460676	2.86114454
C	1.26772344	4.22279978	2.82519555
H	2.11442447	3.60447454	2.48016357
H	1.01961100	3.90223575	3.84861088
C	2.54351091	-4.53700256	-0.49159071
H	2.28006458	-5.27696896	0.28002247
H	3.01596045	-3.67771792	0.01439929
C	3.40592408	-5.10330248	-1.62680185
C	2.96958351	-4.27566767	-2.84580088
C	1.45199060	-4.17038536	-2.64989519

H 3.23259401 -4.73743773 -3.80897236

H 3.40750933 -3.26527619 -2.79795766

H 3.18340182 -6.17135715 -1.78991187

H 4.47838259 -5.00606108 -1.40363514

H 1.01749706 -3.29176569 -3.14713693

Eu 0.81429547 -0.36967418 1.07306361

N -0.82033473 -1.30595517 3.32462025

O 0.45651051 -1.19970810 3.38064837

O -1.35038686 -0.96673775 2.17926574

O -1.50170577 -1.69398928 4.25616980

N 3.60184169 -0.28072315 1.91383076

O 2.94318771 0.78159010 1.57321370

O 2.93229651 -1.37983990 1.86476028

O 4.77147675 -0.23814335 2.25464988

N 2.03645635 0.22176015 -1.54943430

O 2.52846789 0.48454800 -2.63587427

O 2.17195058 -0.93453616 -0.98990023

O 1.33234525 1.08103085 -0.88836420

H 0.94420630 -5.08263397 -3.01488400

H -0.43327910 4.92442036 0.08708664

O -4.01529837 -0.97577184 1.28097975

H -3.15456605 -1.00624120 1.76169646

H -4.39700842 -1.84857285 1.47552598

(NO_3^- in outer coordination sphere)

E= -12985.712184, E0 = -12985.241966, G= 245.7084 at T=298.150 K

H -4.70339489 2.90865397 -0.56187856

H -3.01686358 -3.17925763 -3.17403007

N -1.44167566 1.06345308 0.43115607

N -0.46477857 4.04685974 2.17578840

N -0.75529045 -1.32052541 -0.62642848

N 1.69201982 -3.86564398 -1.15845525

O	0.10025242	1.88172781	2.42482281
O	1.22110128	-2.55349636	0.62265998
C	-1.69956338	2.27707911	0.92178345
C	-2.88350916	2.97004080	0.58946818
H	-3.12005758	3.92687225	1.05328643
C	-3.77068567	2.39842653	-0.30642548
C	-3.47518516	1.15164661	-0.89566255
C	-4.31715012	0.49924156	-1.85283506
H	-5.24061203	0.99573696	-2.16251969
C	-3.97886682	-0.72034526	-2.36975861
H	-4.63027620	-1.21072865	-3.09828949
C	-2.77091002	-1.38378394	-1.97860932
C	-2.37623763	-2.64842224	-2.46520686
C	-1.18460369	-3.20609117	-2.04345131
H	-0.87709284	-4.18609810	-2.40632701
C	-0.38148028	-2.49758863	-1.12420011
C	-1.90888429	-0.76444709	-1.03685057
C	-2.27230191	0.51810122	-0.47665370
C	-0.63140309	2.73593450	1.89121616
C	0.91244125	-2.98454762	-0.51313275
C	-0.96516883	5.22880888	1.44525170
H	-1.92308414	5.57640791	1.87495780
C	0.13106076	6.27643442	1.68341053
H	0.94918042	6.12855291	0.95950586
H	-0.25149962	7.30188274	1.57466853
C	0.60947543	5.94564295	3.10481167
H	-0.10755158	6.33243608	3.84793639
H	1.60048521	6.36233711	3.33504558
C	0.61971909	4.41392994	3.11887050
H	1.57243359	3.99526739	2.75124812
H	0.42508695	3.97078824	4.10754585

C	2.97849584	-4.24147272	-0.52262527
H	2.79286766	-4.81012440	0.40182620
H	3.51501775	-3.31745768	-0.24694104
C	3.71036291	-5.02685642	-1.61479056
C	3.18783116	-4.38286781	-2.90688896
C	1.69875705	-4.16951561	-2.61163712
H	3.34218669	-5.00438976	-3.80120778
H	3.65008998	-3.39566970	-3.06600690
H	3.43283010	-6.09376383	-1.57452059
H	4.80188990	-4.95132065	-1.50251722
H	1.28763211	-3.32389474	-3.18648171
Eu	0.38833588	-0.45345041	1.60558128
N	2.77288866	-0.87460160	3.18442988
O	3.79954600	-1.08499014	3.80042601
O	2.71280193	-0.00873114	2.21279645
O	1.66496360	-1.48260272	3.43847466
O	1.54366744	0.83625197	-0.13588427
H	1.37072289	0.71331996	-1.14506710
H	2.50533700	0.71973139	-0.01180292
N	-1.92582202	-1.36897171	3.12783861
O	-1.62168884	-1.85338295	1.96002185
O	-1.16305161	-0.41275927	3.53709126
O	-2.86837077	-1.78473949	3.77561426
H	1.12740815	-5.09603071	-2.80482888
H	-1.11289692	4.99836445	0.38031942
N	1.44105852	-0.64083791	-2.99760127
O	0.89535099	-1.28096735	-3.92828608
O	0.92153704	0.47926790	-2.62295461
O	2.46512175	-1.07282305	-2.40709686

L3*Eu(NO_3)$_2^+$

E= -12628.752062, E0 = -12628.320471, G= 227.1595 at T=298.150 K

H	-4.07702160	3.41157794	-0.92826158
H	-2.99878168	-2.82586575	-3.48865318
N	-1.08251655	1.21535015	0.18599394
N	0.04377043	3.97098088	2.19049549
N	-0.65115005	-1.25646770	-0.82967985
N	1.43910766	-4.16305161	-1.13423443
O	0.35974950	1.74376154	2.31059527
O	1.13159800	-2.64129925	0.49654049
C	-1.25721228	2.42993975	0.71510154
C	-2.34170747	3.24867010	0.33609578
H	-2.51289320	4.21084595	0.81579047
C	-3.22223783	2.79790592	-0.63246226
C	-3.02807736	1.53574026	-1.23119545
C	-3.88998437	0.99159962	-2.23819089
H	-4.73374414	1.59294569	-2.58631992
C	-3.67373872	-0.25686428	-2.75013041
H	-4.34260321	-0.66512799	-3.51213384
C	-2.57807755	-1.06152916	-2.29672146
C	-2.32809305	-2.37181187	-2.75458980
C	-1.24675679	-3.08339763	-2.26356244
H	-1.07591236	-4.10503435	-2.59824038
C	-0.40613163	-2.48353148	-1.30299306
C	-1.69376183	-0.55042225	-1.30783820
C	-1.92374074	0.77145356	-0.76540720
C	-0.23592252	2.72428203	1.79144084
C	0.77685499	-3.12367153	-0.60984623
C	-0.34037176	5.26444817	1.57727611
H	-1.31033278	5.60265970	1.98356938
C	0.78096104	6.20902586	2.02826095
H	1.64886177	6.10627270	1.35669649
H	0.45526871	7.25889492	2.01852298

C 1.12021005 5.68605852 3.43131065
H 0.36348057 6.02083158 4.15934324
H 2.10663772 6.01526546 3.78671718
C 1.05942500 4.16577673 3.26069498
H 2.01991224 3.74376845 2.91836929
H 0.75638205 3.61853075 4.16583443
C 2.58513212 -4.73067141 -0.37266198
H 2.24729705 -5.04968405 0.62466484
H 3.34311604 -3.94094896 -0.23278114
C 3.08615851 -5.86892843 -1.26543403
C 2.74679303 -5.37914467 -2.68030238
C 1.36304796 -4.74285221 -2.49572182
H 2.72675395 -6.18297768 -3.42988491
H 3.46911669 -4.61396599 -3.00833821
H 2.54193163 -6.80063534 -1.04074097
H 4.15938234 -6.05580235 -1.12056041
H 1.14382720 -3.96461940 -3.24075627
Eu 0.59513074 -0.42153198 1.28488255
N -1.28533566 -1.50703132 3.15495777
O 0.00899591 -1.50430882 3.32033753
O -1.68318892 -0.97553611 2.04048586
O -2.04222155 -1.96794164 3.97398424
N 2.96959043 0.59594697 0.03674282
O 1.80302942 0.77111822 -0.50198603
O 2.95440149 -0.08702952 1.14821362
O 3.99084020 1.02669644 -0.44429556
H 0.57821971 -5.51943111 -2.53333688
H -0.41346949 5.17076826 0.48434222

L3*Gd($(NO_3)_3$)(H_2O)

(H_2O in outer coordination sphere)

E= -13413.273332, E0 = -13412.802524, G= 245.5743 at T=298.150 K

H	-4.37378073	3.29281449	-0.67070132
H	-3.37650776	-2.97287130	-3.24790621
N	-1.24720514	1.15863585	0.17996809
N	0.10659364	3.97900820	1.89750886
N	-0.86986995	-1.29735816	-0.80070525
N	1.32242000	-4.04438448	-1.19186866
O	0.35395080	1.75773156	2.16832519
O	0.72178483	-2.84981751	0.63543648
C	-1.38071203	2.38353825	0.68423134
C	-2.50716114	3.18549752	0.40271026
H	-2.63001418	4.16084576	0.87394303
C	-3.48206258	2.69905806	-0.45290729
C	-3.33138466	1.42485845	-1.03727341
C	-4.27385139	0.83460820	-1.94027770
H	-5.16622782	1.40537679	-2.21133780
C	-4.07532024	-0.41796723	-2.45091867
H	-4.80665827	-0.85652423	-3.13548040
C	-2.92009139	-1.18936491	-2.09869146
C	-2.66609478	-2.49468255	-2.56795073
C	-1.53226483	-3.17125726	-2.14642572
H	-1.33811581	-4.19270468	-2.47792196
C	-0.65155852	-2.53311563	-1.25081444
C	-1.96634328	-0.63545525	-1.20236742
C	-2.17296171	0.69093889	-0.66806829
C	-0.25057584	2.70793271	1.63171089
C	0.52711535	-3.17021132	-0.55448967
C	-0.24367987	5.20435476	1.15269613
H	-1.10912156	5.70978546	1.62034941
C	1.01873827	6.06602716	1.29287982
H	1.75949323	5.75890970	0.53648293
H	0.80285317	7.13643217	1.16029680

C	1.51727474	5.70845699	2.70142293
H	0.91923130	6.23687553	3.46258187
H	2.57551813	5.96073294	2.86035848
C	1.27788508	4.19715261	2.78434157
H	2.12764859	3.60646653	2.40086699
H	1.05611145	3.83271670	3.79901314
C	2.53795552	-4.52291298	-0.48188815
H	2.25862932	-5.23301935	0.31180185
H	3.02238703	-3.65654254	0.00053100
C	3.39046407	-5.14224911	-1.59677148
C	2.96381474	-4.35634327	-2.84671044
C	1.44839537	-4.21757126	-2.65298533
H	3.21630049	-4.86027098	-3.79136539
H	3.42030430	-3.35304260	-2.84084129
H	3.15409112	-6.21264267	-1.71951652
H	4.46444225	-5.05060530	-1.37905312
H	1.02668273	-3.35149813	-3.18231487
Gd	0.84829372	-0.38418272	1.04976654
N	-0.70635915	-1.17176998	3.36433697
O	0.56262332	-1.00738275	3.41277504
O	-1.23902047	-0.95683694	2.18049645
O	-1.38556945	-1.50050282	4.31687307
N	3.56939292	-0.46900052	1.96100473
O	2.90941834	0.63820100	1.76706743
O	2.89497614	-1.54508078	1.71895421
O	4.72568178	-0.48079279	2.33621836
N	2.02399492	0.40035498	-1.49027598
O	2.51110387	0.71680605	-2.56087685
O	2.09661794	-0.81229234	-1.02649283
O	1.39058304	1.23659587	-0.73589855
H	0.92523360	-5.13518000	-2.98086143

H -0.48727572 4.96820784 0.10693965

O -3.91360259 -0.97638750 1.30768621

H -3.04583216 -0.99767303 1.77647579

H -4.29878235 -1.83942616 1.53590739

(NO_3^- in outer coordination sphere)

E= -13413.273675, E0 = -13412.802389, G= 247.4694 at T=298.150 K

H -4.66243505 2.93480635 -0.56832421

H -3.04639769 -3.23188376 -3.08954072

N -1.40151215 1.07462144 0.39382973

N -0.38904995 4.03038740 2.16034961

N -0.77422255 -1.33837020 -0.58433223

N 1.71270120 -3.83702826 -1.16145539

O 0.15277053 1.85678613 2.37603450

O 1.30375528 -2.45324826 0.57999277

C -1.64437568 2.29040265 0.88318378

C -2.82800102 2.98864532 0.55950117

H -3.05109668 3.95202661 1.01596463

C -3.73293877 2.41616774 -0.31858775

C -3.46264505 1.15515471 -0.89036769

C -4.32231760 0.48710734 -1.82124507

H -5.24638033 0.98320997 -2.12968969

C -4.00005674 -0.74601066 -2.31885409

H -4.66425180 -1.24302268 -3.03106236

C -2.79350138 -1.41355479 -1.92923439

C -2.40068507 -2.68662596 -2.39656854

C -1.19688773 -3.22978902 -1.98827088

H -0.88516134 -4.20791864 -2.35179639

C -0.38528305 -2.50974202 -1.08541882

C -1.92355764 -0.78194636 -1.00577044

C -2.26014972 0.51953572 -0.47958064

C -0.57025003 2.72891617 1.85401678

C	0.94488072	-2.94638419	-0.51622730
C	-0.88922942	5.22930574	1.45788372
H	-1.84552288	5.56733847	1.89833462
C	0.20936684	6.26909494	1.71796393
H	1.02579761	6.13645697	0.98926884
H	-0.17161082	7.29734278	1.63332474
C	0.68967873	5.90558290	3.13084531
H	-0.02485368	6.27745867	3.88383579
H	1.68214810	6.31466579	3.36808658
C	0.69625819	4.37385559	3.11171174
H	1.64763665	3.96090150	2.73448634
H	0.49973074	3.90936971	4.09008455
C	3.03707242	-4.15253258	-0.57170379
H	2.90798450	-4.69559860	0.37737575
H	3.55459356	-3.20339131	-0.35089025
C	3.74497223	-4.95099401	-1.66939473
C	3.15585017	-4.35479689	-2.95534062
C	1.67353463	-4.17047548	-2.60893130
H	3.29161096	-4.99491549	-3.83946323
H	3.58318424	-3.35933471	-3.15530586
H	3.49658108	-6.02281189	-1.58892512
H	4.83777142	-4.84511948	-1.60289395
H	1.22635722	-3.34119678	-3.18281913
Gd	0.37692547	-0.44008780	1.55175543
N	2.50520372	-0.63332826	3.42573380
O	3.42116785	-0.71029437	4.21596050
O	2.65012813	-0.12211217	2.23346877
O	1.30496097	-1.04493666	3.69094276
O	1.53406525	0.74295980	-0.17117059
H	1.28509450	0.64550042	-1.19810176
H	2.49298429	0.56408334	-0.12103509

N -1.88403153 -1.58026457 2.92052031

O -1.08569419 -2.28671145 2.18140912

O -1.61722505 -0.30733761 2.94243526

O -2.81157231 -2.06435442 3.53714514

H 1.11601520 -5.11241388 -2.76297116

H -1.03919184 5.02259159 0.38840139

N 1.32643712 -0.67141044 -2.97713804

O 0.74747127 -1.31873858 -3.87939787

O 0.78312218 0.43988478 -2.56282902

O 2.39227676 -1.06547785 -2.43920922

L3*Gd(NO_3)$_2^+$

E= -13056.311580, E0 = -13055.879189, G= 227.6496 at T=298.150 K

H -4.07403088 3.45650625 -1.02098346

H -3.01132607 -2.80111408 -3.56156278

N -1.10055101 1.23988247 0.11093028

N 0.01755770 3.96933055 2.16264224

N -0.67899173 -1.22625446 -0.89408034

N 1.40520513 -4.14157343 -1.14607358

O 0.36310616 1.74638820 2.20239115

O 1.10773766 -2.57109165 0.43729138

C -1.27009535 2.45691180 0.64015996

C -2.34657478 3.28193116 0.25206718

H -2.51178026 4.24748039 0.72686309

C -3.22652888 2.83553553 -0.71947592

C -3.04056168 1.57022655 -1.31409609

C -3.90011191 1.02692986 -2.32397532

H -4.73903894 1.63137817 -2.67819929

C -3.68734407 -0.22403777 -2.83233690

H -4.35451651 -0.62998760 -3.59700561

C -2.59754729 -1.03306711 -2.37228179

C -2.34566855 -2.34515214 -2.82409477

C	-1.26802278	-3.05671430	-2.32387757
H	-1.09703720	-4.07928610	-2.65505981
C	-0.43212700	-2.45604110	-1.35967612
C	-1.71781838	-0.52171278	-1.38078785
C	-1.94330573	0.80065942	-0.84263164
C	-0.24830557	2.73642373	1.71894324
C	0.74853373	-3.08532357	-0.65554941
C	-0.39429095	5.28215408	1.61141360
H	-1.37499857	5.57469320	2.02721214
C	0.70091641	6.23075294	2.11615467
H	1.57220078	6.18719101	1.44262457
H	0.34690392	7.27047396	2.16067767
C	1.05244994	5.64367247	3.49039483
H	0.28642997	5.91878462	4.23352337
H	2.02946711	5.97976828	3.86465406
C	1.03291678	4.13397598	3.23962283
H	2.00214243	3.75605249	2.87148309
H	0.74835396	3.52923131	4.11343336
C	2.54837632	-4.69267607	-0.36660355
H	2.21158886	-4.96338844	0.64509523
H	3.31724882	-3.90766811	-0.26515615
C	3.02797723	-5.87332487	-1.21431541
C	2.69247055	-5.43461561	-2.64672828
C	1.31897807	-4.77021980	-2.48558426
H	2.65814281	-6.26688814	-3.36399364
H	3.42531180	-4.69394207	-3.00613713
H	2.46940207	-6.78624249	-0.95107299
H	4.09847975	-6.07129955	-1.06456065
H	1.11263824	-4.01525927	-3.25771260
Gd	0.57183862	-0.38253820	1.17772245
N	-0.96924049	-1.49493372	3.24953628

O 0.31847668 -1.30654955 3.33787727

O -1.49243450 -1.11944306 2.10430169

O -1.62705064 -1.97164166 4.13794184

N 3.09917593 0.44137338 0.24278902

O 2.00638556 0.54981965 -0.47841486

O 2.91581821 -0.07962209 1.42232227

O 4.17867422 0.79662019 -0.15975346

H 0.52113163 -5.53420019 -2.49278355

H -0.45673716 5.24166059 0.51449668

L3*Tb(NO_3)$_3$(H_2O)

(H_2O in outer coordination sphere)

E= -13851.216728, E0 = -13850.746007, G= 245.6919 at T=298.150 K

H -4.36078978 3.30420899 -0.68751687

H -3.36455083 -2.97470665 -3.24469566

N -1.23306370 1.16710281 0.16741605

N 0.09816170 3.98139429 1.92267990

N -0.85361874 -1.28039074 -0.81539339

N 1.32056999 -4.05533028 -1.16862226

O 0.36685693 1.75647640 2.14280128

O 0.76007885 -2.78780389 0.62046599

C -1.36916769 2.39513421 0.67342317

C -2.49551010 3.19532013 0.38823751

H -2.61978650 4.17038345 0.85948586

C -3.46963310 2.71023512 -0.46873888

C -3.31811571 1.43436766 -1.05252814

C -4.25888824 0.84217268 -1.95474756

H -5.15130138 1.41252923 -2.22668886

C -4.06194353 -0.41281208 -2.46362209

H -4.79437065 -0.85250646 -3.14616346

C -2.90771270 -1.18236303 -2.10977578

C -2.65321875 -2.49112272 -2.56952453

C	-1.51985025	-3.16517329	-2.14306974
H	-1.32936811	-4.19035244	-2.46452594
C	-0.63549608	-2.51991940	-1.25559199
C	-1.95260561	-0.62286592	-1.21713161
C	-2.15952897	0.70167506	-0.68233031
C	-0.24841279	2.71392679	1.62503564
C	0.54416794	-3.14659882	-0.55497104
C	-0.25759822	5.21898031	1.20223284
H	-1.13434637	5.70380735	1.67059803
C	0.99217612	6.09414148	1.37335122
H	1.74221230	5.82286739	0.61218715
H	0.75988048	7.16437244	1.27128088
C	1.48863697	5.70431948	2.77408648
H	0.88299358	6.20699739	3.54652214
H	2.54384947	5.96261406	2.94326615
C	1.26422572	4.18900824	2.81795430
H	2.12126589	3.61695075	2.42248225
H	1.04351211	3.79765058	3.82280278
C	2.53278065	-4.53114128	-0.45172453
H	2.24615622	-5.19993877	0.37459338
H	3.04270625	-3.65720320	-0.01132792
C	3.35749316	-5.21839857	-1.54755509
C	2.93562460	-4.47710466	-2.82602334
C	1.42653537	-4.29072285	-2.62230182
H	3.16349125	-5.02838993	-3.75028753
H	3.41926074	-3.48756266	-2.87055993
H	3.09381509	-6.28684616	-1.62138462
H	4.43582153	-5.14433002	-1.34542716
H	1.02057743	-3.43683863	-3.18285656
Tb	0.84488869	-0.32894078	0.99200594
N	-0.65134889	-1.16915476	3.30151796

O	0.61847001	-0.98971236	3.31982040
O	-1.21220195	-0.93788218	2.13700914
O	-1.29999411	-1.52254760	4.26587105
N	3.57190728	-0.40172505	1.85762429
O	2.89194894	0.69579816	1.66378689
O	2.90794563	-1.48379421	1.63478386
O	4.73346329	-0.39093161	2.21545720
N	2.00308967	0.47567362	-1.52837658
O	2.49984932	0.78965020	-2.59421039
O	2.07497001	-0.73771143	-1.05991054
O	1.36005938	1.30719173	-0.78059727
H	0.87750351	-5.20746279	-2.90773630
H	-0.48678681	5.00259495	0.14887629
O	-3.90231967	-1.01591623	1.29404032
H	-3.03109121	-1.01491940	1.75541580
H	-4.25432587	-1.89642453	1.50880563

(NO_3^- in outer coordination sphere)

E= -13851.216413, E0 = -13850.745715, G= 249.7572 at T=298.150 K

H	-4.74535131	2.96289039	-0.47752759
H	-3.14900517	-3.18049955	-3.09415674
N	-1.45465219	1.10359251	0.39308396
N	-0.42231491	4.04675436	2.18724942
N	-0.85582423	-1.30498862	-0.59806573
N	1.67675567	-3.74839115	-1.22657955
O	0.20551635	1.88961577	2.25649595
O	1.27551627	-2.34148574	0.48617867
C	-1.68823767	2.32060862	0.89536816
C	-2.88499951	3.01247096	0.60600019
H	-3.10278773	3.97119761	1.07392144
C	-3.80904317	2.44478178	-0.25408059
C	-3.54776621	1.18702888	-0.84298438

C	-4.42023134	0.52518767	-1.76399326
H	-5.35260010	1.01991308	-2.04894638
C	-4.10235786	-0.70191330	-2.28510618
H	-4.77852678	-1.19247139	-2.99046302
C	-2.88922477	-1.36931336	-1.92316389
C	-2.49498510	-2.63632035	-2.40785933
C	-1.28362870	-3.18029475	-2.01965427
H	-0.98161590	-4.15719938	-2.39324427
C	-0.46092218	-2.46745992	-1.12073421
C	-2.00858808	-0.74727654	-1.00016153
C	-2.33614206	0.55192608	-0.45901808
C	-0.58171076	2.75775766	1.82153261
C	0.88859183	-2.87742233	-0.58274812
C	-1.00166631	5.26555681	1.58846557
H	-1.95226038	5.52989912	2.08770370
C	0.06200820	6.33509302	1.87375677
H	0.84747100	6.29266214	1.10150850
H	-0.36749592	7.34754181	1.88036180
C	0.62630469	5.89713097	3.23326254
H	-0.06581686	6.18103743	4.04333115
H	1.61070561	6.33444738	3.45303559
C	0.69841415	4.37297297	3.10307550
H	1.64512265	4.03317404	2.64859533
H	0.57630855	3.83167839	4.05364180
C	3.04122901	-3.98096061	-0.68422997
H	2.97607565	-4.34178638	0.35284317
H	3.57188869	-3.01319337	-0.69176185
C	3.65630317	-4.97437048	-1.67126167
C	2.99216151	-4.58874846	-2.99963188
C	1.54007339	-4.29820108	-2.59671831
H	3.04969811	-5.37549019	-3.76617599

H 3.42015147 -3.65589571 -3.40086603

H 3.39869428 -6.00940037 -1.38901842

H 4.75203419 -4.88570261 -1.69838727

H 1.08860099 -3.55219316 -3.26684117

Tb 0.35752183 -0.38047963 1.45768201

N 2.50889206 -0.50904983 3.28811193

O 3.42686319 -0.55351996 4.07739162

O 2.62562132 0.00746694 2.09940720

O 1.32354176 -0.96842319 3.55501437

O 1.47773778 0.65669465 -0.33047393

H 1.36980712 0.29686594 -1.34023786

H 2.44286847 0.58202082 -0.18428646

N -1.81378305 -1.50601280 2.94952774

O -1.05654097 -2.20932889 2.16324353

O -1.55502927 -0.23331112 2.94828486

O -2.69560885 -1.99621582 3.62444091

H 0.93845326 -5.22514868 -2.57122755

H -1.18665600 5.12408209 0.51374388

N 2.27130556 -0.94271314 -2.94077063

O 2.26535940 -1.61302328 -3.99392152

O 1.19295681 -0.24805225 -2.65057707

O 3.24481940 -0.91578519 -2.15138245

L3*Tb$(NO_3)_2^+$

E= -13494.256490, E0 = -13493.824157, G= 227.9087 at T=298.150 K

H -4.09701490 3.43817496 -0.96801138

H -3.02350593 -2.81630731 -3.53552341

N -1.09430635 1.23270798 0.11184809

N 0.04062942 3.95587921 2.16309333

N -0.67506444 -1.22093630 -0.89173031

N 1.42229593 -4.12884331 -1.14126301

O 0.40765584 1.73591626 2.16507435

O	1.14672995	-2.51965308	0.40672332
C	-1.25932407	2.45100522	0.64369851
C	-2.34576893	3.26995063	0.27441075
H	-2.50755930	4.23467541	0.75154185
C	-3.24234557	2.81988573	-0.68193388
C	-3.06251740	1.55483258	-1.27861524
C	-3.93437028	1.00421429	-2.27438617
H	-4.78340054	1.60207009	-2.61550784
C	-3.71956158	-0.24497901	-2.78735685
H	-4.39536667	-0.65490234	-3.54227805
C	-2.61577964	-1.04625511	-2.34622693
C	-2.35462642	-2.35384321	-2.80526519
C	-1.26115048	-3.05405354	-2.32039571
H	-1.08287442	-4.07321024	-2.65784192
C	-0.42020589	-2.44822931	-1.36488163
C	-1.72842634	-0.52698880	-1.36681628
C	-1.95439076	0.79049832	-0.82674944
C	-0.22265334	2.72801924	1.70518970
C	0.77055657	-3.06116033	-0.66979033
C	-0.39031714	5.27156353	1.63467407
H	-1.36694992	5.55089951	2.06875634
C	0.70519274	6.22285938	2.13368297
H	1.56561375	6.19528103	1.44553876
H	0.34335610	7.25888634	2.19737291
C	1.08403540	5.62171173	3.49457407
H	0.32881495	5.88219261	4.25382614
H	2.06476808	5.96053886	3.85648537
C	1.07130432	4.11487865	3.22598815
H	2.03738856	3.74823904	2.83869267
H	0.80455416	3.49819565	4.09709167
C	2.57299519	-4.66319323	-0.36165777

H 2.24751139 -4.90679407 0.66057396

H 3.34545755 -3.87844181 -0.28949669

C 3.03868008 -5.86747599 -1.18352628

C 2.68774986 -5.46541500 -2.62297988

C 1.31975400 -4.78894472 -2.46399832

H 2.63997173 -6.31673670 -3.31672263

H 3.42039990 -4.73946238 -3.01137257

H 2.48045230 -6.77118158 -0.88961536

H 4.11025190 -6.06527042 -1.04135191

H 1.11069655 -4.05093288 -3.25171781

Tb 0.56704527 -0.36208910 1.12513220

N -0.97936207 -1.48085165 3.16635561

O 0.30781773 -1.27870512 3.25977588

O -1.49720490 -1.11009502 2.01912761

O -1.63491011 -1.96469808 4.05167150

N 3.03874803 0.52119082 0.14950199

O 1.92737854 0.64765286 -0.53562564

O 2.88866258 -0.04865601 1.31311548

O 4.10521030 0.90058851 -0.26363486

H 0.51688111 -5.54744768 -2.44567370

H -0.46960247 5.24324512 0.53842926

L3*Dy(NO_3)$_3$(H_2O)

(H_2O in outer coordination sphere)

E= -14299.825833, E0 = -14299.354900, G= 245.9776 at T=298.150 K

H -4.36961746 3.29793930 -0.67329466

H -3.37064934 -2.97203350 -3.25002170

N -1.23733056 1.16387451 0.16419689

N 0.10780333 3.97194314 1.91141939

N -0.86032206 -1.28323543 -0.81499112

N 1.32131636 -4.04675484 -1.17086470

O 0.37460902 1.74768794 2.13223505

O	0.73322505	-2.80437922	0.62787861
C	-1.37227404	2.38729763	0.67303371
C	-2.50059700	3.18824863	0.39615700
H	-2.62411189	4.16161680	0.87102580
C	-3.47704744	2.70414066	-0.45934334
C	-3.32612872	1.43154335	-1.04727948
C	-4.26926374	0.83946550	-1.94865477
H	-5.16326284	1.40859580	-2.21835375
C	-4.07069302	-0.41340557	-2.45920563
H	-4.80345631	-0.85294002	-3.14158964
C	-2.91421580	-1.18368220	-2.10882688
C	-2.65961933	-2.49034476	-2.57317305
C	-1.52466595	-3.16439223	-2.14949107
H	-1.33154714	-4.18766546	-2.47568750
C	-0.64264303	-2.52156663	-1.25869286
C	-1.95888972	-0.62565476	-1.21688890
C	-2.16574001	0.69922370	-0.68258983
C	-0.24091543	2.70409679	1.61920202
C	0.53396237	-3.15104437	-0.55416471
C	-0.25698450	5.21092892	1.19736803
H	-1.12674999	5.69580317	1.67870247
C	0.99654937	6.08262253	1.35508382
H	1.73749292	5.80355453	0.58811778
H	0.76831740	7.15361738	1.25093079
C	1.50499403	5.69476843	2.75200868
H	0.90547913	6.19812489	3.52881622
H	2.56147122	5.95351458	2.91229534
C	1.28074598	4.17954111	2.79827285
H	2.13437891	3.60654378	2.39704466
H	1.06680620	3.78811359	3.80453467
C	2.53338265	-4.51910448	-0.45082644

H 2.24804091 -5.20405149 0.36254567

H 3.02825880 -3.64533639 0.00691781

C 3.37578106 -5.17870760 -1.55022800

C 2.95464182 -4.42347574 -2.82079768

C 1.44161367 -4.26019001 -2.62696385

H 3.19754386 -4.95752621 -3.75131941

H 3.42438102 -3.42663479 -2.84575415

H 3.12699819 -6.24930525 -1.64251077

H 4.45133209 -5.09327793 -1.33799505

H 1.02934802 -3.40326118 -3.17815399

Dy 0.84366512 -0.36039481 0.99712390

N -0.66370273 -1.14592540 3.30970979

O 0.60609436 -0.97497237 3.33258963

O -1.21819711 -0.92678142 2.13945389

O -1.32021260 -1.48282194 4.27535582

N 3.55385995 -0.43414128 1.87923026

O 2.87901354 0.66474402 1.68091798

O 2.88753796 -1.51506722 1.65691006

O 4.71466923 -0.42679253 2.24157548

N 2.00284648 0.42351222 -1.51988411

O 2.49891996 0.73072582 -2.58885431

O 2.07363415 -0.78729302 -1.04547071

O 1.36227512 1.25969613 -0.77626991

H 0.90638137 -5.17962599 -2.92926240

H -0.50025535 4.99759054 0.14662050

O -3.90171552 -0.99415779 1.29601192

H -3.03030920 -0.99752724 1.75807154

H -4.26625395 -1.86560404 1.52604699

(NO_3^- in outer coordination sphere)

E= -14299.827000, E0 = -14299.355518, G= 247.8696 at T=298.150 K

H -4.64992476 2.95819378 -0.59938622

H	-3.03016138	-3.22632527	-3.10502267
N	-1.41229272	1.07231629	0.39116254
N	-0.38317674	4.01281404	2.17447329
N	-0.78677863	-1.33089042	-0.57312578
N	1.71626878	-3.82161593	-1.12393177
O	0.15975840	1.83617949	2.34523892
O	1.30169928	-2.39632297	0.58186710
C	-1.64587557	2.29327416	0.87376273
C	-2.82143354	2.99971962	0.53895849
H	-3.03880525	3.96800780	0.98749810
C	-3.72765923	2.43085599	-0.34140143
C	-3.46604586	1.16440129	-0.90530705
C	-4.32071447	0.49597391	-1.84084702
H	-5.24052095	0.99404615	-2.15870547
C	-3.99776697	-0.73922431	-2.33439732
H	-4.65776253	-1.23396182	-3.05209160
C	-2.79564595	-1.41036987	-1.93614364
C	-2.39410663	-2.68045521	-2.40377235
C	-1.18995142	-3.21885562	-1.98736572
H	-0.87090403	-4.19359398	-2.35334826
C	-0.38766664	-2.49896789	-1.07657337
C	-1.93505204	-0.77941924	-1.00459254
C	-2.27166295	0.52189630	-0.48417461
C	-0.56813818	2.71859646	1.84393668
C	0.94382626	-2.91860056	-0.50224781
C	-0.88769484	5.22542572	1.49943876
H	-1.84548974	5.54943895	1.94702566
C	0.20728859	6.26303768	1.78274465
H	1.02297413	6.15110445	1.04976285
H	-0.17793952	7.29145432	1.72341740
C	0.69162452	5.86775255	3.18572640

H	-0.02340322	6.21835947	3.94838476
H	1.68256199	6.27572012	3.43090677
C	0.70493031	4.33716965	3.12936330
H	1.65648687	3.93794990	2.73799443
H	0.51431197	3.84802222	4.09680891
C	3.04175782	-4.11480093	-0.52518916
H	2.91467476	-4.63730526	0.43567413
H	3.55217814	-3.15739894	-0.32454351
C	3.75724435	-4.93285370	-1.60340953
C	3.16663170	-4.36939669	-2.90325713
C	1.68255854	-4.18729019	-2.56371212
H	3.30845118	-5.02789736	-3.77280831
H	3.58757496	-3.37567067	-3.12452650
H	3.51566911	-6.00424719	-1.49966502
H	4.84913683	-4.81814051	-1.53717518
H	1.23241246	-3.37446666	-3.15830088
Dy	0.34615377	-0.41617826	1.50646257
N	2.51850486	-0.61929798	3.29355454
O	3.45763755	-0.70478529	4.05508757
O	2.62364864	-0.09593574	2.10504580
O	1.32663238	-1.03506911	3.58882356
O	1.45728683	0.74824220	-0.23048937
H	1.21386409	0.62731194	-1.25387061
H	2.41624188	0.57057416	-0.17459732
N	-1.85951233	-1.57023680	2.90396404
O	-1.07465684	-2.27329826	2.15257144
O	-1.60084724	-0.29326537	2.90721631
O	-2.76828194	-2.05272961	3.54859948
H	1.13107133	-5.13588047	-2.69771576
H	-1.03604078	5.04233503	0.42546013
N	1.30566430	-0.71071804	-3.02207971

O 0.76002669 -1.37897229 -3.92969561

O 0.72874504 0.38995013 -2.62689185

O 2.37041402 -1.07568479 -2.46056914

L3*Dy(NO_3)$_2^+$

E= -13942.867702, E0 = -13942.435221, G= 228.1115 at T=298.150 K

H -4.09385490 3.44145060 -0.98281872

H -3.03085232 -2.82197070 -3.52883768

N -1.09155309 1.23945415 0.09995173

N 0.03378637 3.95766735 2.16275716

N -0.67671323 -1.22055483 -0.89734107

N 1.41385388 -4.13005257 -1.13061702

O 0.41854870 1.74157465 2.14544797

O 1.12347364 -2.53444600 0.42889392

C -1.25896645 2.45472145 0.63344455

C -2.34675217 3.27336216 0.26436529

H -2.51247597 4.23610020 0.74408245

C -3.23827291 2.82452798 -0.69625551

C -3.05405426 1.56146729 -1.29632592

C -3.92429328 1.01065576 -2.29332209

H -4.77175903 1.60913813 -2.63727736

C -3.71218276 -0.24076855 -2.80192828

H -4.38839531 -0.65130025 -3.55616474

C -2.61249685 -1.04420888 -2.35488415

C -2.35930848 -2.35783672 -2.80194521

C -1.27328026 -3.06224155 -2.30837393

H -1.10141313 -4.08591318 -2.63584518

C -0.42947018 -2.45199180 -1.35699379

C -1.72268188 -0.52392834 -1.37786555

C -1.94604695 0.79844713 -0.84125745

C -0.22049136 2.73098826 1.69636559

C 0.75809264 -3.06778455 -0.65320283

C	-0.40879491	5.27489948	1.64769626
H	-1.38523746	5.54364443	2.08885789
C	0.68273067	6.22919559	2.14969683
H	1.53996909	6.21329355	1.45723510
H	0.31403518	7.26210785	2.22371840
C	1.07251441	5.61953020	3.50373173
H	0.32061869	5.87050629	4.26942253
H	2.05363488	5.96089745	3.86216259
C	1.06653559	4.11464167	3.22425127
H	2.03310537	3.75413871	2.83265734
H	0.80439085	3.49088740	4.09174728
C	2.56494021	-4.66485310	-0.35180849
H	2.23575473	-4.92500257	0.66520387
H	3.32982230	-3.87431860	-0.26460636
C	3.04613757	-5.85389614	-1.18705487
C	2.69548869	-5.43863249	-2.62288475
C	1.32016671	-4.77818108	-2.46016407
H	2.65850210	-6.28219414	-3.32670832
H	3.42137027	-4.70040083	-3.00070381
H	2.49736977	-6.76734877	-0.90588671
H	4.11945295	-6.04130173	-1.04393649
H	1.10465252	-4.03531170	-3.24144530
Dy	0.57735169	-0.36469552	1.12914050
N	-0.94877607	-1.44948125	3.19331479
O	0.35033190	-1.34721696	3.23594451
O	-1.48207426	-1.00257850	2.08042455
O	-1.60486627	-1.91009939	4.09116268
N	3.05099988	0.44081727	0.11683001
O	1.95110154	0.46373683	-0.59802347
O	2.88212132	0.02314406	1.33878589
O	4.12137794	0.77662170	-0.32428256

H 0.52558327 -5.54552126 -2.45021629

H -0.49275213 5.25641441 0.55168533

L3*Ho(NO_3)$_3$(H_2O)

(H_2O in outer coordination sphere)

E= -14759.269059, E0 = -14758.798090, G= 245.9377 at T=298.150 K

H -4.36896896 3.29457736 -0.66742963

H -3.36200881 -2.97459722 -3.24265790

N -1.23566985 1.16532004 0.17035595

N 0.11096314 3.97619224 1.91261446

N -0.85190475 -1.28165185 -0.80862540

N 1.32677901 -4.05003119 -1.16982245

O 0.38141048 1.75223076 2.13387203

O 0.74740583 -2.80602574 0.63049316

C -1.36961675 2.38873172 0.67762309

C -2.49901462 3.18841267 0.40064287

H -2.62325740 4.16238022 0.87412596

C -3.47554255 2.70200872 -0.45348829

C -3.32391715 1.42919040 -1.04066086

C -4.26656389 0.83576459 -1.94157004

H -5.16120195 1.40375042 -2.21105170

C -4.06569290 -0.41694081 -2.45136547

H -4.79768610 -0.85810608 -3.13352776

C -2.90773129 -1.18542027 -2.10112596

C -2.65164471 -2.49162483 -2.56605911

C -1.51544094 -3.16353583 -2.14299417

H -1.32120228 -4.18664026 -2.46903849

C -0.63308483 -2.51967573 -1.25305319

C -1.95266283 -0.62638515 -1.20969701

C -2.16271472 0.69827878 -0.67577344

C -0.23587570 2.70712805 1.62207496

C 0.54385406 -3.15098858 -0.55034238

C	-0.25696477	5.21390343	1.19810653
H	-1.12589657	5.69882488	1.68094552
C	0.99622619	6.08698559	1.35119426
H	1.73528266	5.80745745	0.58259141
H	0.76681638	7.15764952	1.24622130
C	1.50886309	5.70148134	2.74722290
H	0.91115642	6.20550680	3.52499032
H	2.56558394	5.96126413	2.90422606
C	1.28579724	4.18613482	2.79621196
H	2.13882971	3.61311674	2.39373541
H	1.07470953	3.79632425	3.80371428
C	2.53631258	-4.52949429	-0.45076916
H	2.24759030	-5.21211147	0.36338678
H	3.03726268	-3.65876007	0.00613213
C	3.37313724	-5.19565058	-1.55048370
C	2.95635748	-4.43785906	-2.82095075
C	1.44462550	-4.26368666	-2.62592316
H	3.19459248	-4.97388172	-3.75156736
H	3.43372273	-3.44465613	-2.84680557
H	3.11618114	-6.26436329	-1.64221466
H	4.44948673	-5.11838484	-1.33924663
H	1.03803039	-3.40386271	-3.17684102
Ho	0.84175992	-0.35513917	0.99706960
N	-0.67794830	-1.16324663	3.28029346
O	0.59149230	-0.99461305	3.31167912
O	-1.22404552	-0.92714512	2.10940790
O	-1.34078443	-1.51232243	4.23699903
N	3.54628205	-0.43197513	1.86645293
O	2.87545133	0.66774744	1.67467690
O	2.87257385	-1.50930190	1.64142823
O	4.70815802	-0.43431348	2.22497988

N 1.99287868 0.45972791 -1.50238311

O 2.49172044 0.78570682 -2.56436253

O 2.06858897 -0.75480938 -1.04221833

O 1.34291565 1.28167474 -0.74970859

H 0.90305966 -5.17927933 -2.92883444

H -0.50250381 4.99916315 0.14817905

O -3.92015243 -0.99239141 1.29325950

H -3.04252410 -0.99608606 1.74279630

H -4.28363419 -1.86167765 1.53288841

(NO_3^- in outer coordination sphere)

E= -14759.272419, E0 = -14758.800833, G= 248.1065 at T=298.150 K

H -4.65021944 2.95253348 -0.58722156

H -3.03784060 -3.23287296 -3.09387422

N -1.40049720 1.07626271 0.38086978

N -0.37742829 4.01174831 2.17637920

N -0.78182209 -1.33162344 -0.58081895

N 1.71632409 -3.82801628 -1.12985504

O 0.17631242 1.83625245 2.33049083

O 1.31412840 -2.38898182 0.56767070

C -1.63646984 2.29387403 0.86891484

C -2.81696153 2.99695039 0.54308975

H -3.03511262 3.96300149 0.99609041

C -3.72476268 2.42793512 -0.33505982

C -3.46195531 1.16331267 -0.90293652

C -4.32056999 0.49368536 -1.83422005

H -5.24254751 0.99066752 -2.14751697

C -3.99926281 -0.74146479 -2.32884526

H -4.66300631 -1.23786294 -3.04192185

C -2.79531264 -1.41184735 -1.93475711

C -2.39765382 -2.68489408 -2.39795303

C -1.19243956 -3.22358489 -1.98558331

H	-0.87716967	-4.20074463	-2.34832668
C	-0.38507977	-2.50108933	-1.08080065
C	-1.92973959	-0.77888471	-1.00923085
C	-2.26360154	0.52396703	-0.48892745
C	-0.55669284	2.71808147	1.83830643
C	0.94919026	-2.91906404	-0.50924611
C	-0.88811064	5.22614431	1.50948453
H	-1.84373891	5.54665232	1.96432173
C	0.20672220	6.26475143	1.79030943
H	1.01776350	6.15780497	1.05148828
H	-0.18102621	7.29261541	1.73786211
C	0.70137310	5.86474419	3.18828321
H	-0.00750886	6.21262217	3.95782280
H	1.69426894	6.27173853	3.42728376
C	0.71464342	4.33416700	3.12728095
H	1.66434765	3.93533397	2.73111200
H	0.52821410	3.84262180	4.09433699
C	3.04416156	-4.12005758	-0.53550470
H	2.92071915	-4.64004898	0.42718494
H	3.55536103	-3.16217566	-0.33919191
C	3.75545907	-4.94090700	-1.61419487
C	3.15944433	-4.38171864	-2.91345811
C	1.67657566	-4.19813538	-2.56843281
H	3.29784918	-5.04334927	-3.78116059
H	3.57962036	-3.38881397	-3.13952041
H	3.51506758	-6.01211500	-1.50600207
H	4.84757090	-4.82541943	-1.55284452
H	1.22591305	-3.38566327	-3.16307998
Ho	0.35402527	-0.41475201	1.49040437
N	2.45826483	-0.55628121	3.33795214
O	3.37177444	-0.60846156	4.13292408

O 2.60285830 -0.08514070 2.13143277

O 1.25685942 -0.95811754 3.61148524

O 1.47009206 0.73234814 -0.23211667

H 1.22390187 0.61936390 -1.25652492

H 2.42760611 0.54604149 -0.17965090

N -1.83996034 -1.57165849 2.88051486

O -1.02331996 -2.26979303 2.15583062

O -1.61196661 -0.29192948 2.86367774

O -2.74705958 -2.06738162 3.51777196

H 1.12382162 -5.14648724 -2.69814014

H -1.04271030 5.04761934 0.43557388

N 1.31013191 -0.71516728 -3.02492166

O 0.75529027 -1.38519740 -3.92574024

O 0.73730594 0.38724700 -2.62740374

O 2.37858558 -1.08039200 -2.47116661

L3*Ho(NO_3)$_2$$^+$

E= -14402.310913, E0 = -14401.878517, G= 229.3640 at T=298.150 K

H -4.08853197 3.46860027 -1.04042482

H -3.01900315 -2.79602218 -3.59083748

N -1.10689998 1.25047636 0.06644182

N 0.01439119 3.95240545 2.15056276

N -0.68713868 -1.20313644 -0.93430376

N 1.39517415 -4.12299204 -1.14285254

O 0.38041756 1.73265338 2.12657309

O 1.10883844 -2.50908232 0.39759082

C -1.27510488 2.46447158 0.60314852

C -2.35497117 3.28903627 0.22375800

H -2.51967049 4.25239849 0.70257467

C -3.23970556 2.84604740 -0.74592865

C -3.05672956 1.58256328 -1.34603441

C -3.91930199 1.03627658 -2.35232711

H	-4.76172733	1.63796711	-2.70295215
C	-3.70547724	-0.21457042	-2.86202812
H	-4.37561178	-0.62142998	-3.62361336
C	-2.61154461	-1.02179170	-2.40748191
C	-2.35454822	-2.33437657	-2.85586643
C	-1.27313197	-3.04061675	-2.35463309
H	-1.09956348	-4.06360626	-2.68305779
C	-0.43687809	-2.43460894	-1.39375603
C	-1.73025382	-0.50567842	-1.42116451
C	-1.95602179	0.81476963	-0.88268995
C	-0.24519955	2.72960353	1.67682874
C	0.74434000	-3.05079341	-0.68001252
C	-0.41884109	5.27496910	1.64150834
H	-1.40046287	5.54179335	2.07223582
C	0.66791469	6.22298002	2.16579890
H	1.53289580	6.21324682	1.48293293
H	0.29958892	7.25553322	2.24619722
C	1.04194355	5.59892845	3.51772141
H	0.28116783	5.84171152	4.27731180
H	2.01899576	5.93607092	3.89094615
C	1.03871155	4.09731674	3.22176576
H	2.00847888	3.74227834	2.83288264
H	0.77074087	3.46292758	4.07961082
C	2.53571725	-4.65848112	-0.34905705
H	2.19817328	-4.89920759	0.66990697
H	3.30842042	-3.87481546	-0.26895535
C	3.00820065	-5.86502504	-1.16342652
C	2.67086220	-5.46756315	-2.60761046
C	1.30248749	-4.78828478	-2.46370959
H	2.62797475	-6.32130241	-3.29871726
H	3.40803361	-4.74415684	-2.99216890

H 2.44761801 -6.76808834 -0.87202901

H 4.07854557 -6.06195831 -1.01134443

H 1.10279071 -4.05343866 -3.25676918

Ho 0.54502684 -0.35185656 1.08983934

N -0.79344702 -1.45585692 3.24932933

O 0.49008000 -1.24140680 3.23800874

O -1.40299463 -1.09870315 2.13832831

O -1.37850344 -1.93633711 4.18482113

N 3.09610343 0.39731237 0.30702940

O 2.03165340 0.53720111 -0.45878455

O 2.84890199 -0.12527284 1.46859384

O 4.19664288 0.72985560 -0.05450989

H 0.49813882 -5.54545212 -2.44938493

H -0.48770598 5.26651907 0.54418343

L3*Er(NO_3)$_3$(H_2O)

(H_2O in outer coordination sphere)

E= -15229.645638, E0 = -15229.174599, G= 246.0011 at T=298.150 K

H -4.37257147 3.29144073 -0.67680854

H -3.36761117 -2.97536016 -3.26832056

N -1.24094272 1.15457141 0.15524898

N 0.10360601 3.96268535 1.90735793

N -0.86710453 -1.29052913 -0.82014441

N 1.31786883 -4.05374432 -1.17699754

O 0.37502822 1.73825359 2.12015104

O 0.73228180 -2.80068302 0.61537319

C -1.37310088 2.37799835 0.66416788

C -2.50196218 3.17919803 0.38951829

H -2.62359262 4.15252590 0.86497182

C -3.48035574 2.69646263 -0.46453679

C -3.33164787 1.42414403 -1.05300188

C -4.27460909 0.83234739 -1.95500100

H	-5.16854429	1.40171766	-2.22364140
C	-4.07496977	-0.41932079	-2.46830273
H	-4.80697250	-0.85746306	-3.15238094
C	-2.91782594	-1.18959439	-2.11938334
C	-2.65928960	-2.49400091	-2.58840537
C	-1.52294219	-3.16637492	-2.16592288
H	-1.32639456	-4.18741941	-2.49699759
C	-0.64468747	-2.52567768	-1.26959097
C	-1.96496117	-0.63363045	-1.22389495
C	-2.17159653	0.69095582	-0.68920827
C	-0.24096856	2.69419622	1.61009717
C	0.53193152	-3.15338969	-0.56350666
C	-0.25933346	5.20253897	1.19450426
H	-1.12885690	5.68860817	1.67529213
C	0.99525321	6.07273722	1.35362446
H	1.73593724	5.79360056	0.58648717
H	0.76826268	7.14412975	1.25070167
C	1.50297725	5.68276691	2.75023127
H	0.90457517	6.18677616	3.52748108
H	2.55995989	5.93940258	2.91058111
C	1.27595389	4.16789103	2.79524446
H	2.12868762	3.59318304	2.39458394
H	1.06063163	3.77632737	3.80119276
C	2.52702498	-4.52746534	-0.45302165
H	2.23770118	-5.20865297	0.36213264
H	3.02399969	-3.65401554	0.00297668
C	3.36880255	-5.19489765	-1.54823995
C	2.95154238	-4.44481134	-2.82318330
C	1.43888474	-4.27479744	-2.63173699
H	3.19329715	-4.98472357	-3.75061393
H	3.42563415	-3.45016050	-2.85321093

H 3.11681247 -6.26512241 -1.63568974

H 4.44436312 -5.11188364 -1.33525157

H 1.03020048 -3.41937304 -3.18795967

Er 0.82794672 -0.35711309 0.98179841

N -0.61279297 -1.09793127 3.32414317

O 0.65507370 -0.93172318 3.32483435

O -1.18295705 -0.89345819 2.15607643

O -1.25956070 -1.41912639 4.30158758

N 3.51332092 -0.45055774 1.88711131

O 2.84587383 0.65109152 1.71127963

O 2.84534526 -1.52160227 1.61921227

O 4.66871023 -0.46303329 2.26767159

N 2.00689769 0.50603449 -1.47766447

O 2.51049471 0.85500705 -2.53004813

O 2.06064391 -0.72298193 -1.05458355

O 1.37332487 1.31748474 -0.69983286

H 0.90096390 -5.19387150 -2.93050742

H -0.50155050 4.99096251 0.14313263

O -3.86856318 -0.98694217 1.31743598

H -2.99704027 -0.97709501 1.77910495

H -4.21650839 -1.86651182 1.54228199

(NO_3^- in outer coordination sphere)

E= -15229.648795, E0 = -15229.177172, G= 248.2065 at T=298.150 K

H -4.66377878 2.95139503 -0.59014869

H -3.01892090 -3.21799278 -3.11282158

N -1.40168905 1.09467280 0.36539403

N -0.39153674 4.03594828 2.17048359

N -0.76744187 -1.30735898 -0.60066849

N 1.71318662 -3.83339238 -1.10486686

O 0.23081037 1.87712586 2.24916315

O 1.30320776 -2.38088322 0.57938725

C	-1.64042389	2.31194782	0.85470837
C	-2.82727003	3.00642228	0.53382540
H	-3.05301571	3.96994185	0.98767060
C	-3.73431134	2.43183494	-0.34217632
C	-3.46513128	1.17132974	-0.91526669
C	-4.32085896	0.49858770	-1.84684360
H	-5.24678421	0.99000156	-2.15719748
C	-3.99160910	-0.73367292	-2.34323072
H	-4.65248775	-1.23416996	-3.05615830
C	-2.78255296	-1.39698446	-1.95225656
C	-2.38074493	-2.66819000	-2.41654587
C	-1.17737150	-3.20552468	-1.99796641
H	-0.85992616	-4.18289709	-2.35810494
C	-0.37305203	-2.48118782	-1.09165716
C	-1.91838360	-0.76048028	-1.02826822
C	-2.26167846	0.53859228	-0.50461978
C	-0.54507113	2.74675012	1.80312920
C	0.94948542	-2.90947223	-0.50100380
C	-0.96132189	5.25535965	1.56361246
H	-1.92180860	5.51702833	2.04498863
C	0.09503212	6.32601547	1.87164962
H	0.89402503	6.28772116	1.11322773
H	-0.33657965	7.33757830	1.87383795
C	0.63614148	5.88481998	3.23949003
H	-0.07065079	6.16491938	4.03806925
H	1.61552691	6.32349443	3.47806931
C	0.71296334	4.36118412	3.10589170
H	1.66755688	4.02370024	2.66656566
H	0.57471728	3.81687427	4.05246496
C	3.02717400	-4.14117670	-0.48843107
H	2.88159347	-4.65129185	0.47649249

H 3.55084515 -3.19022226 -0.29104805

C 3.74133468 -4.98155451 -1.55020142

C 3.17268682 -4.42440224 -2.86262083

C 1.68799210 -4.21701241 -2.53992629

H 3.31364465 -5.09495115 -3.72307825

H 3.61044431 -3.43965220 -3.09105706

H 3.48275447 -6.04803324 -1.43725312

H 4.83409929 -4.88247490 -1.47397697

H 1.25509655 -3.40680194 -3.15035892

Er 0.37253773 -0.37310246 1.44957697

N 2.53425193 -0.76249081 3.18247676

O 3.48667002 -0.94890070 3.90948200

O 2.63146996 -0.12552525 2.04858088

O 1.33871877 -1.17172360 3.45917892

O 1.49127150 0.72323024 -0.28655574

H 1.24911845 0.60330969 -1.31214559

H 2.44860744 0.53808874 -0.22743185

N -1.79745770 -1.43462217 2.93479204

O -1.13515675 -2.13383579 2.06851935

O -1.43022275 -0.19065532 3.01043582

O -2.69189095 -1.89783537 3.61353302

H 1.12548113 -5.16014194 -2.66722679

H -1.12468636 5.11730194 0.48496887

N 1.34332883 -0.74665862 -3.06412292

O 0.79916728 -1.41848743 -3.96954107

O 0.76949543 0.36056468 -2.68003225

O 2.40337896 -1.11198282 -2.49485660

L3*Er(NO$_3$)$_2^+$

E= -14872.689825, E0 = -14872.257121, G= 228.5721 at T=298.150 K

H -4.08139801 3.44147348 -0.96787697

H -3.00881958 -2.82408571 -3.52292681

N	-1.08404922	1.23223245	0.11228622
N	0.05018094	3.93662238	2.18623042
N	-0.66522866	-1.21638477	-0.88580167
N	1.43011940	-4.12544584	-1.10205114
O	0.43854299	1.72125900	2.14146590
O	1.15982258	-2.49382305	0.42306781
C	-1.24892843	2.44661140	0.64871860
C	-2.33555532	3.26717091	0.28032073
H	-2.50030041	4.22933722	0.76148516
C	-3.22717619	2.82219672	-0.68248546
C	-3.04416132	1.56050849	-1.28570664
C	-3.91212511	1.00978887	-2.28507090
H	-4.75971794	1.60763943	-2.62980127
C	-3.69800639	-0.24103041	-2.79515004
H	-4.37310600	-0.65081853	-3.55079031
C	-2.59822869	-1.04475391	-2.34805179
C	-2.34053421	-2.35752630	-2.79466486
C	-1.25279403	-3.05838466	-2.29906344
H	-1.07768834	-4.08114290	-2.62734079
C	-0.41157708	-2.44650936	-1.34632480
C	-1.71208477	-0.52363890	-1.36897802
C	-1.93784380	0.79583573	-0.83104622
C	-0.20557776	2.71497917	1.70803654
C	0.78117085	-3.05094481	-0.64300907
C	-0.39743140	5.25881100	1.68831491
H	-1.37340462	5.51922989	2.13539886
C	0.69318128	6.21009207	2.19825006
H	1.54782832	6.20449972	1.50238895
H	0.32252541	7.24128962	2.28526759
C	1.08976686	5.58482790	3.54297686
H	0.33936188	5.82253075	4.31438446

H	2.07083988	5.92527485	3.90243292
C	1.08874810	4.08380318	3.24328828
H	2.05462670	3.73392320	2.84046173
H	0.83485663	3.44677758	4.10351706
C	2.58380842	-4.64859009	-0.31911203
H	2.26098895	-4.88044786	0.70669985
H	3.35502672	-3.86176729	-0.25807053
C	3.04924345	-5.86184645	-1.12799466
C	2.69281983	-5.47792006	-2.57098532
C	1.32375526	-4.80276251	-2.41592121
H	2.64532328	-6.33683300	-3.25534058
H	3.42223024	-4.75436163	-2.96984935
H	2.49425292	-6.76325798	-0.82119870
H	4.12185431	-6.05498171	-0.98722667
H	1.11069727	-4.07584047	-3.21267629
Er	0.57878327	-0.34344599	1.08476198
N	-0.99206078	-1.43018472	3.09043741
O	0.29728949	-1.24897861	3.18721104
O	-1.49604619	-1.04891992	1.94401658
O	-1.65762353	-1.90452635	3.97438359
N	2.92572880	0.54243034	-0.09914268
O	1.77236104	0.59620321	-0.71452069
O	2.86271119	0.04008264	1.10344124
O	3.95455241	0.92313981	-0.59906346
H	0.52257913	-5.56257629	-2.38437128
H	-0.48411003	5.25315857	0.59236908

L3*Tm(NO_3)$_3$(H_2O)

(H_2O in outer coordination sphere)

E= -15711.145698, E0 = -15710.674892, G= 245.8307 at T=298.150 K

H	-4.35207415	3.31162238	-0.70123279
H	-3.37195897	-2.97982430	-3.23333454

N	-1.21792865	1.18228710	0.13417481
N	0.10057260	3.97840762	1.93064713
N	-0.85192555	-1.27610230	-0.81779569
N	1.30864120	-4.06240749	-1.13836491
O	0.39533785	1.75242794	2.09505987
O	0.73181790	-2.78695083	0.64308655
C	-1.35470593	2.40324974	0.64711714
C	-2.48481226	3.20235515	0.37075877
H	-2.61152005	4.17464924	0.84682292
C	-3.45958543	2.71797061	-0.48640031
C	-3.30964088	1.44422340	-1.07185912
C	-4.25469589	0.84863770	-1.96886468
H	-5.14776325	1.41780972	-2.24098873
C	-4.05907202	-0.40824586	-2.47055793
H	-4.79351330	-0.85126287	-3.14887929
C	-2.90527034	-1.17994046	-2.11370754
C	-2.65846372	-2.49369192	-2.56229615
C	-1.52814186	-3.16912627	-2.12907028
H	-1.34093320	-4.19759893	-2.44219303
C	-0.64195156	-2.52148581	-1.24578953
C	-1.94659257	-0.61707586	-1.22888124
C	-2.14855003	0.71324950	-0.70702267
C	-0.22854581	2.71277428	1.60291958
C	0.53003138	-3.15022492	-0.53173196
C	-0.27025652	5.23025942	1.24311030
H	-1.15089929	5.69399214	1.72566652
C	0.97119379	6.11228514	1.43630660
H	1.72354996	5.86192465	0.67056102
H	0.73132908	7.18286276	1.35684454
C	1.46957874	5.69523144	2.82842636
H	0.85672820	6.17285347	3.61101198

H	2.52142215	5.96135330	3.00613666
C	1.26118267	4.17697620	2.83509326
H	2.12543273	3.62245679	2.43071795
H	1.04032433	3.75969672	3.82944322
C	2.51947236	-4.53060436	-0.41410905
H	2.23279595	-5.20446730	0.40798858
H	3.01915622	-3.65302706	0.03095956
C	3.35804439	-5.20733738	-1.50594962
C	2.94142008	-4.46273375	-2.78420258
C	1.42923200	-4.28901052	-2.59245205
H	3.18165922	-5.00742006	-3.70926046
H	3.41645360	-3.46874452	-2.81876922
H	3.10282326	-6.27734661	-1.58706617
H	4.43408251	-5.12613058	-1.29447222
H	1.02316773	-3.43434930	-3.15170932
Tm	0.84215516	-0.33035347	0.93308592
N	-0.59450102	-1.17537129	3.23812652
O	0.68258333	-1.07915640	3.20357394
O	-1.18984723	-0.83627337	2.12080836
O	-1.22003341	-1.54899430	4.21151829
N	3.55238199	-0.36318815	1.78599584
O	2.83879590	0.71613842	1.64355004
O	2.92510772	-1.45160270	1.51608574
O	4.71561909	-0.33080646	2.14418101
N	1.97319961	0.35401717	-1.60341132
O	2.44291592	0.65662724	-2.68614316
O	2.06585121	-0.83935148	-1.11022913
O	1.33489656	1.19806373	-0.85962874
H	0.88886660	-5.20773077	-2.88798904
H	-0.49885193	5.04086256	0.18448389
O	-3.87807155	-0.97135359	1.29887748

H -3.00479794 -0.95020849 1.75667107

H -4.21291828 -1.85576653 1.52455103

(NO_3^- in outer coordination sphere)

E= -15711.148628, E0 = -15710.677500, G= 248.7671 at T=298.150 K

H -4.66133642 2.95834041 -0.58265585

H -3.04410815 -3.22385335 -3.10055280

N -1.38916671 1.10099566 0.34377280

N -0.39065152 4.01858807 2.18973160

N -0.76987630 -1.30208480 -0.62010229

N 1.70563889 -3.83515167 -1.11644304

O 0.22002761 1.85549009 2.26063633

O 1.31624568 -2.35447001 0.54594111

C -1.63218820 2.31069827 0.84742504

C -2.82166576 3.00618672 0.53673577

H -3.04796839 3.96461105 1.00136173

C -3.73009014 2.43856120 -0.34197941

C -3.46200442 1.18064070 -0.92239881

C -4.32388544 0.50804996 -1.84884965

H -5.25152302 1.00018024 -2.15318894

C -4.00169611 -0.72632885 -2.34541607

H -4.66992855 -1.22624075 -3.05187225

C -2.79302216 -1.39282870 -1.95879805

C -2.39909673 -2.67021871 -2.41352582

C -1.19599235 -3.21096349 -1.99670851

H -0.88859099 -4.19478416 -2.34805727

C -0.38219002 -2.48138928 -1.10284770

C -1.92036474 -0.75389338 -1.04391432

C -2.25552344 0.54863721 -0.52135354

C -0.54468864 2.73242140 1.81044424

C 0.94702268 -2.90253782 -0.51961446

C -0.94477099 5.24368858 1.58024037

H	-1.90820253	5.51174164	2.05224967
C	0.11614855	6.30543089	1.90289557
H	0.92408228	6.26241779	1.15425062
H	-0.30744812	7.32040787	1.90220380
C	0.63665724	5.85714197	3.27655697
H	-0.07785740	6.14170027	4.06664848
H	1.61638463	6.28759050	3.52839875
C	0.70293182	4.33300972	3.14113140
H	1.66103077	3.98883891	2.71476889
H	0.54738230	3.78828382	4.08483076
C	3.02427411	-4.13601780	-0.50732142
H	2.88594484	-4.61861372	0.47265145
H	3.55789399	-3.18439674	-0.34276447
C	3.71884561	-5.01137972	-1.55370545
C	3.14014173	-4.48500347	-2.87442493
C	1.66138470	-4.25321436	-2.54078770
H	3.26404071	-5.18093634	-3.71716666
H	3.58718920	-3.51267409	-3.13568878
H	3.45216417	-6.07192373	-1.40854561
H	4.81321144	-4.92042923	-1.49278176
H	1.23030186	-3.45319152	-3.16493511
Tm	0.38107756	-0.37748626	1.43873858
N	2.52438545	-0.52659345	3.22749734
O	3.45783758	-0.59093940	3.99991202
O	2.63688302	-0.02961588	2.03065944
O	1.33423221	-0.94024360	3.51911211
O	1.49628258	0.73708665	-0.29911759
H	1.28621387	0.60028654	-1.31448507
H	2.45802379	0.59224802	-0.21003592
N	-1.78592479	-1.50585151	2.88534093
O	-1.00698137	-2.21014953	2.12693596

O -1.52950037 -0.23311707 2.88217068

O -2.68657804 -1.99256301 3.54096603

H 1.08612967 -5.19181871 -2.64072347

H -1.09846342 5.10945511 0.49966902

N 1.39650023 -0.76595843 -3.10346818

O 0.84484750 -1.44803035 -3.99828482

O 0.82960469 0.34133577 -2.72948241

O 2.46032596 -1.12917161 -2.54187918

L3*Tm(NO_3)$_2^+$

E= -15354.191187, E0 = -15353.758722, G= 228.0095 at T=298.150 K

H -4.08928633 3.48240256 -1.06912804

H -3.01620030 -2.78331637 -3.62572646

N -1.10489833 1.26394606 0.02697230

N 0.00162726 3.94828796 2.14714384

N -0.68492490 -1.18413174 -0.97294396

N 1.38374829 -4.11977291 -1.14663458

O 0.41623434 1.74074697 2.05453587

O 1.14052379 -2.44922352 0.33766147

C -1.27353442 2.47503495 0.56956834

C -2.35552979 3.29925275 0.19423373

H -2.52240276 4.26064348 0.67578983

C -3.24011326 2.85908318 -0.77723533

C -3.05732203 1.59756887 -1.38157749

C -3.91967821 1.05123031 -2.38823652

H -4.76277733 1.65222764 -2.73847580

C -3.70543194 -0.19949542 -2.89868569

H -4.37599277 -0.60610628 -3.66006279

C -2.61116529 -1.00683224 -2.44451332

C -2.35288787 -2.31992531 -2.89079642

C -1.27169788 -3.02525854 -2.38730621

H -1.09879565 -4.04826117 -2.71535659

C	-0.43466040	-2.41696167	-1.42803109
C	-1.72902358	-0.48972133	-1.45998442
C	-1.95532274	0.83058214	-0.92095053
C	-0.23678543	2.73477221	1.63903356
C	0.75213480	-3.02349591	-0.71425921
C	-0.46735001	5.27685595	1.68806958
H	-1.45547748	5.50097513	2.12815332
C	0.59455067	6.23408413	2.24595881
H	1.45574403	6.27707577	1.55967677
H	0.19682102	7.25140238	2.36906672
C	0.99412173	5.56826019	3.57021093
H	0.23172197	5.75927114	4.34282589
H	1.96375120	5.91789865	3.95116949
C	1.03097308	4.08016014	3.21519470
H	2.00695944	3.76806927	2.80556130
H	0.78852838	3.40490532	4.04904413
C	2.52655101	-4.64102554	-0.34664348
H	2.20276856	-4.82108355	0.68907082
H	3.31875086	-3.87319469	-0.32062584
C	2.95498157	-5.89808559	-1.10651612
C	2.60793710	-5.56155920	-2.56371832
C	1.26030695	-4.83840132	-2.43666363
H	2.53255820	-6.44594765	-3.21216130
H	3.35902357	-4.87832451	-2.99214268
H	2.37402916	-6.76987362	-0.76408792
H	4.02167845	-6.11682367	-0.95891517
H	1.07508731	-4.13311815	-3.25952387
Tm	0.57448846	-0.31758726	1.02017939
N	-0.58421320	-1.43340456	3.25697398
O	0.68274009	-1.18261600	3.18551922
O	-1.25322449	-1.10637569	2.16716170

O -1.12220502 -1.91948116 4.21851110

N 3.13789678 0.42093277 0.31111270

O 2.08557343 0.56155634 -0.47273204

O 2.86754417 -0.10441027 1.46010697

O 4.24301958 0.75914794 -0.03283515

H 0.43488294 -5.57059669 -2.38325071

H -0.53635663 5.30803728 0.59121472

L3*Yb(NO_3)$_3$(H_2O)

(H_2O in outer coordination sphere)

E= -16203.916575, E0 = -16203.445940, G= 245.7998 at T=298.150 K

H -4.34556150 3.31052256 -0.68713999

H -3.35518074 -2.97790337 -3.22467566

N -1.21337533 1.18203175 0.15317966

N 0.10211512 3.96748734 1.96269119

N -0.83044851 -1.26465046 -0.82251227

N 1.31750238 -4.06760788 -1.12915683

O 0.38405439 1.73853123 2.12968349

O 0.76843858 -2.76229310 0.63972288

C -1.35461664 2.39977813 0.67301100

C -2.48352742 3.19915915 0.39345974

H -2.61382532 4.16895628 0.87364107

C -3.45287919 2.71751785 -0.47145256

C -3.29786801 1.44731379 -1.06339586

C -4.23915243 0.85358793 -1.96567690

H -5.13195133 1.42273128 -2.23864794

C -4.04196119 -0.40242666 -2.46906590

H -4.77540255 -0.84491086 -3.14881802

C -2.88805842 -1.17383730 -2.11171389

C -2.64107752 -2.48925209 -2.55614495

C -1.51171362 -3.16434669 -2.11959291

H -1.32773066 -4.19518185 -2.42673469

C	-0.62150759	-2.51277828	-1.24276638
C	-1.92916763	-0.60935402	-1.22888613
C	-2.13675904	0.71660131	-0.69893599
C	-0.23283331	2.70353723	1.63588762
C	0.55291086	-3.13835263	-0.52819484
C	-0.26415941	5.21998501	1.27325618
H	-1.14304066	5.68712234	1.75557077
C	0.98044688	6.09776258	1.46490860
H	1.73156178	5.84404039	0.69906217
H	0.74426371	7.16907120	1.38452601
C	1.47814631	5.68034172	2.85715342
H	0.86714756	6.16029310	3.63974762
H	2.53092718	5.94329786	3.03401041
C	1.26521850	4.16276836	2.86475611
H	2.12692022	3.60670662	2.45693922
H	1.04510093	3.74629951	3.85957217
C	2.52770710	-4.53805256	-0.40672040
H	2.24043846	-5.19630718	0.42770675
H	3.04028797	-3.65938997	0.02112890
C	3.35194802	-5.23858309	-1.49413204
C	2.94091249	-4.50022078	-2.77764177
C	1.43177354	-4.30480814	-2.58230782
H	3.17044640	-5.05646420	-3.69854689
H	3.42941356	-3.51323605	-2.82315469
H	3.07931590	-6.30515385	-1.56299436
H	4.42997932	-5.17231464	-1.28766453
H	1.03614354	-3.44812465	-3.14594007
Yb	0.81833434	-0.30809838	0.92167509
N	-0.64327168	-1.17559326	3.20912886
O	0.62895274	-1.02504301	3.20036840
O	-1.22659266	-0.90302330	2.06999254

O -1.27349985 -1.54267955 4.18331575

N 3.55767202 -0.22296333 1.69318926

O 2.86848307 0.83254689 1.39405382

O 2.90656090 -1.32776570 1.61460185

O 4.73074722 -0.16492140 2.02102828

N 1.95562482 0.30506280 -1.64008117

O 2.43195152 0.57475758 -2.73042846

O 2.09398580 -0.85612375 -1.08770359

O 1.26496053 1.15485585 -0.95498919

H 0.87847281 -5.21777296 -2.87159348

H -0.49373186 5.02974558 0.21502249

O -3.92089772 -0.95663983 1.27266061

H -3.04312778 -0.96868587 1.72253716

H -4.27594709 -1.83755326 1.48022044

(NO_3^- in outer coordination sphere)

E= -16203.921195, E0 = -16203.450024, G= 247.5264 at T=298.150 K

H -4.67150068 2.94698429 -0.56647617

H -3.02366161 -3.19829702 -3.13517714

N -1.39132261 1.10255480 0.35587084

N -0.40655956 4.04753542 2.18904543

N -0.74516624 -1.28316760 -0.65029061

N 1.70654953 -3.84822392 -1.10780740

O 0.30552557 1.91690016 2.16608405

O 1.30238187 -2.36834025 0.55121154

C -1.63287258 2.31750178 0.85235554

C -2.82747602 3.00527239 0.54499775

H -3.05848622 3.96302104 1.00749683

C -3.73614025 2.43248963 -0.33022276

C -3.46002579 1.18079436 -0.91838974

C -4.31800795 0.51291680 -1.85155785

H -5.24750948 1.00368595 -2.15224504

C	-3.98914599	-0.71513659	-2.35794544
H	-4.65472937	-1.21369660	-3.06788135
C	-2.77597761	-1.37705338	-1.97754180
C	-2.38038468	-2.65006804	-2.44222617
C	-1.18042767	-3.19499755	-2.02252984
H	-0.87524682	-4.17938423	-2.37415719
C	-0.36677438	-2.46779180	-1.12660396
C	-1.90254545	-0.74018502	-1.06123531
C	-2.25049067	0.55170190	-0.51928908
C	-0.52092886	2.76460338	1.77837551
C	0.94806445	-2.90668893	-0.52319992
C	-1.03576159	5.26866102	1.64886773
H	-1.99949765	5.46678448	2.15383387
C	-0.02159201	6.36920500	1.99281812
H	0.76256555	6.40658569	1.21898723
H	-0.49778900	7.35866070	2.05512071
C	0.56746805	5.88426781	3.32539010
H	-0.13384277	6.09093142	4.15077639
H	1.53145814	6.35417366	3.56737375
C	0.70815289	4.37489557	3.11092138
H	1.66531062	4.10442257	2.63224268
H	0.62039065	3.77695870	4.03075790
C	3.01202250	-4.16305542	-0.47724819
H	2.85334063	-4.62891245	0.50760740
H	3.56029558	-3.21877766	-0.31865022
C	3.70368719	-5.06537724	-1.50251639
C	3.14948440	-4.55054522	-2.83823991
C	1.67140269	-4.28830671	-2.52459502
H	3.27032328	-5.26181602	-3.66851044
H	3.61678886	-3.59089684	-3.11059165
H	3.41691828	-6.11878443	-1.34447658

H 4.79866600 -4.99234581 -1.42981040

H 1.25895596 -3.49492717 -3.16866970

Yb 0.39466798 -0.35382459 1.41893637

N 2.59492779 -0.74503046 3.09415221

O 3.57164168 -0.94923341 3.78668833

O 2.63461900 0.01507680 2.03759241

O 1.43541169 -1.25660241 3.33918548

O 1.52864897 0.69931209 -0.35057703

H 1.35442543 0.56070900 -1.35696852

H 2.49232721 0.60745925 -0.22426216

N -1.79912043 -1.27079725 2.95888090

O -1.30122566 -1.95297229 1.97520244

O -1.26446807 -0.10318989 3.12693095

O -2.70123863 -1.68881512 3.65942955

H 1.08202720 -5.21914339 -2.61599588

H -1.20861983 5.17677307 0.56666183

N 1.47643077 -0.81236666 -3.18076897

O 0.94241112 -1.50769114 -4.07834053

O 0.91068476 0.29289556 -2.82925582

O 2.53055978 -1.17729056 -2.59954119

L3*Yb(NO_3)$_2^+$

E= -15846.962677, E0 = -15846.530353, G= 228.2709 at T=298.150 K

H -4.04655600 3.47053313 -1.01229203

H -2.98394299 -2.80364776 -3.56094790

N -1.06880879 1.24598992 0.08729505

N 0.04981723 3.93245268 2.19616485

N -0.65551019 -1.20273268 -0.90982550

N 1.42356157 -4.12541437 -1.08571327

O 0.44816747 1.71965837 2.12436891

O 1.14852786 -2.48235726 0.42737150

C -1.23370695 2.45794034 0.62839305

C	-2.31366515	3.28418350	0.25214881
H	-2.47874928	4.24610758	0.73340553
C	-3.19901991	2.84538937	-0.71963739
C	-3.01845908	1.58332503	-1.32307768
C	-3.88025880	1.03742957	-2.33078146
H	-4.72124386	1.64011765	-2.68327355
C	-3.66862893	-0.21438070	-2.84009194
H	-4.33997488	-0.61866796	-3.60226893
C	-2.57729197	-1.02469885	-2.38391328
C	-2.32079244	-2.33943582	-2.82651854
C	-1.24116445	-3.04555845	-2.31971931
H	-1.06993759	-4.07019138	-2.64433050
C	-0.40484962	-2.43552732	-1.36065006
C	-1.69602990	-0.50732732	-1.39904654
C	-1.91906762	0.81354362	-0.86107850
C	-0.19619380	2.71501732	1.69909489
C	0.77820802	-3.04274035	-0.63936836
C	-0.40392587	5.25925064	1.71740961
H	-1.38878286	5.50227690	2.15480185
C	0.66977555	6.21216679	2.25965810
H	1.53151906	6.23315525	1.57299161
H	0.28502896	7.23633194	2.36621261
C	1.06074953	5.56289339	3.59471440
H	0.30066657	5.77649546	4.36369419
H	2.03465176	5.90637255	3.97044992
C	1.07833338	4.06865788	3.26381397
H	2.05124974	3.73722625	2.86212993
H	0.82495761	3.41128898	4.10870504
C	2.56510735	-4.65289068	-0.28915074
H	2.23198223	-4.87401104	0.73568994
H	3.34389043	-3.87346506	-0.22816098

C 3.02593541 -5.87712622 -1.08381605

C 2.68640804 -5.50146914 -2.53324509

C 1.32343137 -4.81007767 -2.39612126

H 2.63556290 -6.36622190 -3.20994544

H 3.42740870 -4.78982496 -2.93210196

H 2.45887303 -6.77035332 -0.77510023

H 4.09519243 -6.08002329 -0.93185443

H 1.12764382 -4.08574963 -3.19966888

Yb 0.57257980 -0.33600831 1.06219709

N -1.01730049 -1.42336571 3.05031967

O 0.25002530 -1.15701461 3.18598843

O -1.49899852 -1.11988544 1.87796581

O -1.68426359 -1.90591192 3.93128681

N 2.93524170 0.49606994 -0.12106804

O 1.80861092 0.47969621 -0.77514404

O 2.83522725 0.10751449 1.11760378

O 3.98011088 0.84103835 -0.61728656

H 0.51337934 -5.56059837 -2.36902761

H -0.47470233 5.27455378 0.62035078

L3*Lu(NO_3)$_3$(H_2O)

(H_2O in outer coordination sphere)

E= -16708.119428, E0 = -16707.647810, G= 246.8703 at T=298.150 K

H -4.36776161 3.31028891 -0.69717026

H -3.34930801 -2.96595669 -3.26748776

N -1.23251867 1.18291616 0.12995955

N 0.09667175 3.95941830 1.94181621

N -0.83808279 -1.25041652 -0.85013163

N 1.31269670 -4.05742550 -1.13239241

O 0.40364727 1.73450613 2.06121898

O 0.75870973 -2.72427058 0.60905421

C -1.36984301 2.40018535 0.64907461

C	-2.50126314	3.19906950	0.37620494
H	-2.63227963	4.16783810	0.85824203
C	-3.47352624	2.71784544	-0.48645008
C	-3.31792092	1.44990289	-1.08346307
C	-4.25711060	0.85441238	-1.98700380
H	-5.15307617	1.41949201	-2.25818157
C	-4.05353498	-0.39919454	-2.49474502
H	-4.78493977	-0.84243464	-3.17620516
C	-2.89537811	-1.16670799	-2.14179730
C	-2.63948393	-2.47711706	-2.59453321
C	-1.50573456	-3.14696431	-2.16075754
H	-1.31596601	-4.17510748	-2.47290421
C	-0.62180835	-2.49472117	-1.27833962
C	-1.94016230	-0.60171449	-1.25514054
C	-2.15546584	0.72119349	-0.72082371
C	-0.23366426	2.70073843	1.59588468
C	0.54745728	-3.11659884	-0.55625838
C	-0.29967508	5.22427654	1.29371703
H	-1.17765105	5.66397762	1.80276513
C	0.93497276	6.11539030	1.48940122
H	1.67577040	5.89532995	0.70341390
H	0.68126506	7.18473196	1.44406259
C	1.46427870	5.66618776	2.85993195
H	0.86235887	6.11569834	3.66723680
H	2.51642394	5.93916130	3.02473736
C	1.27291214	4.14616728	2.82903647
H	2.13470292	3.61258864	2.39232612
H	1.07573533	3.69834328	3.81492496
C	2.51183414	-4.52727175	-0.38940164
H	2.20766449	-5.16052628	0.45819160
H	3.03309202	-3.64601374	0.02296040

C 3.33126664 -5.26806831 -1.45369494

C 2.93230891 -4.56527090 -2.76092625

C 1.42533946 -4.34352350 -2.57620215

H 3.15624928 -5.15353966 -3.66309071

H 3.43477464 -3.58718681 -2.83712530

H 3.04812002 -6.33338737 -1.49194801

H 4.40940666 -5.20678759 -1.24663031

H 1.04135168 -3.50058222 -3.16804218

Lu 0.86224383 -0.31642464 0.91250181

N -0.54158443 -1.10246861 3.22828412

O 0.72518229 -0.91543651 3.21263361

O -1.12636554 -0.89384383 2.06867194

O -1.16943932 -1.44303870 4.21054983

N 3.55812740 -0.35995275 1.71131289

O 2.86005878 0.72662765 1.52576423

O 2.89907432 -1.44895506 1.50511980

O 4.72654104 -0.33582312 2.04694033

N 1.94428825 0.44922629 -1.58935428

O 2.41494179 0.76268983 -2.66682339

O 2.04091740 -0.75558823 -1.10804152

O 1.30162072 1.27624881 -0.83327961

H 0.86078948 -5.25850201 -2.83589602

H -0.54275352 5.05974483 0.23418131

O -3.82945204 -0.98557580 1.28348935

H -2.94812441 -0.97753572 1.72600245

H -4.18892479 -1.85027313 1.54463017

(NO_3^- in outer coordination sphere)

E= -16708.120723, E0 = -16707.649789, G= 249.7340 at T=298.150 K

H -4.64258289 2.96658874 -0.59558582

H -3.02786350 -3.23209810 -3.10084319

N -1.39330530 1.08486140 0.35523155

N	-0.36179805	4.00234079	2.18362355
N	-0.78095227	-1.31463993	-0.59457827
N	1.72212827	-3.81736565	-1.09777057
O	0.24409246	1.83843195	2.23728967
O	1.32431233	-2.32698083	0.55415595
C	-1.62159514	2.30289030	0.84580582
C	-2.80396914	3.00608659	0.52579188
H	-3.02125835	3.97179055	0.97913957
C	-3.71747947	2.43891597	-0.34871376
C	-3.46033239	1.17436814	-0.91933751
C	-4.32004595	0.50125813	-1.84763527
H	-5.24356270	0.99599212	-2.15988231
C	-3.99890852	-0.73537660	-2.34042907
H	-4.66428995	-1.23257470	-3.05140424
C	-2.79380035	-1.40544987	-1.94789314
C	-2.39094806	-2.67883635	-2.40615320
C	-1.18494582	-3.21332836	-1.98856473
H	-0.86752731	-4.19073057	-2.34836102
C	-0.37965581	-2.48599505	-1.08542085
C	-1.92949688	-0.76844686	-1.02561092
C	-2.26196027	0.53482950	-0.50886089
C	-0.52384442	2.71955395	1.79937518
C	0.95621008	-2.88920784	-0.50560981
C	-0.92147028	5.23268270	1.58998060
H	-1.87993932	5.49672270	2.07417059
C	0.14362881	6.29120016	1.90973783
H	0.94258708	6.25449085	1.15119898
H	-0.27962601	7.30620861	1.92271078
C	0.68038464	5.83109426	3.27310562
H	-0.02398804	6.10971975	4.07432747
H	1.66354609	6.25861263	3.51636028

C	0.74366450	4.30812931	3.12448883
H	1.69583106	3.96557283	2.68383598
H	0.59852856	3.75589728	4.06542873
C	3.04854941	-4.09455490	-0.49339709
H	2.92189288	-4.57539368	0.48892748
H	3.56636930	-3.13324428	-0.33520237
C	3.75270653	-4.96223593	-1.53954136
C	3.16154289	-4.44776773	-2.85920143
C	1.68024051	-4.23833513	-2.52187586
H	3.29382372	-5.14272642	-3.70143151
H	3.59078479	-3.46817398	-3.12316990
H	3.50267196	-6.02627993	-1.39046907
H	4.84576464	-4.85418749	-1.48241365
H	1.23727977	-3.44453287	-3.14658380
Lu	0.35499945	-0.37637538	1.41259861
N	2.44401979	-0.54511094	3.21944928
O	3.36524415	-0.61869061	4.00307274
O	2.57322741	-0.04242679	2.02336407
O	1.24364066	-0.95244652	3.49050546
O	1.42685688	0.71167582	-0.31464937
H	1.18765485	0.57326007	-1.34073997
H	2.38953710	0.55751026	-0.25359401
N	-1.79919374	-1.48438084	2.84815264
O	-1.02716219	-2.19004035	2.08232236
O	-1.53918517	-0.21073884	2.83453941
O	-2.69168568	-1.96672320	3.51543570
H	1.11894572	-5.18572807	-2.61788559
H	-1.08561563	5.10751152	0.50991118
N	1.31976974	-0.77921766	-3.08761907
O	0.78629786	-1.46720624	-3.98719025
O	0.72059739	0.31583220	-2.70439768

O 2.39065742 -1.11647975 -2.52202177

L3*Lu(NO$_3$)$_2^+$

E= -15846.962677, E0 = -15846.530353, G= 228.2709 at T=298.150 K

H -4.04655600 3.47053313 -1.01229203

H -2.98394299 -2.80364776 -3.56094790

N -1.06880879 1.24598992 0.08729505

N 0.04981723 3.93245268 2.19616485

N -0.65551019 -1.20273268 -0.90982550

N 1.42356157 -4.12541437 -1.08571327

O 0.44816747 1.71965837 2.12436891

O 1.14852786 -2.48235726 0.42737150

C -1.23370695 2.45794034 0.62839305

C -2.31366515 3.28418350 0.25214881

H -2.47874928 4.24610758 0.73340553

C -3.19901991 2.84538937 -0.71963739

C -3.01845908 1.58332503 -1.32307768

C -3.88025880 1.03742957 -2.33078146

H -4.72124386 1.64011765 -2.68327355

C -3.66862893 -0.21438070 -2.84009194

H -4.33997488 -0.61866796 -3.60226893

C -2.57729197 -1.02469885 -2.38391328

C -2.32079244 -2.33943582 -2.82651854

C -1.24116445 -3.04555845 -2.31971931

H -1.06993759 -4.07019138 -2.64433050

C -0.40484962 -2.43552732 -1.36065006

C -1.69602990 -0.50732732 -1.39904654

C -1.91906762 0.81354362 -0.86107850

C -0.19619380 2.71501732 1.69909489

C 0.77820802 -3.04274035 -0.63936836

C -0.40392587 5.25925064 1.71740961

H -1.38878286 5.50227690 2.15480185

C	0.66977555	6.21216679	2.25965810
H	1.53151906	6.23315525	1.57299161
H	0.28502896	7.23633194	2.36621261
C	1.06074953	5.56289339	3.59471440
H	0.30066657	5.77649546	4.36369419
H	2.03465176	5.90637255	3.97044992
C	1.07833338	4.06865788	3.26381397
H	2.05124974	3.73722625	2.86212993
H	0.82495761	3.41128898	4.10870504
C	2.56510735	-4.65289068	-0.28915074
H	2.23198223	-4.87401104	0.73568994
H	3.34389043	-3.87346506	-0.22816098
C	3.02593541	-5.87712622	-1.08381605
C	2.68640804	-5.50146914	-2.53324509
C	1.32343137	-4.81007767	-2.39612126
H	2.63556290	-6.36622190	-3.20994544
H	3.42740870	-4.78982496	-2.93210196
H	2.45887303	-6.77035332	-0.77510023
H	4.09519243	-6.08002329	-0.93185443
H	1.12764382	-4.08574963	-3.19966888
Yb	0.57257980	-0.33600831	1.06219709
N	-1.01730049	-1.42336571	3.05031967
O	0.25002530	-1.15701461	3.18598843
O	-1.49899852	-1.11988544	1.87796581
O	-1.68426359	-1.90591192	3.93128681
N	2.93524170	0.49606994	-0.12106804
O	1.80861092	0.47969621	-0.77514404
O	2.83522725	0.10751449	1.11760378
O	3.98011088	0.84103835	-0.61728656
H	0.51337934	-5.56059837	-2.36902761
H	-0.47470233	5.27455378	0.62035078

Ligand L4

E= -2142.322482, E0 = -2141.943196, G= 200.5447 at T=298.150 K

Cl -4.65378714 3.99378085 -1.50815678

Cl -3.47726941 -2.91266418 -4.40026712

N -1.14455450 1.39452755 -0.23621914

N 0.88670796 2.73105788 1.38006496

N -0.79553300 -1.14055133 -1.17604840

N 1.10540342 -2.95182633 0.03058689

O -0.85728842 3.97260189 2.14610434

O 1.08520746 -4.01330662 -1.97929978

C -1.33009493 2.60832334 0.26236436

C -2.42187214 3.43653727 -0.08372008

H -2.52922106 4.40148544 0.41146547

C -3.31226492 2.97607946 -1.02582753

C -3.15152264 1.68723452 -1.60143054

C -4.02668715 1.14222491 -2.58644271

H -4.86977339 1.74733448 -2.92395663

C -3.81709337 -0.10515072 -3.10302877

H -4.48735428 -0.50971705 -3.86325002

C -2.72516727 -0.91062397 -2.66403961

C -2.45182300 -2.21145511 -3.16556001

C -1.37969708 -2.93052459 -2.69101501

H -1.10991192 -3.91568112 -3.07168579

C -0.58977431 -2.35310960 -1.67163599

C -1.82774091 -0.41951004 -1.66471505

C -2.03376675 0.92642987 -1.13683045

C -0.40536746 3.15952706 1.33754981

C 0.59971559 -3.17912412 -1.21387267

C 1.64230227 1.92531943 0.39602691

H 1.82252121 2.51440001 -0.52340811

H 1.07744324 1.02638686 0.12127180

C 2.96258593 1.64891815 1.12331522

H 2.84569240 0.78314310 1.79736674

H 3.78192782 1.43285966 0.42153043

C 3.18250632 2.93188167 1.94123340

H 3.57358193 3.73075676 1.28954875

H 3.88324118 2.80015588 2.77879572

C 1.77009475 3.28874421 2.42266273

H 1.53722310 2.82401943 3.39750576

H 1.59305704 4.37080288 2.52540612

C 2.27824569 -3.73876595 0.45342606

H 3.10659552 -3.59952903 -0.25829107

H 2.02767897 -4.81510353 0.45896098

C 2.57850289 -3.21151686 1.86134076

C 1.18865013 -2.81811905 2.38543105

C 0.52001405 -2.19065738 1.15347350

H 1.22244620 -2.11917233 3.23442078

H 0.63421047 -3.71802306 2.69997644

H 3.22797132 -2.32190537 1.80266964

H 3.08224344 -3.96428776 2.48538494

H -0.57460755 -2.28580070 1.16603982

H 0.75640196 -1.11840630 1.06477737

L4*La(NO_3)$_3$(H_2O)

(H_2O in outer coordination sphere)

E= -11555.128220, E0 = -11554.677591, G= 228.5033 at T=298.150 K

Cl -4.89738369 3.64537525 -0.73742729

Cl -3.78762484 -3.34525824 -3.57334924

N -1.28458834 1.11064088 0.26824898

N 0.14199445 3.97497582 1.80161405

N -0.91210419 -1.35245550 -0.70037997

N 1.37783527 -3.96768093 -1.29094815

O 0.20017353 1.79666364 2.39211082

O	0.76285243	-2.97080779	0.64826882
C	-1.41124797	2.34744716	0.74511337
C	-2.52028918	3.16060495	0.45109200
H	-2.63071942	4.15168524	0.89022970
C	-3.50253701	2.66018414	-0.38768941
C	-3.37979627	1.36732125	-0.95128632
C	-4.32517815	0.76977229	-1.83875704
H	-5.21608829	1.33906376	-2.10832667
C	-4.12848711	-0.48474088	-2.34365702
H	-4.85941839	-0.92659765	-3.02270555
C	-2.97178435	-1.24832392	-1.99923146
C	-2.68610477	-2.54919434	-2.48054934
C	-1.53469765	-3.21210170	-2.08671045
H	-1.32696497	-4.21890163	-2.44964671
C	-0.66819733	-2.56803560	-1.18684459
C	-2.01661921	-0.69987273	-1.09671831
C	-2.22099090	0.63568914	-0.56780881
C	-0.29916516	2.70914268	1.70349860
C	0.55471003	-3.19963312	-0.56032258
C	-0.08653336	5.09153986	0.86171877
H	-0.90519083	5.74110508	1.22338068
C	1.24998534	5.84433651	0.89543235
H	1.96903598	5.34275770	0.22710146
H	1.14098763	6.89144230	0.57692027
C	1.68632722	5.68868399	2.36048818
H	1.12901652	6.39222670	3.00137520
H	2.76112962	5.86575842	2.51026225
C	1.30307317	4.24130964	2.68968964
H	2.10406637	3.52161956	2.44778633
H	1.02037477	4.07973719	3.74124885
C	2.63642859	-4.44098949	-0.65527976

H 2.41698432 -5.23778629 0.07218103

H 3.08574080 -3.59840870 -0.10224037

C 3.49454045 -4.90017557 -1.84121943

C 2.98872757 -4.03040981 -3.00277805

C 1.47382200 -4.00512218 -2.76532459

H 3.24536610 -4.42871237 -3.99539232

H 3.38318491 -3.00494409 -2.91281438

H 3.32069635 -5.96755648 -2.05807447

H 4.56636524 -4.76178360 -1.63765669

H 0.98460573 -3.12607026 -3.20759106

La 0.84659690 -0.46117336 1.32462835

N -0.95491135 -1.39926553 3.56271911

O 0.30802971 -1.27091157 3.72720313

O -1.39359009 -1.10355890 2.35466981

O -1.71744430 -1.76610327 4.43542671

N 3.58035398 -0.59539592 2.50373173

O 2.87603283 0.49861664 2.49362230

O 3.00161028 -1.62980115 1.97123408

O 4.69964600 -0.64429563 2.97490120

N 2.10535836 0.55871612 -1.23859715

O 2.57142901 0.96606803 -2.28887010

O 2.06194949 -0.70679313 -0.94908088

O 1.61918092 1.35707319 -0.34186357

H 0.99935555 -4.92305470 -3.15908909

H -0.34225461 4.71270084 -0.13802040

O -4.00360823 -0.94416296 1.34744322

H -3.16719460 -1.04560661 1.86149848

H -4.51685286 -1.72657275 1.61141205

(NO_3^- in outer coordination sphere)

E= -11555.122669, E0 = -11554.671427, G= 231.0993 at T=298.150 K

Cl -5.20045567 3.24251842 -0.69036120

Cl	-3.40397215	-3.61800528	-3.46291733
N	-1.42220354	1.03468645	0.45163086
N	-0.39966443	4.03238297	2.10701561
N	-0.77784383	-1.37794387	-0.54126942
N	1.71871459	-3.83047748	-1.23133826
O	-0.02153555	1.86114931	2.59019852
O	1.27586663	-2.62016582	0.63142890
C	-1.67500627	2.25226450	0.92889303
C	-2.84230113	2.96190047	0.59136915
H	-3.06880617	3.93009496	1.03477049
C	-3.73830485	2.38432384	-0.29134253
C	-3.47604394	1.11406767	-0.86172575
C	-4.32597446	0.45049250	-1.79684365
H	-5.24393892	0.94855189	-2.11197662
C	-4.00083828	-0.78116083	-2.29268026
H	-4.65597582	-1.27783716	-3.01007032
C	-2.80108738	-1.44621909	-1.89773941
C	-2.39392018	-2.72216105	-2.36457324
C	-1.18921089	-3.26550508	-1.96272814
H	-0.88024253	-4.24100018	-2.33367395
C	-0.38923237	-2.54198766	-1.05930007
C	-1.92895317	-0.82265598	-0.96508682
C	-2.27340841	0.48193625	-0.43316415
C	-0.64151156	2.71862841	1.93128347
C	0.93921953	-3.01261091	-0.51058352
C	-0.77631444	5.16420507	1.23390114
H	-1.72070479	5.62356663	1.57968020
C	0.38792232	6.14601851	1.42019498
H	1.22583413	5.85592270	0.76522619
H	0.09516925	7.17828417	1.17918742
C	0.76554531	5.94234133	2.89521098

H	0.04590090	6.46351242	3.54819512
H	1.77417409	6.30670166	3.13730526
C	0.65154725	4.42516756	3.07811856
H	1.58711731	3.89868927	2.82274842
H	0.36277196	4.11442280	4.09395695
C	3.04273820	-4.19297981	-0.66790420
H	2.91399598	-4.83715868	0.21594737
H	3.54703665	-3.26745892	-0.34125993
C	3.76840854	-4.86034441	-1.83943534
C	3.17485666	-4.14333439	-3.06021166
C	1.68845391	-4.02200556	-2.70548534
H	3.32571650	-4.68599319	-4.00494766
H	3.58644938	-3.12570405	-3.15341043
H	3.53793430	-5.93864012	-1.87255859
H	4.85902548	-4.74450016	-1.75540674
H	1.22231603	-3.14927149	-3.19364667
La	0.40490597	-0.54144663	1.78494990
N	2.63266110	-0.71060914	3.74317479
O	3.55127096	-0.77351713	4.53282166
O	2.78343892	-0.22389553	2.53997564
O	1.43316031	-1.11564279	4.02737093
O	1.64609587	0.84996283	0.08789846
H	1.37120795	0.79406869	-0.93129170
H	2.60716200	0.67803377	0.10549285
N	-2.03688979	-1.76311183	3.01214504
O	-1.17447758	-2.46543527	2.34168720
O	-1.78712487	-0.48593581	3.07149339
O	-3.01258397	-2.25752354	3.54040217
H	1.15127861	-4.95582247	-2.95231366
H	-0.89901561	4.83174658	0.19312771
N	1.28270352	-0.48357105	-2.74361777

O 0.64920139 -1.08505666 -3.64155054

O 0.80239534 0.63873893 -2.28924012

O 2.34534550 -0.93769270 -2.24714732

L4*La(NO_3)$_2^+$

E= -11198.152211, E0 = -11197.739891, G= 212.6490 at T=298.150 K

Cl -4.57999468 3.79377484 -1.04870248

Cl -3.39114976 -3.14701843 -3.88123965

N -1.12770307 1.18401659 0.24909462

N 0.05406790 3.99699116 2.13222551

N -0.70348066 -1.28873873 -0.76093841

N 1.44492805 -4.12877464 -1.18346667

O 0.25678095 1.77149355 2.42844868

O 1.03832126 -2.77262354 0.57083422

C -1.29176295 2.41263342 0.75268358

C -2.35771775 3.24223328 0.36747098

H -2.51960444 4.21443462 0.82869434

C -3.24904490 2.78657579 -0.59139526

C -3.07435513 1.50887644 -1.18356395

C -3.92978120 0.96777630 -2.19019365

H -4.76498508 1.57315421 -2.54539990

C -3.71577954 -0.28143057 -2.70007563

H -4.37811708 -0.68526995 -3.46717095

C -2.62953901 -1.08719885 -2.24327755

C -2.36037374 -2.40048242 -2.70856380

C -1.26993835 -3.10586834 -2.22387719

H -1.09167600 -4.12075186 -2.57359385

C -0.44853026 -2.50550842 -1.25548851

C -1.74846590 -0.58608437 -1.24333429

C -1.97613800 0.74177027 -0.70113540

C -0.27753183 2.73917651 1.82594109

C 0.73526347 -3.16688156 -0.58578247

C	-0.26052511	5.25555277	1.41317594
H	-1.21259522	5.67454624	1.78498876
C	0.90655488	6.17331362	1.79779422
H	1.77336478	5.96788740	1.14863706
H	0.63808191	7.23439932	1.69616854
C	1.20376885	5.75244141	3.24418068
H	0.45768896	6.18467808	3.93073773
H	2.20205665	6.05959988	3.58594298
C	1.06681609	4.22836876	3.19958496
H	2.00737834	3.73137975	2.90607023
H	0.72623736	3.77293205	4.14151382
C	2.59883189	-4.72289324	-0.45266628
H	2.25458860	-5.15542078	0.49868542
H	3.31758118	-3.91940141	-0.21688575
C	3.16949344	-5.74094152	-1.44337499
C	2.83851433	-5.12343454	-2.80959058
C	1.42138159	-4.57667828	-2.59727120
H	2.87442565	-5.84555387	-3.63757777
H	3.52866983	-4.29480410	-3.03768802
H	2.66311812	-6.71319628	-1.32878482
H	4.24662256	-5.89574194	-1.28989661
H	1.17201257	-3.74098349	-3.26666784
La	0.53446931	-0.52539086	1.51217866
N	-1.44549167	-1.63380980	3.39687562
O	-0.17043664	-1.52587581	3.67095280
O	-1.78940666	-1.20869005	2.20785618
O	-2.24121642	-2.08945274	4.17915297
N	3.17723560	0.34615192	0.50710952
O	2.09231472	0.45896161	-0.21476682
O	3.00091314	-0.17576624	1.69118333
O	4.26141262	0.69852668	0.10785739

H 0.67885017 -5.38456488 -2.72460151

H -0.33640340 5.07758474 0.33093241

L4*Ce(NO_3)$_3$(H_2O)

(H_2O in outer coordination sphere)

E= -11922.549498, E0 = -11922.099577, G= 229.4037 at T=298.150 K

Cl -4.86945105 3.67636013 -0.75528437

Cl -3.64236045 -3.24267578 -3.73543525

N -1.20732653 1.18852544 0.17770723

N 0.06300719 4.03236866 1.95875609

N -0.79049182 -1.25260735 -0.82314038

N 1.34082890 -4.10539913 -1.20406735

O 0.44861877 1.82004380 2.10374546

O 0.90905398 -2.72668719 0.53318846

C -1.35475147 2.41249156 0.69965512

C -2.49227238 3.20105839 0.43077672

H -2.64602780 4.16441965 0.91309321

C -3.45137262 2.71186876 -0.43480903

C -3.30018568 1.44247627 -1.05194604

C -4.21690416 0.86126655 -1.96317720

H -5.11133432 1.42301273 -2.23571920

C -3.99344802 -0.38946810 -2.49151444

H -4.70943451 -0.82640827 -3.18931699

C -2.84445596 -1.14678895 -2.14216280

C -2.55046248 -2.45396137 -2.62451029

C -1.41634309 -3.12419701 -2.20527220

H -1.22970116 -4.13998222 -2.55186391

C -0.54823685 -2.49310160 -1.29757595

C -1.89668512 -0.60426390 -1.22436166

C -2.12680149 0.71848196 -0.67475587

C -0.23470546 2.76101732 1.62764573

C 0.61776590 -3.12988400 -0.62506849

C	-0.36517102	5.27982903	1.29442739
H	-1.26435423	5.69361830	1.78738606
C	0.83515549	6.21334362	1.50193799
H	1.59529006	6.01472616	0.72859824
H	0.54518062	7.27279520	1.44559312
C	1.35772991	5.79074669	2.88303256
H	0.72351879	6.21755648	3.67783999
H	2.39545560	6.10455227	3.06595564
C	1.22518718	4.26496458	2.85333300
H	2.11231160	3.76913404	2.42293572
H	1.03958273	3.81063485	3.83858132
C	2.52275014	-4.63032818	-0.47420549
H	2.20090175	-5.18558168	0.42034355
H	3.13536716	-3.77811289	-0.13326137
C	3.23793697	-5.49458504	-1.51895154
C	2.87119460	-4.81509972	-2.84728074
C	1.39674580	-4.44540119	-2.63934016
H	3.01496124	-5.46009064	-3.72658205
H	3.46304750	-3.89473128	-2.98130107
H	2.85043597	-6.52700377	-1.49883926
H	4.32216024	-5.53123617	-1.33940268
H	1.07597244	-3.58918023	-3.25023961
Ce	0.93451172	-0.31085441	0.96484810
N	-0.55679941	-1.16525197	3.36767793
O	0.71666402	-0.97443110	3.36085153
O	-1.14135718	-0.95149773	2.21446514
O	-1.17735004	-1.51802802	4.34791613
N	3.73957849	-0.30990776	1.83184254
O	3.05933261	0.78108943	1.61704302
O	3.07395577	-1.40272903	1.63841307
O	4.90097427	-0.29552400	2.18112516

N 2.10564303 0.54868454 -1.61023080

O 2.60022235 0.90419650 -2.66031003

O 2.22208405 -0.66515851 -1.15861917

O 1.41526628 1.34518898 -0.85637599

H 0.74506849 -5.31142807 -2.86002946

H -0.58880079 5.09774685 0.23341271

O -3.85433435 -0.89013427 1.36111963

H -2.99395561 -0.96371859 1.83206701

H -4.23858690 -1.77676046 1.46963644

(NO_3^- in outer coordination sphere)

E= -11922.542964, E0 = -11922.093542, G= 230.4261 at T=298.150 K

Cl -5.14171219 3.32758212 -0.79864460

Cl -3.40777802 -3.61219645 -3.48094153

N -1.39745986 1.06006455 0.39618582

N -0.41226143 4.04711723 2.16453099

N -0.79120320 -1.34690917 -0.57626605

N 1.70289326 -3.83684993 -1.16867363

O 0.09788672 1.86535192 2.40103126

O 1.29545915 -2.46466756 0.57985944

C -1.63102698 2.31194925 0.85592043

C -2.78491688 3.02428460 0.49470270

H -3.00188470 4.00399733 0.91545457

C -3.69457936 2.44866157 -0.37887114

C -3.45549679 1.15801513 -0.92583317

C -4.30950689 0.49060309 -1.85080373

H -5.22136545 0.99789965 -2.16988897

C -4.00503254 -0.75551170 -2.33937287

H -4.66922903 -1.24880648 -3.05023980

C -2.81548262 -1.42387092 -1.93881512

C -2.40359426 -2.70347285 -2.38691187

C -1.20143390 -3.24934602 -1.97323930

H	-0.90042979	-4.23134232	-2.33172154
C	-0.40056869	-2.51950502	-1.07491684
C	-1.94280374	-0.79000193	-1.00621545
C	-2.26597881	0.51524448	-0.48488507
C	-0.60658848	2.75129747	1.84378183
C	0.93043917	-2.95804882	-0.51919663
C	-0.86650866	5.25252342	1.44413233
H	-1.84222865	5.59567022	1.83545911
C	0.22423986	6.28318214	1.76512194
H	1.07472217	6.15131235	1.07617784
H	-0.14572997	7.31447077	1.67000794
C	0.63199157	5.90668535	3.19731092
H	-0.11629377	6.27873516	3.91672373
H	1.61447430	6.30654716	3.48655295
C	0.62785059	4.37496948	3.16897225
H	1.59464228	3.95904493	2.83600378
H	0.38153937	3.90828705	4.13514233
C	3.02901697	-4.15539455	-0.58271360
H	2.90085793	-4.68617773	0.37335378
H	3.55451179	-3.20698094	-0.37808487
C	3.72386765	-4.97116375	-1.67561364
C	3.13394046	-4.38217592	-2.96434450
C	1.65466642	-4.18379450	-2.61358285
H	3.26025605	-5.03154659	-3.84290957
H	3.56914520	-3.39237809	-3.17485690
H	3.46592021	-6.03967476	-1.58263290
H	4.81780958	-4.87454557	-1.61529577
H	1.21027327	-3.35622597	-3.19096541
Ce	0.39055350	-0.40325338	1.56868470
N	2.63637209	-0.57923585	3.42687583
O	3.57845330	-0.65218413	4.18286562

O	2.74325705	-0.09486385	2.21860194
O	1.44121015	-0.96933514	3.74409652
O	1.59380007	0.73663753	-0.18309233
H	1.34200704	0.64514065	-1.24443841
H	2.55874324	0.59173918	-0.12631713
N	-1.80897093	-1.64990878	3.05189514
O	-1.01910245	-2.32444525	2.26827455
O	-1.57921648	-0.36973298	3.07727361
O	-2.69063616	-2.17036581	3.69944239
H	1.08873785	-5.12211227	-2.75543809
H	-0.96487647	5.04708767	0.36798796
N	1.42182255	-0.67271203	-2.95782757
O	0.84715462	-1.29421091	-3.87374306
O	0.88553172	0.45173907	-2.54184318
O	2.46985054	-1.08289313	-2.40328646

L4*Ce$(NO_3)_2^+$

E= -11565.572850, E0 = -11565.161665, G= 211.0802 at T=298.150 K

Cl	-4.54329586	3.82554245	-1.08012652
Cl	-3.34653997	-3.13205433	-3.92779016
N	-1.07949114	1.20096397	0.19028138
N	0.06126584	3.98259068	2.17093253
N	-0.65948457	-1.24573123	-0.80945504
N	1.45306206	-4.14244318	-1.14561689
O	0.38721523	1.75671482	2.26710820
O	1.13795817	-2.61603022	0.47927701
C	-1.24009430	2.43851423	0.69545859
C	-2.30660057	3.26488924	0.31725493
H	-2.46583557	4.23817015	0.77524614
C	-3.21230006	2.80967426	-0.63528496
C	-3.04981947	1.53100610	-1.22265625
C	-3.91688657	0.98134291	-2.21568441

H	-4.76017809	1.58148658	-2.56081533
C	-3.70193505	-0.26803076	-2.72683740
H	-4.37163067	-0.67707497	-3.48488259
C	-2.60318017	-1.06671202	-2.28525567
C	-2.32136822	-2.37284040	-2.75555348
C	-1.21834171	-3.06973290	-2.27340341
H	-1.03655434	-4.08152199	-2.62789130
C	-0.39775354	-2.46812701	-1.31008387
C	-1.72120452	-0.55234343	-1.29439950
C	-1.94700491	0.76183879	-0.75715548
C	-0.22283173	2.74514866	1.75679636
C	0.78083223	-3.10776544	-0.63456368
C	-0.33311993	5.28435183	1.58156252
H	-1.30680752	5.60444403	1.99351609
C	0.77972221	6.22958040	2.05102324
H	1.64786625	6.14905214	1.37673557
H	0.44365335	7.27607489	2.06242967
C	1.12496662	5.68250608	3.44330549
H	0.36655107	5.99649096	4.17876387
H	2.10919714	6.01278973	3.80373740
C	1.07725608	4.16540623	3.24453354
H	2.04035878	3.75768948	2.89243960
H	0.77940851	3.59848881	4.13917780
C	2.59511328	-4.70824862	-0.37504372
H	2.25253987	-5.02013731	0.62289125
H	3.35488963	-3.91954398	-0.23914631
C	3.09614301	-5.85341120	-1.25876760
C	2.76412535	-5.37189674	-2.67820549
C	1.38172698	-4.73076534	-2.50431895
H	2.74418211	-6.18103504	-3.42192602
H	3.48999262	-4.61136532	-3.00915956

H 2.54835486 -6.78193474 -1.02972257

H 4.16822720 -6.04196310 -1.10783327

H 1.16572821 -3.95354939 -3.25148296

Ce 0.58014679 -0.42538595 1.28496373

N -1.25539601 -1.53174973 3.22567868

O 0.03670657 -1.38845372 3.41842604

O -1.66605747 -1.12537146 2.05635309

O -1.98743463 -1.99699581 4.05806923

N 3.07466030 0.48320404 0.12469640

O 1.94608831 0.55956757 -0.52678245

O 2.97011256 -0.03244285 1.32451630

O 4.12495422 0.85382789 -0.33366522

H 0.59365088 -5.50407028 -2.53879809

H -0.40551013 5.20737267 0.48713359

L4*Pr(NO_3)$_3$(H_2O)

(H_2O in outer coordination sphere)

E= -12299.618839, E0 = -12299.168413, G= 229.4959 at T=298.150 K

Cl -4.87759018 3.66419935 -0.74198043

Cl -3.70807505 -3.28846693 -3.65770674

N -1.24044621 1.14925623 0.22293910

N 0.10597614 3.99943829 1.89623737

N -0.83983701 -1.29369819 -0.76534235

N 1.36177647 -4.04263115 -1.23273408

O 0.34152770 1.78456628 2.23478580

O 0.81393272 -2.84878922 0.60912466

C -1.37947667 2.37636995 0.72509563

C -2.50052333 3.17986822 0.44566068

H -2.63298821 4.15616941 0.90925437

C -3.47171187 2.68713880 -0.40977243

C -3.33269429 1.41002262 -1.00394261

C -4.26333523 0.82148385 -1.90766847

H	-5.15507603	1.38803554	-2.17979860
C	-4.05223370	-0.42862794	-2.42439008
H	-4.77392387	-0.86808968	-3.11485600
C	-2.89833951	-1.19139099	-2.07597923
C	-2.61311388	-2.49503112	-2.55487704
C	-1.47073066	-3.16333890	-2.14299583
H	-1.27403009	-4.17633915	-2.49359560
C	-0.60495561	-2.52438712	-1.24179864
C	-1.94751000	-0.64380825	-1.16781497
C	-2.16715765	0.68076730	-0.62730932
C	-0.25762731	2.72406340	1.66882825
C	0.58157855	-3.16622305	-0.57931256
C	-0.24102873	5.20558786	1.11773169
H	-1.10663188	5.72372580	1.57058299
C	1.02216458	6.06849861	1.23867393
H	1.76518023	5.73935699	0.49379364
H	0.80818212	7.13492775	1.07535398
C	1.51425183	5.74962044	2.65865517
H	0.91371483	6.29913855	3.40264606
H	2.57214761	6.00504351	2.81497169
C	1.27348745	4.24142885	2.78250599
H	2.12501383	3.64108658	2.41839314
H	1.04665852	3.90465236	3.80560303
C	2.57670331	-4.54698706	-0.54123026
H	2.29284620	-5.22553587	0.27795324
H	3.10614395	-3.68827891	-0.09393704
C	3.37676334	-5.22358227	-1.66094100
C	2.95493317	-4.44455004	-2.91650891
C	1.45115662	-4.23896599	-2.69370294
H	3.16478825	-4.97644663	-3.85613036
H	3.45469570	-3.46229315	-2.94230509

H 3.09120321 -6.28466988 -1.75606155

H 4.45843458 -5.17518616 -1.46891737

H 1.05272114 -3.36332750 -3.22560334

Pr 0.86894482 -0.38740110 1.10097980

N -0.74328035 -1.21383703 3.43094373

O 0.52365166 -1.02195096 3.50242066

O -1.25885344 -1.02669096 2.23667717

O -1.42968547 -1.54559422 4.37602615

N 3.62020659 -0.46009403 2.09343028

O 2.96685028 0.65054196 1.91919672

O 2.95117044 -1.53224254 1.80198348

O 4.76602221 -0.48919401 2.49395251

N 2.09285069 0.49792060 -1.44178462

O 2.59656286 0.86640668 -2.48618793

O 2.15044022 -0.73508072 -1.03871989

O 1.45205295 1.30393231 -0.65524137

H 0.88478976 -5.13834667 -2.99941754

H -0.48334366 4.94097519 0.07857906

O -3.92966533 -0.91918057 1.33861399

H -3.07462192 -1.00110149 1.82167101

H -4.37103701 -1.76286447 1.53529882

(NO_3^- in outer coordination sphere)

E= -12299.613180, E0 = -12299.162519, G= 230.9470 at T=298.150 K

Cl -5.16419220 3.29152536 -0.71620572

Cl -3.38556194 -3.58216119 -3.51640177

N -1.39764857 1.04523754 0.41856086

N -0.39024690 4.02104568 2.15979218

N -0.77847570 -1.35309815 -0.57166177

N 1.71174467 -3.83995199 -1.17737794

O 0.08821510 1.84057403 2.46030164

O 1.27078259 -2.52044702 0.60725003

C	-1.63988590	2.27284575	0.89794111
C	-2.80403757	2.98599911	0.56325662
H	-3.02735519	3.95304418	1.00968397
C	-3.70786095	2.41983938	-0.32128337
C	-3.45580339	1.14816082	-0.89643031
C	-4.30608940	0.49059159	-1.83485842
H	-5.22073984	0.99546826	-2.14921761
C	-3.98915267	-0.74335980	-2.33646536
H	-4.64715624	-1.23238587	-3.05636501
C	-2.79521680	-1.41559386	-1.94093812
C	-2.38431621	-2.69036579	-2.40663934
C	-1.18328595	-3.23885059	-1.99507344
H	-0.87671387	-4.21450663	-2.36671829
C	-0.38794643	-2.52121353	-1.08352292
C	-1.92684627	-0.79457957	-1.00234342
C	-2.26005650	0.50613451	-0.46877265
C	-0.59585947	2.71719003	1.88620424
C	0.93151516	-2.97739577	-0.51466316
C	-0.83399457	5.20727587	1.39931762
H	-1.79132724	5.58960724	1.79924822
C	0.28844365	6.22226524	1.65355325
H	1.12227952	6.03542089	0.95710713
H	-0.05683975	7.25742483	1.51686406
C	0.71248990	5.90084410	3.09440136
H	-0.01325440	6.32392263	3.80865479
H	1.70948029	6.28854132	3.34812641
C	0.67149740	4.36988258	3.13573909
H	1.62178934	3.91398668	2.80842686
H	0.42722178	3.95219612	4.12453651
C	3.02728772	-4.17997789	-0.58085471
H	2.88392544	-4.75513363	0.34711698

H 3.54418468 -3.24026752 -0.32112917

C 3.74770617 -4.94235754 -1.69600642

C 3.17448831 -4.30434418 -2.96911407

C 1.68855131 -4.13033962 -2.63515449

H 3.32047415 -4.91641521 -3.87117648

H 3.60574293 -3.30391574 -3.13258791

H 3.49771070 -6.01593494 -1.65321505

H 4.83962774 -4.83978605 -1.61264980

H 1.24502194 -3.28392482 -3.18664765

Pr 0.37157157 -0.47855127 1.62688589

N 2.59809208 -0.55074131 3.50236440

O 3.52519178 -0.58661807 4.28136826

O 2.72147441 -0.10769025 2.28445363

O 1.40056515 -0.94737893 3.81349230

O 1.57135105 0.77050138 -0.10178254

H 1.31128800 0.69246662 -1.13168561

H 2.52975392 0.58372766 -0.06705199

N -1.93560934 -1.66358066 2.98946643

O -1.10946286 -2.35963392 2.26350927

O -1.69510317 -0.38727531 3.01448369

O -2.86146641 -2.16750288 3.58896756

H 1.13351655 -5.06759739 -2.82196856

H -0.96168870 4.96278334 0.33489335

N 1.33649051 -0.61935502 -2.90301800

O 0.75182527 -1.24969697 -3.81204963

O 0.81322402 0.50201648 -2.48837113

O 2.38867044 -1.03859091 -2.35656548

L4*Pr(NO$_3$)$_2^+$

E= -11942.646743, E0 = -11942.234687, G= 213.1257 at T=298.150 K

Cl -4.53877211 3.81441259 -1.06147969

Cl -3.34564376 -3.13799787 -3.90612936

N	-1.08117414	1.19405973	0.21344189
N	0.06908377	3.97814202	2.17635369
N	-0.66272318	-1.26162171	-0.78722042
N	1.45690250	-4.14424133	-1.13578653
O	0.37157443	1.75094914	2.31909442
O	1.11970460	-2.65915680	0.52385163
C	-1.24395728	2.42285132	0.72532034
C	-2.31310010	3.25099921	0.34803784
H	-2.47703958	4.22051144	0.81302470
C	-3.20823812	2.80099702	-0.61238205
C	-3.03786635	1.52603495	-1.20922804
C	-3.89532208	0.98331213	-2.21399117
H	-4.73241377	1.58745742	-2.56753373
C	-3.68089318	-0.26592749	-2.72498083
H	-4.34470844	-0.67027634	-3.49068284
C	-2.59249759	-1.07135010	-2.27114630
C	-2.31940055	-2.38420081	-2.73235035
C	-1.22773027	-3.08696246	-2.24190784
H	-1.05144250	-4.10266066	-2.58880854
C	-0.40611926	-2.48371458	-1.27642977
C	-1.71202528	-0.56452906	-1.27496243
C	-1.93819141	0.75750577	-0.73509264
C	-0.22227576	2.73210406	1.78975677
C	0.77602100	-3.12736487	-0.59804899
C	-0.31120384	5.27050352	1.55744386
H	-1.27962887	5.61306953	1.96385360
C	0.81372964	6.21313286	2.00321865
H	1.68058038	6.10466528	1.33118629
H	0.49107823	7.26382780	1.98876798
C	1.15219462	5.69555902	3.40834045
H	0.39725885	6.03604174	4.13558531

H 2.14004064 6.02310896 3.76131940

C 1.08705044 4.17481184 3.24543142

H 2.04541588 3.74792194 2.90323114

H 0.78292751 3.63205385 4.15288591

C 2.60517097 -4.71548605 -0.37873518

H 2.26549530 -5.05642605 0.61066085

H 3.35494304 -3.92108321 -0.22182316

C 3.12119508 -5.83138752 -1.29080820

C 2.78525209 -5.31703711 -2.69775844

C 1.39407372 -4.69916916 -2.50905228

H 2.77721834 -6.10628939 -3.46279931

H 3.50135779 -4.53828716 -3.00689721

H 2.58407617 -6.77197933 -1.08708024

H 4.19520235 -6.01137495 -1.14314997

H 1.16865432 -3.90759397 -3.23808479

Pr 0.57670742 -0.45161539 1.34403181

N -1.34477150 -1.53983855 3.20054746

O -0.05912225 -1.41081643 3.44037366

O -1.71278381 -1.12749112 2.02084827

O -2.10932422 -2.00141835 4.00712061

N 3.01942706 0.48873571 0.12104714

O 1.87863588 0.56266874 -0.50476223

O 2.94784021 -0.03850502 1.31934321

O 4.05813599 0.87239653 -0.35588703

H 0.61762303 -5.48303270 -2.56417060

H -0.38620195 5.17100096 0.46505219

L4*Nd(NO_3)$_3$(H_2O)

(H_2O in outer coordination sphere)

E= -12686.521627, E0 = -12686.070821, G= 229.6118 at T=298.150 K

Cl -4.87008715 3.66031504 -0.73161685

Cl -3.76875257 -3.32681537 -3.59673119

N	-1.25004280	1.12002933	0.23800462
N	0.14445066	3.97984600	1.84641087
N	-0.88470292	-1.33243763	-0.73057896
N	1.36649179	-4.00161314	-1.23471785
O	0.31380722	1.77249753	2.27738261
O	0.73552710	-2.93785238	0.66408753
C	-1.37277055	2.35454440	0.72374189
C	-2.48723388	3.16596842	0.44344866
H	-2.60237002	4.15086222	0.89419866
C	-3.47218490	2.67315245	-0.39703500
C	-3.34976578	1.38668358	-0.97414821
C	-4.29472494	0.79409862	-1.86544883
H	-5.18445158	1.36585522	-2.13380218
C	-4.09984779	-0.45943174	-2.37417698
H	-4.83080959	-0.89775109	-3.05548668
C	-2.94613171	-1.22749460	-2.02919912
C	-2.66646314	-2.53170824	-2.50358558
C	-1.52258801	-3.20162964	-2.09783340
H	-1.32376242	-4.21429682	-2.44921565
C	-0.65336204	-2.55790710	-1.20122564
C	-1.98816180	-0.67857349	-1.13021934
C	-2.18976665	0.65341175	-0.59867734
C	-0.24748802	2.70447826	1.66812754
C	0.54575694	-3.19747066	-0.54111582
C	-0.15457056	5.15442944	1.00260150
H	-1.00042903	5.72984791	1.42271972
C	1.14102209	5.97282934	1.08209717
H	1.87324607	5.57186604	0.36218500
H	0.97045469	7.03593349	0.85729444
C	1.61403596	5.71644068	2.52125120
H	1.03338206	6.33171511	3.22870541

H	2.68080544	5.93776989	2.66925168
C	1.31232214	4.22831488	2.72991920
H	2.14202547	3.57420802	2.41099000
H	1.06390703	3.96219659	3.76875281
C	2.60090876	-4.48593187	-0.56248778
H	2.34838796	-5.24790573	0.19106165
H	3.06628418	-3.63584280	-0.03470168
C	3.46070719	-5.01564169	-1.71748686
C	2.99830675	-4.17543793	-2.91849279
C	1.48169601	-4.09397364	-2.70463991
H	3.25613523	-4.61900187	-3.89143610
H	3.42446089	-3.16039944	-2.86137676
H	3.25588894	-6.08441401	-1.89721525
H	4.53353071	-4.90312672	-1.50372112
H	1.02713335	-3.21566725	-3.18416715
Nd	0.83718175	-0.43214339	1.17912841
N	-0.85527730	-1.28487432	3.43459487
O	0.41627881	-1.15199757	3.53453732
O	-1.34404230	-0.99728251	2.24967575
O	-1.57034755	-1.64729571	4.34753942
N	3.55395603	-0.57084668	2.21804261
O	2.89135432	0.54397625	2.11036897
O	2.90134072	-1.62604356	1.84579456
O	4.69506168	-0.61422867	2.63285494
N	2.07805538	0.50156212	-1.33778441
O	2.59132099	0.86666679	-2.38044453
O	2.09378242	-0.74367625	-0.95910174
O	1.47579741	1.31273317	-0.53431487
H	0.98332232	-5.00930977	-3.07448053
H	-0.40442881	4.84388256	-0.02195549
O	-3.99284291	-0.96879971	1.30973792

H -3.14272285 -1.02025461 1.80706108

H -4.43799782 -1.79703641 1.55705345

(NO_3^- in outer coordination sphere)

E= -12686.517753, E0 = -12686.066532, G= 231.4654 at T=298.150 K

Cl -5.18136549 3.26687717 -0.69776183

Cl -3.39267588 -3.61195588 -3.46663523

N -1.40425694 1.04813278 0.42498451

N -0.38504347 4.02298498 2.14067411

N -0.76977414 -1.35490763 -0.55682242

N 1.72663987 -3.82648921 -1.18731582

O 0.05827362 1.84178388 2.49866295

O 1.29126787 -2.52953839 0.61695021

C -1.65058184 2.26586032 0.90800393

C -2.81917691 2.97648931 0.57662195

H -3.04520512 3.94311833 1.02307618

C -3.72009468 2.40361524 -0.30569911

C -3.46399426 1.13386095 -0.87993199

C -4.31582642 0.46854806 -1.81286335

H -5.23380947 0.96704924 -2.12739468

C -3.99337912 -0.76533502 -2.30691266

H -4.65092325 -1.26206791 -3.02209044

C -2.79391837 -1.43207872 -1.91199291

C -2.38488626 -2.71006632 -2.37126517

C -1.17965269 -3.25127101 -1.96431446

H -0.87129593 -4.22841167 -2.33108211

C -0.37974894 -2.52357912 -1.06428337

C -1.92137134 -0.80394024 -0.98436117

C -2.26195312 0.50009555 -0.45625740

C -0.60316145 2.71448779 1.90119243

C 0.94855827 -2.97518587 -0.50450873

C -0.80887538 5.19293165 1.34372461

H	-1.75902796	5.60395098	1.73219097
C	0.33135605	6.19533491	1.56686497
H	1.16208708	5.97117329	0.87783325
H	0.00470492	7.23158264	1.39673686
C	0.74855298	5.91286325	3.01792479
H	0.03122517	6.37285471	3.71774673
H	1.75274062	6.28943014	3.25956368
C	0.67899334	4.38492775	3.10947490
H	1.62148964	3.90052414	2.80134463
H	0.42321366	4.00475836	4.11042452
C	3.05061507	-4.16333675	-0.60833359
H	2.92174840	-4.76088190	0.30758446
H	3.55807257	-3.22360992	-0.33064792
C	3.77216387	-4.89202452	-1.74528146
C	3.17989063	-4.23504448	-2.99995708
C	1.69446385	-4.08881426	-2.65017748
H	3.32692671	-4.82562160	-3.91610885
H	3.59576583	-3.22531676	-3.14515114
H	3.53693867	-5.96957207	-1.72379184
H	4.86332512	-4.77679110	-1.66857660
H	1.23427320	-3.23829365	-3.18127227
Nd	0.37640455	-0.49289730	1.66679537
N	2.54059768	-0.65475196	3.57988024
O	3.45357728	-0.72023350	4.37438583
O	2.69664478	-0.18210806	2.37385559
O	1.33466208	-1.04314291	3.85881662
O	1.57571363	0.79589742	-0.03418800
H	1.31226587	0.72894740	-1.05851924
H	2.53227806	0.60001040	-0.00297226
N	-1.95223212	-1.68707693	2.96135378
O	-1.10648358	-2.38518429	2.26705766

O -1.70998669 -0.40921888 2.99358988

O -2.90170670 -2.18471646 3.53072548

H 1.15146589 -5.03025675 -2.85136056

H -0.94112968 4.91933346 0.28703868

N 1.29216075 -0.56558573 -2.85249114

O 0.68216085 -1.19343424 -3.74792600

O 0.78287959 0.55446327 -2.42128849

O 2.35743904 -0.98914617 -2.33554292

L4*Nd(NO_3)$_2^+$

E= -12329.550857, E0 = -12329.138320, G= 212.3969 at T=298.150 K

Cl -4.53976870 3.81310391 -1.05924988

Cl -3.35926461 -3.14432931 -3.89125705

N -1.09267735 1.18882251 0.22257991

N 0.07448998 3.96753454 2.17475224

N -0.66332674 -1.26130056 -0.79185593

N 1.45218742 -4.14133167 -1.12673390

O 0.34830678 1.73842609 2.34444118

O 1.09209526 -2.67735076 0.54735243

C -1.25183630 2.41490912 0.73439205

C -2.31819773 3.24734497 0.35489672

H -2.48072100 4.21712208 0.82017916

C -3.21040034 2.79976010 -0.60827076

C -3.03907776 1.52587581 -1.20815456

C -3.89542937 0.98403317 -2.21433806

H -4.73132324 1.58897018 -2.56888127

C -3.68336225 -0.26675457 -2.72282100

H -4.34854460 -0.67123860 -3.48721790

C -2.59617209 -1.07336676 -2.26801276

C -2.32759643 -2.38964534 -2.72361135

C -1.23968375 -3.09435987 -2.22994590

H -1.06707323 -4.11339664 -2.56955719

C	-0.41388386	-2.48605275	-1.26991916
C	-1.71204436	-0.56653792	-1.27497983
C	-1.94088495	0.75706661	-0.73107260
C	-0.22842354	2.72014260	1.80290139
C	0.76464868	-3.13299179	-0.58199775
C	-0.29029909	5.25645876	1.53880405
H	-1.25363839	5.61660004	1.94205391
C	0.84717339	6.19035244	1.97117782
H	1.71180880	6.06106520	1.29996300
H	0.53815955	7.24482632	1.94170725
C	1.18014610	5.68792152	3.38303447
H	0.43069583	6.04827118	4.10634518
H	2.17264962	6.00730419	3.73032594
C	1.09492385	4.16600323	3.24132085
H	2.04726696	3.72126842	2.90518808
H	0.78323811	3.64010000	4.15609312
C	2.59766340	-4.71671629	-0.36841655
H	2.25091600	-5.07752848	0.61146510
H	3.33878708	-3.91877937	-0.19031110
C	3.13161612	-5.81247234	-1.29468417
C	2.80194449	-5.27860737	-2.69596338
C	1.40341425	-4.67730427	-2.50840187
H	2.80778575	-6.05540657	-3.47366643
H	3.51279902	-4.48795319	-2.98648787
H	2.60218978	-6.76145983	-1.11079299
H	4.20615959	-5.98461723	-1.14181840
H	1.17445695	-3.87832189	-3.22806859
Nd	0.57395750	-0.46176621	1.36684608
N	-1.36511087	-1.57908010	3.17813373
O	-0.09417091	-1.41739428	3.45551705
O	-1.70157611	-1.19134462	1.98050272

O -2.14809632 -2.04610729 3.96501756

N 2.98963451 0.53961110 0.14738575

O 1.83188009 0.65669149 -0.43859038

O 2.94660640 -0.05989091 1.30983174

O 4.01701164 0.94805175 -0.33601394

H 0.63559991 -5.46834373 -2.57904792

H -0.36762846 5.14411497 0.44793117

L4*Pm(NO_3)$_3$(H_2O)

(H_2O in outer coordination sphere)

E= -13083.384056, E0 = -13082.933207, G= 232.1081 at T=298.150 K

Cl -4.90932274 3.64834094 -0.83989012

Cl -3.69141531 -3.38371086 -3.55224562

N -1.29265797 1.15450549 0.24835126

N 0.02743386 3.93065548 1.97498667

N -0.85839134 -1.28791618 -0.71133274

N 1.45256591 -3.94235659 -1.27749944

O -0.09094303 1.74238586 2.56633186

O 1.04263628 -2.65013266 0.52302581

C -1.46952617 2.37109852 0.76201510

C -2.57488847 3.17536116 0.43659610

H -2.70755005 4.15814924 0.88864285

C -3.51809478 2.67468739 -0.44665402

C -3.36608028 1.38413656 -1.01112211

C -4.28574419 0.76961553 -1.91418004

H -5.17868853 1.32607830 -2.20346117

C -4.06558514 -0.48651367 -2.40660644

H -4.78140831 -0.94335330 -3.09131384

C -2.90613699 -1.23354697 -2.03548622

C -2.60147095 -2.54175639 -2.48289514

C -1.43743145 -3.17431688 -2.07686114

H -1.22287524 -4.18627262 -2.41832781

C	-0.57508689	-2.49590421	-1.19498837
C	-1.96788704	-0.66099113	-1.13180554
C	-2.20232916	0.66996562	-0.61295158
C	-0.44955635	2.68451977	1.83287406
C	0.70181024	-3.05051422	-0.60640699
C	0.00172479	4.99169254	0.94884372
H	-0.71421444	5.78478765	1.23467243
C	1.44268739	5.51745415	0.97170365
H	2.07909274	4.82955265	0.39125475
H	1.52212524	6.52936125	0.55032092
C	1.79676402	5.45024586	2.46652699
H	1.36091936	6.30959082	3.00297904
H	2.88058138	5.44529533	2.64665484
C	1.15237236	4.13506413	2.92841148
H	1.83375871	3.27112532	2.84537244
H	0.77130717	4.16613817	3.96083045
C	2.73933840	-4.35955477	-0.66126657
H	2.54939008	-4.93024874	0.25909233
H	3.30361056	-3.45445204	-0.37816194
C	3.42379189	-5.17057085	-1.76798582
C	2.87805963	-4.53901100	-3.05674052
C	1.40322578	-4.28354502	-2.71354055
H	2.98982501	-5.17949629	-3.94260073
H	3.38027477	-3.57795954	-3.25469255
H	3.12798333	-6.23164797	-1.70725024
H	4.51948357	-5.11511707	-1.69179118
H	0.96161765	-3.46060300	-3.29431581
Pm	0.77765888	-0.24273492	1.20458996
N	-0.83038503	-1.46883607	3.33251882
O	0.41661471	-1.20784438	3.47182226
O	-1.32600522	-1.16286302	2.15529609

O -1.51961517 -1.95561099 4.20714760

N 3.50094748 -0.11054466 2.21947169

O 2.55996656 0.64307147 2.67940760

O 3.12558675 -0.94662374 1.29091918

O 4.64961720 -0.05275642 2.61366987

N 1.85941672 1.35869348 -1.02458274

O 2.32259631 2.01886630 -1.93849206

O 1.54569221 0.11232580 -1.15642738

O 1.63912129 1.86279380 0.15069838

H 0.80480158 -5.19971800 -2.87395048

H -0.28844821 4.57757378 -0.02668540

O -3.96284842 -1.20418942 1.20206439

H -3.12027073 -1.26203156 1.71070492

H -4.62954140 -1.53988814 1.82471406

(NO_3^- in outer coordination sphere)

E= -13083.380097, E0 = -13082.928647, G= 232.1472 at T=298.150 K

Cl -5.17671490 3.26278281 -0.67236203

Cl -3.40457749 -3.59702873 -3.50422740

N -1.40092635 1.02711427 0.42036197

N -0.37154952 3.98761225 2.15354156

N -0.77954435 -1.37015283 -0.57681942

N 1.71407688 -3.84301758 -1.20996404

O 0.06055341 1.80340505 2.50970888

O 1.28181505 -2.53986454 0.59014434

C -1.64293909 2.24003363 0.91512775

C -2.81256771 2.95510101 0.59483135

H -3.03563595 3.91710591 1.05277085

C -3.71481133 2.39458346 -0.29341510

C -3.46084499 1.13134098 -0.88291389

C -4.31318188 0.47748172 -1.82379961

H -5.22859955 0.98250932 -2.13549352

C	-3.99525452	-0.75325698	-2.32889867
H	-4.65388870	-1.24057937	-3.04948878
C	-2.79984140	-1.42889535	-1.93658423
C	-2.39498186	-2.70662141	-2.40130329
C	-1.19416726	-3.25744629	-1.99357414
H	-0.89013183	-4.23422384	-2.36488008
C	-0.39248028	-2.53807783	-1.08817136
C	-1.92668879	-0.81093258	-1.00327754
C	-2.26093459	0.49080533	-0.46514151
C	-0.59248483	2.67908955	1.91128373
C	0.93691206	-2.98997188	-0.52951759
C	-0.78708202	5.15910149	1.35501623
H	-1.73273802	5.57962799	1.74469984
C	0.36211076	6.15235186	1.57352734
H	1.19017684	5.91783714	0.88466507
H	0.04502385	7.19101048	1.39986217
C	0.77796990	5.87039757	3.02511215
H	0.06418089	6.33794498	3.72352290
H	1.78502190	6.24013805	3.26549268
C	0.69618046	4.34327936	3.12057853
H	1.63433254	3.84979200	2.81353879
H	0.43835267	3.96757412	4.12271547
C	3.04036450	-4.17461824	-0.63277709
H	2.91383028	-4.76412725	0.28862447
H	3.54812574	-3.23205519	-0.36551809
C	3.75728869	-4.91283703	-1.76610970
C	3.16502452	-4.26081800	-3.02299643
C	1.68002808	-4.10882473	-2.67226100
H	3.30964804	-4.85467672	-3.93736196
H	3.58260512	-3.25233388	-3.17218041
H	3.51789021	-5.98932314	-1.73727047

H 4.84901714 -4.80142546 -1.69196391

H 1.22417068 -3.25599456 -3.20405340

Pm 0.38049844 -0.51554042 1.63029444

N 2.42481303 -0.37356156 3.65905333

O 3.28750157 -0.27311307 4.50600433

O 2.63328695 -0.04958897 2.41134000

O 1.23150945 -0.80243754 3.91391897

O 1.56651592 0.82782370 -0.03129913

H 1.33552468 0.73975086 -1.05869019

H 2.53214502 0.70538270 0.04391146

N -1.87978148 -1.73919284 2.98566484

O -0.95249271 -2.43286180 2.39838362

O -1.74144268 -0.45136312 2.88466144

O -2.80925012 -2.25234890 3.57407546

H 1.13336408 -5.04862642 -2.87114382

H -0.92420965 4.88545752 0.29896829

N 1.30619323 -0.58191168 -2.84909058

O 0.68545806 -1.21193182 -3.73791552

O 0.81133872 0.54718959 -2.42946196

O 2.36689472 -1.01404381 -2.33147717

L4*Pm(NO$_3$)$_2$$^+$

E= -12726.415740, E0 = -12726.003075, G= 212.6284 at T=298.150 K

Cl -4.53627205 3.81415892 -1.06143916

Cl -3.34444571 -3.13740349 -3.90188885

N -1.08750236 1.19282007 0.22079812

N 0.06906384 3.96612597 2.18604565

N -0.66633338 -1.26391327 -0.78283048

N 1.45659637 -4.13992357 -1.12186611

O 0.34820330 1.73617625 2.34554052

O 1.10240173 -2.66843748 0.54688865

C -1.25185490 2.41576815 0.73816049

C	-2.31941009	3.24700046	0.35912761
H	-2.48575258	4.21424866	0.82830691
C	-3.20732093	2.80117464	-0.60835153
C	-3.03303194	1.52876687	-1.21085382
C	-3.88466930	0.98947072	-2.22236013
H	-4.71814871	1.59588277	-2.58004093
C	-3.67042542	-0.26013958	-2.73290968
H	-4.33107424	-0.66206843	-3.50254750
C	-2.58764672	-1.06930029	-2.27231336
C	-2.31830215	-2.38564324	-2.72746611
C	-1.23323286	-3.09166884	-2.23006535
H	-1.05933595	-4.10971546	-2.57153916
C	-0.41197091	-2.48629093	-1.26394141
C	-1.70904183	-0.56567299	-1.27293336
C	-1.93627059	0.75961053	-0.73148614
C	-0.22991489	2.71896887	1.80900955
C	0.77138853	-3.12888932	-0.57847393
C	-0.29875699	5.25662994	1.55497658
H	-1.26561034	5.61018705	1.95551455
C	0.83190209	6.19404411	1.99739814
H	1.70019627	6.07424450	1.32923460
H	0.51665825	7.24683666	1.97395587
C	1.16169465	5.68455505	3.40767074
H	0.40619886	6.03532934	4.12940216
H	2.15028834	6.00799656	3.76223445
C	1.08662450	4.16317654	3.25548029
H	2.04253173	3.72762895	2.91738057
H	0.77741504	3.62816453	4.16571665
C	2.60451531	-4.71159124	-0.36453924
H	2.26248288	-5.06069899	0.62114841
H	3.34991956	-3.91477227	-0.19935443

C 3.12915373 -5.81810284 -1.28304684

C 2.79834366 -5.29326344 -2.68741441

C 1.40327084 -4.68356991 -2.50047064

H 2.79787302 -6.07588243 -3.45928836

H 3.51248765 -4.50862312 -2.98587513

H 2.59441161 -6.76227379 -1.08989465

H 4.20304537 -5.99539948 -1.13146603

H 1.17771387 -3.88737535 -3.22430372

Pm 0.57513154 -0.44982731 1.34317219

N -1.33950901 -1.52656651 3.18049479

O -0.05816809 -1.40646267 3.42434287

O -1.69855177 -1.10741138 2.00051689

O -2.11336875 -1.98536193 3.98152781

N 2.96937084 0.48456785 0.06742837

O 1.81652498 0.53536052 -0.53592616

O 2.92432952 -0.01964417 1.27392340

O 3.99570894 0.86850679 -0.43799171

H 0.63128328 -5.47099543 -2.56567788

H -0.37080756 5.14948654 0.46315852

L4*Sm(NO_3)$_3$(H_2O)

(H_2O in outer coordination sphere)

E= -13490.321048, E0 = -13489.870300, G= 229.4138 at T=298.150 K

Cl -4.85492325 3.67828751 -0.77527303

Cl -3.78973222 -3.35090709 -3.54380679

N -1.22432649 1.15158689 0.19503006

N 0.13379863 4.01453114 1.83757234

N -0.88431388 -1.32418096 -0.72230202

N 1.35784960 -4.00697899 -1.22381592

O 0.30630800 1.80453920 2.26012850

O 0.77242875 -2.89163923 0.66035801

C -1.35363960 2.38503790 0.68117887

C	-2.47132087	3.19214153	0.40077204
H	-2.58836937	4.17796135	0.84894544
C	-3.45665407	2.69366980	-0.43517438
C	-3.33701897	1.40123165	-0.99932516
C	-4.28906965	0.79902250	-1.87663174
H	-5.17933226	1.36898994	-2.14690733
C	-4.10008764	-0.46164995	-2.36936355
H	-4.83584309	-0.90778679	-3.04035378
C	-2.94762707	-1.22857368	-2.01789665
C	-2.67677760	-2.54200697	-2.47156620
C	-1.53158855	-3.20774412	-2.06452775
H	-1.33717501	-4.22553015	-2.40316653
C	-0.65562552	-2.55383086	-1.18118894
C	-1.98315799	-0.67086977	-1.13155603
C	-2.17393708	0.67277467	-0.62355185
C	-0.24308428	2.73579001	1.64286304
C	0.55572456	-3.18064499	-0.53167117
C	-0.14552598	5.18761301	0.98530275
H	-1.00398350	5.76147842	1.38147497
C	1.14487815	6.01030064	1.09974706
H	1.89823866	5.61069679	0.40116969
H	0.97755432	7.07261372	0.86879498
C	1.57757854	5.75692225	2.55207396
H	0.97455621	6.37097836	3.24163485
H	2.63871980	5.98258686	2.73079991
C	1.27473128	4.26793003	2.75380397
H	2.11548042	3.61594272	2.46096087
H	0.99741411	4.00271177	3.78558564
C	2.60653758	-4.47397804	-0.56617421
H	2.36921787	-5.20225477	0.22454646
H	3.09374356	-3.60699296	-0.08767737

C 3.42977428 -5.05560398 -1.72190785

C 2.95389843 -4.24366331 -2.93671227

C 1.44339514 -4.13162470 -2.69365072

H 3.18373442 -4.71886635 -3.90173507

H 3.39729428 -3.23465061 -2.91882157

H 3.20093799 -6.12526560 -1.86365604

H 4.50917864 -4.95711517 -1.53616846

H 0.99542248 -3.25525498 -3.18292165

Sm 0.85586876 -0.39164019 1.16402018

N -0.77631652 -1.49500561 3.32523298

O 0.50464475 -1.51302946 3.34643149

O -1.29904151 -0.89294505 2.28364277

O -1.47206879 -1.99167669 4.19012117

N 3.51242566 -0.49051470 2.32785726

O 2.70655918 0.51777881 2.47086763

O 3.04688573 -1.45262992 1.60328698

O 4.61744785 -0.51906794 2.83477187

N 2.09414744 0.49569455 -1.35122049

O 2.57643723 0.89988589 -2.39510226

O 1.98773086 -0.75962573 -1.07303846

O 1.64643896 1.30544841 -0.43933776

H 0.92283672 -5.04546070 -3.03560996

H -0.36680356 4.87672853 -0.04575050

O -3.94288039 -0.94845837 1.32914150

H -3.09277487 -0.96971518 1.82893062

H -4.36681938 -1.78349006 1.59051478

(NO_3^- in outer coordination sphere)

E= -13490.316599, E0 = -13489.865648, G= 232.5921 at T=298.150 K

Cl -5.19620228 3.27002025 -0.68644023

Cl -3.39916968 -3.60503101 -3.47056460

N -1.39831507 1.06811178 0.39415771

N	-0.39842486	4.02161884	2.15719914
N	-0.76671082	-1.33173156	-0.58371449
N	1.71374857	-3.83283520	-1.17164803
O	0.07184003	1.83962619	2.46969271
O	1.29094458	-2.49607706	0.60528541
C	-1.65222681	2.27525306	0.89596587
C	-2.82886219	2.98072529	0.57914704
H	-3.06263852	3.93899369	1.03936720
C	-3.72760367	2.41235399	-0.30818743
C	-3.46656585	1.14967787	-0.89609653
C	-4.31929588	0.48335171	-1.82772517
H	-5.24128819	0.97814959	-2.13648009
C	-3.99549437	-0.74953735	-2.32434201
H	-4.65620947	-1.24805820	-3.03540659
C	-2.79410958	-1.41519260	-1.93272889
C	-2.38795686	-2.69773769	-2.38229227
C	-1.18581903	-3.24129152	-1.96886551
H	-0.88417053	-4.22459316	-2.32478356
C	-0.38192835	-2.50768709	-1.07617259
C	-1.91767478	-0.78377533	-1.01130509
C	-2.25872469	0.52110541	-0.48223543
C	-0.60270882	2.71551204	1.89257514
C	0.94376642	-2.95914507	-0.50650823
C	-0.84074432	5.20217037	1.38655436
H	-1.79596901	5.59097052	1.78528690
C	0.28507963	6.21627235	1.62956274
H	1.11727750	6.02053881	0.93367434
H	-0.05762599	7.25104332	1.48375571
C	0.71050847	5.90737343	3.07289886
H	-0.01208261	6.34040356	3.78431320
H	1.70940995	6.29398489	3.32058311

C	0.66435730	4.37694025	3.12964034
H	1.61296022	3.91424346	2.80712938
H	0.41841140	3.96984172	4.12242079
C	3.03179836	-4.17437458	-0.58233720
H	2.89252114	-4.74814606	0.34715077
H	3.55247045	-3.23588920	-0.32571486
C	3.74458766	-4.94004869	-1.70035183
C	3.16525698	-4.30310965	-2.97133350
C	1.68148124	-4.12617397	-2.62829351
H	3.30479431	-4.91692781	-3.87328863
H	3.59836030	-3.30413342	-3.13892674
H	3.49295163	-6.01309013	-1.65471411
H	4.83718872	-4.83955431	-1.62382782
H	1.23545790	-3.28154516	-3.17957354
Sm	0.39437273	-0.46508390	1.63064134
N	2.59376025	-0.61318070	3.48464751
O	3.51874542	-0.67424941	4.26753616
O	2.72271657	-0.12204419	2.28743339
O	1.40100169	-1.02754629	3.77272749
O	1.59182799	0.80374157	-0.07000085
H	1.35899806	0.72252244	-1.08882117
H	2.55351973	0.64735174	-0.00514300
N	-1.89977348	-1.67499602	2.95814466
O	-1.04007232	-2.37093663	2.28369308
O	-1.68646669	-0.39280641	2.95726824
O	-2.83952522	-2.17861605	3.54155588
H	1.12455750	-5.06317234	-2.81134272
H	-0.97099668	4.95023918	0.32423425
N	1.34539294	-0.59186047	-2.90276575
O	0.73273206	-1.22632539	-3.79287291
O	0.84656221	0.53411585	-2.48818970

O 2.40599656 -1.01974976 -2.38124871

L4*Sm$(NO_3)_2^+$

E= -13133.355047, E0 = -13132.942414, G= 212.7990 at T=298.150 K

Cl -4.53173161 3.82072735 -1.06843984

Cl -3.33768463 -3.13381648 -3.91324568

N -1.07170045 1.20604539 0.19160460

N 0.07226521 3.96590137 2.19086051

N -0.65084833 -1.24810410 -0.81146318

N 1.46092284 -4.14163113 -1.11893678

O 0.40346375 1.74142194 2.27932262

O 1.15038013 -2.60946298 0.49979737

C -1.23197079 2.42852426 0.70937639

C -2.30369711 3.25835872 0.33667919

H -2.46937108 4.22637367 0.80389029

C -3.19867682 2.80919814 -0.62309468

C -3.02995539 1.53493464 -1.22294116

C -3.89019799 0.98977721 -2.22421670

H -4.72967243 1.59210634 -2.57480359

C -3.67569256 -0.25981107 -2.73530984

H -4.34219360 -0.66527832 -3.49806786

C -2.58404899 -1.06342304 -2.28552437

C -2.30861044 -2.37713027 -2.74406028

C -1.21674979 -3.07829118 -2.25403738

H -1.04221177 -4.09493637 -2.59867573

C -0.39219800 -2.46888518 -1.29231775

C -1.69918454 -0.55546421 -1.29408550

C -1.92660820 0.77012360 -0.75214946

C -0.20471129 2.72529745 1.77707553

C 0.79444164 -3.10323715 -0.60456002

C -0.32930359 5.26834440 1.60741448

H -1.30213606 5.58479118 2.02428961

C	0.78197092	6.21650362	2.07576704
H	1.64745784	6.14171076	1.39751577
H	0.44167879	7.26159525	2.09210205
C	1.13556969	5.66566610	3.46440983
H	0.37944183	5.97394562	4.20459223
H	2.12008381	5.99888420	3.82125401
C	1.09298444	4.14927721	3.25971317
H	2.05560160	3.74596357	2.90121293
H	0.80164450	3.57719731	4.15324068
C	2.60826015	-4.70157957	-0.35198119
H	2.27132487	-5.00629187	0.65016985
H	3.36762643	-3.91096854	-0.22524068
C	3.10521698	-5.85214424	-1.23099780
C	2.76539516	-5.37944508	-2.65153813
C	1.38345122	-4.73748732	-2.47430873
H	2.74170661	-6.19284153	-3.39052868
H	3.48915100	-4.62062407	-2.99071074
H	2.55942035	-6.77955914	-0.99313247
H	4.17826223	-6.03919601	-1.08535492
H	1.16691875	-3.96587801	-3.22693634
Sm	0.61205018	-0.42715171	1.30317318
N	-1.29821670	-1.50754631	3.14818144
O	-0.01623247	-1.38351095	3.38208842
O	-1.66839647	-1.09187114	1.97490227
O	-2.06244040	-1.96917462	3.95815015
N	2.97460151	0.52396387	-0.02060761
O	1.81611335	0.56717247	-0.60593951
O	2.95171404	0.02110916	1.18572462
O	3.99235964	0.91380399	-0.54055315
H	0.59625912	-5.51202154	-2.50151491
H	-0.40329701	5.19804382	0.51276672

L4*Eu(NO3)3(H2O)

(H2O in outer coordination sphere)

E= -13907.519099, E0 = -13907.068405, G= 230.0721 at T=298.150 K

Cl	-4.84747505	3.68764496	-0.75023586
Cl	-3.74979830	-3.30239868	-3.60663986
N	-1.23715436	1.14456260	0.23302105
N	0.12730131	3.97706914	1.91676891
N	-0.86075240	-1.30368686	-0.75269419
N	1.33845043	-4.03953886	-1.18053079
O	0.30591160	1.75901484	2.29258227
O	0.73070353	-2.89871597	0.68462116
C	-1.36876428	2.37031746	0.73677206
C	-2.48102522	3.18319249	0.45136127
H	-2.60664535	4.16104126	0.91419673
C	-3.45237947	2.69891715	-0.40954930
C	-3.32115746	1.41926253	-1.00017416
C	-4.25841522	0.83296192	-1.90395355
H	-5.14413786	1.40774512	-2.17883825
C	-4.06416035	-0.42117324	-2.41143394
H	-4.79251814	-0.85705352	-3.09712577
C	-2.91730094	-1.19490767	-2.05580807
C	-2.64685822	-2.50735617	-2.51388121
C	-1.51557755	-3.18649745	-2.08927727
H	-1.32898164	-4.20742273	-2.42305660
C	-0.64221352	-2.54083395	-1.19712162
C	-1.95969033	-0.64664561	-1.15721142
C	-2.16433072	0.68369538	-0.62044847
C	-0.25448161	2.70354104	1.70231712
C	0.54225498	-3.18712330	-0.51192623
C	-0.17475653	5.17313623	1.10449195
H	-1.03184891	5.72590256	1.53132999

C	1.10967040	6.00442457	1.22471976
H	1.85509729	5.63665724	0.50064689
H	0.92777777	7.07187510	1.03181863
C	1.57031512	5.70824480	2.66013408
H	0.97405654	6.29310083	3.38021398
H	2.63241601	5.93814325	2.82714152
C	1.28531361	4.21067905	2.81745601
H	2.12574005	3.57822514	2.48298717
H	1.03140664	3.90818906	3.84491467
C	2.56261802	-4.52500439	-0.49116036
H	2.29759574	-5.27136517	0.27365756
H	3.03298879	-3.66927576	0.02274871
C	3.42713284	-5.08122349	-1.62967682
C	2.99081469	-4.24610472	-2.84357691
C	1.47297692	-4.14339209	-2.64864278
H	3.25457191	-4.70177174	-3.80934691
H	3.42822361	-3.23583436	-2.78958082
H	3.20738769	-6.14858198	-1.80021429
H	4.49903965	-4.98290396	-1.40458417
H	1.03857100	-3.26199150	-3.14093995
Eu	0.82010388	-0.37698114	1.10089898
N	-0.80332655	-1.32360983	3.35360336
O	0.47373655	-1.21753943	3.40538335
O	-1.33638513	-0.98594868	2.20824265
O	-1.48239040	-1.70932937	4.28708029
N	3.60346627	-0.29236925	1.95187664
O	2.93785977	0.77352613	1.63455033
O	2.94201159	-1.39458239	1.87084234
O	4.77043009	-0.24953765	2.29970670
N	2.04210567	0.25154626	-1.51106811
O	2.53614473	0.52829611	-2.59239531

O 2.16685081 -0.91601276 -0.97121567

O 1.34631217 1.10557652 -0.83431691

H 0.96657526 -5.05377340 -3.01987934

H -0.40868476 4.89199591 0.06773264

O -3.98104477 -0.94690740 1.27649915

H -3.13346314 -1.00438845 1.77798223

H -4.40821457 -1.79670274 1.47820473

(NO_3^- in outer coordination sphere)

E= -13907.516763, E0 = -13907.065825, G= 231.1364 at T=298.150 K

Cl -5.23319340 3.23908448 -0.64997387

Cl -3.35647488 -3.55817938 -3.54132462

N -1.43028057 1.05322754 0.44276121

N -0.44572860 4.04500532 2.17025638

N -0.74596083 -1.31372392 -0.60661757

N 1.72012818 -3.83818722 -1.16064751

O 0.10531761 1.87796926 2.44680595

O 1.25228989 -2.53617287 0.62975401

C -1.68466008 2.26665807 0.93371636

C -2.86355162 2.96590376 0.61323684

H -3.10367775 3.92309856 1.07195175

C -3.75659871 2.39297462 -0.27694905

C -3.47924590 1.14051461 -0.87783211

C -4.31879377 0.48492169 -1.82845926

H -5.24155426 0.97836125 -2.13687921

C -3.98005080 -0.73586273 -2.34258127

H -4.63027191 -1.22871125 -3.06714225

C -2.77413797 -1.39552939 -1.95507693

C -2.35838318 -2.66606140 -2.42772579

C -1.16052306 -3.21399474 -2.00937724

H -0.85611373 -4.19395304 -2.37193632

C -0.36542356 -2.48937154 -1.10143578

C	-1.90552306	-0.77304345	-1.01885033
C	-2.27101016	0.51328689	-0.45890459
C	-0.61677688	2.73234391	1.90175438
C	0.93874842	-2.96792412	-0.50346988
C	-0.93383574	5.22032738	1.41922653
H	-1.89010489	5.58199978	1.84068072
C	0.17007145	6.26208973	1.64617205
H	0.99085498	6.09523630	0.92947143
H	-0.20350341	7.28838062	1.51740360
C	0.63748497	5.95072317	3.07557702
H	-0.08064063	6.35489559	3.80821037
H	1.63042450	6.36329746	3.30472565
C	0.63614374	4.41937256	3.11439133
H	1.58755136	3.98773146	2.75878024
H	0.43174803	3.99318290	4.10851526
C	3.01548839	-4.20603609	-0.53771847
H	2.84174919	-4.77462626	0.38902205
H	3.54913616	-3.27838778	-0.26887941
C	3.74019003	-4.98846960	-1.63657343
C	3.20100594	-4.34875870	-2.92387795
C	1.71387625	-4.14270782	-2.61399150
H	3.34889674	-4.97083092	-3.81878924
H	3.65684915	-3.35958958	-3.08940291
H	3.46872330	-6.05669832	-1.59205830
H	4.83230400	-4.90676546	-1.53530049
H	1.29207599	-3.29910302	-3.18449950
Eu	0.39192316	-0.46163613	1.64393318
N	2.75061989	-0.88659567	3.25299644
O	3.76822543	-1.10172176	3.88153243
O	2.70519328	-0.01376663	2.28648591
O	1.63861620	-1.49586701	3.48786569

O 1.57626498 0.77216142 -0.10326542

H 1.39347780 0.69166261 -1.11558509

H 2.54046965 0.66836423 0.00929093

N -1.93640125 -1.40419626 3.12165809

O -1.59438694 -1.90954304 1.97442806

O -1.20703316 -0.41573966 3.52226162

O -2.88027167 -1.82698846 3.76179433

H 1.14524257 -5.07214689 -2.80075216

H -1.07958794 4.97488832 0.35741767

N 1.43866122 -0.61377221 -2.97575760

O 0.86671537 -1.25799692 -3.88754034

O 0.92715943 0.50640881 -2.58872032

O 2.48007226 -1.04141259 -2.41418171

L4*Eu(NO$_3$)$_2^+$

E= -13550.552946, E0 = -13550.140636, G= 212.4587 at T=298.150 K

Cl -4.54089928 3.81096601 -1.05873406

Cl -3.33832788 -3.13675690 -3.91055226

N -1.08157527 1.19856584 0.20480029

N 0.06425181 3.96224022 2.19006443

N -0.65292984 -1.25666761 -0.80354452

N 1.45546389 -4.15107298 -1.12481654

O 0.35464430 1.73222756 2.33855343

O 1.12875974 -2.65111589 0.52373165

C -1.24855900 2.41536903 0.73093224

C -2.32005358 3.24510336 0.35707939

H -2.49010348 4.20986176 0.83012468

C -3.20782590 2.80000925 -0.61112159

C -3.03217220 1.52978170 -1.21826780

C -3.88785386 0.98700619 -2.22467780

H -4.72772789 1.58870006 -2.57543421

C -3.67129493 -0.26167783 -2.73683572

H	-4.33649397	-0.66717678	-3.50068879
C	-2.58188844	-1.06656599	-2.28362656
C	-2.31043243	-2.38248777	-2.73883057
C	-1.22366071	-3.08763647	-2.24324226
H	-1.05140686	-4.10590267	-2.58475757
C	-0.39967969	-2.48068118	-1.27995849
C	-1.69892561	-0.56022924	-1.28944254
C	-1.92961645	0.76494068	-0.74567842
C	-0.22677217	2.71386981	1.80676413
C	0.78508449	-3.12297535	-0.59008968
C	-0.30477634	5.25247717	1.55975413
H	-1.27347851	5.60447454	1.95725369
C	0.82281131	6.19199562	2.00600553
H	1.69288349	6.07453537	1.33975160
H	0.50586176	7.24428892	1.98299086
C	1.15026283	5.68160057	3.41645527
H	0.39232969	6.03027439	4.13662100
H	2.13739395	6.00667620	3.77363396
C	1.07822132	4.16025352	3.26186872
H	2.03640079	3.72765779	2.92624545
H	0.76810199	3.62415123	4.17121887
C	2.60129356	-4.72197723	-0.36465845
H	2.26082015	-5.05313635	0.62775898
H	3.35497952	-3.93014336	-0.21365494
C	3.11094832	-5.84739399	-1.26842129
C	2.77897954	-5.34050322	-2.67902589
C	1.39140189	-4.71235514	-2.49538112
H	2.76647377	-6.13473320	-3.43888950
H	3.50041461	-4.56861925	-2.99286509
H	2.56771398	-6.78321600	-1.05902815
H	4.18370104	-6.03333235	-1.11916482

H 1.17379594 -3.92415452 -3.23033738

Eu 0.58688891 -0.43554407 1.31864500

N -1.30327415 -1.52324784 3.17652678

O -0.00952540 -1.52516925 3.34622836

O -1.69521916 -0.98452312 2.06290841

O -2.06467462 -1.98595345 3.98986459

N 2.97102857 0.57481939 0.08429389

O 1.80750299 0.75283998 -0.46120319

O 2.94714236 -0.10661073 1.19633996

O 3.99584794 1.00182199 -0.39193940

H 0.61052448 -5.49200344 -2.54907775

H -0.37278131 5.14705467 0.46752992

L4*Gd(NO_3)$_3$(H_2O)

(H_2O in outer coordination sphere)

E= -14335.078043, E0 = -14334.626358, G= 231.0576 at T=298.150 K

Cl -4.86625290 3.68129373 -0.77335769

Cl -3.74760628 -3.31149602 -3.63813806

N -1.25097203 1.13975179 0.19497979

N 0.12274052 3.96192980 1.89133704

N -0.86806297 -1.29574323 -0.78496581

N 1.34933817 -4.02077627 -1.19418979

O 0.33028767 1.74117911 2.20707512

O 0.73656750 -2.84896970 0.64444596

C -1.37794912 2.36598587 0.69634199

C -2.49190593 3.17904401 0.41619751

H -2.61564684 4.15800667 0.87704152

C -3.46904039 2.69340777 -0.43840963

C -3.34102631 1.41316974 -1.02810812

C -4.27968550 0.82108492 -1.92703617

H -5.16917753 1.39126027 -2.19962835

C -4.08129072 -0.43285933 -2.43456149

H	-4.80993938	-0.87218624	-3.11778140
C	-2.92856407	-1.20114207	-2.08446169
C	-2.64867210	-2.50974369	-2.54687119
C	-1.50905740	-3.17934966	-2.12852216
H	-1.31401610	-4.19709444	-2.46709561
C	-0.63948506	-2.52826047	-1.23641455
C	-1.97263479	-0.64859545	-1.18690550
C	-2.18205762	0.68079382	-0.65120935
C	-0.25149772	2.69261575	1.64880407
C	0.54632622	-3.16247582	-0.54760569
C	-0.20610048	5.17890358	1.12255383
H	-1.06574786	5.70524025	1.57756364
C	1.06874835	6.02355003	1.25345385
H	1.80833960	5.69059420	0.50696850
H	0.86957628	7.09421635	1.09934449
C	1.55443060	5.68543577	2.67134500
H	0.96162313	6.23805428	3.41920710
H	2.61586952	5.92376518	2.83034134
C	1.29126775	4.17998838	2.78271651
H	2.13374782	3.56823850	2.41654801
H	1.05589652	3.83900428	3.80243182
C	2.56840158	-4.49886560	-0.48929983
H	2.29182720	-5.20880270	0.30538723
H	3.05393767	-3.63197780	-0.00882056
C	3.41592550	-5.11737823	-1.60816324
C	2.98483181	-4.32995462	-2.85558033
C	1.47014618	-4.18986988	-2.65660143
H	3.23283458	-4.83331251	-3.80162358
H	3.44261694	-3.32727599	-2.85133004
H	3.17845964	-6.18745089	-1.73078728
H	4.49080181	-5.02649355	-1.39479280

H 1.04787612 -3.32175827 -3.18215156

Gd 0.85053831 -0.39062530 1.08262777

N -0.70170289 -1.19784284 3.38777542

O 0.56694162 -1.02962828 3.43789840

O -1.23268962 -0.98382896 2.20231700

O -1.38135457 -1.52836716 4.33856583

N 3.56194878 -0.47879311 2.01743627

O 2.89139986 0.62716579 1.85704815

O 2.90108657 -1.55249000 1.72882330

O 4.71407843 -0.49260336 2.40379453

N 2.03246474 0.44939709 -1.43551838

O 2.52102470 0.78793609 -2.49819183

O 2.08783650 -0.77690935 -1.00551045

O 1.41379046 1.27375138 -0.65590465

H 0.94499457 -5.10611963 -2.98474383

H -0.44810569 4.92771721 0.07996369

O -3.88948488 -0.93566567 1.30336881

H -3.03313446 -0.99445671 1.79001892

H -4.33566236 -1.76331878 1.55055988

(NO_3^- in outer coordination sphere)

E= -14335.077658, E0 = -14334.625583, G= 232.9019 at T=298.150 K

Cl -5.16535187 3.28397584 -0.70312601

Cl -3.38708711 -3.61521840 -3.45822239

N -1.38985288 1.05986536 0.39928472

N -0.36730883 4.01834011 2.15550184

N -0.76592731 -1.33929014 -0.56697583

N 1.73221850 -3.82459354 -1.15680265

O 0.14142996 1.83918905 2.40696716

O 1.32001173 -2.45270944 0.59541160

C -1.62723088 2.27710581 0.88566226

C -2.79850769 2.98800087 0.56138396

H	-3.02449179	3.95349312	1.00945103
C	-3.70418620	2.41786933	-0.31860986
C	-3.45434523	1.14840996	-0.89595324
C	-4.30785227	0.48090100	-1.82628119
H	-5.22593451	0.97892338	-2.14144516
C	-3.98754430	-0.75564039	-2.31681895
H	-4.64733076	-1.25330687	-3.02939224
C	-2.78903747	-1.42432249	-1.92073476
C	-2.37886834	-2.70562530	-2.37033248
C	-1.17296696	-3.24364090	-1.95950520
H	-0.86575413	-4.22306013	-2.32065725
C	-0.37153307	-2.50983953	-1.06495070
C	-1.91644382	-0.79297978	-0.99741429
C	-2.25326800	0.51357108	-0.47464925
C	-0.56046653	2.71613932	1.86554229
C	0.96361369	-2.94255829	-0.50236052
C	-0.84623706	5.21393061	1.43157685
H	-1.80343306	5.56816435	1.85693252
C	0.26116431	6.24438715	1.69147301
H	1.08440113	6.09310484	0.97421640
H	-0.10740594	7.27542925	1.58887672
C	0.72058862	5.89417982	3.11461782
H	0.00145286	6.28356171	3.85418558
H	1.71471190	6.29527998	3.35827756
C	0.71058512	4.36243486	3.11574793
H	1.66127908	3.93392825	2.75451207
H	0.49718335	3.91274691	4.09746790
C	3.05951762	-4.14162159	-0.57394397
H	2.93459415	-4.69695568	0.36860284
H	3.57318091	-3.19296098	-0.34222901
C	3.76819205	-4.92346096	-1.68308473

C 3.17199039 -4.31620693 -2.96059895

C 1.68978000 -4.14462137 -2.60790777

H 3.30867696 -4.94588232 -3.85197139

H 3.59392476 -3.31666422 -3.15135241

H 3.52570009 -5.99735641 -1.61399615

H 4.86057091 -4.81275129 -1.61863029

H 1.23397338 -3.31248498 -3.17073655

Gd 0.38026372 -0.45255741 1.57995200

N 2.48237419 -0.61726761 3.48375273

O 3.38630176 -0.67901981 4.28833771

O 2.64910603 -0.14661609 2.27734184

O 1.27350509 -1.00642657 3.74506211

O 1.54367590 0.75464427 -0.12204624

H 1.28952658 0.67206419 -1.14877212

H 2.50287795 0.57646865 -0.07650857

N -1.88286448 -1.62156427 2.91529942

O -1.05662584 -2.31983662 2.19868994

O -1.64344406 -0.34260133 2.92422271

O -2.80956054 -2.11728358 3.52264071

H 1.13836598 -5.08896303 -2.76861596

H -0.98582339 4.99557114 0.36301377

N 1.29456401 -0.63632452 -2.93052411

O 0.69102943 -1.28372920 -3.81638575

O 0.76701963 0.47941232 -2.50768781

O 2.36933327 -1.03515017 -2.41478133

L4*Gd(NO_3)$_2^+$

E= -13978.112398, E0 = -13977.699292, G= 212.9570 at T=298.150 K

Cl -4.56790638 3.83238745 -1.10777736

Cl -3.37647390 -3.12951422 -3.94821429

N -1.10102069 1.22069502 0.13418517

N 0.04267312 3.96548510 2.15325737

N	-0.68176556	-1.22816896	-0.86509079
N	1.42775059	-4.12475300	-1.14780283
O	0.37475610	1.74003029	2.21943927
O	1.11547840	-2.57673764	0.45632502
C	-1.26402462	2.43946242	0.66138482
C	-2.33829594	3.26746154	0.29341888
H	-2.50476718	4.23295259	0.76583624
C	-3.23384643	2.82185602	-0.66833377
C	-3.06405640	1.55004990	-1.27453268
C	-3.92462158	1.00402820	-2.27540660
H	-4.76522636	1.60527086	-2.62520337
C	-3.71060443	-0.24638541	-2.78558230
H	-4.37878275	-0.65270948	-3.54642630
C	-2.61900544	-1.05030119	-2.33546400
C	-2.34564281	-2.36798000	-2.78574634
C	-1.25410295	-3.06769872	-2.29127598
H	-1.08097029	-4.08712101	-2.62865210
C	-0.42631474	-2.45434976	-1.33542442
C	-1.73235273	-0.53857940	-1.34914470
C	-1.95919061	0.78656489	-0.80845886
C	-0.23286013	2.72850442	1.72954321
C	0.76240683	-3.08210182	-0.64196551
C	-0.36389601	5.27285862	1.58428359
H	-1.33772802	5.58076429	2.00529504
C	0.74410903	6.21984816	2.06318331
H	1.60879362	6.15761995	1.38270462
H	0.39910010	7.26306295	2.09346962
C	1.10281265	5.65304661	3.44421029
H	0.34670126	5.94820118	4.18970203
H	2.08647490	5.98625088	3.80329204
C	1.06745434	4.13948727	3.22022486

H 2.03011513 3.74557710 2.85183072

H 0.78348261 3.55174589 4.10571194

C 2.57693338 -4.67641926 -0.37704217

H 2.24231386 -4.96798706 0.62960464

H 3.33670092 -3.88452005 -0.26177922

C 3.06999111 -5.83744478 -1.24398303

C 2.72865176 -5.38017130 -2.66922402

C 1.34790432 -4.73384905 -2.49720001

H 2.70237017 -6.20186853 -3.39886737

H 3.45311642 -4.62646914 -3.01807475

H 2.52296090 -6.76116514 -0.99480230

H 4.14292383 -6.02463436 -1.09792328

H 1.13200259 -3.96945930 -3.25736380

Gd 0.56676060 -0.39847997 1.21419907

N -0.97977823 -1.50587249 3.28256869

O 0.30854276 -1.32123148 3.37220192

O -1.49986935 -1.12856424 2.13595724

O -1.64036965 -1.98022830 4.16967773

N 3.10929871 0.41002169 0.31221536

O 2.02592206 0.52690363 -0.42212453

O 2.90767813 -0.11056521 1.48885620

O 4.19602203 0.75774264 -0.07704235

H 0.55925512 -5.50721931 -2.51554322

H -0.43798462 5.21467066 0.48889333

L4*Tb$(NO_3)_3(H_2O)$

(H_2O in outer coordination sphere)

E= -14773.021627, E0 = -14772.570072, G= 231.3208 at T=298.150 K

Cl -4.85855055 3.68776083 -0.78173250

Cl -3.69813824 -3.28768873 -3.68006110

N -1.22728789 1.16343069 0.16734809

N 0.10304678 3.97558451 1.93253398

N	-0.83103406	-1.26635599	-0.81244075
N	1.34440565	-4.05377483	-1.16857648
O	0.39949176	1.75219691	2.12017035
O	0.79434001	-2.76632714	0.60903811
C	-1.36067545	2.38591886	0.67905176
C	-2.48305297	3.19129467	0.40608719
H	-2.61822724	4.16371250	0.87651348
C	-3.45477295	2.70639753	-0.45378193
C	-3.31895351	1.43181551	-1.05525064
C	-4.24998856	0.84128815	-1.95918286
H	-5.14179611	1.40740490	-2.23238301
C	-4.04125881	-0.41092053	-2.47259593
H	-4.76512909	-0.85089475	-3.16044450
C	-2.88841200	-1.17533660	-2.12164378
C	-2.60402203	-2.48475790	-2.58415079
C	-1.46713388	-3.15317416	-2.15789723
H	-1.27696025	-4.17347908	-2.49030972
C	-0.60004234	-2.50386333	-1.26341522
C	-1.93640745	-0.62228531	-1.21981943
C	-2.15481377	0.70356017	-0.68126100
C	-0.23050150	2.70877743	1.62311959
C	0.57553440	-3.13345385	-0.56588602
C	-0.28111047	5.22315454	1.24261796
H	-1.16342509	5.68015671	1.72807729
C	0.95398974	6.11545324	1.42834294
H	1.70400274	5.87100506	0.65842944
H	0.70379746	7.18357229	1.34912574
C	1.46405268	5.70428705	2.81782818
H	0.85059589	6.17526960	3.60389948
H	2.51410055	5.98090601	2.98979974
C	1.27154231	4.18439245	2.82557726

H	2.13722348	3.64009166	2.41037416
H	1.06491339	3.76245022	3.82092190
C	2.54341650	-4.54538441	-0.44060209
H	2.23865461	-5.17519426	0.40931311
H	3.08526182	-3.67479110	-0.03245790
C	3.33769870	-5.29477978	-1.51708794
C	2.94501185	-4.57040977	-2.81430054
C	1.44425058	-4.31879330	-2.61785841
H	3.15026188	-5.15381241	-3.72389627
H	3.46841192	-3.60292268	-2.88466930
H	3.02887321	-6.35261965	-1.56236184
H	4.41838074	-5.26078033	-1.31708157
H	1.07509661	-3.45813990	-3.19367886
Tb	0.87737256	-0.34222609	0.97534132
N	-0.58389175	-1.15079010	3.31517124
O	0.68491447	-0.96409702	3.31689644
O	-1.15656555	-0.94410133	2.15136433
O	-1.22079861	-1.49224663	4.29087305
N	3.60295272	-0.38230103	1.84554589
O	2.92127562	0.71302849	1.67739964
O	2.94190836	-1.46366739	1.58804834
O	4.76189184	-0.38375783	2.21044421
N	2.00719619	0.48230034	-1.54862535
O	2.48101640	0.82635862	-2.61469960
O	2.09176493	-0.73701721	-1.10754228
O	1.37338722	1.29632807	-0.76788753
H	0.85879201	-5.21688843	-2.88947320
H	-0.51204073	5.02854347	0.18544316
O	-3.84539199	-0.90471649	1.31615174
H	-2.97814393	-0.96498644	1.78096759
H	-4.27030182	-1.74970484	1.54123902

(NO_3^- in outer coordination sphere)

E= -14773.021904, E0 = -14772.570032, G= 232.9343 at T=298.150 K

Cl -5.16410971 3.29988408 -0.70445418

Cl -3.37726426 -3.59304762 -3.49165273

N -1.38438606 1.07031560 0.38003451

N -0.37050369 4.01538324 2.17642665

N -0.77141464 -1.32668519 -0.58039773

N 1.72337174 -3.82933831 -1.13196647

O 0.19120221 1.84203660 2.33438087

O 1.32140851 -2.39912176 0.57340086

C -1.61942148 2.28975058 0.87200212

C -2.79389787 2.99841642 0.55366051

H -3.02091050 3.96132541 1.00620270

C -3.70114374 2.43320155 -0.32712430

C -3.45286918 1.16449535 -0.91165668

C -4.30442953 0.50050992 -1.84360874

H -5.22240448 0.99958873 -2.15750527

C -3.98435068 -0.73634821 -2.33971286

H -4.64436722 -1.23063612 -3.05431724

C -2.78893089 -1.40696597 -1.94371355

C -2.37513232 -2.68800139 -2.39443612

C -1.17187643 -3.22791219 -1.97856390

H -0.86458945 -4.20640373 -2.34176803

C -0.37401414 -2.49887490 -1.07675970

C -1.91900063 -0.77879339 -1.01510859

C -2.25240183 0.52780968 -0.49168941

C -0.54512566 2.72180390 1.83940959

C 0.95790780 -2.92314434 -0.50865453

C -0.88373989 5.22883320 1.50821805

H -1.84492135 5.54181147 1.95628071

C 0.20164061 6.27390146 1.80104089

H	1.01888299	6.17575407	1.06788194
H	-0.19355257	7.29887486	1.74936712
C	0.68841964	5.87242842	3.20123577
H	-0.02970703	6.21054411	3.96651578
H	1.67580128	6.28668308	3.45023465
C	0.71515125	4.34253311	3.13365364
H	1.67000973	3.95450187	2.73912334
H	0.52846831	3.84441805	4.09728622
C	3.04757214	-4.13466311	-0.53533399
H	2.91642070	-4.65366411	0.42683038
H	3.56828094	-3.18204808	-0.33845982
C	3.75162029	-4.96196890	-1.61373413
C	3.16519141	-4.39360428	-2.91334963
C	1.68352079	-4.19564772	-2.57211161
H	3.29832578	-5.05468559	-3.78219986
H	3.59754634	-3.40534258	-3.13680696
H	3.49844766	-6.03039169	-1.50788498
H	4.84478760	-4.85924721	-1.54965258
H	1.23979843	-3.37740541	-3.16415668
Tb	0.37826040	-0.41626182	1.50779951
N	2.50915527	-0.55764997	3.35737514
O	3.42270374	-0.61595106	4.15045023
O	2.65297794	-0.09112274	2.14990830
O	1.30312157	-0.94967121	3.63604379
O	1.51913643	0.73742735	-0.21461193
H	1.26327538	0.63076985	-1.24321771
H	2.47675014	0.54882991	-0.17165783
N	-1.81593907	-1.57480085	2.93107557
O	-1.01154411	-2.27282810	2.18889332
O	-1.58448482	-0.29536253	2.91585040
O	-2.71274209	-2.07147861	3.57942414

H 1.12233984 -5.13856459 -2.70460796

H -1.02820933 5.05187178 0.43262169

N 1.32881737 -0.70448589 -2.99832702

O 0.75904864 -1.36585677 -3.89492702

O 0.77248472 0.40682524 -2.59727812

O 2.39553690 -1.08255303 -2.45122004

L4*Tb(NO$_3$)$_2^+$

E= -14416.057306, E0 = -14415.644256, G= 213.2188 at T=298.150 K

Cl -4.56534338 3.83420801 -1.09588480

Cl -3.36896515 -3.13460755 -3.94656801

N -1.09624827 1.21595263 0.13152774

N 0.05864719 3.94757438 2.16206503

N -0.67984384 -1.22265685 -0.86244220

N 1.43912458 -4.11356449 -1.12781358

O 0.39471871 1.72207713 2.19562101

O 1.12692785 -2.53908062 0.45061862

C -1.25404572 2.43738055 0.65833086

C -2.32814384 3.26599908 0.29401335

H -2.49027586 4.23284912 0.76509494

C -3.23160768 2.82032919 -0.66212326

C -3.06881523 1.54703069 -1.26620436

C -3.93389225 0.99750507 -2.26174688

H -4.77768373 1.59656262 -2.60770202

C -3.71908832 -0.25274870 -2.77314734

H -4.38989973 -0.66049498 -3.53097749

C -2.62239027 -1.05406463 -2.32969379

C -2.34128523 -2.36840296 -2.78387237

C -1.24270594 -3.06219697 -2.29315209

H -1.06398892 -4.07939720 -2.63422894

C -0.41731334 -2.44745207 -1.33721292

C -1.73636365 -0.53844613 -1.34592819

C	-1.96266687	0.78292620	-0.80644852
C	-0.21949191	2.71726704	1.72197318
C	0.77149820	-3.06403732	-0.64031190
C	-0.35368487	5.26185369	1.61441338
H	-1.32454860	5.56276369	2.04718685
C	0.75862432	6.20231915	2.09619093
H	1.61700022	6.14678621	1.40714276
H	0.41489810	7.24536514	2.14204049
C	1.12853372	5.61824989	3.46700120
H	0.37917167	5.90529871	4.22241545
H	2.11553526	5.94614315	3.82177854
C	1.08952188	4.10736227	3.22535801
H	2.04940224	3.71602798	2.84706497
H	0.80912125	3.51125598	4.10647297
C	2.58919048	-4.65112305	-0.34866390
H	2.25569701	-4.92613840	0.66299474
H	3.34845018	-3.85682750	-0.24732724
C	3.08189249	-5.82659626	-1.19623375
C	2.73725939	-5.39508390	-2.62874699
C	1.35678983	-4.74584627	-2.46594644
H	2.70956373	-6.22975206	-3.34344411
H	3.46087503	-4.64771795	-2.99264812
H	2.53650689	-6.74644566	-0.92977440
H	4.15531683	-6.00999117	-1.04896474
H	1.13989854	-3.99445748	-3.23873425
Tb	0.54663843	-0.38073561	1.16576803
N	-1.02241230	-1.49046504	3.19285011
O	0.26641759	-1.30448902	3.29412365
O	-1.52897894	-1.10876036	2.04299498
O	-1.68964827	-1.96869266	4.07186604
N	3.03966498	0.46001789	0.20663667

O 1.94013727 0.57050389 -0.50118506

O 2.86642838 -0.06972171 1.38544559

O 4.11501789 0.81882048 -0.20148335

H 0.56737965 -5.51863337 -2.46931815

H -0.43651685 5.21819830 0.51893878

L4*Dy(NO_3)$_3$(H_2O)

(H_2O in outer coordination sphere)

E= -15221.630413, E0 = -15221.178712, G= 231.2105 at T=298.150 K

Cl -4.85850096 3.68810773 -0.78474092

Cl -3.73904657 -3.30698276 -3.65299821

N -1.24364650 1.14243424 0.17879541

N 0.12104460 3.95766139 1.89980984

N -0.86658877 -1.29026043 -0.79179788

N 1.34148288 -4.02865696 -1.17803264

O 0.34790999 1.73300922 2.17234564

O 0.73113406 -2.82829547 0.64348781

C -1.37009907 2.36829901 0.68197137

C -2.48439169 3.18148255 0.40398586

H -2.60843349 4.15921211 0.86713892

C -3.46208024 2.69847035 -0.45165247

C -3.33487129 1.41934073 -1.04321325

C -4.27250481 0.82818848 -1.94397080

H -5.16125965 1.39903092 -2.21771383

C -4.07379436 -0.42554155 -2.45226240

H -4.80139589 -0.86369550 -3.13736629

C -2.92271137 -1.19533312 -2.09980249

C -2.64281011 -2.50440311 -2.55969119

C -1.50506318 -3.17444992 -2.13582325

H -1.31167459 -4.19329262 -2.47162557

C -0.63809597 -2.52384353 -1.24168813

C -1.96876633 -0.64325047 -1.19996917

C	-2.17666340	0.68596524	-0.66644663
C	-0.24266550	2.68936634	1.63408995
C	0.54277951	-3.15621519	-0.54468650
C	-0.21936230	5.18616295	1.15501726
H	-1.08540213	5.69445276	1.61846614
C	1.04609334	6.04182053	1.30464625
H	1.78935909	5.73377371	0.55121404
H	0.83499056	7.11331558	1.17378485
C	1.53565848	5.67872524	2.71491146
H	0.93707359	6.20882750	3.47436452
H	2.59448504	5.92504501	2.87903094
C	1.28825533	4.16854000	2.79419255
H	2.13659430	3.57330298	2.41470313
H	1.05762112	3.80379033	3.80675387
C	2.55398035	-4.50800991	-0.46300158
H	2.26871037	-5.20482111	0.34019551
H	3.04597545	-3.63930082	0.00740356
C	3.39882421	-5.14969969	-1.57081723
C	2.97875309	-4.37570095	-2.83039355
C	1.46500659	-4.21761847	-2.63769555
H	3.22405195	-4.89500809	-3.76849508
H	3.44708967	-3.37796450	-2.83944893
H	3.15153122	-6.21903276	-1.67951667
H	4.47390842	-5.06611443	-1.35562062
H	1.05328298	-3.35206938	-3.17567325
Dy	0.84448487	-0.37952891	1.03751338
N	-0.63478893	-1.17508137	3.36197424
O	0.63135463	-1.00417113	3.39251256
O	-1.18151510	-0.95909983	2.18163800
O	-1.30363321	-1.50809658	4.31937885
N	3.53571320	-0.45987719	1.95443058

O 2.85634971 0.64136392 1.81013691

O 2.87927175 -1.53187060 1.65112388

O 4.68795300 -0.47253272 2.33985853

N 2.00614810 0.46821347 -1.45629454

O 2.49321270 0.80994946 -2.51837230

O 2.04986000 -0.76256490 -1.03550065

O 1.40238118 1.28991914 -0.66427076

H 0.93179727 -5.13263750 -2.95626116

H -0.45654088 4.95352602 0.10699231

O -3.84942269 -0.94513059 1.30800736

H -2.98795819 -0.99136758 1.78648329

H -4.28043556 -1.77977753 1.55848420

(NO_3^- in outer coordination sphere)

E= -15221.631164, E0 = -15221.179022, G= 233.2607 at T=298.150 K

Cl -5.15198374 3.29977083 -0.71237957

Cl -3.37211657 -3.60182929 -3.47458053

N -1.38124907 1.06791461 0.38896042

N -0.36117446 4.01461267 2.17714381

N -0.76363337 -1.33051050 -0.56902897

N 1.73159075 -3.83129072 -1.12811315

O 0.18877798 1.83836079 2.34880400

O 1.32993448 -2.40869260 0.58523667

C -1.61343718 2.28725386 0.87487775

C -2.78391242 2.99951744 0.55018872

H -3.00974131 3.96489167 0.99803275

C -3.69093585 2.43158698 -0.33029896

C -3.44441366 1.16168690 -0.90828705

C -4.29703856 0.49551985 -1.84057319

H -5.21322584 0.99539781 -2.15827966

C -3.97730112 -0.74159217 -2.33080816

H -4.63595390 -1.23752189 -3.04564095

C	-2.78138232	-1.41308796	-1.93156695
C	-2.36919641	-2.69369578	-2.38051605
C	-1.16467786	-3.23257065	-1.96491563
H	-0.85682118	-4.21112394	-2.32742691
C	-0.36677858	-2.50187016	-1.06498730
C	-1.91180885	-0.78309077	-1.00501478
C	-2.24628067	0.52395070	-0.48514390
C	-0.53876156	2.71894765	1.84759545
C	0.96758860	-2.92732668	-0.49728829
C	-0.87167084	5.22662497	1.50480390
H	-1.83423817	5.54141092	1.94883955
C	0.21343127	6.27178860	1.79882503
H	1.03267419	6.17177773	1.06817043
H	-0.18103121	7.29688883	1.74411702
C	0.69621104	5.87267017	3.20109773
H	-0.02394242	6.21252871	3.96369934
H	1.68307245	6.28695154	3.45207214
C	0.72213125	4.34263229	3.13650799
H	1.67776942	3.95307302	2.74521255
H	0.53281242	3.84643197	4.10058260
C	3.05719280	-4.13773966	-0.53613591
H	2.92939901	-4.66305017	0.42310154
H	3.57670379	-3.18553233	-0.33365101
C	3.76077771	-4.95729876	-1.62089694
C	3.16989017	-4.38321257	-2.91599131
C	1.68863475	-4.19063711	-2.56973267
H	3.30252171	-5.03905106	-3.78890681
H	3.59926319	-3.39269471	-3.13534403
H	3.50984550	-6.02678490	-1.52055073
H	4.85391521	-4.85307741	-1.55873775
H	1.24103749	-3.37173343	-3.15795016

Dy 0.37794918 -0.41946656 1.51331663

N 2.48297453 -0.55865335 3.36539268

O 3.39813375 -0.61028248 4.15692806

O 2.62662196 -0.09180484 2.15469813

O 1.28036034 -0.95568860 3.64338112

O 1.48991871 0.74733961 -0.22191057

H 1.23693538 0.63864219 -1.24579775

H 2.45064998 0.57722253 -0.17614856

N -1.82739556 -1.57380211 2.90017176

O -1.01376772 -2.27238393 2.17056131

O -1.59324527 -0.29419258 2.88752556

O -2.73566723 -2.06792498 3.53559017

H 1.13013601 -5.13486147 -2.70566916

H -1.01245165 5.04844093 0.42884621

N 1.28612697 -0.70223516 -3.00589705

O 0.71063417 -1.37198460 -3.89339781

O 0.73133236 0.40969098 -2.60754085

O 2.35828638 -1.07523131 -2.46517158

L4*Dy(NO_3)$_2^+$

E= -14864.668600, E0 = -14864.255327, G= 213.6019 at T=298.150 K

Cl -4.55432320 3.83858466 -1.11452985

Cl -3.37698245 -3.13842559 -3.93859076

N -1.07926726 1.22866058 0.10801244

N 0.05317262 3.95422006 2.16303182

N -0.66692054 -1.21662939 -0.88247836

N 1.43235433 -4.12257862 -1.12636459

O 0.42598715 1.73540556 2.16101503

O 1.13598490 -2.53617024 0.44247928

C -1.24191523 2.44525933 0.64020073

C -2.31871295 3.27259946 0.27782026

H -2.48477912 4.23595381 0.75396007

C	-3.21763420	2.82821321	-0.68200982
C	-3.04984522	1.55756390	-1.29065609
C	-3.91492009	1.00803292	-2.28605962
H	-4.75777054	1.60819149	-2.63225937
C	-3.70397282	-0.24459457	-2.79287839
H	-4.37656260	-0.65446883	-3.54789472
C	-2.61078548	-1.04793143	-2.34489846
C	-2.34087873	-2.36950183	-2.78539038
C	-1.24941325	-3.06811142	-2.28777480
H	-1.08030856	-4.09063911	-2.61759734
C	-0.41640404	-2.44801378	-1.34081304
C	-1.71865511	-0.53054178	-1.36705351
C	-1.94198036	0.79577905	-0.83022308
C	-0.20455015	2.72603965	1.70415759
C	0.77447426	-3.06529140	-0.64238775
C	-0.38392508	5.27150965	1.64209473
H	-1.36325014	5.54214621	2.07579613
C	0.70451486	6.22523546	2.15175486
H	1.56773424	6.20683575	1.46687937
H	0.33629361	7.25864124	2.22036576
C	1.08094680	5.61767149	3.51044512
H	0.32154244	5.86977673	4.26826286
H	2.05850029	5.95923281	3.87820339
C	1.07734489	4.11248684	3.23291683
H	2.04727888	3.75243068	2.84911609
H	0.80820876	3.48898911	4.09840631
C	2.58357906	-4.66081572	-0.34956622
H	2.25616336	-4.91483831	0.66940075
H	3.35258102	-3.87363195	-0.26798347
C	3.05600882	-5.85361338	-1.18399465
C	2.70913768	-5.43542099	-2.61993074

C 1.33747983 -4.76722956 -2.45814681

H 2.66732645 -6.27840424 -3.32411408

H 3.44003654 -4.70171976 -2.99674416

H 2.49983454 -6.76259565 -0.90288591

H 4.12772799 -6.04928255 -1.04028082

H 1.12765813 -4.02103567 -3.23787284

Dy 0.58550560 -0.37013051 1.14668024

N -0.93996376 -1.45720041 3.20913458

O 0.35897657 -1.34923983 3.25376701

O -1.47244799 -1.01528382 2.09350872

O -1.59562325 -1.91794574 4.10674334

N 3.06409287 0.44003540 0.15210603

O 1.96577728 0.47307235 -0.56584120

O 2.89001179 0.01001787 1.36905241

O 4.13620949 0.77785021 -0.28231889

H 0.53876752 -5.53031206 -2.45120931

H -0.45942065 5.25116158 0.54543829

L4*Ho(NO_3)$_3$(H_2O)

(H_2O in outer coordination sphere)

E= -15681.073845, E0 = -15680.622052, G= 231.3687 at T=298.150 K

Cl -4.85930777 3.68332815 -0.76703650

Cl -3.72827840 -3.30751681 -3.63921833

N -1.23368549 1.15009284 0.18252057

N 0.12598109 3.96494770 1.91060126

N -0.84796697 -1.27992952 -0.79291725

N 1.35005307 -4.03204775 -1.16956973

O 0.37666047 1.74011767 2.15638041

O 0.75664204 -2.81039095 0.64261514

C -1.36139429 2.37452793 0.68777299

C -2.47994947 3.18409514 0.41457587

H -2.60739923 4.16045332 0.87959641

C	-3.45795012	2.69872379	-0.43937713
C	-3.32783985	1.42119408	-1.03404009
C	-4.26581621	0.82778907	-1.93286896
H	-5.15726376	1.39566576	-2.20388818
C	-4.06455660	-0.42526790	-2.44157600
H	-4.79283953	-0.86604810	-3.12423539
C	-2.91032720	-1.19177604	-2.09224796
C	-2.62989187	-2.50115490	-2.55063367
C	-1.49070168	-3.16915703	-2.12792659
H	-1.29848754	-4.18923330	-2.46040726
C	-0.62067884	-2.51519179	-1.23896539
C	-1.95400023	-0.63629967	-1.19703245
C	-2.16597104	0.69163460	-0.66170704
C	-0.22835098	2.69625711	1.63346422
C	0.56061542	-3.14674973	-0.54154742
C	-0.23233856	5.19879723	1.18328202
H	-1.10097826	5.69166803	1.65833521
C	1.02459633	6.06658888	1.33536172
H	1.76566136	5.77719402	0.57241255
H	0.80057466	7.13730049	1.22055185
C	1.52860987	5.69033670	2.73699903
H	0.92955840	6.20336771	3.50772357
H	2.58571362	5.94653797	2.89694071
C	1.29858124	4.17659378	2.79755402
H	2.15014052	3.59628725	2.40238428
H	1.08114064	3.79536605	3.80690432
C	2.55993366	-4.51586390	-0.45351520
H	2.26994371	-5.19563818	0.36242157
H	3.06627393	-3.64652920	0.00023596
C	3.38943100	-5.18686581	-1.55548096
C	2.97493768	-4.42539120	-2.82443476

C 1.46473026 -4.24134636 -2.62708497

H 3.20697665 -4.96261311 -3.75586891

H 3.45940137 -3.43564892 -2.85125637

H 3.12478423 -6.25366402 -1.64692128

H 4.46676207 -5.11708784 -1.34694684

H 1.06396234 -3.37670088 -3.17473888

Ho 0.85178095 -0.36279488 1.02338970

N -0.66509473 -1.17850244 3.30302572

O 0.60421062 -1.00707746 3.33384132

O -1.21108365 -0.94404483 2.13097501

O -1.32738888 -1.52776754 4.25921822

N 3.54972219 -0.44238096 1.90533090

O 2.87421584 0.65762389 1.73085594

O 2.88217092 -1.51839161 1.65451741

O 4.70883465 -0.44640133 2.27086115

N 2.00460649 0.47516179 -1.46617866

O 2.50461173 0.81098461 -2.52397966

O 2.07214904 -0.74573916 -1.02096081

O 1.36080909 1.29195321 -0.70216328

H 0.91638881 -5.15214062 -2.93194032

H -0.47340289 4.97641468 0.13393241

O -3.88881350 -0.94778454 1.28257143

H -3.02305055 -0.98868215 1.75319326

H -4.30635738 -1.79118359 1.52631998

(NO_3^- in outer coordination sphere)

E= -15681.076691, E0 = -15680.623914, G= 234.0345 at T=298.150 K

Cl -5.14383554 3.30037045 -0.73008555

Cl -3.38333440 -3.63159561 -3.44814229

N -1.39339626 1.04814351 0.39811853

N -0.34324303 3.99451804 2.15662241

N -0.77848762 -1.34575009 -0.55087024

N	1.73121941	-3.82116294	-1.11857390
O	0.16000991	1.81015551	2.37494159
O	1.30864489	-2.42265344	0.61372995
C	-1.61909330	2.27057433	0.87667477
C	-2.78301167	2.98998117	0.54375994
H	-3.00119185	3.96119046	0.98320365
C	-3.69233680	2.42256808	-0.33521628
C	-3.45328593	1.14717901	-0.90418845
C	-4.30596638	0.47786134	-1.83463085
H	-5.21987772	0.97857976	-2.15752912
C	-3.98924041	-0.76311201	-2.31782699
H	-4.64840031	-1.26039970	-3.03131652
C	-2.79500318	-1.43580580	-1.91443706
C	-2.38145876	-2.71823239	-2.35774469
C	-1.17548847	-3.25319290	-1.94061494
H	-0.86496198	-4.23295021	-2.29834509
C	-0.37846503	-2.51583242	-1.04496074
C	-1.92676020	-0.80265743	-0.98957700
C	-2.25950146	0.50616980	-0.47496146
C	-0.54404324	2.69684362	1.85190034
C	0.95775509	-2.93344116	-0.47641170
C	-0.82118845	5.19994354	1.44876099
H	-1.77554619	5.55227757	1.88202941
C	0.29103097	6.22376204	1.71475422
H	1.10910821	6.07926369	0.99026525
H	-0.07513532	7.25709152	1.62771046
C	0.75884318	5.85439396	3.13027287
H	0.04633353	6.23722887	3.87954092
H	1.75607407	6.24895430	3.37187147
C	0.74327630	4.32272434	3.11247492
H	1.68966711	3.89537096	2.73880792

H	0.53533989	3.86177492	4.09011221
C	3.05857825	-4.12936211	-0.53072119
H	2.93368673	-4.72736645	0.38584533
H	3.54254103	-3.18095851	-0.24324958
C	3.80457520	-4.85301208	-1.65670455
C	3.18608713	-4.24952078	-2.92613840
C	1.70026469	-4.13774776	-2.57079053
H	3.34549665	-4.86299610	-3.82511187
H	3.56874871	-3.23218942	-3.10465240
H	3.61377072	-5.93834162	-1.61041021
H	4.89034271	-4.69229460	-1.58588183
H	1.21356606	-3.32184935	-3.13289762
Ho	0.34226653	-0.44438794	1.54024458
N	2.42230225	-0.60937864	3.40972137
O	3.32699633	-0.67588079	4.21283579
O	2.58405924	-0.13599402	2.20570087
O	1.21271050	-0.99624068	3.67005229
O	1.47391796	0.72863352	-0.15215605
H	1.21028566	0.65162104	-1.17542160
H	2.43028808	0.53270584	-0.12026408
N	-1.87290967	-1.59376371	2.89601755
O	-1.05599821	-2.29243541	2.17085171
O	-1.63378060	-0.31554550	2.89114141
O	-2.78837848	-2.08708382	3.52164793
H	1.18508959	-5.10125923	-2.73415923
H	-0.96564686	4.99426985	0.37830132
N	1.24810159	-0.64953673	-2.95709705
O	0.66559279	-1.31137311	-3.84563661
O	0.69502640	0.45581958	-2.53998828
O	2.32736945	-1.02466583	-2.43119836

L4*Ho$(NO_3)_2^+$

E= -15324.111885, E0 = -15323.698660, G= 212.8647 at T=298.150 K

Cl	-4.54983330	3.84354973	-1.11158204
Cl	-3.35619688	-3.12861609	-3.95722699
N	-1.08432412	1.22364938	0.11486546
N	0.05865136	3.93670750	2.17769146
N	-0.66658849	-1.21150064	-0.88008219
N	1.43759847	-4.11719036	-1.10927320
O	0.42890000	1.71732473	2.15554595
O	1.15343499	-2.50168848	0.43086368
C	-1.24477482	2.43948579	0.64960593
C	-2.31976652	3.26923633	0.28667921
H	-2.48594141	4.23276901	0.76273549
C	-3.21630478	2.82947206	-0.67781276
C	-3.04840159	1.56084025	-1.29068673
C	-3.90850234	1.01418197	-2.29223490
H	-4.75022793	1.61442089	-2.64122343
C	-3.69434237	-0.23668857	-2.80277967
H	-4.36375666	-0.64309496	-3.56258130
C	-2.60274005	-1.04155672	-2.35301161
C	-2.32755113	-2.36095548	-2.79646587
C	-1.23628116	-3.05829406	-2.29616308
H	-1.06312871	-4.07879972	-2.62979627
C	-0.40891853	-2.44018912	-1.34267402
C	-1.71724010	-0.52670318	-1.36861956
C	-1.94398642	0.79618108	-0.82835859
C	-0.20362251	2.71301579	1.71046793
C	0.78370774	-3.04982138	-0.64264029
C	-0.38099751	5.25789022	1.66890824
H	-1.35857844	5.52476263	2.10874963
C	0.70979673	6.20850563	2.17967343
H	1.56780839	6.19823360	1.48816538

H	0.34048268	7.24060059	2.26093817
C	1.09769678	5.58917189	3.52976251
H	0.34443101	5.83383417	4.29610729
H	2.07802773	5.92840624	3.89226007
C	1.09289753	4.08649921	3.23914242
H	2.05931687	3.73047948	2.84295344
H	0.83217376	3.45544004	4.10172272
C	2.58927369	-4.64404726	-0.32516220
H	2.26345992	-4.87898779	0.69897884
H	3.36078978	-3.85780287	-0.25979352
C	3.05523872	-5.85467434	-1.13704443
C	2.70448232	-5.46504927	-2.58002138
C	1.33629751	-4.78721046	-2.42783785
H	2.65622067	-6.32249594	-3.26608324
H	3.43713498	-4.74275684	-2.97498107
H	2.49775076	-6.75623369	-0.83544666
H	4.12695694	-6.05043364	-0.99341029
H	1.12940204	-4.05506659	-3.22156405
Ho	0.57640362	-0.35791999	1.11919844
N	-0.93637800	-1.45740640	3.16761327
O	0.35283682	-1.26123965	3.23812866
O	-1.47068465	-1.08584857	2.02971601
O	-1.57823122	-1.93629169	4.06572914
N	2.99047208	0.50692254	0.03604475
O	1.86386549	0.59223467	-0.62616634
O	2.87458134	-0.01243377	1.22765636
O	4.04460287	0.87693834	-0.41709784
H	0.53326362	-5.54543638	-2.40449595
H	-0.46065733	5.24567890	0.57241356

L4*Er(NO_3)$_3$(H_2O)

(H_2O in outer coordination sphere)

E= -16151.450460, E0 = -16150.998578, G= 231.5624 at T=298.150 K

Cl	-4.86028147	3.68116188	-0.78343624
Cl	-3.73447943	-3.31330514	-3.65819836
N	-1.23743701	1.14077687	0.16419402
N	0.11715614	3.94903779	1.90741861
N	-0.86143565	-1.28889906	-0.80376154
N	1.34489691	-4.03462267	-1.17765272
O	0.35436991	1.72289002	2.15643597
O	0.75343329	-2.79777193	0.62435639
C	-1.36553621	2.36436892	0.67247570
C	-2.48294449	3.17537069	0.39968228
H	-2.60842252	4.15118456	0.86648864
C	-3.46151042	2.69315457	-0.45534918
C	-3.33374286	1.41539395	-1.04973328
C	-4.27245140	0.82269692	-1.94852972
H	-5.16305542	1.39187217	-2.21980882
C	-4.07297230	-0.43058357	-2.45802283
H	-4.80167913	-0.86937243	-3.14154100
C	-2.91975594	-1.19863486	-2.10849071
C	-2.63753605	-2.50744152	-2.56800437
C	-1.49741280	-3.17463493	-2.14598751
H	-1.30340254	-4.19340515	-2.48143482
C	-0.63005185	-2.52152824	-1.25358617
C	-1.96497381	-0.64468497	-1.21108997
C	-2.17308617	0.68423009	-0.67749393
C	-0.23962678	2.68166327	1.62703979
C	0.55414009	-3.14630795	-0.55553359
C	-0.22608210	5.18279600	1.17311919
H	-1.09478593	5.68422318	1.63916707
C	1.03622115	6.04153013	1.33238816
H	1.78130460	5.74334383	0.57683837

H	0.82137775	7.11348915	1.21170664
C	1.52572381	5.66665030	2.73959374
H	0.92457378	6.18744516	3.50345874
H	2.58353043	5.91504812	2.90710974
C	1.28328502	4.15494156	2.80413961
H	2.13338661	3.56574869	2.41909814
H	1.05360663	3.77973986	3.81309080
C	2.55758834	-4.50903463	-0.45982021
H	2.27100825	-5.18338490	0.36184061
H	3.06144452	-3.63472438	-0.01308120
C	3.38750648	-5.18624640	-1.55777943
C	2.96569180	-4.43977547	-2.83331585
C	1.45528293	-4.25986767	-2.63293195
H	3.19755864	-4.98548222	-3.75977206
H	3.44578004	-3.44835973	-2.87196994
H	3.12851858	-6.25527859	-1.63807309
H	4.46499014	-5.10857916	-1.35286415
H	1.04878175	-3.40324020	-3.18891835
Er	0.84025681	-0.36038312	1.01139355
N	-0.60492015	-1.12778056	3.33914304
O	0.66219717	-0.95543540	3.34566140
O	-1.17071390	-0.91814619	2.16918421
O	-1.25442421	-1.45750284	4.31113482
N	3.51727486	-0.44454938	1.93484843
O	2.83956051	0.65414363	1.78233767
O	2.86227822	-1.51514030	1.63108325
O	4.66849756	-0.45630470	2.32604098
N	2.00977230	0.54338968	-1.43694961
O	2.50554705	0.90773994	-2.48727417
O	2.05011678	-0.69570482	-1.04103076
O	1.39939272	1.34696841	-0.63261330

H 0.90997112 -5.17644787 -2.92559505

H -0.46022370 4.95872736 0.12253288

O -3.84278679 -0.93466705 1.30893922

H -2.97990274 -0.96714860 1.78581595

H -4.26038933 -1.77535069 1.56188619

(NO_3^- in outer coordination sphere)

E= -16151.453010, E0 = -16151.000605, G= 233.6760 at T=298.150 K

Cl -5.14624262 3.30742264 -0.70808166

Cl -3.38755178 -3.60349178 -3.48231387

N -1.37714565 1.06156623 0.37180379

N -0.35019848 3.99230170 2.18316841

N -0.77176464 -1.33080983 -0.58726043

N 1.72787571 -3.82863522 -1.13288188

O 0.20655710 1.81629503 2.32265592

O 1.32483947 -2.38456655 0.56143713

C -1.60477209 2.27849889 0.86388612

C -2.77549124 2.99422503 0.54655957

H -2.99681687 3.95916176 0.99746668

C -3.68818521 2.43156672 -0.33157489

C -3.44855690 1.16217959 -0.91300148

C -4.30648994 0.49680156 -1.84156084

H -5.22383547 0.99800950 -2.15408063

C -3.99169469 -0.74098706 -2.33406568

H -4.65502453 -1.23531568 -3.04573035

C -2.79441810 -1.41453612 -1.94104886

C -2.38205433 -2.69481492 -2.39120674

C -1.17575431 -3.23379946 -1.97909653

H -0.86899209 -4.21190596 -2.34372950

C -0.37493780 -2.50221515 -1.08224189

C -1.92086089 -0.78488511 -1.01875031

C -2.24947691 0.52247769 -0.49693745

C	-0.52490121	2.70073700	1.83606899
C	0.96264923	-2.91926146	-0.51392001
C	-0.86427081	5.21266651	1.52885854
H	-1.82712317	5.51863194	1.97846985
C	0.21835279	6.25650311	1.83688164
H	1.03635085	6.17055368	1.10307634
H	-0.17956425	7.28103304	1.79880786
C	0.70536655	5.83746958	3.23185754
H	-0.01406691	6.16355228	4.00110817
H	1.69160259	6.25074434	3.48687387
C	0.73560292	4.30876350	3.14368701
H	1.69101489	3.92801142	2.74329591
H	0.55116475	3.79680443	4.10041332
C	3.05545211	-4.12349224	-0.53908515
H	2.92997742	-4.63060379	0.43010604
H	3.57450294	-3.16707516	-0.35668343
C	3.75576735	-4.96302938	-1.61047113
C	3.16144252	-4.41299391	-2.91442609
C	1.68126416	-4.21162844	-2.56826949
H	3.29080653	-5.08567858	-3.77490258
H	3.59154677	-3.42749500	-3.15369368
H	3.50552297	-6.03048897	-1.48925996
H	4.84900618	-4.85711098	-1.55361164
H	1.23505533	-3.40002322	-3.16769862
Er	0.37518126	-0.42045668	1.47279942
N	2.42509031	-0.40747920	3.37493944
O	3.31678367	-0.37546474	4.19551420
O	2.59451628	-0.02965080	2.13854790
O	1.22597170	-0.81339723	3.64424872
O	1.47856545	0.74018741	-0.23531437
H	1.25244904	0.62135243	-1.26405048

H 2.44494772 0.62515247 -0.15680884

N -1.78240919 -1.58260882 2.89468646

O -0.93198436 -2.28070712 2.20827055

O -1.60547256 -0.29855165 2.81421232

O -2.67220426 -2.08347845 3.55104160

H 1.11966741 -5.15616751 -2.68727636

H -1.00552857 5.05035782 0.45046037

N 1.31060767 -0.72751963 -3.02297997

O 0.72750992 -1.40186512 -3.90293264

O 0.75922555 0.38720047 -2.62829971

O 2.38555312 -1.09803581 -2.48795605

L4*Er(NO_3)$_2^+$

E= -15794.490674, E0 = -15794.077249, G= 213.9134 at T=298.150 K

Cl -4.54334450 3.83924365 -1.09693301

Cl -3.34867644 -3.13479376 -3.94249177

N -1.07641947 1.21940768 0.12551661

N 0.06854865 3.92903376 2.18958426

N -0.65929693 -1.21368766 -0.86765158

N 1.44655609 -4.11680841 -1.09366059

O 0.43463087 1.70849657 2.16872334

O 1.15750003 -2.50344372 0.44869435

C -1.23744035 2.43405223 0.66228431

C -2.31314874 3.26356149 0.30101430

H -2.47998834 4.22625113 0.77870959

C -3.20956302 2.82485175 -0.66418958

C -3.04137540 1.55701768 -1.27829158

C -3.90091324 1.01030838 -2.28042150

H -4.74224043 1.61071956 -2.63008428

C -3.68664098 -0.24068324 -2.79083347

H -4.35556078 -0.64689112 -3.55118275

C -2.59550476 -1.04594064 -2.34034705

C	-2.32047725	-2.36579251	-2.78203988
C	-1.22952056	-3.06264830	-2.28016090
H	-1.05623162	-4.08386517	-2.61150455
C	-0.40185043	-2.44304681	-1.32794940
C	-1.71000838	-0.53013432	-1.35652924
C	-1.93665469	0.79243815	-0.81679404
C	-0.19610612	2.70523143	1.72387815
C	0.79045469	-3.05139279	-0.62563699
C	-0.36878026	5.25028181	1.67877078
H	-1.34511781	5.51999617	2.11956978
C	0.72472781	6.19947529	2.18625402
H	1.58186018	6.18605804	1.49370134
H	0.35777876	7.23254776	2.26588154
C	1.11297143	5.58183908	3.53705931
H	0.36136168	5.82975054	4.30397034
H	2.09458923	5.91954041	3.89750934
C	1.10444534	4.07860661	3.24943137
H	2.06947279	3.71987081	2.85235906
H	0.84352297	3.44975567	4.11352730
C	2.59854484	-4.64404011	-0.31028968
H	2.27192068	-4.88409472	0.71239376
H	3.36785007	-3.85602903	-0.24072376
C	3.06887150	-5.85012817	-1.12678409
C	2.71820593	-5.45606089	-2.56852770
C	1.34802330	-4.78292942	-2.41446495
H	2.67308354	-6.31090450	-3.25804830
H	3.44905639	-4.73016119	-2.96020532
H	2.51390123	-6.75450993	-0.82906806
H	4.14107275	-6.04313231	-0.98302221
H	1.13875151	-4.04888630	-3.20573926
Er	0.57827908	-0.35449848	1.11205840

N -1.00731099 -1.43985438 3.10614204

O 0.28156990 -1.26179218 3.21142888

O -1.50254464 -1.05570006 1.95623755

O -1.68037522 -1.91351628 3.98439980

N 2.93502688 0.52805048 -0.05491454

O 1.78469396 0.58637655 -0.67674476

O 2.86359715 0.02252885 1.14558315

O 3.96713281 0.90779603 -0.54829228

H 0.54745495 -5.54380083 -2.39363313

H -0.45036542 5.23608065 0.58247811

L4*Tm(NO_3)$_3$(H_2O)

(H_2O in outer coordination sphere)

E= -16632.950468, E0 = -16632.498789, G= 231.3732 at T=298.150 K

Cl -4.84146404 3.70217729 -0.80293179

Cl -3.73665309 -3.31151891 -3.63208032

N -1.21708703 1.16695440 0.14742374

N 0.11507273 3.96765614 1.92843509

N -0.84776628 -1.27356732 -0.80244714

N 1.33207154 -4.04464769 -1.13960981

O 0.38765958 1.73995543 2.12091208

O 0.74280846 -2.79015398 0.65343982

C -1.34770381 2.38883018 0.65842605

C -2.46681023 3.19787145 0.38555065

H -2.59707808 4.17221594 0.85375053

C -3.44220901 2.71511292 -0.47268197

C -3.31335449 1.43702269 -1.06636596

C -4.25270891 0.84239006 -1.96307051

H -5.14222050 1.41181743 -2.23724747

C -4.05591536 -0.41449106 -2.46433115

H -4.78568745 -0.85654467 -3.14459085

C -2.90629101 -1.18452311 -2.10741949

C	-2.63432932	-2.50101852	-2.55045319
C	-1.50154924	-3.17297554	-2.11690450
H	-1.31597841	-4.19816017	-2.43737984
C	-0.62852073	-2.51568532	-1.23341751
C	-1.94748282	-0.62609738	-1.21706378
C	-2.15206385	0.70713156	-0.69314337
C	-0.22280686	2.70178127	1.61625540
C	0.54742438	-3.14531565	-0.52449507
C	-0.24583499	5.21547270	1.22713482
H	-1.12579906	5.68833017	1.70183587
C	0.99985218	6.09190559	1.41806304
H	1.75429547	5.82985258	0.65836698
H	0.76615793	7.16282558	1.32701492
C	1.48889637	5.68565035	2.81664729
H	0.87478095	6.17381525	3.59160280
H	2.54121637	5.94787359	2.99704766
C	1.27268350	4.16872597	2.83698869
H	2.13550735	3.60561991	2.44152308
H	1.04411900	3.76160622	3.83375931
C	2.54110432	-4.52012396	-0.41675076
H	2.25037527	-5.18854570	0.40823525
H	3.04891157	-3.64501286	0.02412495
C	3.37042284	-5.20616388	-1.50931096
C	2.96128178	-4.45586109	-2.78656960
C	1.45083869	-4.26622915	-2.59507442
H	3.19537568	-5.00199223	-3.71222734
H	3.44728994	-3.46722269	-2.82061744
H	3.10179734	-6.27282286	-1.59045303
H	4.44755936	-5.13833761	-1.29895651
H	1.05503464	-3.40450811	-3.15093970
Tm	0.85355222	-0.33665755	0.96106732

N -0.57870907 -1.20013714 3.25916219

O 0.69804251 -1.10086167 3.22622204

O -1.17337394 -0.85349584 2.14284801

O -1.20507550 -1.58169615 4.22827816

N 3.55604911 -0.37362564 1.82886314

O 2.83648491 0.70472711 1.71162939

O 2.93704939 -1.45771372 1.52180314

O 4.71553898 -0.34533173 2.19757032

N 1.98188889 0.38654494 -1.56349921

O 2.45141315 0.70405287 -2.64143753

O 2.06075168 -0.81785852 -1.09389675

O 1.35744989 1.22369194 -0.80016029

H 0.90076971 -5.17766094 -2.89483070

H -0.47031945 5.01755142 0.16919750

O -3.84552670 -0.93635827 1.28686082

H -2.98239589 -0.94650722 1.76405430

H -4.23881197 -1.78973925 1.53626359

(NO_3^- in outer coordination sphere)

E= -16632.953476, E0 = -16632.501303, G= 233.4576 at T=298.150 K

Cl -5.13874388 3.31361246 -0.73249751

Cl -3.39185023 -3.62100220 -3.45252657

N -1.37600064 1.06681347 0.36935732

N -0.34527993 3.99832416 2.17232466

N -0.77250469 -1.33321667 -0.56883836

N 1.72505486 -3.83150673 -1.13063467

O 0.19575065 1.81820917 2.32529902

O 1.33481932 -2.39510632 0.57305133

C -1.60448742 2.28491855 0.85632193

C -2.77289820 3.00190687 0.53264648

H -2.99389982 3.96924186 0.97880334

C -3.68352365 2.43696451 -0.34623864

C	-3.44449854	1.16359627	-0.91957021
C	-4.30153275	0.49395683	-1.84590411
H	-5.21703053	0.99463356	-2.16460276
C	-3.98783970	-0.74766523	-2.32896233
H	-4.65022612	-1.24575317	-3.03884721
C	-2.79309225	-1.42122126	-1.92804992
C	-2.38439298	-2.70590353	-2.36823988
C	-1.17826080	-3.24262428	-1.95353520
H	-0.87173021	-4.22323465	-2.31181049
C	-0.37614813	-2.50618052	-1.06188488
C	-1.92011225	-0.78759146	-1.00746012
C	-2.24770498	0.52430063	-0.49664664
C	-0.52633995	2.70555425	1.83205831
C	0.96405977	-2.92363620	-0.50015080
C	-0.85412884	5.21741819	1.51184797
H	-1.81881595	5.52644920	1.95538056
C	0.22853981	6.26063395	1.82215750
H	1.04975867	6.17170238	1.09229898
H	-0.16757643	7.28569221	1.77962768
C	0.70876849	5.84434986	3.22035551
H	-0.01373964	6.17307663	3.98559666
H	1.69430196	6.25717306	3.47883415
C	0.73734760	4.31536627	3.13579559
H	1.69402599	3.93333268	2.73960042
H	0.54914886	3.80545521	4.09284401
C	3.05557418	-4.13060713	-0.54524595
H	2.93479371	-4.65406179	0.41588011
H	3.57169962	-3.17583776	-0.34716332
C	3.75657535	-4.95110416	-1.63113058
C	3.15423846	-4.38654709	-2.92514420
C	1.67423129	-4.19935369	-2.56958866

H 3.28408122 -5.04601955 -3.79577208

H 3.57737350 -3.39491820 -3.15123105

H 3.51334929 -6.02169180 -1.52415490

H 4.84939146 -4.83942461 -1.57706797

H 1.21915472 -3.38605857 -3.15881205

Tm 0.36089528 -0.43355149 1.49249542

N 2.41627216 -0.42011908 3.38348675

O 3.30266309 -0.38961160 4.21112394

O 2.62510252 -0.19736947 2.11979747

O 1.18006504 -0.67120987 3.68113256

O 1.49704492 0.74381751 -0.19085066

H 1.26429820 0.64557612 -1.20728636

H 2.45638847 0.57452714 -0.12234237

N -1.78732002 -1.63267434 2.88038993

O -0.89142352 -2.31241822 2.23462963

O -1.66630411 -0.34690276 2.76248121

O -2.66807246 -2.15457058 3.53281307

H 1.11881781 -5.14642572 -2.69761753

H -0.99005711 5.05193996 0.43322271

N 1.29923689 -0.69179380 -3.01211095

O 0.70258629 -1.36009824 -3.88821936

O 0.76035100 0.41988236 -2.60602522

O 2.37977505 -1.07141435 -2.49549079

L4*Tm(NO_3)$_2^+$

E= -16275.992948, E0 = -16275.579604, G= 213.9323 at T=298.150 K

Cl -4.54130125 3.84079432 -1.09528112

Cl -3.34410429 -3.13462520 -3.94511771

N -1.06263566 1.22789800 0.10606498

N 0.07585930 3.92411447 2.19324136

N -0.64595324 -1.20218098 -0.88610119

N 1.45094800 -4.11526775 -1.08549786

O	0.47790384	1.71242154	2.11954784
O	1.19033396	-2.46300936	0.41843662
C	-1.22437978	2.43965387	0.64746833
C	-2.30455637	3.26719952	0.29350808
H	-2.47346067	4.22815943	0.77369845
C	-3.20310235	2.82853508	-0.67002523
C	-3.03454375	1.56264722	-1.28792274
C	-3.89693904	1.01373756	-2.28662038
H	-4.74149895	1.61179149	-2.63260174
C	-3.68229389	-0.23716511	-2.79758430
H	-4.35425711	-0.64496279	-3.55443192
C	-2.58789587	-1.04056919	-2.35122633
C	-2.31262016	-2.36135602	-2.78997254
C	-1.21989191	-3.05648518	-2.28912497
H	-1.04851568	-4.07841396	-2.61901259
C	-0.38909134	-2.43262172	-1.34176779
C	-1.69876051	-0.52186865	-1.37248123
C	-1.92565882	0.80084211	-0.83229220
C	-0.17521437	2.70752883	1.70197213
C	0.80701166	-3.03318119	-0.63946176
C	-0.39122552	5.24972439	1.72189987
H	-1.37121379	5.48627281	2.17340612
C	0.68495888	6.20733118	2.25064254
H	1.53653073	6.23456097	1.55171788
H	0.29553354	7.22890520	2.36443639
C	1.09834659	5.55836725	3.57905912
H	0.34830746	5.76633596	4.35923338
H	2.07550216	5.90675879	3.94143128
C	1.11971295	4.06469822	3.24625230
H	2.08803582	3.73953390	2.82871127
H	0.88204902	3.40481949	4.09377384

C 2.60367036 -4.63323164 -0.29731253

H 2.28380275 -4.83863878 0.73500603

H 3.38352704 -3.85345578 -0.25846529

C 3.05106497 -5.86925077 -1.08114040

C 2.69353938 -5.51295710 -2.53093553

C 1.33415210 -4.81593990 -2.38666224

H 2.63061762 -6.38714266 -3.19430280

H 3.43131304 -4.80961800 -2.94977593

H 2.48646116 -6.75668383 -0.75219440

H 4.12176561 -6.07251978 -0.94035041

H 1.13175011 -4.10064316 -3.19663906

Tm 0.61099899 -0.33743834 1.08301616

N -0.98867846 -1.42082965 3.07089472

O 0.30044955 -1.24205494 3.17370939

O -1.48427594 -1.03926384 1.92617869

O -1.65701282 -1.89486969 3.95371413

N 2.92475843 0.56555325 -0.15584609

O 1.76520002 0.61113417 -0.75041431

O 2.88382459 0.06402140 1.04799402

O 3.94524717 0.95162910 -0.66992819

H 0.52239507 -5.56372595 -2.34043813

H -0.47649065 5.26500177 0.62591404

L4*Yb(NO_3)$_3$(H_2O)

(H_2O in outer coordination sphere)

E= -17125.721365, E0 = -17125.269951, G= 231.1715 at T=298.150 K

Cl -4.84003782 3.69956136 -0.78223974

Cl -3.72434306 -3.30737281 -3.62596440

N -1.21503890 1.16624546 0.16597655

N 0.11821893 3.95658398 1.95554793

N -0.82870364 -1.26132584 -0.81021255

N 1.33860159 -4.04829788 -1.13118398

O	0.37508017	1.72591627	2.15397525
O	0.76766193	-2.77022719	0.65136766
C	-1.34906232	2.38517475	0.68347770
C	-2.46782136	3.19419909	0.41013300
H	-2.60092998	4.16587448	0.88315004
C	-3.43920732	2.71415854	-0.45435986
C	-3.30548286	1.44008374	-1.05577660
C	-4.24217510	0.84745842	-1.95663571
H	-5.13234615	1.41639698	-2.22960567
C	-4.04388666	-0.40805468	-2.46086001
H	-4.77346134	-0.84966183	-3.14163327
C	-2.89312387	-1.17738819	-2.10596752
C	-2.62073708	-2.49460196	-2.54705381
C	-1.48854291	-3.16656351	-2.11159515
H	-1.30584657	-4.19354725	-2.42791724
C	-0.61150151	-2.50641632	-1.23382747
C	-1.93322170	-0.61779922	-1.21812558
C	-2.14355230	0.71049184	-0.68476075
C	-0.22705247	2.69232464	1.64641929
C	0.56380367	-3.13519502	-0.52165902
C	-0.23749383	5.20401049	1.25041521
H	-1.11469793	5.68217564	1.72477162
C	1.01252377	6.07496452	1.43715799
H	1.76492941	5.80697632	0.67752361
H	0.78377169	7.14668274	1.34319913
C	1.50130486	5.67067003	2.83644748
H	0.89003456	6.16350698	3.61068034
H	2.55495715	5.92879772	3.01494765
C	1.27862227	4.15473413	2.86115885
H	2.13824534	3.58812451	2.46357441
H	1.05054331	3.75107741	3.85943103

C 2.54643726 -4.52405787 -0.40795457

H 2.25549078 -5.18365145 0.42404625

H 3.06065059 -3.64790106 0.02318919

C 3.36964965 -5.22339439 -1.49670374

C 2.96323442 -4.47940159 -2.77849030

C 1.45441031 -4.27870178 -2.58555102

H 3.19197989 -5.03360701 -3.70069528

H 3.45576239 -3.49432230 -2.82052779

H 3.09337258 -6.28868961 -1.56951535

H 4.44756889 -5.16159058 -1.28852606

H 1.06384301 -3.41752791 -3.14596558

Yb 0.82680589 -0.31938326 0.95315403

N -0.62857038 -1.19359052 3.23603487

O 0.64391887 -1.04278874 3.22635150

O -1.21209741 -0.91988003 2.09610486

O -1.25847805 -1.56080174 4.20946503

N 3.56058931 -0.25765529 1.74387872

O 2.86923981 0.80601221 1.47879279

O 2.91333818 -1.36038983 1.61998665

O 4.73072577 -0.20776542 2.08163095

N 1.97354460 0.33887574 -1.58931863

O 2.45983768 0.62763387 -2.66959310

O 2.08928347 -0.83898628 -1.06558692

O 1.29410052 1.18209004 -0.88539511

H 0.89794743 -5.18797779 -2.88005686

H -0.46442053 5.00357008 0.19349580

O -3.88723326 -0.92952526 1.25943720

H -3.02200699 -0.96859968 1.73166287

H -4.28895617 -1.78773129 1.47667301

(NO_3^- in outer coordination sphere)

E= -17125.725615, E0 = -17125.273655, G= 232.9416 at T=298.150 K

Cl	-5.15610266	3.31237769	-0.71434826
Cl	-3.40772104	-3.62601781	-3.42468476
N	-1.37207603	1.08436406	0.35808715
N	-0.34943479	4.00107622	2.17833686
N	-0.77741897	-1.32292902	-0.56436557
N	1.72833180	-3.81493497	-1.13606727
O	0.15851793	1.81388187	2.35754991
O	1.36283743	-2.34515262	0.54283398
C	-1.61001861	2.29547763	0.85617644
C	-2.78458953	3.00754571	0.54294938
H	-3.00858450	3.97149277	0.99553239
C	-3.69533420	2.44143009	-0.33485684
C	-3.45392609	1.16884518	-0.90965259
C	-4.31558084	0.49395034	-1.82819986
H	-5.23558760	0.99043876	-2.14047217
C	-4.00190735	-0.74774402	-2.31122804
H	-4.66838980	-1.24935603	-3.01478362
C	-2.80340719	-1.41767514	-1.91523206
C	-2.39511752	-2.70383453	-2.35086465
C	-1.18547356	-3.23742557	-1.94077897
H	-0.88184261	-4.22010469	-2.29578352
C	-0.37939140	-2.49555635	-1.05683088
C	-1.92628980	-0.77965325	-1.00102222
C	-2.25080657	0.53590524	-0.49614987
C	-0.54145515	2.70881510	1.84479773
C	0.97274369	-2.89790606	-0.50918365
C	-0.83673114	5.21887541	1.49980533
H	-1.80000043	5.54646158	1.93295848
C	0.25833476	6.24987030	1.80662155
H	1.08585393	6.13786840	1.08710444
H	-0.12127254	7.28023148	1.74568880

C	0.71698719	5.84603548	3.21591020
H	-0.00855739	6.19717550	3.96820140
H	1.70593858	6.24731541	3.47950411
C	0.72277099	4.31537867	3.15412331
H	1.67916524	3.91241097	2.77867365
H	0.51197731	3.82332015	4.11583853
C	3.06751394	-4.09939814	-0.56414104
H	2.96160054	-4.59218025	0.41467905
H	3.59011745	-3.14086270	-0.40475011
C	3.74770045	-4.95533419	-1.63556623
C	3.12928128	-4.42499781	-2.93653321
C	1.65589106	-4.21782303	-2.56387639
H	3.24116373	-5.11138105	-3.78863192
H	3.55813980	-3.44518375	-3.19995022
H	3.49937344	-6.02070189	-1.49410319
H	4.84181786	-4.84930325	-1.60127425
H	1.19686997	-3.41776443	-3.16648793
Yb	0.35695237	-0.41764045	1.50309408
N	2.38475466	-0.36739776	3.42190957
O	3.26414680	-0.29495135	4.25617790
O	2.60212731	-0.15438229	2.15647626
O	1.15944278	-0.65646982	3.71013641
O	1.51269090	0.76868850	-0.17396142
H	1.30437362	0.65989774	-1.18025792
H	2.46904516	0.59362328	-0.08035686
N	-1.77377272	-1.72764933	2.82081747
O	-0.77328730	-2.36304307	2.30339742
O	-1.76285708	-0.44535160	2.60890913
O	-2.65768147	-2.28030658	3.44560528
H	1.09197450	-5.16334820	-2.66277146
H	-0.96848637	5.04172039	0.42254928

N 1.35332453 -0.67865312 -3.04157114

O 0.75812674 -1.35026979 -3.91696501

O 0.81222075 0.42216107 -2.63070536

O 2.44099307 -1.05395055 -2.53803658

L4*Yb($(NO_3)_2^+$)

E= -16768.763641, E0 = -16768.350608, G= 213.6745 at T=298.150 K

Cl -4.53099203 3.84630418 -1.11160123

Cl -3.34964681 -3.14021778 -3.94074488

N -1.06805527 1.22383189 0.10889833

N 0.07599076 3.92548299 2.18744588

N -0.65259403 -1.21186543 -0.87860388

N 1.44653141 -4.12408495 -1.08973563

O 0.46410921 1.70921850 2.13647056

O 1.18072212 -2.48577666 0.42976043

C -1.22485805 2.43736148 0.64629239

C -2.29969740 3.26870036 0.28445768

H -2.46564198 4.23266649 0.75923812

C -3.19913292 2.82908154 -0.67773819

C -3.03674150 1.55871999 -1.28836489

C -3.90033722 1.00872993 -2.28533697

H -4.74337816 1.60759234 -2.63363743

C -3.68780994 -0.24403597 -2.79249573

H -4.35999203 -0.65268719 -3.54866290

C -2.59440541 -1.04797471 -2.34435129

C -2.31864047 -2.36831307 -2.78439617

C -1.22513330 -3.06348109 -2.28512406

H -1.05379403 -4.08525610 -2.61574054

C -0.39504191 -2.44197345 -1.33577776

C -1.70589960 -0.53003216 -1.36466765

C -1.93132687 0.79498911 -0.82799137

C -0.17843667 2.70432305 1.70692205

C	0.80092990	-3.04618549	-0.63184595
C	-0.37849894	5.24902344	1.69830477
H	-1.36100113	5.49687767	2.13824415
C	0.69842792	6.20538330	2.22765183
H	1.55750358	6.21907473	1.53751993
H	0.31505141	7.23080730	2.32633209
C	1.09332764	5.56762314	3.56701541
H	0.33489183	5.78680754	4.33604908
H	2.06793976	5.91455221	3.93762660
C	1.11064696	4.07086897	3.24837804
H	2.08170938	3.73844028	2.84294057
H	0.86248046	3.41992259	4.09971952
C	2.60171556	-4.64442158	-0.30701923
H	2.28189850	-4.86737776	0.72168541
H	3.37491536	-3.85886431	-0.25454000
C	3.06029963	-5.86536026	-1.10777414
C	2.70062542	-5.49212217	-2.55306554
C	1.33485365	-4.81033850	-2.39892936
H	2.64703727	-6.35857344	-3.22735882
H	3.43267155	-4.77640581	-2.96081018
H	2.50194526	-6.76175833	-0.79251701
H	4.13228321	-6.06295347	-0.96872067
H	1.12439835	-4.08776855	-3.20037770
Yb	0.58839869	-0.34300706	1.08370006
N	-0.98180532	-1.41309786	3.09515643
O	0.31662741	-1.32159185	3.14553189
O	-1.50048232	-0.96103531	1.98806643
O	-1.64204109	-1.87512457	3.99149108
N	2.92502427	0.57006305	-0.09085657
O	1.76684880	0.69973022	-0.67363328
O	2.87320948	-0.03041228	1.06347227

O 3.95541334 0.96880156 -0.57642132

H 0.53061604 -5.56673908 -2.36095953

H -0.45365980 5.25385714 0.60142905

L4*Lu($(NO_3)_3(H_2O)$)

(H_2O in outer coordination sphere)

E= -17629.924236, E0 = -17629.471678, G= 232.5959 at T=298.150 K

Cl -4.88080740 3.68029141 -0.74388206

Cl -3.73431587 -3.30866647 -3.63720155

N -1.23456323 1.16160321 0.15570208

N 0.11664879 3.94874477 1.93816745

N -0.84074765 -1.25390816 -0.82509768

N 1.33719647 -4.03695440 -1.13914776

O 0.41917241 1.72321844 2.07504606

O 0.76879269 -2.73259020 0.62111336

C -1.36413753 2.37940216 0.67422956

C -2.49059391 3.18405175 0.41754213

H -2.62352872 4.15430927 0.89340955

C -3.47130895 2.70086980 -0.43539736

C -3.33849978 1.42941511 -1.04260898

C -4.27840662 0.83280092 -1.93761969

H -5.17545080 1.39529037 -2.20140123

C -4.07377100 -0.41908032 -2.44933128

H -4.80568790 -0.86302412 -3.12609172

C -2.91378832 -1.18132043 -2.10833311

C -2.63181996 -2.49248362 -2.56047964

C -1.48997617 -3.15624642 -2.13693404

H -1.30178988 -4.18034124 -2.45901561

C -0.61474383 -2.49372196 -1.25941396

C -1.95302415 -0.61918116 -1.22307575

C -2.16983438 0.70548254 -0.68367559

C -0.21854834 2.68805718 1.60850167

C	0.56395841	-3.11402392	-0.54871863
C	-0.28066850	5.20846033	1.27882826
H	-1.15115774	5.65771866	1.79219663
C	0.96004057	6.09575701	1.45163739
H	1.69179654	5.86276388	0.66095436
H	0.71040696	7.16547489	1.39576948
C	1.50048912	5.66062593	2.82226396
H	0.90693057	6.12095642	3.62961721
H	2.55509734	5.93161869	2.97398639
C	1.30354536	4.14116764	2.81079578
H	2.15804887	3.59909773	2.37017441
H	1.11484420	3.70515084	3.80358195
C	2.54165888	-4.50789833	-0.40473536
H	2.24244261	-5.14502668	0.44158685
H	3.06311750	-3.62686777	0.00807493
C	3.35567307	-5.24197626	-1.47743750
C	2.95047379	-4.53120375	-2.77846622
C	1.44420910	-4.31047726	-2.58638477
H	3.16992736	-5.11390972	-3.68519545
H	3.45250845	-3.55273533	-2.85128617
H	3.07256818	-6.30706263	-1.52042663
H	4.43468380	-5.18138885	-1.27488995
H	1.05909228	-3.46177745	-3.16923809
Lu	0.87831533	-0.32634500	0.93057096
N	-0.50075781	-1.11508000	3.25792289
O	0.76573032	-0.92609149	3.22929335
O	-1.09653318	-0.90827888	2.10310721
O	-1.11854482	-1.45549262	4.24590063
N	3.57874537	-0.36974770	1.70592344
O	2.87516022	0.71627146	1.53757644
O	2.92079759	-1.45874500	1.49384165

O 4.74958134 -0.34536159 2.03107047

N 1.93522942 0.45735863 -1.57586753

O 2.39815855 0.77908587 -2.65359521

O 2.03368616 -0.75208431 -1.10521460

O 1.29913616 1.27825189 -0.80747908

H 0.87791145 -5.22267866 -2.85142541

H -0.53512436 5.03298187 0.22378550

O -3.79254603 -0.93982220 1.31568849

H -2.91515112 -0.97274625 1.76551902

H -4.20994711 -1.77193844 1.59568787

(NO_3^- in outer coordination sphere)

E= -17629.931080, E0 = -17629.478036, G= 234.4627 at T=298.150 K

Cl -5.15501738 3.30927372 -0.70456171

Cl -3.36983562 -3.61042500 -3.46673083

N -1.38218987 1.07159865 0.36784342

N -0.34094384 3.99234796 2.18369150

N -0.77290672 -1.31411815 -0.57464904

N 1.74190533 -3.80320811 -1.09784186

O 0.22952224 1.81839895 2.28203344

O 1.34517908 -2.32124734 0.56367999

C -1.60666072 2.28944230 0.85837412

C -2.77998257 3.00241613 0.54402405

H -3.00124288 3.96874785 0.99191809

C -3.69556451 2.43628478 -0.33021313

C -3.45713973 1.16563177 -0.90992141

C -4.31279421 0.49352115 -1.83588803

H -5.23252916 0.98933530 -2.15003347

C -3.99268317 -0.74471843 -2.32561064

H -4.65458584 -1.24305904 -3.03587461

C -2.79281306 -1.41367531 -1.93159294

C -2.37177277 -2.69274950 -2.37704110

C	-1.16137195	-3.22148299	-1.96250415
H	-0.84767729	-4.19795227	-2.32535362
C	-0.36582488	-2.48309803	-1.06613255
C	-1.92427886	-0.77796805	-1.01041675
C	-2.25724578	0.52919036	-0.49444959
C	-0.51524693	2.70643687	1.82013595
C	0.97646618	-2.88197374	-0.49606058
C	-0.87428248	5.21938038	1.55786455
H	-1.83120084	5.51070976	2.02920461
C	0.20705219	6.26535559	1.86331391
H	1.01215613	6.19677210	1.11360967
H	-0.19807161	7.28763485	1.84875512
C	0.72223175	5.82775021	3.24236250
H	0.01586807	6.13820314	4.02993059
H	1.71088374	6.24269342	3.48498011
C	0.75919318	4.30070400	3.13080573
H	1.70929897	3.92999864	2.70908904
H	0.59258294	3.77385831	4.08267784
C	3.07191634	-4.08092546	-0.50095862
H	2.95012212	-4.57024622	0.47777346
H	3.58707237	-3.11915946	-0.33688554
C	3.77398849	-4.93749666	-1.55748987
C	3.17618036	-4.41333961	-2.87045598
C	1.69554341	-4.21158218	-2.52621245
H	3.30707073	-5.10094786	-3.71881437
H	3.60281062	-3.43089080	-3.12794447
H	3.52769446	-6.00341320	-1.41647375
H	4.86688566	-4.82650900	-1.50388682
H	1.24640787	-3.41290784	-3.14044762
Lu	0.35726923	-0.39054769	1.44319630
N	2.42547154	-0.53427958	3.27412891

O 3.33673525 -0.59243393 4.07003164

O 2.56868887 -0.05319720 2.07050943

O 1.22183144 -0.93785810 3.53750849

O 1.43603957 0.72418338 -0.26531711

H 1.18817306 0.60636812 -1.29057097

H 2.39756989 0.56007820 -0.21310665

N -1.79567564 -1.53868949 2.84255624

O -0.99063778 -2.23291564 2.10063124

O -1.56807089 -0.25791067 2.81662989

O -2.68768501 -2.03476548 3.49894619

H 1.13743377 -5.16013241 -2.62845969

H -1.03255522 5.07290554 0.47964886

N 1.28317273 -0.73805147 -3.04379964

O 0.72520339 -1.42541564 -3.92922878

O 0.70094138 0.36298949 -2.65255499

O 2.36192417 -1.08292127 -2.49837232

L4*Lu(NO$_3$)$_2^+$

E= -17272.970752, E0 = -17272.556767, G= 214.5861 at T=298.150 K

Cl -4.55833626 3.85447264 -1.13173532

Cl -3.37119889 -3.13185501 -3.96836996

N -1.07835853 1.24137175 0.06022176

N 0.05243400 3.92531061 2.17042637

N -0.66351610 -1.18562090 -0.92870164

N 1.42837465 -4.10544777 -1.10683870

O 0.46984917 1.71826923 2.06529212

O 1.18462813 -2.42744231 0.37001535

C -1.23909616 2.45237422 0.60538930

C -2.31958055 3.27937722 0.25243443

H -2.48642039 4.24077940 0.73234957

C -3.22100592 2.84155607 -0.70932400

C -3.05576849 1.57478893 -1.32705164

C	-3.92206573	1.02306545	-2.32094908
H	-4.76854944	1.61971760	-2.66462302
C	-3.70908570	-0.22921650	-2.82973289
H	-4.38444138	-0.63930649	-3.58230138
C	-2.61226869	-1.03104866	-2.38615680
C	-2.33703089	-2.35364437	-2.82042885
C	-1.24197030	-3.04607415	-2.31977510
H	-1.07127583	-4.06929588	-2.64588380
C	-0.40729848	-2.41920877	-1.37831807
C	-1.71966207	-0.50841278	-1.41387749
C	-1.94523787	0.81445044	-0.87558919
C	-0.18920606	2.71393037	1.66240883
C	0.79238564	-3.01345372	-0.67436564
C	-0.42185971	5.25507259	1.71857548
H	-1.40451956	5.47821379	2.17124796
C	0.64669931	6.21096849	2.26574898
H	1.50048029	6.25297785	1.57027459
H	0.25114012	7.22851276	2.39337039
C	1.05943084	5.54392195	3.58538818
H	0.30491757	5.73404026	4.36585379
H	2.03273535	5.89334202	3.95701671
C	1.09309900	4.05634737	3.22821665
H	2.06451535	3.74498415	2.80740809
H	0.85869837	3.37888598	4.06253338
C	2.58515620	-4.61207628	-0.31663373
H	2.27228165	-4.79290247	0.72236413
H	3.36812067	-3.83467531	-0.30001226
C	3.02020264	-5.86677551	-1.07702684
C	2.65200543	-5.54085827	-2.53138232
C	1.29802835	-4.83211327	-2.39217091
H	2.57823825	-6.42920923	-3.17449260

H 3.39026380 -4.85155678 -2.97205567

H 2.45500231 -6.74443102 -0.72382230

H 4.09128666 -6.07145023 -0.94145179

H 1.09516406 -4.13315201 -3.21624875

Lu 0.58216357 -0.31133303 1.00840914

N -0.72805321 -1.40558422 3.13461041

O 0.55478150 -1.18194187 3.12435794

O -1.33787763 -1.05341554 2.01930213

O -1.31226110 -1.88768446 4.06849289

N 3.01416135 0.49425820 0.06329885

O 1.90158713 0.58914238 -0.63776636

O 2.84866500 -0.01831878 1.24737191

O 4.08145857 0.85277063 -0.36356395

H 0.48162901 -5.57322264 -2.32435155

H -0.50363868 5.28782606 0.62262261

Nitrates

$La(NO_3)_3$

E= -9336.284233, E0 = -9336.240076, G= -1.6603 at T=298.150 K

La 0.00001769 -0.00003848 -0.00000325

N -2.90586615 0.01336963 0.05776037

O -2.20656466 0.68517798 -0.82677323

O -4.10616684 0.01895838 0.08170674

O -2.17813969 -0.66517508 0.91379160

N 1.41289687 -1.98400593 -1.58599830

O 1.08368850 -2.17620158 -0.32982165

O 1.04821169 -0.81732994 -2.06362891

O 1.99683034 -2.80388880 -2.24032688

N 1.49282134 1.97067058 1.52826393

O 1.11489129 2.16643786 0.28641924

O 2.10974574 2.78510880 2.15868187

O 1.13763368 0.80691624 2.01992869

$Ce(NO_3)_3$

E= -9703.698544, E0 = -9703.654621, G= -3.1254 at T=298.150 K

Ce	0.01325159	-0.00005793	-0.00031676
N	-2.85419488	0.01306607	0.05679771
O	-2.15112543	0.68750197	-0.82212991
O	-4.05391550	0.01851519	0.08067576
O	-2.12286687	-0.66788226	0.90718979
N	1.38688433	-1.96393526	-1.56819594
O	1.07031250	-2.14300060	-0.30613726
O	1.04337847	-0.78423816	-2.03209925
O	1.92896450	-2.80121565	-2.23577523
N	1.46596551	1.95087302	1.51144469
O	1.10103607	2.13311219	0.26296231
O	2.04162216	2.78310466	2.15686202
O	1.13068771	0.77415687	1.98872221

$Pr(NO_3)_3$

E= -10080.779371, E0 = -10080.735193, G= -2.1144 at T=298.150 K

Pr	0.01586251	0.00000078	-0.00031168
N	-2.82815266	0.01285240	0.05620209
O	-2.12219620	0.68811464	-0.82121718
O	-4.02770329	0.01835372	0.08015907
O	-2.09396267	-0.66901970	0.90489709
N	1.37120199	-1.95889008	-1.56366873
O	1.03669703	-2.14566994	-0.30869672
O	1.01051748	-0.78776538	-2.03282094
O	1.95495474	-2.77799606	-2.21763659
N	1.45025003	1.94604659	1.50757396
O	1.06751931	2.13635969	0.26701921
O	2.06728244	2.75945830	2.13766003
O	1.09772921	0.77815503	1.99084032

$Nd(NO_3)_3$

E= -10467.685921, E0 = -10467.641630, G= -2.0626 at T=298.150 K

Nd 0.00072433 0.00504316 -0.00696500

N -2.83198118 0.01291180 0.05764951

O -2.12624669 0.79934633 -0.72058380

O -4.03116798 0.01686896 0.08455694

O -2.09697580 -0.77782995 0.80304176

N 1.37683094 -1.93652308 -1.54500532

O 1.17032194 -2.05412650 -0.25478247

O 0.88404632 -0.83126140 -2.05267286

O 1.95899403 -2.75817943 -2.19672585

N 1.45471191 1.92182791 1.48990571

O 1.20102465 2.04891944 0.20865670

O 2.07016277 2.73366427 2.12308311

O 0.96955508 0.81933838 2.00984168

$Pm(NO_3)_3$

E= -10864.548907, E0 = -10864.504658, G= -0.5963 at T=298.150 K

Pm -0.02315194 -0.00152896 0.00314578

N -2.84424090 -0.02219432 0.10059166

O -2.13659453 0.65779483 -0.77898616

O -4.04364967 -0.01119621 0.11704404

O -2.11465216 -0.70167094 0.94314265

N 1.38741612 -1.88944674 -1.54561615

O 0.84865528 -2.15826154 -0.37648380

O 1.21148396 -0.65132111 -1.92846835

O 1.98172879 -2.70513415 -2.19418263

N 1.46499348 1.91251624 1.44347525

O 1.26223552 1.99964273 0.15446903

O 2.09227204 2.71961355 2.07140279

O 0.91350412 0.85118693 1.99046576

$Sm(NO_3)_3$

E= -11271.487493, E0 = -11271.443375, G= -1.7063 at T=298.150 K

Sm -0.11102058 0.00127051 0.00197510

N -2.92669868 0.02567798 0.06345502

O -2.23266435 0.23235433 -1.02981436

O -4.12645435 0.03640363 0.08975135

O -2.18924832 -0.19368589 1.12541103

N 1.43372667 -1.83199322 -1.46817291

O 0.73742867 -2.17122221 -0.41384146

O 1.34296644 -0.55980039 -1.77231610

O 2.09454870 -2.60973811 -2.09974933

N 1.52787316 1.80589199 1.40407479

O 0.79103631 2.15863061 0.38220060

O 2.22884321 2.57141423 2.00662827

O 1.42966318 0.53479666 1.71039796

$Eu(NO_3)_3$

E= -11688.686686, E0 = -11688.642767, G= -1.6288 at T=298.150 K

Eu -0.00040956 0.00005385 0.00008606

N -2.81777573 0.01283353 0.05601045

O -2.10939622 1.01220977 -0.40208748

O -4.01907682 0.01823581 0.07981354

O -2.10082650 -0.99293768 0.48593813

N 1.37018275 -1.92394626 -1.53614879

O 1.48323298 -1.83753026 -0.23606995

O 0.56286407 -1.03771532 -2.05889511

O 1.95527649 -2.74380279 -2.19129682

N 1.44773650 1.91116858 1.48007619

O 1.50827849 1.82384074 0.17656264

O 2.06590962 2.72551823 2.11127162

O 0.65400380 1.03207183 2.03473973

$Gd(NO_3)_3$

E= -12116.240312, E0 = -12116.195668, G= -1.6729 at T=298.150 K

Gd -0.00034861 0.00149084 -0.00177597

N -2.77423048 0.01277978 0.05502230

O -2.06638837 0.80802512 -0.71570069

O -3.97202015 0.01748536 0.07951467

O -2.04177594 -0.78816861 0.79631269

N 1.34883487 -1.89458323 -1.51259303

O 1.15525925 -2.01016402 -0.21777789

O 0.84183085 -0.79453140 -2.02290750

O 1.93178630 -2.71301770 -2.16501427

N 1.42537725 1.88122678 1.45822752

O 1.17897975 2.00278735 0.17277186

O 2.04149508 2.69297504 2.08813334

O 0.93120033 0.78369468 1.98578703

$Tb(NO_3)_3$

E= -12554.185252, E0 = -12554.140565, G= -1.1803 at T=298.150 K

Tb 0.00022869 0.00008624 -0.00025795

N -2.75734234 0.01270564 0.05493535

O -2.04427004 0.93380946 -0.55440629

O -3.95488858 0.01821032 0.07876861

O -2.02904773 -0.91502607 0.63542390

N 1.34073031 -1.88379765 -1.50392771

O 1.31626105 -1.89500308 -0.18963613

O 0.66524190 -0.88740408 -2.03219819

O 1.92245948 -2.70218515 -2.15703583

N 1.41651845 1.87096119 1.44912195

O 1.33888316 1.88364744 0.13688448

O 2.03146839 2.68358541 2.07856154

O 0.75375700 0.88041043 2.00376630

$Dy(NO_3)_3$

E= -13002.797004, E0 = -13002.752377, G= -1.3150 at T=298.150 K

Dy 0.01642095 -0.01882173 0.00158654

N -2.72606516 0.03294614 0.05116201

O -2.00636911 0.82482022 -0.71024448

O -3.92338967 0.04955097 0.07503923

O -2.00069284 -0.78169459 0.78757453

N 1.33424139 -1.89134765 -1.51141453

O 1.24541831 -1.95097136 -0.20029895

O 0.73277402 -0.84197813 -2.02541542

O 1.90803230 -2.71048784 -2.16981554

N 1.38598907 1.86375952 1.45873475

O 1.14482260 1.97613215 0.17088243

O 1.99149191 2.68346357 2.08799291

O 0.89732605 0.76462865 1.98421681

$Ho(NO_3)_3$

E= -13462.241767, E0 = -13462.197053, G= -0.9266 at T=298.150 K

Ho 0.00281437 -0.11804630 0.15013550

N -2.72314405 0.02620037 0.03395709

O -1.93993187 0.85188919 -0.61232531

O -3.91885209 0.07421960 -0.00667118

O -2.06241083 -0.87069666 0.74226415

N 1.32492495 -1.84441030 -1.50425935

O 1.24710476 -1.99283671 -0.19481511

O 0.69869673 -0.78140330 -1.94091988

O 1.90977955 -2.61064291 -2.21454883

N 1.39715326 1.86370134 1.41251338

O 1.20178080 1.84617698 0.11854985

O 2.00395465 2.72456503 1.98205626

O 0.85812968 0.83128357 2.03406358

$Er(NO_3)_3$

E= -13932.619529, E0 = -13932.574812, G= -1.3639 at T=298.150 K

Er -0.01323813 0.01545679 0.01511501

N -2.73721170 -0.02880375 0.06403738

O -2.02470183 0.85735059 -0.60282665

O -3.93460917 -0.02822050 0.07980536

O -2.00160885 -0.90401423 0.70253593

N 1.31173337 -1.84955382 -1.45855296

O 0.90599537 -2.06129837 -0.22872068

O 1.01267254 -0.64807713 -1.90241742

O 1.89516759 -2.66587043 -2.11239338

N 1.42944181 1.87323296 1.38941205

O 1.19666362 1.97164917 0.10606029

O 2.05178881 2.68035793 2.01808262

O 0.90790647 0.78779072 1.92986250

$Tm(NO_3)_3$

E= -14414.122148, E0 = -14414.077615, G= -1.1839 at T=298.150 K

Tm -0.06158710 0.00014260 0.00088560

N -2.77747059 0.01307720 0.05542854

O -2.06103277 0.99270809 -0.44849125

O -3.97501135 0.01877779 0.07950559

O -2.05071306 -0.97343326 0.53033495

N 1.36232030 -1.80947161 -1.43707752

O 1.31431973 -1.80446017 -0.12482589

O 0.65545577 -0.84974426 -1.98489296

O 1.99008846 -2.61049652 -2.06904101

N 1.43432212 1.79635060 1.38139117

O 1.33332145 1.79300046 0.07213638

O 2.09366560 2.59161592 1.98796666

O 0.74232137 0.84193325 1.95667970

$Yb(NO_3)_3$

E= -14906.893758, E0 = -14906.849325, G= -0.7877 at T=298.150 K

Yb -0.00056226 0.00007002 0.00003668

N -2.71518612 0.01231269 0.05396819

O -2.00053859 1.00079978 -0.42699859

O -3.91387081 0.01775904 0.07787323

O -1.99094391 -0.98272717 0.50622183

N 1.32025504 -1.85368240 -1.47983837

O	1.40966678	-1.77866948	-0.17383891
O	0.53043497	-0.94620472	-2.00129819
O	1.90390944	-2.67197704	-2.13340092
N	1.39508200	1.84132910	1.42586780
O	1.43151259	1.76596260	0.11734218
O	2.01197839	2.65392876	2.05557013
O	0.61826199	0.94109887	1.97849476

$Lu(NO_3)_3$

E= -15411.094022, E0 = -15411.048970, G= -0.9794 at T=298.150 K

Lu	0.00038829	0.00028653	0.00002928
N	-2.68301368	0.01198070	0.05306493
O	-1.96719754	0.86947393	-0.64548492
O	-3.87848854	0.01708121	0.07664666
O	-1.94756734	-0.85176516	0.72300607
N	1.30441868	-1.83314216	-1.46325481
O	1.18484581	-1.90169585	-0.15306827
O	0.71929699	-0.77273554	-1.98193359
O	1.88447928	-2.65040088	-2.11545086
N	1.37856102	1.82110512	1.41021049
O	1.20604563	1.89202631	0.10601298
O	1.99333358	2.63179636	2.03836322
O	0.80489767	0.76598930	1.95185888
